# Supplementary material for: Asymmetric hydrogenation of 1,1-diarylethylenes and benzophenones through a relay strategy
Source: Nat Commun. 2023 Apr 15;14:2170. doi: 10.1038/s41467-023-37882-2 (PMC10105712; doi:10.1038/s41467-023-37882-2)
Supplement: Supplementary file 1 — Supplementary Information [file 41467_2023_37882_MOESM1_ESM.pdf]

Supplementary Information

Asymmetric Hydrogenation of 1,1-Diarylethylenes and Benzophenones Through a Relay Strategy

Ke Li[§,1], Wen-Qiang Wu[§,1], Yunzhi Lin[1] and Hang Shi*[,1,2]

[1] Key Laboratory of Precise Synthesis of Functional Molecules of Zhejiang Province, Department of Chemistry, School of Science and Research Center for Industries of the Future, Westlake University, 600 Dunyu Road, Hangzhou 310030 (P. R. China).

[2] Institute of Natural Sciences, Westlake Institute for Advanced Study, 18 Shilongshan Road, Hangzhou 310024 (P. R. China).

[§] K.L. and W.-Q.W. contributed equally.

* Corresponding author. E-mail: shihang@westlake.edu.cn.

Table of Contents

Supplementary Methods **2**

1 General Information 2

2 Investigation of Arene Exchange 2

2.1 The regioisomeric ratios (r.r.) of 1a complexed with different metal species 2

2.2 Selective forming of $Cr(CO)_3$-complexed benzophenones 3

2.3 The preparation of substrates 9

3 Asymmetric Hydrogenation of 1,1-Diarylethenes and Benzophenones 12

3.1 Synthesis of ruthenium-catalysts 12

3.2 Asymmetric hydrogenation of 1a-Cr with different ruthenium-catalysts 13

3.3 General procedure for asymmetric hydrogenation of benzophenones and 1,1-diarylethenes 13

4 Computational Studies 29

5 NMR Chromatograms of Compounds 31

Supplementary References **104**

Supplementary Methods

1 General Information

Solvents: Anhydrous toluene was freshly distilled from sodium, other anhydrous solvents were all purchased from J&K Scientific Co., Ltd, stored in glovebox, and used without further purification.
Chromatography: Analytical thin layer chromatography was performed on 0.25 mm silica gel 60-F254. Visualization was carried out with UV light. Flash column chromatography was performed on 200–300 mesh silica gel, which purchased from Yantai Jiangyou Co., China.
Spectroscopy and Instruments: ^{1}H NMR was recorded on Bruker AVANCE NEO instrument (500 MHz, 600 MHz). Chemical shifts were quoted in parts per million (ppm) referenced with tetramethylsilane (δ = 0.00 ppm) or solvent peak (δ = 7.26 ppm in chloroform-*d*, δ = 2.05 ppm in Acetone-d_6). ^{13}C NMR spectra were recorded on Bruker AVANCE NEO instrument (126 MHz, 151 MHz), and were fully decoupled by broad band proton decoupling. Chemical shifts were reported in ppm referenced to the center peak of a triplet at 77.00 ppm of chloroform-d_1.^{19}F NMR spectra were recorded on Bruker AVANCE NEO instrument (471 MHz, 565 NMz). The following abbreviations (or combinations thereof) were used to explain multiplicities: s = singlet, d = doublet, dd = doublet of doublets, t = triplet, m = multiplet, br = broad. Coupling constants, *J*, were reported in Hertz unit (Hz). High-resolution mass spectra (HRMS) were recorded on a Waters Mass spectrometer using ESI/APCI-TOF (electrospray ionization-time of flight). Low-resolution mass spectras were recorded on Agilent 5977B GC/MSD.The enantiomeric excess value was detected on Agilent Infinity II SFC or HPLC system with Chiralpak columns.

2 Investigation of Arene Exchange

2.1 The regioisomeric ratios (r.r.) of 1a complexed with different metal species

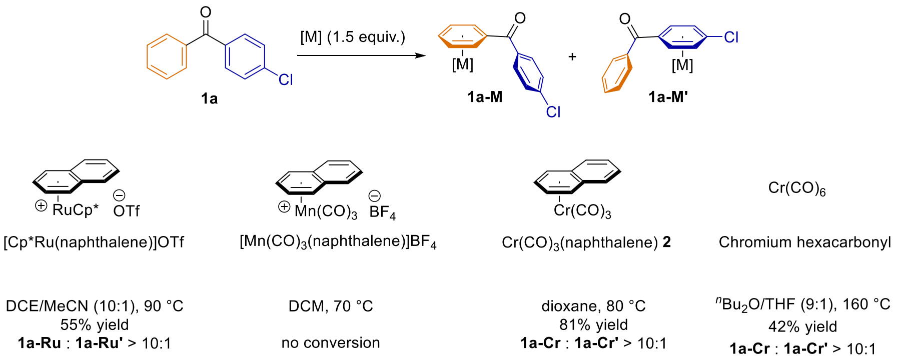

Supplementary Fig. 1 The regioisomeric ratios (r.r.) of 1a complexed with different metal species.

$Cr(CO)_6$ was commercially available and used after sublimation.
$[Mn(CO)_3(naphthalene)]BF_4$ was prepared by a modified procedure[1]. $Mn(CO)_5Br$ (1.0 g) and $AgBF_4$ (1.1 equiv) were dissolved in 50 mL of CH_2Cl_2 and refluxed for 1 h under N_2 in the dark. The naphthalene (2 equiv) in 10 mL of CH_2Cl_2 was then added and the reaction mixture refluxed overnight. After it was cooled to room temperature, the solution was filtered through Celite and and concentrated to ca. 20 mL in vacuo and the product precipitated with diethyl ether. The yellow $[Mn(CO)_3(naphthalene)]BF_4$ salts were washed repeatedly with ether and dried in vacuo. Yellow solid. **^{1}H NMR** (500 MHz, Acetone-d_6) δ 7.90 (s, 4H), 7.49 (s, 4H).
$Cr(CO)_3$(naphthalene) **2** was prepared by a modified procedure[2]. Chromium hexacarbonyl (17.6 g, 0.08 mol), KOH (32.0 g), degassed ethanol (80 ml), degassed *n*-butanol (80 ml) and degassed water (25 ml) were placed in a 500 ml two-necked flask provided with a stirrer and reflux condenser under N_2. The contents of the flask were warmed gradually on a metal bath to 150°C. Chromium hexacarbonyl which sublimed initially, was washed out later by the solvent and no more sublimation occurred during the reaction. The reaction solution turned gradually red. After 4 h of refluxing the mixture was cooled, 150 ml of concentrated aqueous ammonia solution added and the mixture was slowly stirred for 2 h. A yellow precipitate was filtered through a glass filter under the nitrogen atmospher, washed with aqueous ammonia and small quantities of methanol and ester.
To a cold (0 °C) ether solution (250 mL) of $[(NH_3)_3Cr(CO)_3]$ (60 mmol) and naphthalene (72 mmol) was added $BF_3{\cdot}OEt_2$ (240 mmol). The mixture was then allowed to warm to ambient temperature and stir for 8 days. Excess $BF_3{\cdot}OEt_2$ was quenched with 1 M HC1 solution. The solvent was removed from the dired organic layer and the residual naphthalene sublmimed from the crude mixture at 50 °C (15 mbar) to give the crude product. Pure product can be obtained by recrystallization from DCM/hexane. Red solid. **^{1}H NMR** (500 MHz, Chloroform-*d*) δ 7.56 (dd, *J* = 6.5, 3.2 Hz, 2H), 7.41 (dd, *J* = 6.5, 3.2 Hz, 2H), 6.13 (dd, *J* = 4.8, 2.8 Hz, 2H), 5.52 (dd, *J* = 5.0, 2.8 Hz, 2H).
[Cp*Ru(naphthalene)]OTf was prepared by a modified procedure for $[Cp^{*}Ru(naphthalene)]BF_4$[3]. A solution of $RuCl_3{\cdot}3H_2O$ (0.970 g, 4.0 mmol) in degassed ethanol (25 mL) was kept under reflux under N_2 in a two-neck round bottom flask until the color of the solution turned green (about 2 h). Pentamethylcyclopentadiene (3.0 mL, 20 mmol) and naphthalene (2.560 g, 20 mmol) were added to the reaction mixture, and the resulting solution was heated at reflux for 6 h. The reaction mixture was worked up in air. After the solvent was evaporated, water (15 mL) was added to the residue, and the solids were filtered off. The filtrate was treated with HOTf (5 mL) and extracted with CH_2Cl_2 (3 x 40 mL). The combined organic extracts

were dried over anhydrous Na_2SO_4 and evaporated to give crude product. Pure product can be obtained by recrystallization from DCM/hexane. Yellow solid. 1.556 g, 76% yield. **^{1}H NMR** (600 MHz, Chloroform-*d*) δ 7.64 (dd, J = 6.7, 3.1 Hz, 2H), 7.55 (dd, J = 6.7, 3.1 Hz, 2H), 6.59 (dd, J = 3.8, 2.1 Hz, 2H), 6.08 (dd, J = 4.1, 2.3 Hz, 2H), 1.67 (s, 15H). **^{13}C NMR** (151 MHz, Chloroform-*d*) δ 130.81, 127.72, 96.99, 93.87, 88.50, 85.47, 9.62. **^{19}F NMR** (565 MHz, Chloroform-*d*) δ -77.98. **HRMS-ESI** (m/z): [M-OTf]$^+$ calcd. for $[C_{20}H_{23}Ru]^+$, 365.0838, found, 365.0851.

General procedure:

*With [Cp*Ru(naphthalene)]OTf*: In a glovebox, a solution of [Cp*Ru(naphthalene)]OTf (0.15 mmol, 1.5 equiv.), **1a** (0.1 mmol, 1.0 equiv.) in DCE/MeCN (1 mL/0.1 ml) was stirred at room temperature for 2 min, then the sealed reaction vial was taken out of the glovebox and the reaction mixture was stirred at 90 °C in the dark for 24 h. Yield and regioisomeric ratio were determined by ^{1}H NMR using 1,1,2,2-tetrachloroethane as the internal standard.

With [Mn(CO)$_3$(naphthalene)]BF$_4$: In a glovebox, a solution of $[Mn(CO)_3(naphthalene)]BF_4$ (0.15 mmol, 1.5 equiv.), **1a** (0.1 mmol, 1.0 equiv.) in DCM (1 mL) was stirred at room temperature for 2 min, then the sealed reaction vial was taken out of the glovebox and the reaction mixture was stirred at 70 °C in the dark for 24 h. Yield and regioisomeric ratio were determined by ^{1}H NMR using CH_2Br_2 as the internal standard.

With Cr(CO)$_3$(naphthalene) ***2***: In a glovebox, a solution of $Cr(CO)_3$(naphthalene) **2** (0.15 mmol, 1.5 equiv.), **1a** (0.1 mmol, 1.0 equiv.) in 1,4-dioxane (1 mL) was stirred at room temperature for 2 min, then the sealed reaction vial was taken out of the glovebox and the reaction mixture was stirred at 80 °C in the dark for 24 h. Yield and regioisomeric ratio were determined by ^{1}H NMR using CH_2Br_2 as the internal standard.

With Cr(CO)$_6$: A flame-dried round bottom flask equipped with a magnetic stirrer and a reflux condenser was charged with**1a** (2.0 mmol, 1.0 equiv), $Cr(CO)_6$ (3. 0 mmol, 1.5 equiv) and anh. ^{n}Bu$_2$O and THF (9:1 v/v, 0.2 M) evacuated and backfilled with Ar. The resulting suspension was subjected freeze-pump-thaw cycles (3 × 30 min) and then refluxed (external temperature 160 °C) for 48 h. The solution was then cooled down to room temperature and filtered through a short pad of silica. The silica pad was washed with DCM (3 × 20 mL) and the organic layer was then concentrated in vacuo. Yield and regioisomeric ratio were determined by ^{1}H NMR using CH_2Br_2 as the internal standard.

[Cp*Ru(4-chlorobenzoylbenzene)]OTf (1a-Ru)

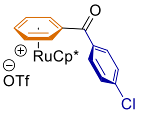

White solid. **^{1}H NMR** (600 MHz, Chloroform-*d*) δ 7.81 (d, J = 8.2 Hz, 2H), 7.55 (d, J = 8.3 Hz, 2H), 6.31 – 6.24 (m, 3H), 6.22 (d, J = 5.5 Hz, 2H), 1.94 (s, 15H). **^{13}C NMR** (151 MHz, Chloroform-*d*) δ 191.45, 141.02, 133.63, 131.00, 129.52, 98.13, 94.96, 89.28, 87.71, 87.43, 10.23. **^{19}F NMR** ^{19}F NMR (565 MHz, Chloroform-*d*) δ -78.03. **HRMS-ESI** (m/z): [M-OTf]$^+$ calcd. for $[C_{23}H_{24}ClORu]^+$, 453.0554, found, 453.0569.

2.2 Selective forming of Cr(CO)$_3$-complexed benzophenones

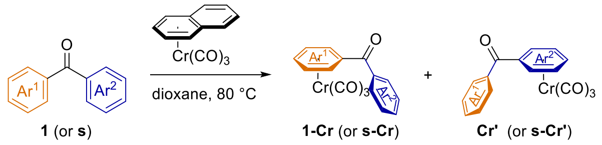

1-Cr (or s-Cr) yield		Ar^1		Ar^2	1-Cr (or s-Cr) yield		Ar^1		Ar^2
78%	**1a**	Ph		(4-Cl)C_6H_4	77%	**1l**	(3-OMe)C_6H_4		(4-F)C_6H_4
75%	**s1**	Ph		(2-F)C_6H_4	68%	**1m**	(4-OMe)C_6H_4		(4-Cl)C_6H_4
80%	**1c**	Ph		(3-F)C_6H_4	61%	**1n**	(4-OMe)C_6H_4		(4-Br)C_6H_4
80%	**1d**	Ph		(4-F)C_6H_4	76%	**1o**	(4-OMe)C_6H_4		(4-CF_3)C_6H_4
73%	**1e**	Ph		(4-Br)C_6H_4	77%	**1p**	(4-OMe)C_6H_4		(4-OCF_3)C_6H_4
72%	**1y**	Ph		(3-CF_3)C_6H_4	70%	**s3**	(4-OMe)C_6H_4		(3,5-bisCF_3)C_6H_3
78%	**1f**	Ph		(4-CF_3)C_6H_4	74%	**1v**	(4-OMe)C_6H_4		(3-F, 5-OMe)C_6H_3
82%	**1g**	Ph		(4-OCF_3)C_6H_4	47%	**1q**	(4-OTBS)C_6H_4	5.9:1	(4-Cl)C_6H_4
60%	**1h**	Ph	6.5:1	(4-CO_2Me)C_6H_4	80%	**1r**	(4-NMe_2)C_6H_4		(4-OMe)C_6H_4
38%	**1i**	Ph		[4-C(O)NMe_2]C_6H_4	84%	**1s**	(4-NMe_2)C_6H_4		(4-CO_2Me)C_6H_4
58%	**s2**	Ph	6.4:1	(4-OAc)C_6H_4	72%	**1j**	(4-NMe_2)C_6H_4		phenyl
35% (**1b-Cr'** 23%)	**1b**	Ph	1.5:1	(4-OMe)C_6H_4	85%	**1u**	(2-Me)C_6H_4		(2-CF_3)C_6H_4
80%	**1z**	Ph		(4-OTf)C_6H_4	65%	**1x**	(2-Me)C_6H_4		(4-Cl)C_6H_4
82%	**1aa**	Ph		(3,4-bisCl)C_6H_3	76%	**1t**	(4-Me)C_6H_4		(4-F)C_6H_4
84%	**1k**	Ph		(3,4-OCH_2O)C_6H_3	66%	**1ac**	(4-vinyl)C_6H_4		(4-F)C_6H_4
80%	**1ab**	Ph		2-naphthyl	85%	**1w**	[3,4-$(CH_2)_4$]C_6H_3		2-naphthyl

ratio > 10 : 1 | 10 : 1 ≥ ratio > 5 : 1 | 5 : 1 ≥ ratio

Supplementary Fig. 2 Selective formation of Cr(CO)$_3$-benzophenone complex.

General procedure:

For ^{1}H NMR analysis: In a glovebox, a solution of $Cr(CO)_3$(naphthalene) **2** (0.15 mmol, 1.5 equiv.), **1** (0.1 mmol, 1.0 equiv.) in 1,4-dioxane (1 mL) in a 4 ml glass vial was stirred at room temperature for 2 min, then the sealed reaction vial was taken out of the glovebox and the reaction mixture was stirred at 80 °C in the dark for 24 h. After cooling to room temperature, the reaction solution was analyzed by ^{1}H NMR directly and the regioisomeric ratios were determined by ^{1}H NMR.

For product purification: In a glovebox, a solution of $Cr(CO)_3$(naphthalene) **2** (0.3 mmol, 1.5 equiv.), **1** (0.2 mmol, 1.0 equiv.) in 1,4-dioxane (2 mL) in a 4 ml glass vial was stirred at room temperature for 2 min, then the sealed reaction vial was taken out of the glovebox and the reaction mixture was stirred at 80 °C in the dark for 24 h. After cooling to room temperature, the solution was evaporated under reduced pressure. The red residue was purified by column chromatography eluting with EtOAc/Petroleum ether (impurities that cannot be separated by column chromatography can be removed by recrystallization from diethyl ether and pentane).

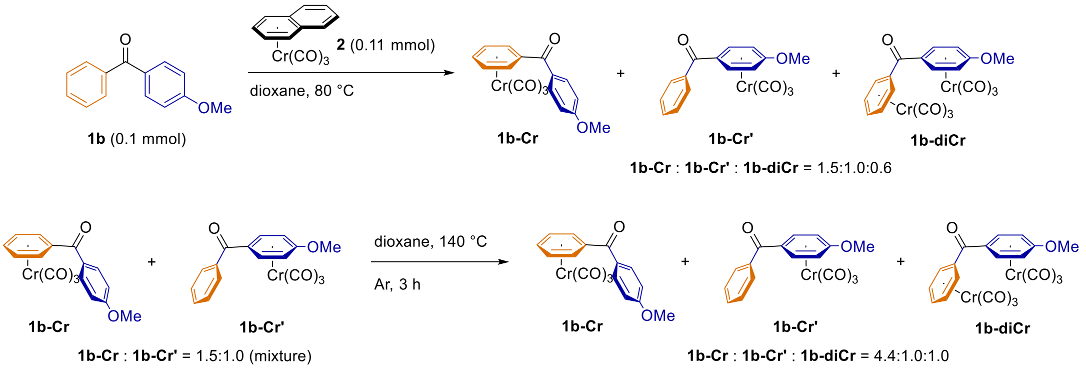

Supplementary Fig. 3 The regioisomeric ratios (r.r.) of 1b complexed with $Cr(CO)_3$.

In a glovebox, a solution of $Cr(CO)_3$(naphthalene) **2** (0.11 mmol, 1.1 equiv.), **1b** (0.1 mmol, 1.0 equiv.) in 1,4-dioxane (1 mL) was stirred at room temperature for 2 min, then the sealed reaction vial was taken out of the glovebox and the reaction mixture was stirred at 80 °C in the dark for 24 h. The regioisomeric ratio was determined by ^{1}H NMR.

Then, the solution was evaporated under reduced pressure. The red residue was purified by column chromatography to get the mixture (**1b-Cr** and **1b-Cr'**, 1.5:1.0). In a glovebox, a solution of the mixture, in 1,4-dioxane (0.5 mL) was stirred at room temperature for 2 min, then the sealed reaction vial was taken out of the glovebox and the reaction mixture was stirred at 140 °C in the dark for 3 h. The regioisomeric ratio was determined by ^{1}H NMR.

4-chlorobenzoylbenzene chromium tricarbonyl (1a-Cr)

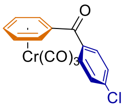

Known compound.[4] Red solid. 55.2 mg, 78% yield. The regioisomeric ratio was > 10:1. R_f = 0.33 (petroleum ether/ethyl acetate, PE/EA = 9:1 v/v). Elution with PE/EA = 20:1 to 5:1 v/v. **^{1}H NMR** (500 MHz, Chloroform-*d*) δ 7.74 (d, J = 8.6 Hz, 2H), 7.49 (d, J = 8.6 Hz, 2H), 5.99 (d, J = 6.8 Hz, 2H), 5.64 (t, J = 6.3 Hz, 1H), 5.33 (t, J = 6.5 Hz, 2H). **^{13}C NMR** (126 MHz, Chloroform-*d*) δ 230.33, 192.47, 138.91, 134.75, 130.09, 128.96, 95.60, 95.49, 94.57, 89.44. **HRMS-APCI** (m/z): [M]$^+$ calcd. for $[C_{16}H_{10}ClCrO_4]^+$, 352.9667, found, 352.9663.

2-fluorobenzoylbenzene chromium tricarbonyl (s1-Cr)

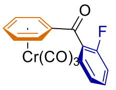

Red solid. 50.3 mg, 75% yield. The regioisomeric ratio was > 10:1. R_f = 0.45 (PE/EA = 9:1 v/v). Elution with PE/EA = 20:1 to 5:1 v/v. **^{1}H NMR** (500 MHz, Chloroform-*d*) δ 7.58–7.49 (m, 2H), 7.30 (td, J = 7.6, 0.9 Hz, 1H), 7.22–7.14 (m, 1H), 6.01 (d, J = 6.8 Hz, 2H), 5.67 (t, J = 6.3 Hz, 1H), 5.28 (t, J = 6.6 Hz, 2H). **^{13}C NMR** (126 MHz, Chloroform-*d*) δ 230.17, 190.55, 159.01 (d, J = 250.1 Hz), 133.11 (d, J = 8.1 Hz), 129.96 (d, J = 2.9 Hz), 125.35 (d, J = 16.3 Hz), 124.84 (d, J = 3.5 Hz), 116.12 (d, J = 21.4 Hz), 95.59 (d, J = 1.9 Hz), 95.19, 94.65, 88.97. **^{19}F NMR** (471 MHz, Chloroform-*d*) δ -111.81. **HRMS-APCI** (m/z): [M]$^+$ calcd. for $[C_{16}H_{10}CrFO_4]^+$, 336.9963, found, 336.9958.

3-fluorobenzoylbenzene chromium tricarbonyl (1c-Cr)

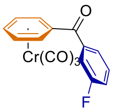

Red solid. 53.7 mg, 80% yield. The regioisomeric ratio was > 10:1. R_f = 0.33 (PE/EA = 9:1 v/v). Elution with PE/EA = 20:1 to 5:1 v/v. **^{1}H NMR** (500 MHz, Chloroform-*d*) δ 7.56 (d, J = 7.7 Hz, 1H), 7.53–7.43 (m, 2H), 7.30 (tdd, J = 8.3, 2.5, 0.8 Hz, 1H), 6.02 (d, J = 6.5 Hz, 2H), 5.65 (t, J = 6.2 Hz, 1H), 5.33 (t, J = 6.5 Hz, 2H). **^{13}C NMR** (126 MHz, Chloroform-*d*) δ 230.28, 192.29 (d, J = 2.2 Hz), 162.42 (d, J = 249.3 Hz), 138.40 (d, J = 6.5 Hz), 130.38 (d, J = 7.9 Hz), 124.32 (d, J = 3.2 Hz), 119.39 (d, J = 21.3 Hz), 115.63 (d, J = 22.9 Hz), 95.54, 95.15, 94.68, 89.39.**^{19}F NMR** (471 MHz, Chloroform-*d*) δ -110.98. **HRMS-APCI** (m/z): [M]$^+$ calcd. for $[C_{16}H_{10}CrFO_4]^+$, 336.9963, found, 336.9958.

4-fluorobenzoylbenzene chromium tricarbonyl (1d-Cr)

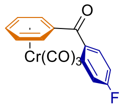

Red solid. 54.1 mg,80% yield. The regioisomeric ratio was > 10:1. R_f = 0.33 (PE/EA = 9:1 v/v). Elution with PE/EA = 20:1 to 5:1 v/v. **^{1}H NMR** (500 MHz, Chloroform-*d*) δ 7.84 (dd, *J* = 8.5, 5.4 Hz, 2H), 7.19 (t, *J* = 8.5 Hz, 2H), 6.00 (d, *J* = 6.5 Hz, 2H), 5.63 (t, *J* = 6.2 Hz, 1H), 5.34 (t, *J* = 6.4 Hz, 2H). **^{13}C NMR** (126 MHz, Chloroform-*d*) δ 230.42, 192.16, 165.30 (d, *J* = 254.5 Hz), 132.65 (d, *J* = 3.3 Hz), 131.33 (d, *J* = 9.2 Hz), 115.85 (d, *J* = 21.8 Hz), 96.11, 95.53, 94.51, 89.48. **^{19}F NMR** (471 MHz, $CDCl_3$) δ -105.48. **HRMS-APCI** (m/z): $[M]^+$ calcd. for $[C_{16}H_{10}CrFO_4]^+$, 336.9963, found, 336.9964.

4-bromobenzoylbenzene chromium tricarbonyl (1e-Cr)

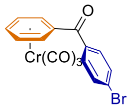

Known compound[4]. Red solid. 57.7 mg, 73% yield. The regioisomeric ratio was > 10:1. R_f = 0.33 (PE/EA = 9:1 v/v). Elution with PE/EA = 20:1 to 5:1 v/v. **^{1}H NMR** (500 MHz, Chloroform-*d*) δ 7.65 (s, 4H), 5.99 (d, *J* = 6.6 Hz, 2H), 5.64 (t, *J* = 6.4 Hz, 1H), 5.33 (t, *J* = 6.5 Hz, 2H). **^{13}C NMR** (126 MHz, Chloroform-*d*) δ 230.31, 192.64, 135.20, 131.93, 130.17, 127.41, 95.48, 94.58, 89.43. **HRMS-APCI** (m/z): $[M]^+$ calcd. for $[C_{16}H_{10}BrCrO_4]^+$, 396.9162, found, 396.9165.

3-(trifluoromethyl)benzoylbenzene chromium tricarbonyl (1y-Cr)

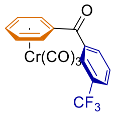

Orange solid. 55.8 mg, 72% yield. The regioisomeric ratio was > 10:1. R_f = 0.33 (PE/EA = 9:1 v/v) Elution with PE/EA = 20:1 to 5:1 v/v. **^{1}H NMR** (500 MHz, Chloroform-*d*) δ 8.03 (s, 1H), 7.97 (d, *J* = 7.6 Hz, 1H), 7.86 (d, *J* = 7.7 Hz, 1H), 7.66 (t, *J* = 7.7 Hz, 1H), 5.99 (d, *J* = 6.4 Hz, 2H), 5.67 (t, *J* = 6.1 Hz, 1H), 5.34 (t, *J* = 6.4 Hz, 2H). **^{13}C NMR** (126 MHz, Chloroform-*d*) δ 230.12, 192.43, 137.18, 131.76, 131.23 (q, *J* = 33.5 Hz), 129.36, 128.84 (q, *J* = 3.8 Hz), 125.34 (q, *J* = 4.5 Hz), 123.49 (q, *J* = 272.5 Hz), 95.39, 94.68, 89.42. **^{19}F NMR** (471 MHz, Chloroform-*d*) δ -62.76. **HRMS-APCI** (m/z): $[M]^+$ calcd. for $[C_{17}H_{10}CrF_3O_4]^+$, 386.9931, found, 386.9927.

4-(trifluoromethyl)benzoylbenzene chromium tricarbonyl (1f-Cr)

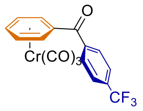

Red solid. 60.0 mg, 78% yield. The regioisomeric ratio was > 10:1. R_f = 0.33 (PE/EA = 9:1 v/v). Elution with PE/EA = 20:1 to 5:1 v/v. **^{1}H NMR** (500 MHz, Chloroform-*d*) δ 7.86 (d, *J* = 8.1 Hz, 2H), 7.78 (d, *J* = 8.2 Hz, 2H), 5.99 (d, *J* = 6.4 Hz, 2H), 5.67 (t, *J* = 6.2 Hz, 1H), 5.33 (t, *J* = 6.5 Hz, 2H). **^{13}C NMR** (126 MHz, Chloroform-*d*) δ 230.13, 192.82, 139.74, 133.70 (q, *J* = 32.9 Hz), 128.73, 125.68 (q, *J* = 3.7 Hz), 123.51 (q, *J* = 272.7 Hz), 95.44, 94.78, 94.47, 89.41. **^{19}F NMR** (471 MHz, Chloroform-*d*) δ -63.06. **HRMS-APCI** (m/z): $[M]^+$ calcd. for $[C_{17}H_{10}CrF_3O_4]^+$, 386.9931, found, 386.9931.

4-(trifluoromethyloxy)benzoylbenzene chromium tricarbonyl (1g-Cr)

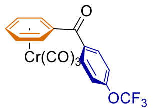

Orange solid. 66.1 mg, 82% yield. The regioisomeric ratio was > 10:1. R_f = 0.33 (PE/EA = 9:1 v/v). Elution with PE/EA = 20:1 to 5:1 v/v. **^{1}H NMR** (500 MHz, Chloroform-*d*) δ 7.85 (d, *J* = 8.7 Hz, 2H), 7.34 (d, *J* = 8.1 Hz, 2H), 6.00 (d, *J* = 6.1 Hz, 2H), 5.65 (t, *J* = 6.2 Hz, 1H), 5.34 (t, *J* = 6.5 Hz, 2H). **^{13}C NMR** (126 MHz, Chloroform-*d*) δ 230.31, 192.25, 152.09 (q, *J* = 1.5 Hz), 134.75, 130.64, 120.59, 120.28 (q, *J* = 258.9 Hz), 95.46, 94.59, 89.49. **^{19}F NMR** (471 MHz, Chloroform-*d*) δ -57.60. **HRMS-APCI** (m/z): $[M]^+$ calcd. for $[C_{17}H_{10}CrF_3O_5]^+$, 402.9880, found, 402.9885.

4-(methoxycarbonyl)benzoylbenzene chromium tricarbonyl (1h-Cr)

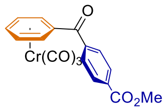

Red solid. 45.0 mg, 60% yield. The regioisomeric ratio was 6.5:1. R_f = 0.50 (PE/EA = 4:1 v/v). Elution with PE/EA = 10:1 to 3:1 v/v. **^{1}H NMR** (500 MHz, Chloroform-*d*) δ 8.17 (d, *J* = 8.3 Hz, 2H), 7.80 (d, *J* = 8.3 Hz, 2H), 6.01 (s, 2H), 5.66 (t, *J* = 6.2 Hz, 1H), 5.32 (t, *J* = 6.5 Hz, 2H), 3.97 (s, 3H). **^{13}C NMR** (126 MHz, Chloroform-*d*) δ 230.19, 193.21, 166.07, 140.31, 133.21, 129.78, 128.34, 95.53, 94.77, 94.75, 89.34, 52.50. **HRMS-APCI** (m/z): $[M]^+$ calcd. for $[C_{18}H_{13}CrO_6]^+$, 377.0112, found, 377.0107.

4-(dimethylcarbamoyl)benzoylbenzene chromium tricarbonyl (1i-Cr)

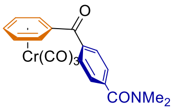

Orange solid. 29.7 mg, 38% yield. The regioisomeric ratio was > 10:1. R_f = 0.20 (PE/EA = 1:1 v/v). Elution with PE/EA/DCM = 2:1:1 to 1:1:1 v/v/v. **^{1}H NMR** (500 MHz, Chloroform-*d*) δ 7.80 (d, *J* = 8.0 Hz, 2H), 7.55 (d, *J* = 8.0 Hz, 2H), 6.02 (d, *J* = 6.4 Hz, 2H), 5.65 (t, *J* = 6.2 Hz, 1H), 5.33 (t, *J* = 6.4 Hz, 2H), 3.15 (s, 3H), 3.00 (s, 3H). **^{13}C NMR** (126 MHz, Chloroform-*d*) δ 230.31, 193.20, 170.30, 140.10, 137.36, 128.67, 127.22, 95.58, 95.23, 94.67, 89.46, 39.42, 35.30. **HRMS-APCI** (m/z): $[M]^+$ calcd. for $[C19H16CrNO5]^+$, 390.0428, found, 390.0418.

4-(acetoxy)benzoylbenzene chromium tricarbonyl (s2-Cr)

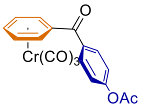

Red solid. 43.6 mg, 58% yield. The regioisomeric ratio was 6.4:1. R_f = 0.35 (PE/EA = 4:1 v/v). Elution with PE/EA = 10:1 to 3:1 v/v. (1.1 equiv **2**) **^{1}H NMR** (500 MHz, Chloroform-*d*) δ 7.84 (d, *J* = 8.6 Hz, 2H), 7.24 (d, *J* = 8.7 Hz, 2H), 6.02 (d, *J* = 6.0 Hz, 2H), 5.62 (t, *J* = 6.2 Hz, 1H), 5.33 (t, *J* = 6.5 Hz, 2H), 2.35 (s, 3H). **^{13}C NMR** (126 MHz, Chloroform-*d*) δ 230.45, 192.48, 168.85, 153.87, 133.90, 130.37, 121.88, 96.11, 95.58, 94.48, 89.50, 21.14. **HRMS-APCI** (m/z): $[M]^+$ calcd. for $[C_{18}H_{13}CrO_6]^+$, 377.0112, found, 377.0105.

4-(methoxy)benzoylbenzene chromium tricarbonyl (1b-Cr)

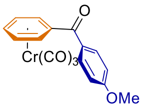

Known compound.[4] Yellow solid. 24.3 mg, 35% yield. The regioisomeric ratio was 1.5:1.0. R_f = 0.20 (PE/EA = 9:1 v/v). Elution with PE/EA = 20:1 to 5:1 v/v. (1.1 equiv **2**) **^{1}H NMR** (500 MHz, Chloroform-*d*) δ 7.85 (d, *J* = 8.4 Hz, 2H), 6.98 (d, *J* = 8.4 Hz, 2H), 6.01 (d, *J* = 6.5 Hz, 2H), 5.59 (t, *J* = 6.2 Hz, 1H), 5.34 (t, *J* = 6.4 Hz, 2H), 3.90 (s, 3H). **^{13}C NMR** (126 MHz, Chloroform-*d*) δ 230.80, 191.91, 163.38, 131.43, 128.85, 113.89, 97.95, 95.65, 94.28, 89.60, 55.54. **HRMS-APCI** (m/z): $[M]^+$ calcd. for $[C_{17}H_{13}CrO_5]^+$, 349.0163, found, 349.0156.

4-benzoylanisole chromium tricarbonyl (1b-Cr')

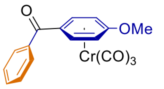

Orange solid. 16.2 mg, 23% yield. R_f = 0.23 (PE/EA = 9:1 v/v). Elution with PE/EA = 20:1 to 5:1 v/v. **^{1}H NMR** (500 MHz, Chloroform-*d*) δ 7.72 (d, *J* = 7.5 Hz, 2H), 7.58 (t, *J* = 5.1 Hz, 1H), 7.50 (d, *J* = 6.4 Hz, 2H), 6.19 (d, *J* = 6.9 Hz, 2H), 5.18 (d, *J* = 6.9 Hz, 2H), 3.78 (s, 3H). **^{13}C NMR** (126 MHz, Chloroform-*d*) δ 230.48, 192.85, 144.16, 136.76, 132.16, 128.55, 128.38, 96.20, 91.42, 76.60, 55.88. **HRMS-APCI** (m/z): $[M]^+$ calcd. for $[C_{17}H_{13}CrO_5]^+$, 349.0163, found, 349.0158.

(4-methoxyphenyl chromium tricarbonyl)(phenyl chromium tricarbonyl)methanone (1b-diCr)

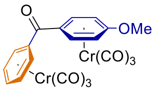

Red solid. 10.6 mg, 11% yield. R_f = 0.35 (PE/EA = 4:1 v/v). Elution with PE/EA = 4:1 v/v. **^{1}H NMR** (600 MHz, Chloroform-*d*) δ 6.27 (d, *J* = 6.2 Hz, 2H), 6.06 (d, *J* = 5.2 Hz, 2H), 5.61 (d, *J* = 6.6 Hz, 1H), 5.33 (d, *J* = 7.1 Hz, 2H), 5.20 (d, *J* = 5.3 Hz, 2H), 3.80 (s, 3H). **^{13}C NMR** (151 MHz, Chloroform-*d*) δ 230.27, 230.19, 188.53, 143.92, 96.58, 95.43, 94.81, 94.32, 91.02, 89.10, 76.38, 55.97. **HRMS-APCI** (m/z): $[M]^+$ calcd. for $[C_{20}H_{13}Cr_2O_8]^+$, 484.9415, found, 484.9421.

4-(((Trifluoromethyl)sulfonyl)oxy)benzoylbenzene chromium tricarbonyl (1z-Cr)

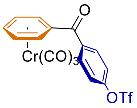

Orange solid. 74.4 mg, 80% yield. The regioisomeric ratio was > 10:1. R_f = 0.33 (PE/EA = 9:1 v/v). Elution with PE/EA = 20:1 to 5:1 v/v. **^{1}H NMR** (500 MHz, Chloroform-*d*) δ 7.89 (d, *J* = 8.8 Hz, 2H), 7.43 (d, *J* = 8.7 Hz, 2H), 5.99 (d, *J* = 6.1 Hz, 2H), 5.67 (t, *J* = 6.3 Hz, 1H), 5.35 (t, *J* = 6.5 Hz, 2H). **^{13}C NMR** (126 MHz, Chloroform-*d*) δ 230.13, 192.01, 151.83, 136.56, 130.80, 121.78, 118.69 (d, *J* = 320.8 Hz), 95.37, 94.70, 94.67, 89.46. **^{19}F NMR** (471 MHz, Chloroform-*d*) δ -72.66. **HRMS-APCI** (m/z): $[M]^+$ calcd. for $[C_{17}H_{10}CrF_3O_7S]^+$, 466.9499, found, 466.9501.

3,4-bischlorobenzoylbenzene chromium tricarbonyl (1aa-Cr)

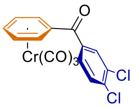

Red solid. 63.4 mg, 82% yield. The regioisomeric ratio was > 10:1. R_f = 0.33 (PE/EA = 9:1 v/v). Elution with PE/EA = 20:1 to 5:1 v/v. **^{1}H NMR** (500 MHz, Chloroform-*d*) δ 7.88 (d, *J* = 1.8 Hz, 1H), 7.63 (dd, *J* = 8.3, 1.9 Hz, 1H), 7.59 (d, *J* = 8.2 Hz, 1H), 5.98 (d, *J* = 6.3 Hz, 2H), 5.66 (t, *J* =

6.2 Hz, 1H), 5.34 (t, J = 6.5 Hz, 2H). **^{13}C NMR** (126 MHz, Chloroform-*d*) δ 230.10, 191.28, 137.07, 136.00, 133.30, 130.78, 130.53, 127.73, 95.36, 94.73, 94.66, 89.36. **HRMS-APCI** (m/z): [M]$^+$ calcd. for $[C_{16}H_9Cl_2CrO_4]^+$, 386.9278, found, 386.9271.

(benzo[d][1,3]dioxole-5-carbonyl)benzene chromium tricarbonyl (1k-Cr)

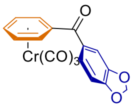

Yellow solid. 61.1mg, 84% yield. The regioisomeric ratio was > 10:1. R_f = 0.55 (PE/EA = 4:1 v/v). Elution with PE/EA = 10:1 to 3:1 v/v. **^{1}H NMR** (500 MHz, Chloroform-*d*) δ 7.44 (dd, J = 8.1, 1.4 Hz, 1H), 7.32 (d, J = 1.3 Hz, 1H), 6.89 (d, J = 8.1 Hz, 1H), 6.08 (s, 2H), 6.01 (d, J = 6.4 Hz, 2H), 5.59 (t, J = 6.2 Hz, 1H), 5.33 (t, J = 6.4 Hz, 2H). **^{13}C NMR** (126 MHz, Chloroform-*d*) δ 230.68, 191.50, 151.67, 148.08, 130.48, 125.18, 109.20, 107.98, 101.97, 97.58, 95.65, 94.37, 89.44. **HRMS-APCI** (m/z): [M]$^+$ calcd. for $[C_{17}H_{11}CrO_6]^+$, 362.9955, found, 362.9949.

2-naphthoylbenzene chromium tricarbonyl (1ab-Cr)

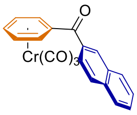

Known compound.[4] Orange solid. 58.7 mg, 80% yield. The regioisomeric ratio was > 10:1. R_f = 0.35 (PE/EA = 9:1 v/v). Elution with PE/EA = 20:1 to 5:1 v/v. **^{1}H NMR** (500 MHz, Chloroform-*d*) δ 8.39 (s, 1H), 7.97 (t, J = 9.1 Hz, 2H), 7.92 (d, J = 8.0 Hz, 1H), 7.84 (d, J = 8.3 Hz, 1H), 7.61 (dt, J = 20.6, 7.0 Hz, 2H), 6.10 (d, J = 6.5 Hz, 2H), 5.65 (t, J = 6.2 Hz, 1H), 5.35 (t, J = 6.4 Hz, 2H). **^{13}C NMR** (126 MHz, Chloroform-*d*) δ 230.62, 193.56, 135.14, 133.55, 132.16, 129.97, 129.27, 128.70, 128.44, 127.84, 127.12, 124.91, 96.50, 95.94, 94.62, 89.51. **HRMS-APCI** (m/z): [M]$^+$ calcd. for $[C_{20}H_{13}CrO_4]^+$, 369.0213, found, 369.0208.

3-(4-fluorobenzoyl)anisole chromium tricarbonyl (1l-Cr)

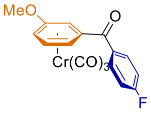

Red solid. 56.5 mg, 77% yield. The regioisomeric ratio was > 10:1. R_f = 0.40 (PE/EA = 4:1 v/v). Elution with PE/EA = 10:1 to 3:1 v/v. **^{1}H NMR** (500 MHz, Chloroform-*d*) δ 7.86 (dd, J = 8.7, 5.3 Hz, 2H), 7.19 (t, J = 8.6 Hz, 2H), 5.69 (dd, J = 2.2, 1.2 Hz, 1H), 5.57 (t, J = 6.6 Hz, 1H), 5.44 (d, J = 6.4 Hz, 1H), 5.41 (dd, J = 6.8, 2.0 Hz, 1H), 3.76 (s, 3H). **^{13}C NMR** (126 MHz, Chloroform-*d*) δ 231.60, 193.51, 165.40 (d, J = 254.8 Hz), 141.25, 132.69 (d, J = 3.2 Hz), 131.50 (d, J = 9.0 Hz), 115.84 (d, J = 21.9 Hz), 97.86, 92.30, 89.57, 81.19, 78.01, 56.10. **^{19}F NMR** (471 MHz, Chloroform-*d*) δ -105.20. **HRMS-APCI** (m/z): [M]$^+$ calcd. for $[C_{17}H_{12}CrFO_5]^+$, 367.0068, found, 367.0070.

4-(4-chlorobenzoyl)anisole chromium tricarbonyl (1m-Cr)

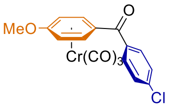

Yellow solid. 52.0 mg, 68% yield. The regioisomeric ratio was > 10:1. R_f = 0.33 (PE/EA = 9:1 v/v). Elution with PE/EA = 20:1 to 5:1 v/v. **^{1}H NMR** (500 MHz, Chloroform-*d*) δ 7.69 (d, J = 8.5 Hz, 2H), 7.48 (d, J = 8.5 Hz, 2H), 6.15 (d, J = 7.1 Hz, 2H), 5.18 (d, J = 7.2 Hz, 2H), 3.79 (s, 3H). **^{13}C NMR** (126 MHz, Chloroform-*d*) δ 230.29, 191.66, 144.16, 138.61, 135.01, 129.82, 128.91, 95.97, 90.94, 76.58, 55.92. **HRMS-APCI** (m/z): [M]$^+$ calcd. for $[C_{17}H_{12}ClCrO_5]^+$, 382.9773, found, 382.9772.

4-(4-bromobenzoyl)anisole chromium tricarbonyl (1n-Cr)

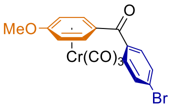

Yellow solid. 52.4 mg, 61% yield. The regioisomeric ratio was > 10:1. R_f = 0.33 (PE/EA = 9:1 v/v). Elution with PE/EA = 20:1 to 5:1 v/v. **^{1}H NMR** (500 MHz, Chloroform-*d*) δ 7.64 (d, J = 8.2 Hz, 2H), 7.61 (d, J = 8.2 Hz, 2H), 6.15 (d, J = 6.8 Hz, 2H), 5.18 (d, J = 6.8 Hz, 2H), 3.79 (s, 3H). **^{13}C NMR** (126 MHz, Chloroform-*d*) δ 230.27, 191.81, 144.17, 135.46, 131.88, 129.90, 127.09, 95.94, 90.83, 76.58, 55.93. **HRMS-APCI** (m/z): [M]$^+$ calcd. for $[C_{17}H_{12}BrCrO_5]^+$, 426.9268, found, 426.9271.

4-(4-(trifluomethyl)benzoyl)anisole chromium tricarbonyl (1o-Cr)

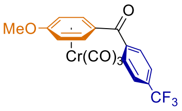

Orange solid. 63.4 mg, 76% yield. The regioisomeric ratio was > 10:1. R_f = 0.33 (PE/EA = 9:1 v/v). Elution with PE/EA = 20:1 to 5:1 v/v. **^{1}H NMR** (500 MHz, Chloroform-*d*) δ 7.81 (d, J = 7.9 Hz, 2H), 7.76 (d, J = 8.1 Hz, 2H), 6.14 (d, J = 6.9 Hz, 2H), 5.19 (d, J = 7.0 Hz, 2H), 3.79 (s, 3H). **^{13}C NMR** (126 MHz, Chloroform-*d*) δ 230.09, 192.01, 144.27, 140.03, 133.51 (q, J = 33.5, 32.3 Hz), 128.45, 125.65, 123.54 (q, J = 273.2, 271.8 Hz), 95.77, 90.02, 76.66, 55.95. **^{19}F NMR** (471 MHz, Chloroform-*d*) δ -63.03. **HRMS-APCI** (m/z): [M]$^+$ calcd. for $[C_{18}H_{12}CrF_3O_5]^+$, 417.0036, found,

417.0039.

4-(4-(trifluoromethoxy)benzoyl)anisole chromium tricarbonyl (1p-Cr)

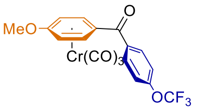

Orange solid. 66.2 mg, 77% yield. The regioisomeric ratio was > 10:1. R_f = 0.35 (PE/EA = 9:1 v/v). Elution with PE/EA = 20:1 to 5:1 v/v. **^{1}H NMR** (500 MHz, Chloroform-*d*) δ 7.80 (d, J = 8.3 Hz, 2H), 7.33 (d, J = 8.2 Hz, 2H), 6.16 (d, J = 6.9 Hz, 2H), 5.19 (d, J = 6.9 Hz, 2H), 3.79 (s, 3H). **^{13}C NMR** (126 MHz, Chloroform-*d*) δ 230.26, 191.45, 151.91, 144.18, 135.03, 130.35, 120.59, 120.29 (q, J = 258.6 Hz), 95.94, 90.82, 76.61, 55.93. **^{19}F NMR** (471 MHz, Chloroform-*d*) δ -57.61. **HRMS-APCI** (m/z): [M]$^+$ calcd. for $[C_{18}H_{12}CrF_3O_6]^+$, 432.9986, found, 432.9992.

4-(3,5-bis(trifluomethyl)benzoyl)anisole chromium tricarbonyl (s3-Cr)

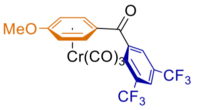

Orange solid. 67.9 mg, 70% yield. The regioisomeric ratio was > 10:1. R_f = 0.40 (PE/EA = 9:1 v/v). Elution with PE/EA = 20:1 to 5:1 v/v. **^{1}H NMR** (500 MHz, Chloroform-*d*) δ 8.16 (s, 2H), 8.09 (s, 1H), 6.09 (d, J = 5.5 Hz, 2H), 5.22 (d, J = 5.4 Hz, 2H), 3.81 (s, 3H). **^{13}C NMR** (126 MHz, Chloroform-*d*) δ 229.69, 190.21, 144.23, 138.61, 132.34 (q, J = 34.1 Hz), 128.26, 125.42, 122.73 (q, J = 273.4 Hz), 95.35, 89.00, 76.70, 56.02. **^{19}F NMR** (471 MHz, Chloroform-*d*) δ -62.85. **HRMS-APCI** (m/z): [M]$^+$ calcd. for $[C_{19}H_{11}CrF_6O_5]^+$, 484.9910, found, 484.9917.

4-(3-methoxy-5-fluorobenzoyl)anisole chromium tricarbonyl (1v-Cr)

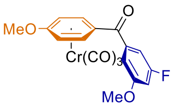

Orange solid. 58.3 mg, 74% yield. The regioisomeric ratio was > 10:1. R_f = 0.35 (PE/EA = 4:1 v/v). Elution with PE/EA = 10:1 to 3:1 v/v. **^{1}H NMR** (500 MHz, Chloroform-*d*) δ 7.02 (s, 1H), 6.98 (d, J = 8.0 Hz, 1H), 6.82 (d, J = 10.2 Hz, 1H), 6.19 (d, J = 6.9 Hz, 2H), 5.19 (d, J = 6.9 Hz, 2H), 3.86 (s, 3H), 3.79 (s, 3H). **^{13}C NMR** (126 MHz, Chloroform-*d*) δ 230.26, 191.54 (d, J = 2.7 Hz), 164.06, 162.09, 161.08 (d, J = 10.9 Hz), 144.23, 139.00 (d, J = 8.4 Hz), 109.47 (d, J = 2.8 Hz), 107.67 (d, J = 23.5 Hz), 105.54 (d, J = 24.8 Hz), 95.95, 90.41, 76.61, 55.93. **^{19}F NMR** (471 MHz, Chloroform-*d*) δ -109.80. **HRMS-APCI** (m/z): [M]$^+$ calcd. for $[C_{18}H_{14}CrFO_6]^+$, 397.0174, found, 397.0173.

4-(4-chlorobenzoyl)(((tert-butyldimethylsilyl)oxy)benzene) chromium tricarbonyl (1q-Cr)

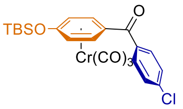

Orange solid. 45.0 mg, 47% yield. The regioisomeric ratio was > 10:1. R_f = 0.70 (PE/EA = 9:1 v/v). Elution with PE/EA = 25:1 to 10:1 v/v. **^{1}H NMR** (600 MHz, Chloroform-*d*) δ 7.68 (d, J = 8.5 Hz, 2H), 7.47 (d, J = 8.5 Hz, 2H), 6.11 (d, J = 7.1 Hz, 2H), 5.10 (d, J = 7.1 Hz, 2H), 0.98 (s, 9H), 0.31 (s, 6H). **^{13}C NMR** (151 MHz, Chloroform-*d*) δ 230.58, 191.81, 140.88, 138.51, 135.09, 129.80, 128.87, 96.23, 90.59, 81.68, 25.28, 18.04, -4.43. **HRMS-APCI** (m/z): [M]$^+$ calcd. for $[C_{22}H_{24}ClCrO_5Si]^+$, 483.0481, found, 483.0483.

4-(4-methoxybenzoyl)nn-dimethylaniline chromium tricarbonyl (1r-Cr)

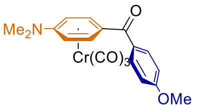

Yellow solid. 62.6 mg, 80% yield. The regioisomeric ratio was > 10:1. (1.1 equiv **2**) R_f = 0.45 (PE/EA = 2:1 v/v). Elution with PE/EA = 4:1 to 2:1 v/v. **^{1}H NMR** (500 MHz, Chloroform-*d*) δ 7.82 (d, J = 8.6 Hz, 2H), 6.97 (d, J = 8.6 Hz, 2H), 6.24 (d, J = 7.2 Hz, 2H), 4.87 (d, J = 7.2 Hz, 2H), 3.88 (s, 3H), 2.98 (s, 6H). **^{13}C NMR** (126 MHz, Chloroform-*d*) δ 232.27, 191.89, 162.88, 136.12, 130.99, 129.53, 113.71, 98.25, 90.75, 73.03, 55.47, 39.80. **HRMS-APCI** (m/z): [M]$^+$ calcd. for $[C_{19}H_{18}CrNO_5]^+$, 392.0585, found, 392.0585.

η^6-4-(4-methoxycarbonyl)nn-dimethylaniline chromium tricarbonyl (1s-Cr)

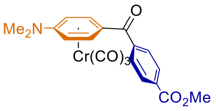

Red solid. 70.2 mg, 84% yield. The regioisomeric ratio was > 10:1. R_f = 0.40 (PE/EA = 2:1 v/v). Elution with PE/EA = 4:1 to 2:1 v/v. (1.1 equiv **2**) **^{1}H NMR** (500 MHz, Chloroform-*d*) δ 8.14 (d, J = 8.4 Hz, 2H), 7.76 (d, J = 8.4 Hz, 2H), 6.18 (d, J = 7.5 Hz, 2H), 4.86 (d, J = 7.6 Hz, 2H), 3.96 (s, 3H), 2.99 (s, 6H). **^{13}C NMR** (126 MHz, Chloroform-*d*) δ 231.69, 193.09, 166.23, 141.15, 136.37, 132.66, 129.67, 127.99, 97.68, 88.41, 73.08, 52.42, 39.82. **HRMS-APCI** (m/z): [M]$^+$ calcd. for $[C_{20}H_{18}CrNO_6]^+$, 420.0534, found, 420.0538.

4-(benzoyl)nn-dimethylaniline chromium tricarbonyl (1j-Cr)

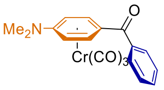

Orange solid. 51.8 mg, 72% yield. The regioisomeric ratio was > 10:1. R_f = 0.35 (PE/EA = 4:1 v/v). Elution with PE/EA = 10:1 to 3:1 v/v. (1.1 equiv **2**) **^{1}H NMR** (500 MHz, Chloroform-*d*) δ 7.74 (d, J = 7.1 Hz, 2H), 7.56 (t, J = 7.4 Hz, 1H), 7.48 (t, J = 7.5 Hz, 2H), 6.23 (d, J = 7.5 Hz, 2H), 4.86 (d, J = 7.5 Hz, 2H), 2.99 (s, 6H). **^{13}C NMR** (126 MHz, Chloroform-*d*) δ 231.98, 193.58, 137.23, 136.22, 131.78, 128.44, 128.29, 98.02, 89.48, 73.08, 39.82. **HRMS-APCI** (m/z): [M]$^+$ calcd. for $[C_{18}H_{16}CrNO_4]^+$, 362.0479, found, 362.0476.

2-(2-(trifluoromethyl)benzoyl)toluene chromium tricarbonyl (1u-Cr)

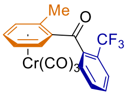

Orange solid. 67.9 mg, 85% yield. The regioisomeric ratio was > 10:1. R_f = 0.55 (PE/EA = 9:1 v/v). Elution with PE/EA = 20:1 to 5:1 v/v. **^{1}H NMR** (500 MHz, Chloroform-*d*) δ 7.75 (d, J = 7.7 Hz, 1H), 7.68 (t, J = 7.3 Hz, 1H), 7.65–7.58 (m, 2H), 5.69 (t, J = 6.2 Hz, 1H), 5.45 (d, J = 6.5 Hz, 1H), 5.11 (d, J = 6.3 Hz, 1H), 5.03 (t, J = 6.4 Hz, 1H), 2.53 (s, 3H). **^{13}C NMR** (126 MHz, Chloroform-*d*) δ 230.63, 195.35, 137.41 (q, J = 2.2 Hz), 131.95, 130.25, 128.69, 127.40 (q, J = 32.1 Hz), 126.61 (q, J = 4.6 Hz), 123.59 (q, J = 274.0 Hz), 112.51, 98.85, 96.03, 94.95 (q, J = 1.7 Hz), 92.01, 86.95, 21.11. **^{19}F NMR** (471 MHz, Chloroform-*d*) δ -57.58. **HRMS-APCI** (m/z): [M]$^+$ calcd. for $[C_{18}H_{12}CrF_3O_4]^+$, 401.0087, found, 401.0083.

2-(4-chlorobenzoyl)toluene chromium tricarbonyl (1x-Cr)

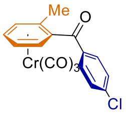

Orange solid. 47.8 mg, 65% yield. The regioisomeric ratio was > 10:1. R_f = 0.40 (PE/EA = 9:1 v/v). Elution with PE/EA = 20:1 to 5:1 v/v. **^{1}H NMR** (600 MHz, Chloroform-d) δ 7.84 (d, J = 8.1 Hz, 2H), 7.47 (d, J = 8.2 Hz, 2H), 5.61–5.54 (m, 2H), 5.17–5.12 (m, 2H), 2.25 (s, 3H). **^{13}C NMR** (151 MHz, Chloroform-d) δ 231.27, 192.70, 140.11, 135.24, 130.91, 129.07, 108.98, 103.93, 95.69, 94.72, 90.95, 86.56, 20.00. **HRMS-APCI** (m/z): [M]$^+$ calcd. for $[C_{17}H_{12}ClCrO_4]^+$, 366.9824, found, 366.9826.

4-(4-fluorobenzoyl)toluene chromium tricarbonyl (1t-Cr)

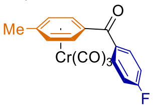

Orange solid. 53.3 mg, 76% yield. The regioisomeric ratio was > 10:1. R_f = 0.35 (PE/EA = 9:1 v/v). Elution with PE/EA = 20:1 to 5:1 v/v. **^{1}H NMR** (500 MHz, Chloroform-*d*) δ 7.81 (dd, J = 8.7, 5.3 Hz, 2H), 7.18 (t, J = 8.6 Hz, 2H), 6.05 (d, J = 6.7 Hz, 2H), 5.18 (d, J = 6.7 Hz, 2H), 2.31 (s, 3H). **^{13}C NMR** (126 MHz, Chloroform-*d*) δ 230.67, 191.97, 165.22 (d, J = 254.2 Hz), 132.83 (d, J = 3.2 Hz), 131.20 (d, J = 9.0 Hz), 115.80 (d, J = 22.0 Hz), 110.94, 96.24, 94.23, 90.45, 20.73. **^{19}F NMR** (471 MHz, Chloroform-*d*) δ -105.76. **HRMS-APCI** (m/z): [M]$^+$ calcd. for $[C_{17}H_{12}CrFO_4]^+$, 351.0119, found, 351.0117.

4-(4-fluorobenzoyl)styrene chromium tricarbonyl (1ac-Cr)

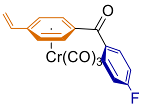

Red solid. 47.7 mg, 66% yield. The regioisomeric ratio was > 10:1. R_f = 0.50 (PE/EA = 9:1 v/v). Elution with PE/EA = 20:1 to 5:1 v/v. **^{1}H NMR** (500 MHz, Chloroform-*d*) δ 7.83 (dd, J = 8.6, 5.5 Hz, 2H), 7.19 (t, J = 8.6 Hz, 2H), 6.38 (dd, J = 17.5, 10.8 Hz, 1H), 6.08 (d, J = 6.7 Hz, 2H), 5.79 (d, J = 17.4 Hz, 1H), 5.49 (d, J = 10.8 Hz, 1H), 5.44 (d, J = 6.7 Hz, 2H). **^{13}C NMR** (126 MHz, Chloroform-*d*) δ 230.62, 192.14, 165.39 (d, J = 254.5 Hz), 133.19, 132.87 (d, J = 3.2 Hz), 131.39 (d, J = 9.1 Hz), 118.53, 115.94 (d, J = 22.0 Hz), 107.29, 95.53, 95.28, 87.91. **^{19}F NMR** (471 MHz, Chloroform-*d*) δ -105.55. **HRMS-APCI** (m/z): [M]$^+$ calcd. for $[C_{18}H_{12}CrFO_4]^+$, 363.0119, found, 363.0125.

2-(2-naphthoyl)(5,6,7,8-tetrahydronaphthalene) chromium tricarbonyl (1w-Cr)

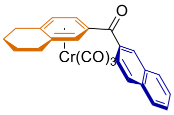

Orange solid. 71.7 mg, 85% yield. The regioisomeric ratio was > 10:1. R_f = 0.45 (PE/EA = 9:1 v/v). Elution with PE/EA = 20:1 to 5:1 v/v. **^{1}H NMR** (500 MHz, Chloroform-*d*) δ 8.41 (s, 1H), 7.97 (dd, J = 16.0, 8.3 Hz, 2H), 7.91 (d, J = 8.0 Hz, 1H), 7.83 (d, J = 8.5 Hz, 1H), 7.60 (dt, J = 19.8, 7.2 Hz, 2H), 6.03 (d, J = 6.8 Hz, 1H), 5.93 (s, 1H), 5.27 (d, J = 6.8 Hz, 1H), 2.77 (s, 2H), 2.63 (tq, J = 16.4, 7.9, 5.1 Hz, 2H), 1.87–1.70 (m, 4H). **^{13}C NMR** (126 MHz, Chloroform-*d*) δ 231.68, 194.09, 135.11, 133.82, 132.18, 129.93, 129.31, 128.62, 128.35, 127.82, 127.05, 124.99, 111.78, 106.91, 97.64, 95.92, 93.98, 91.39, 28.24, 28.10, 22.00, 21.85. **HRMS-APCI** (m/z): [M]$^+$ calcd. for $[C_{24}H_{19}CrO_4]^+$, 423.0683, found, 423.0685.

2.3 The preparation of substrates

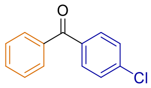 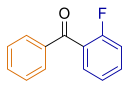 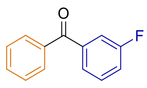 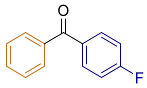 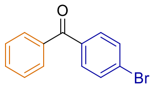 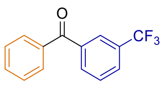

1a **s1** **1c** **1d** **1e** **1y**

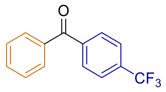 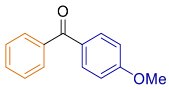 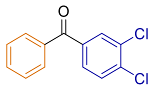 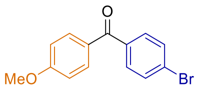 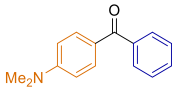

1f **1b** **1aa** **1n** **1j**

The following known substrates were commercially available and used without further purifications: **1a**, **s1**, **1c**, **1d**, **1e**, **1y**, **1f**, **1b**, **1aa**, **1n**, **1j**.

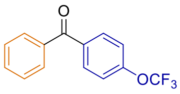 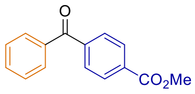 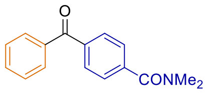 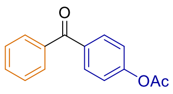 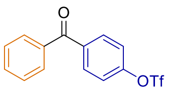

1g **1h** **1i** **s2** **1z**

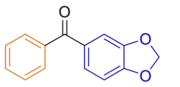 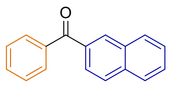 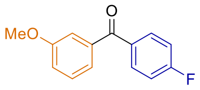 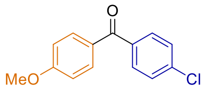

1k **1ab** **1l** **1m**

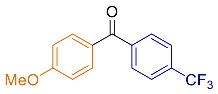 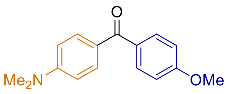 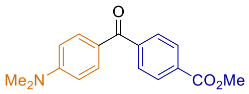 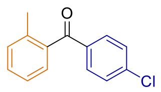

1o **1r** **1s** **1x**

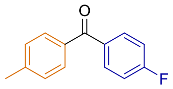 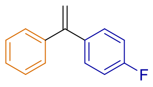 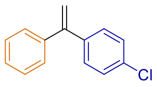 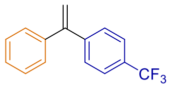

1t **3a** **3b** **3c**

The following known substrates were prepared according to the literature procedures: **1g**[5], **1h**[5], **1i**[5], **s2**[6],**1z**[7], **1k**[5], **1ab**[5], **1l**[8] **1m**[9], **1o**[9], **1r**[9], **1s**[10], **1x**[11], **1t**[11], **3a**[12], **3b**[12], **3c**[12].

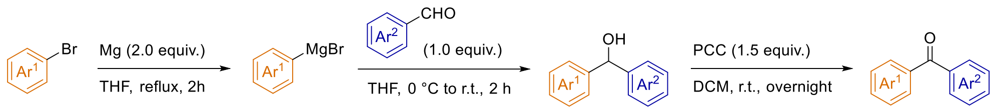

Step i. Under argon atmosphere, magnesium turnings (20.0 mmol, 2.0 equiv) and dry THF (15 mL) were charged in an oven-dried 100 mL round bottom flask equipped with a magnetic stir bar. In a separate oven-dried flask, a 1M solution of the bromoarene (10.0 mmol, 1.0 equiv) in dry THF was prepared, and 20% of this solution was added to the flask containing Mg turnings and heated, followed by addition of 20 µL 1,2-dibromoethane. Once the reaction was initiated with a change or color or gas evolution, the remaining 80% of the bromoarene solution was added dropwise. The solution was refluxed for additional 2 h. Upon completion of the reaction, the Grignard reagent solution was cooled to room temperature and used for next step without titration.

Step ii. A dry 250 mL round bottom flask equipped with a magnetic stir bar, was charged with benzaldehyde (10.0 mmol, 1.0 equiv) and THF (25 mL) under argon. Under vigorous stirring, the prepared Grignard reagent was slowly added under 0 °C. The reaction mixture was stirred under argon for 2.0 h at room temperature and quenched with saturated aqueous NH_4Cl (20 mL). The aqueous layer was extracted with diethyl ether (3 × 20 mL). The combined organic extract was washed with brine, then dried over anhydrous Na_2SO_4, filtered, and concentrated to give crude product diarylmethanol directly used in the next step.

Step iii. A 100 mL round bottom flask equipped with a magnetic stir bar, was charged with diarylmethanol, celite (5.0 g) and DCM (30 mL). Under vigorous stirring, PCC (15.0 mmol) was slowly added at room temperature, and the reaction mixture was stirred at room temperature overnight. Then the reaction mixture was filtered, the filter residue was washed with DCM (30 ml × 3), and the filtrate was concentrated and purified by silica gel column chromatography to afford the desired diaryl ketone.

(4-methoxyphenyl)(4-(trifluoromethoxy)phenyl)methanone (**1p**)

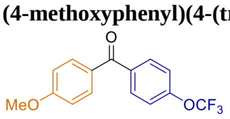

White solid. 86% yield. R_f = 0.50 (PE/EA = 9:1 v/v). Elution with PE/EA = 20:1 to 5:1 v/v. **^{1}H NMR** (500 MHz, Chloroform-*d*) δ 7.81 (d, J = 8.5 Hz, 4H), 7.31 (d, J = 8.0 Hz, 2H), 7.01–6.95 (m, 2H), 3.90 (s, 3H). **^{13}C NMR** (126 MHz, Chloroform-*d*) δ 193.98, 163.46, 151.75, 136.60, 132.49,

131.59, 129.68, 120.34 (q, J = 258.4 Hz), 120.22, 113.71, 55.52. **^{19}F NMR** (471 MHz, Chloroform-*d*) δ -57.61. **HRMS-ESI** (m/z): $[M+H]^+$ calcd. for $[C_{15}H_{12}F_3O_3]^+$, 297.0733, found, 297.0738.

(3,5-bis(trifluoromethyl)phenyl)(4-methoxyphenyl)methanone (s3)

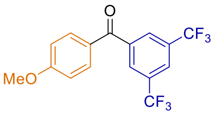

Pale yellow oil. 87% yield. R_f = 0.55 (PE/EA = 9:1 v/v). Elution with PE/EA = 20:1 to 5:1 v/v. **^{1}H NMR** (500 MHz, Chloroform-*d*) δ 8.19 (s, 2H), 8.07 (s, 1H), 7.84–7.77 (m, 2H), 7.05–6.99 (m, 2H), 3.92 (s, 3H). **^{13}C NMR** (126 MHz, Chloroform-*d*) δ 192.24, 164.13, 140.15, 132.57, 131.89 (q, J = 33.7 Hz), 129.52 (q, J = 3.2 Hz), 128.52, 125.12 (h, J = 3.6 Hz), 122.95 (q, J = 273.0 Hz), 114.15, 55.61. **^{19}F NMR** (471 MHz, Chloroform-*d*) δ -62.90. **GCMS-EI** (m/z): $[M]^+$ calcd. for $[C_{16}H_{10}F_6O_2]^+$, 348.1, found, 348.0.

(3-fluoro-5-methoxyphenyl)(4-methoxyphenyl)methanone (1v)

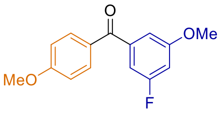

White solid. 92% yield. R_f = 0.40 (PE/EA = 9:1 v/v). Elution with PE/EA = 20:1 to 5:1 v/v. **^{1}H NMR** (500 MHz, Chloroform-*d*) δ 7.83 (d, J = 8.9 Hz, 2H), 7.11–7.06 (m, 1H), 7.01 (ddd, J = 8.5, 2.2, 1.3 Hz, 1H), 6.97 (d, J = 8.9 Hz, 2H), 6.81 (dt, J = 10.2, 2.3 Hz, 1H), 3.90 (s, 3H), 3.85 (s, 3H). **^{13}C NMR** (126 MHz, Chloroform-*d*) δ 193.98 (d, J = 2.5 Hz), 163.51, 162.99 (d, J = 247.1 Hz), 160.76 (d, J = 10.8 Hz), 140.69 (d, J = 8.3 Hz), 132.54, 129.50, 113.68, 110.70 (d, J = 2.7 Hz), 109.06 (d, J = 23.1 Hz), 105.28 (d, J = 25.0 Hz), 55.81, 55.51. **^{19}F NMR** (471 MHz,) δ -110.83. **HRMS-ESI** (m/z): $[M+H]^+$ calcd. for $[C_{15}H_{14}FO_3]^+$, 261.0921, found, 261.0932.

***o*-tolyl(2-(trifluoromethyl)phenyl)methanone (1u)**

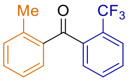

White solid. 93% yield. R_f = 0.55 (PE/EA = 9:1 v/v). Elution with PE/EA = 20:1 to 5:1 v/v. **^{1}H NMR** (500 MHz, Chloroform-*d*) δ 7.80–7.75 (m, 1H), 7.63–7.56 (m, 2H), 7.43 (t, J = 7.5 Hz, 1H), 7.40–7.35 (m, 1H), 7.32 (d, J = 7.6 Hz, 1H), 7.27 (d, J = 7.8 Hz, 1H), 7.18 (t, J = 7.5 Hz, 1H), 2.62 (s, 3H). **^{13}C NMR** (126 MHz, Chloroform-*d*) δ 197.38, 140.35, 139.95 (q, J = 2.0 Hz), 136.05, 132.58, 132.43, 131.98, 131.40 (q, J = 1.2 Hz), 130.00, 128.81, 128.16 (q, J = 32.3 Hz), 126.75 (q, J = 4.9 Hz), 125.34, 123.61 (q, J = 274.0 Hz), 21.41. **^{19}F NMR** (471 MHz, Chloroform-*d*) δ -58.05. **GCMS-EI** (m/z): $[M]^+$ calcd. for $[C_{15}H_{11}F_3O]^+$, 264.1, found, 264.1.

(4-fluorophenyl)(4-vinylphenyl)methanone (1ac)

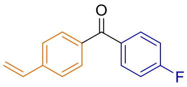

White solid. 86% yield. R_f = 0.55 (PE/EA = 9:1 v/v). Elution with PE/EA = 20:1 to 5:1 v/v. **^{1}H NMR** (500 MHz, Chloroform-*d*) δ 7.87–7.80 (m, 2H), 7.75 (d, J = 8.1 Hz, 2H), 7.51 (d, J = 8.2 Hz, 2H), 7.16 (t, J = 8.6 Hz, 2H), 6.78 (dd, J = 17.6, 10.9 Hz, 1H), 5.90 (d, J = 17.6 Hz, 1H), 5.42 (d, J = 10.9 Hz, 1H). **^{13}C NMR** (126 MHz, Chloroform-*d*) δ 194.72, 165.32 (d, J = 253.8 Hz), 141.61, 136.53, 135.91, 133.92 (d, J = 2.8 Hz), 132.53 (d, J = 9.2 Hz), 130.36, 126.09, 116.69, 115.43 (d, J = 21.9 Hz). **^{19}F NMR** (471 MHz, Chloroform-*d*) δ -106.10. **GCMS-EI** (m/z): $[M]^+$ calcd. for $[C_{15}H_{11}FO]^+$, 226.1, found, 226.1.

naphthalen-2-yl(5,6,7,8-tetrahydronaphthalen-2-yl)methanone (1w)

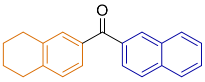

White solid. 84% yield. R_f = 0.55 (PE/EA = 9:1 v/v, elution with PE/EA = 20:1 to 5:1 v/v). **^{1}H NMR** (500 MHz, Chloroform-*d*) δ 8.26 (s, 1H), 7.96–7.88 (m, 4H), 7.64–7.52 (m, 4H), 7.19 (d, J = 7.9 Hz, 1H), 2.88–2.81 (m, 4H), 1.85 (hept, J = 5.2 Hz, 4H). **^{13}C NMR** (126 MHz, Chloroform-*d*) δ 196.78, 142.52, 137.37, 135.31, 135.18, 135.12, 132.26, 131.49, 131.01, 129.34, 129.00, 128.13, 128.10, 127.79, 127.39, 126.68, 125.89, 29.65, 29.37, 22.98, 22.87. **HRMS-ESI** (m/z): $[M+H]^+$ calcd. for $[C_{21}H_{19}O]^+$, 287.1430, found, 287.1438.

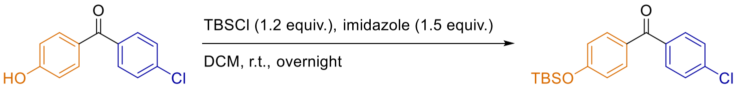

A dry 100 mL round bottom flask equipped with a magnetic stir bar, was charged with (4-chlorophenyl)(4-hydroxyphenyl)methanone (10.0 mmol, 1.0 equiv), TBSCl (12.0 mmol) and DCM (20 mL). Under vigorous stirring, imidazole (15.0 mmol) was slowly added under 0 °C. The reaction mixture was stirred overnight at room temperature and quenched with saturated aqueous NH_4Cl (20 mL). The aqueous layer was extracted with EtOAc (3 × 20 mL). The combined organic extract was washed with brine, then dried over anhydrous Na_2SO_4, filtered, and concentrated to give crude product. The crude product was purified by silica gel column chromatography to afford the desired diaryl ketone **1q**. Colorless oil. 96% yield. R_f = 0.40 (PE/EA = 20:1 v/v). Elution with PE/EA = 30:1 to 10:1 v/v. **^{1}H NMR** (500 MHz, Chloroform-*d*) δ 7.73 (d, J = 8.6 Hz, 2H), 7.71 (d, J =

8.4 Hz, 2H), 7.45 (d, J = 8.4 Hz, 2H), 6.90 (d, J = 8.6 Hz, 2H), 1.00 (s, 9H), 0.25 (s, 6H). **^{13}C NMR** (126 MHz, Chloroform-*d*) δ 194.31, 160.12, 138.24, 136.47, 132.32, 131.14, 130.29, 128.46, 119.79, 25.54, 18.20, -4.39. **HRMS-ESI** (m/z): [M+H]$^+$ calcd. for [$C_{19}H_{24}ClO_2Si$]$^+$, 347.1229, found, 347.1232.

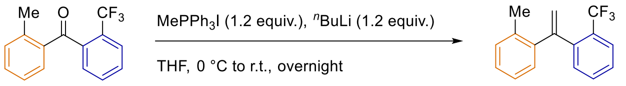

3d

A solution of ^{n}BuLi in hexanes (C = 1.6 M, 12.0 mmol, 1.2 equiv.) was added dropwise, at 0 °C, to a solution of $MePPh_3I$ (12.0 mmol, 1.2 equiv.) in dry THF (30 mL). The reaction mixture was stirred at 0 °C for 30 minutes. A solution of the *o*-tolyl(2-(trifluoromethyl)phenyl)methanone (10.0 mmol, 1.0 equiv.) in dry THF (10 mL) was added dropwise at 0 °C, and the reaction mixture was stirred at room temperature overnight. It was then diluted with brine and extracted with EtOAc (3 × 40 mL). The combined organic extracts were washed with brine and dried with Na_2SO_4. The solvent was removed under reduced pressure and the residue was purified by flash chromatography on silica to yield the desired product. Yellow solid. 65% yield. R_f = 0.80 (PE). Elution with PE. **^{1}H NMR** (600 MHz, Chloroform-*d*) δ 7.68 (d, J = 7.9 Hz, 1H), 7.46 (t, J = 7.5 Hz, 1H), 7.38 (t, J = 7.6 Hz, 1H), 7.27 (d, J = 7.7 Hz, 1H), 7.21–7.15 (m, 2H), 7.16–7.07 (m, 2H), 5.54 (d, J = 18.4 Hz, 2H), 2.23 (s, 3H). **^{13}C NMR** (151 MHz, Chloroform-*d*) δ 146.69, 141.87, 141.04, 135.58, 131.75, 131.35, 130.82, 130.01, 127.91 (q, J = 30.0, 29.6 Hz), 127.48, 127.29, 126.57 (q, J = 5.6 Hz), 125.52, 124.18 (q, J = 273.6 Hz), 120.79, 20.83. **^{19}F NMR** (565 MHz, Chloroform-*d*) δ -57.13. **GCMS-EI** (m/z): [M]$^+$ calcd. for [$C_{16}H_{13}F$]$^+$, 262.1, found, 262.1.

3 Asymmetric Hydrogenation of 1,1-Diarylethenes and Benzophenones

3.1 Synthesis of ruthenium-catalysts

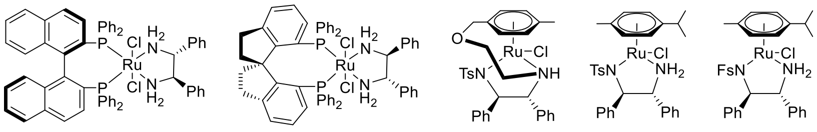

Ru-3 **Ru-4** **Ru-5** **Ru-6** **Ru-7**

The following known ruthenium-catalysts were commercially available and used without further purifications: **Ru-3**, **Ru-5**, **Ru-6**, **Ru-7**. **Ru-4**[13] was prepared according to the literature.

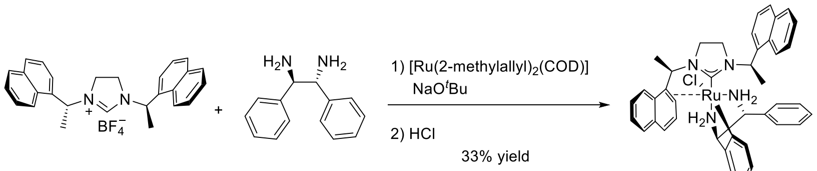

(*R*,*R*)-SINpEt·HBF$_4$ (*R*,*R*)-DPEN **Ru-1**

Ru-1 was prepared according to the literature.[14] A typical procedure is described as follow. In a glove box, to an over-dried tube was added [Ru(2-methylallyl)$_2$(COD)] (524 mg, 1.5 mmol), (*R*,*R*)-SINpEt·HBF$_4$ (717 mg, 1.5 mmol), (*R*,*R*)-1,2-diphenylethylenediamine (318 mg, 1.5 mmol), and NaO^{t}Bu (173 mg, 1.8 mmol). The mixture was suspended in *n*-hexane (60 mL) and stirred at 40 °C for 4 days. HCl (4M in dioxane) (2.7 mmol) was then added to the reaction mixture at 0 °C. After stirring for 30 min at 0 °C, the mixture was concentrated and purified by column chromatography on silica gel (elution with PE/EA = 20:1 to 2:1 v/v) to yield the complexes [Ru-**1**] (360 mg, 0.49 mmol) in 33% yield. **Ru-1'** was prepared by the same method from (*S*,*S*)-SINpEt·HBF$_4$ and (*S*,*S*)-1,2-diphenylethylenediamine. **^{1}H NMR** (500 MHz, Chloroform-*d*) δ 8.38 (d, J = 8.2 Hz, 1H), 7.79 (d, J = 7.3 Hz, 1H), 7.71 (d, J = 8.0 Hz, 1H), 7.63 (d, J = 8.1 Hz, 1H), 7.52 (d, J = 7.1 Hz, 1H), 7.44 (t, J = 7.7 Hz, 1H), 7.40–7.35 (m, 1H), 7.33 (t, J = 7.3 Hz, 1H), 7.29–7.27 (m, 1H), 7.15–7.14 (m, 2H), 7.13–7.09 (m, 1H), 7.09–7.05 (m, 3H), 7.04–7.00 (m, 3H), 6.56 (d, J = 18.7, 1H), 6.56 (d, J = 8.0 Hz, 1H), 6.15 (dd, J = 9.0, 5.7 Hz, 1H), 5.46 (q, J = 6.4 Hz, 1H), 4.06–3.92 (m, 2H), 3.79 (td, J = 9.4, 2.9 Hz, 1H), 3.75 (d, J = 5.7 Hz, 1H), 3.72–3.65 (m, 2H), 3.58 (d, J = 7.9 Hz, 1H), 2.89–2.74 (m, 2H), 1.63 (d, J = 7.0 Hz, 3H), 1.26 (t, J = 7.1 Hz, 2H), 1.24–1.17 (m, 4H), 1.07 (d, J = 9.4 Hz, 1H), 0.13 (d, J = 9.3 Hz, 1H).

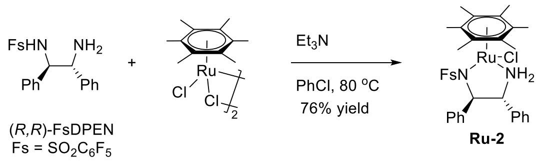

(*R*,*R*)-FsDPEN
Fs = $SO_2C_6F_5$
Ru-2

To a premixed solution of [$RuCl_2(\eta^6\text{-}C_6Me_6)$]$_2$ (334.0 mg, 0.5 mmol) and (*R*,*R*)-FsDPEN (442.4 mg, 1.0 mmol) in chlorobenzene (15 mL), Et_3N (404.8 mg, 4.00 mmol) was added and refluxed for 2 h. After evaporation of the resulting reddish orange solution, the residual mixture was dissolved in CH_2Cl_2 (15 mL), washed with water (2 mL× 3) and dried over Na_2SO_4. The solvent was removed in vacuum and the residue was purified by column chromatography on silica gel (elution with PE/EA = 1:1 v/v) to afford an orange powder of **Ru-2** (562.4 mg, 0.76 mmol) in 76% yield. **^{1}H NMR** (500 MHz, Chloroform-*d*) δ 7.20–7.12 (m, 3H), 6.90–6.83 (m, 7H), 4.15 (d, J = 11.0 Hz, 1H), 3.85 (dd, J = 13.6, 9.9 Hz, 1H), 3.75–3.63 (m,

2H). ^{13}C NMR (126 MHz, $CDCl_3$) δ 139.11, 138.29, 128.78, 128.72, 127.88, 127.27, 126.96, 126.85, 91.66, 71.59, 67.99, 16.07. ^{19}F NMR (471 MHz, CDCl3) δ -134.33 (d, J = 22.6 Hz), -153.58 (t, J = 20.8 Hz), -163.20 (t, J = 20.8 Hz). **HRMS-ESI** (m/z): $[M\text{-}Cl]^+$ calcd. for $[C_{32}H_{32}F_5N_2O_2RuS]^+$, 705.1143, found, 705.1165.

3.2 Asymmetric hydrogenation of 1a-Cr with different ruthenium-catalysts

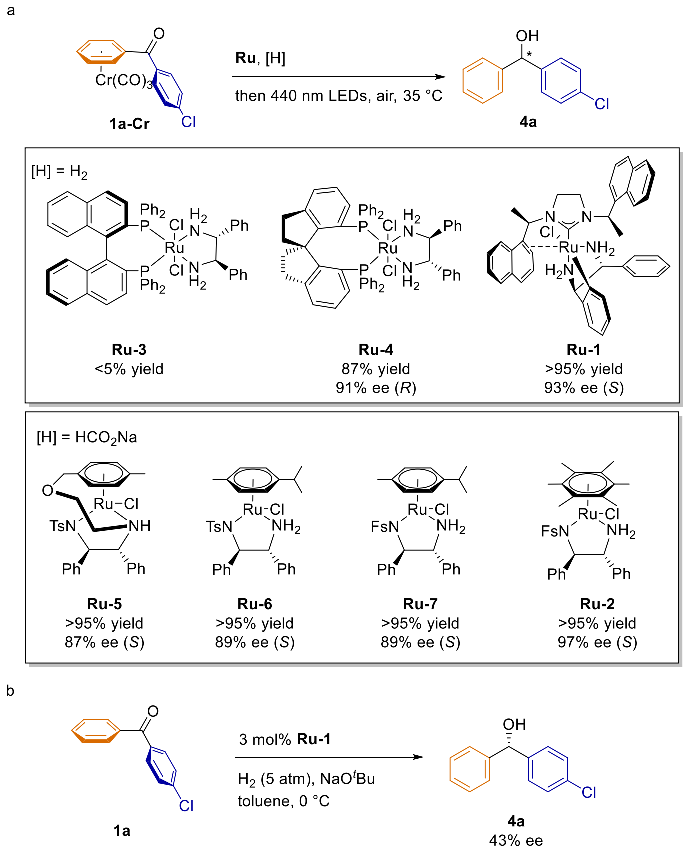

Supplementary Fig. 4 a) Asymmetric hydrogenation of **1a-Cr** with different ruthenium-catalysts. b) Asymmetric hydrogenation of **1a** with **Ru-1**.

General procedure:

[H] = H₂: To a 5 ml glass tube **1a-Cr** or **1a** (0.1 mmol), **Ru** (3 mol%), NaO^tBu (15 mol%), and toluene (1.0 mL) were added in a glovebox. The glass tube was taken out of the glovebox and placed in a pre-cooled (0 °C) stainless steel autoclave, then the autoclave and pressurized and depressurized with hydrogen gas five times before the indicated pressure (5 atm) was set. The reaction mixture was stirred at 0 °C for 36 h. After the autoclave was carefully depressurized, the tube was irradiated under 440 nm LEDs (20 W) for 2 hours at room temperature in air atmosphere. Yields were determined by ^{1}H NMR using 1,1,2,2-tetrachloroethane as the internal standard, and enantiomeric excess was determined by SFC analysis using chiral column.

[H] = HCO_2Na: To a 4ml glass vial, **1a-Cr** (0.1 mmol), **Ru** (7 mol%), HCO_2Na (10.0 equiv.), and DMF/H_2O (10:1, 0.5 ml/0.05 ml) were added in a glovebox. The reaction mixture was stirred at 35 °C for 36 h. Then the vial was irradiated under 440 nm LEDs (20 W) for 2 hours at room temperature in air atmosphere. Yields were determined by ^{1}H NMR using 1,1,2,2-tetrachloroethane as the internal standard, and enantiomeric excess was determined by SFC analysis using chiral column.

3.3 General procedure for asymmetric hydrogenation of benzophenones and 1,1-diarylethenes

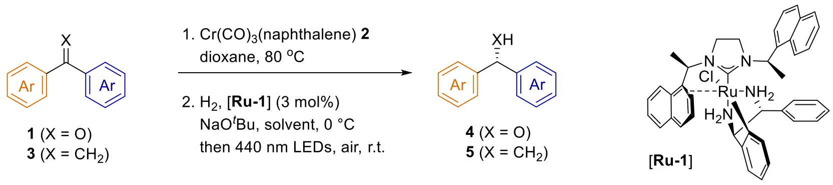

General procedure A:

(1) In a glovebox, a solution of $Cr(CO)_3$(naphthalene) **2** (0.3 mmol, 1.5 equiv.), **1** or **3** (0.2 mmol, 1.0 equiv.) in 1,4-dioxane (2 mL) in a 4 ml glass vial was stirred at room temperature for 2 min, then the sealed reaction vial was taken out of the glovebox and the reaction mixture was stirred at

80 °C in the dark for 24 h. After cooling to room temperature, the solution was evaporated under reduced pressure. The residue was purified by column chromatography eluting with EtOAc/Petroleum ether (impurities that cannot be separated by column chromatography can be removed by recrystallization from diethyl ether and pentane).

(2) To a 5 ml glass tube, **Ru-1** (3 mol%), NaO^{t}Bu (15 mol%), **1-Cr** or **3-Cr** (1.0 equiv.), and toluene (hexane for 1,1-diarylethenes) (0.10 M) were added in a glove box. The glass tube was taken out of the glovebox and placed in a pre-cooled (0 °C) stainless steel autoclave, then the autoclave and pressurized and depressurized with hydrogen gas five times before the indicated pressure (5 atm) (50 atm for 1,1-diarylethenes) was set. The reaction mixture was stirred at 0 °C for 36 h. After the autoclave was carefully depressurized, the tube was irradiated under 440 nm LEDs (20 W) for 2 hours at room temperature in air atmosphere. Then the product was purified by flash column chromatography on silica gel. The enantiomeric excess of the product was determined by SFC or HPLC analysis using chiral column (when ours chiral column cannot separate the product, the enantiomeric excess was measured before the chromium is removed).

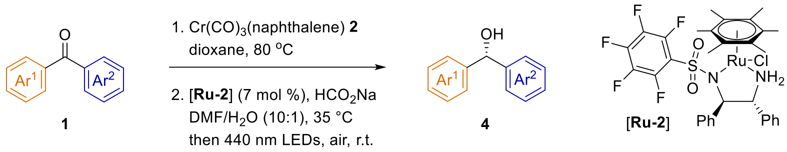

General procedure B:

(1) In a glovebox, a solution of $Cr(CO)_3$(naphthalene) **2** (0.3 mmol, 1.5 equiv.), **1** (0.2 mmol, 1.0 equiv.) in 1,4-dioxane (2 mL) in a 4 ml glass vial was stirred at room temperature for 2 min, then the sealed reaction vial was taken out of the glovebox and and the reaction mixture was stirred at 80 °C in the dark for 24 h. After cooling to room temperature, the solution was evaporated under reduced pressure. The red residue was purified by column chromatography eluting with EtOAc/Petroleum ether (impurities that cannot be separated by column chromatography can be removed by recrystallization from diethyl ether and pentane).

(2) To a 4ml glass vial, **Ru-2** (7 mol%), HCO_2Na (10 equiv.), **1-Cr** (1.0 equiv.), and DMF/H_2O (10:1, 0.18 M) were added in a glovebox. The reaction mixture was stirred at 35 °C for 36 h. Then the vial was irradiated under 440 nm LEDs (20 W) for 2 hours at room temperature in air atmosphere. Then the product was purified by flash column chromatography on silica gel. The enantiomeric excess of the product was determined by SFC or HPLC analysis using chiral column (when ours chiral column cannot separate the product, the enantiomeric excess was measured before the chromium is removed).

(*S*)-(4-chlorophenyl)(phenyl)methanol (4a)

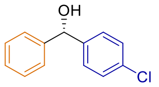

Known compound.[15] **Procedure A**, white solid, 34.0 mg, 78% yield, 93% ee. **Procedure B**, white solid, 32.3 mg, 74% yield, 96% ee. R_f = 0.50 (PE/EA = 6:1 v/v). Elution with PE/EA = 10:1 to 5:1 v/v. **^{1}H NMR** (500 MHz, Chloroform-*d*) δ 7.50–7.09 (m, 9H), 5.66 (s, 1H), 2.75 (s, 1H).

Racemic

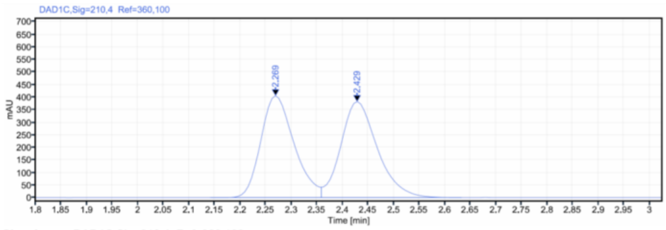

Signal: DAD1C,Sig=210,4 Ref=360,100

RT [min]	Type	Width [min]	Area	Height	Area%	Name
2.269	BV	0.22	1835.34	406.19	49.14	
2.429	VV	0.27	1899.57	383.18	50.86	
		Sum	3734.91			

Chiral

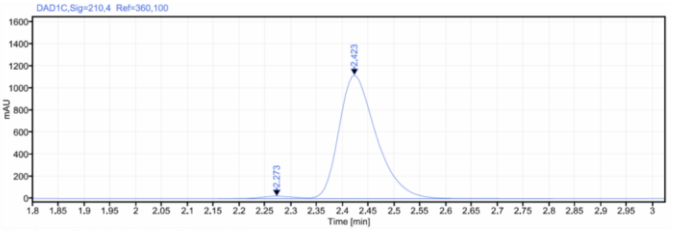

Signal: DAD1C,Sig=210,4 Ref=360,100

RT [min]	Type	Width [min]	Area	Height	Area%	Name
2.273	BV	0.17	90.76	21.54	1.59	
2.423	VBA	0.47	5607.61	1120.02	98.41	
		Sum	5698.37			

Supplementary Fig. 5 SFC chromatograms of 4a. AD-3 column, MeOH/CO_2 = 10:90, 1.0 mL/min, 210 nm, t_{minor} = 2.3 min, t_{major} = 2.4 min. $[\alpha]_D^{20}$ +19.8 (c 1.02 in $CHCl_3$) 93% ee (*S*) (lit.[16] $[\alpha]_D^{20}$ +8.0 (c 1.51 in $CHCl_3$) 48% ee (*S*)).

(*S*)-(4-methoxyphenyl)(phenyl)methanol (4b)

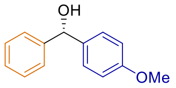

Known compound.[17] **Procedure A**, step (1) with 0.22 mmol **2**, 24.8 mg, white solid, 58% yield, 90% ee. R_f = 0.55 (PE/EA = 4:1 v/v). Elution with PE/EA = 10:1 to 5:1 v/v. **^{1}H NMR** (600 MHz, Chloroform-*d*) δ 7.35 (d, J = 7.4 Hz, 2H), 7.31 (t, J = 7.6 Hz, 2H), 7.28–7.22 (m, 3H), 6.84 (d, J = 8.7 Hz, 2H), 5.76 (s, 1H), 3.76 (s, 3H), 2.32 (s, 1H).

Racemic

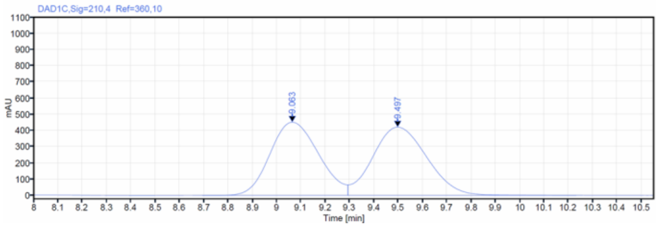

Signal: DAD1C,Sig=210,4 Ref=360,10

RT [min]	Type	Width [min]	Area	Height	Area%	Name
9.063	BV	0.60	6272.49	451.81	49.04	
9.497	VV	0.94	6518.44	421.21	50.96	
		Sum	12790.93			

Chiral

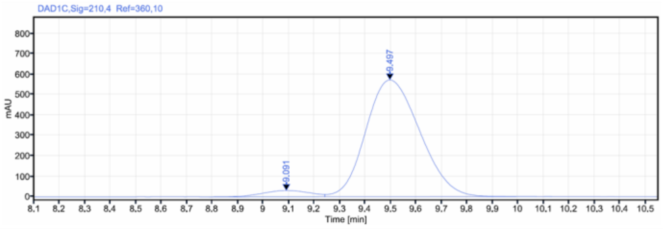

Signal: DAD1C,Sig=210,4 Ref=360,10

RT [min]	Type	Width [min]	Area	Height	Area%	Name
9.091	BV	0.45	403.86	30.95	4.56	
9.497	VV	0.75	8445.58	572.96	95.44	
		Sum	8849.44			

Supplementary Fig. 6 SFC chromatograms of 4b. OJ-3 column, MeOH/CO_2 = 10:90, 0.6 mL/min, 210 nm, t_{minor}= 9.1 min, t_{major} = 9.5 min.

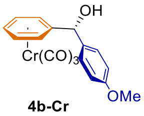

4b-Cr

4b-Cr, 91% ee. (**1b-Cr** with **Ru-1**)

Racemic

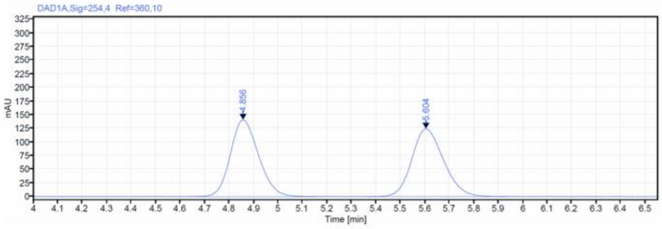

Signal: DAD1A,Sig=254,4 Ref=360,10

RT [min]	Type	Width [min]	Area	Height	Area%	Name
4.856	BV	0.50	1133.54	140.89	50.05	
5.604	BV	0.57	1131.23	124.55	49.95	
		Sum	2264.77			

Chiral

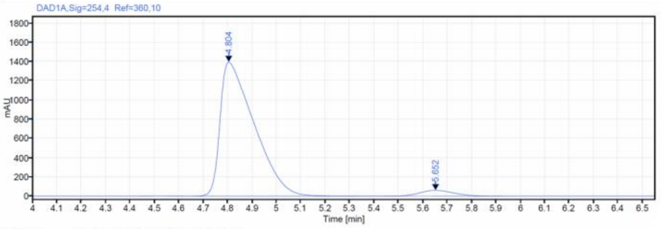

Signal: DAD1A,Sig=254,4 Ref=360,10

RT [min]	Type	Width [min]	Area	Height	Area%	Name
4.804	VV	0.71	13504.38	1396.81	95.82	
5.652	VV	0.52	589.75	60.16	4.18	
		Sum	14094.13			

Supplementary Fig. 7 SFC chromatograms of 4b-Cr. OD-3 column, MeOH/CO_2 = 10:90, 1.5 mL/min, 254 nm, t_{minor}= 5.6 min, t_{major} = 4.8 min.

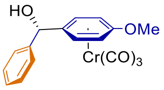

4b-Cr'

4b-Cr', 93% ee. (**1b-Cr'** with **Ru-1'**)

Racemic

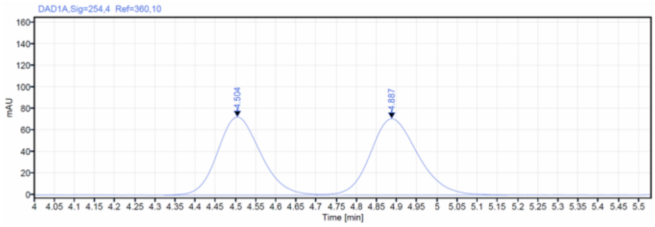

Signal: DAD1A,Sig=254,4 Ref=360,10

RT [min]	Type	Width [min]	Area	Height	Area%	Name
4.504	VV	0.38	539.26	72.54	48.13	
4.887	VV	0.47	581.10	70.96	51.87	
		Sum	1120.36			

Chiral

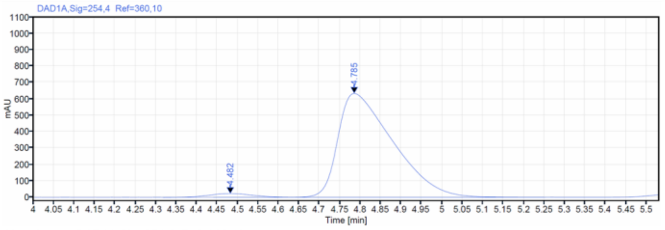

Signal: DAD1A,Sig=254,4 Ref=360,10

RT [min]	Type	Width [min]	Area	Height	Area%	Name
4.482	BV	0.31	177.56	23.25	2.91	
4.785	VV	0.62	5930.63	634.52	97.09	
		Sum	6108.19			

Supplementary Fig. 8 SFC chromatograms of 4b-Cr'. OD-3 column, MeOH/CO_2 = 10:90, 1.5 mL/min, 254 nm, t_{minor}= 4.5 min, t_{major} = 4.8 min.

(*S*)-(3-fluorophenyl)(phenyl)methanol (**4c**)

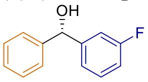

Known compound.[18] **Procedure A**, white solid, 32.3 mg, 80% yield, 82% ee. **Procedure B**, white solid, 30.6 mg, 76% yield, 95% ee. R_f = 0.45 (PE/EA = 6:1 v/v). Elution with PE/EA = 10:1 to 5:1 v/v. **^{1}H NMR** (500 MHz, Chloroform-*d*) δ 7.36–7.30 (m, 4H), 7.30–7.24 (m, 2H), 7.15–7.07 (m, 2H), 6.98–6.90 (m, 1H), 5.77 (s, 1H), 2.40 (s, 1H).

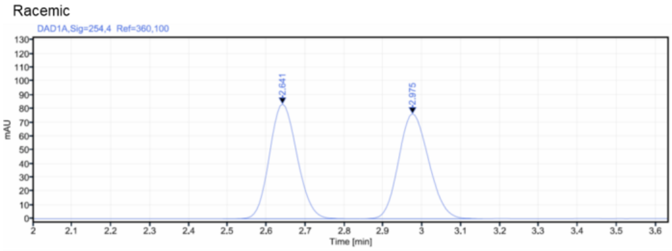

Signal: DAD1A,Sig=254,4 Ref=360,100

RT [min]	Type	Width [min]	Area	Height	Area%	Name
2.641	BB	0.35	410.02	83.49	50.05	
2.975	BB	0.31	409.26	76.44	49.95	
		Sum	819.28			

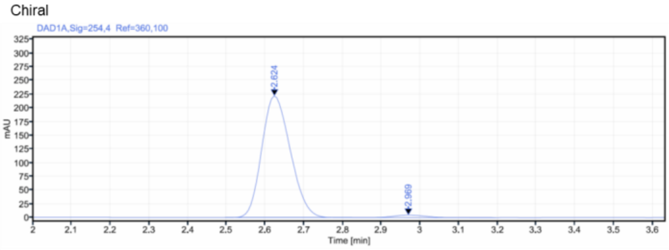

Signal: DAD1A,Sig=254,4 Ref=360,100

RT [min]	Type	Width [min]	Area	Height	Area%	Name
2.624	BB	0.33	1135.82	222.11	97.72	
2.969	BB	0.37	26.54	4.83	2.28	
		Sum	1162.36			

Supplementary Fig. 9 SFC chromatograms of 4c-Cr. OD-3 column, MeOH/CO_2 = 7:93, 1.0 mL/min, 254 nm, t_{minor} = 3.0 min, t_{major} = 2.6 min.

(*S*)-(4-fluorophenyl)(phenyl)methanol (4d)

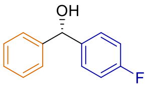

Known compound.[15] **Procedure A**, white solid, 31.0 mg, 77% yield, 96% ee. **Procedure B**, white solid, 31.1 mg, 77% yield, 96% ee. R_f = 0.45 (PE/EA = 6:1 v/v). Elution with PE/EA = 10:1 to 5:1 v/v. **^{1}H NMR** (500 MHz, Chloroform-*d*) *δ* 7.39–7.31 (m, 6H), 7.31–7.24 (m, 1H), 7.01 (t, J = 8.7 Hz, 2H), 5.82 (s, 1H), 2.23 (s, 1H).

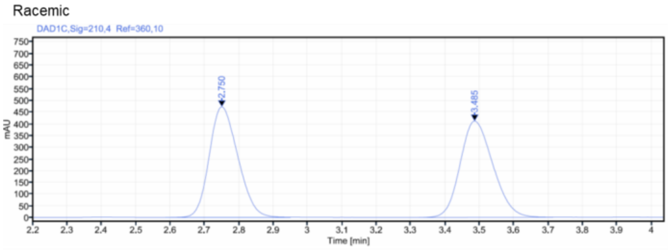

Signal: DAD1C,Sig=210,4 Ref=360,10

RT [min]	Type	Width [min]	Area	Height	Area%	Name
2.750	VV	0.36	2734.62	472.39	50.10	
3.485	BV	0.39	2723.74	411.03	49.90	
		Sum	5458.37			

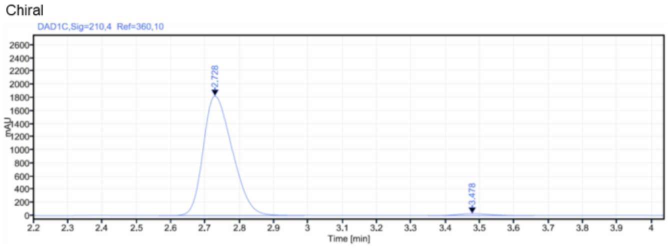

Signal: DAD1C,Sig=210,4 Ref=360,10

RT [min]	Type	Width [min]	Area	Height	Area%	Name
2.728	BV	0.42	10707.25	1820.90	98.23	
3.478	VB	0.31	193.35	29.53	1.77	
		Sum	10900.60			

Supplementary Fig. 10 SFC chromatograms of 4d-Cr. AD-3 column, MeOH/CO_2 = 10:90, 1.0 mL/min, 210 nm, t_{minor} = 3.5 min, t_{major} = 2.7 min.

(*S*)-(4-bromophenyl)(phenyl)methanol (4e)

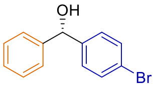

Known compound[15]. **Procedure A**, white solid, 38.3 mg, 73% yield, 92% ee. **Procedure B**, white solid, 36.7 mg, 70% yield, 96% ee. R_f = 0.45 (PE/EA = 6:1 v/v). Elution with PE/EA = 10:1 to 5:1 v/v. **^{1}H NMR** (500 MHz, Chloroform-*d*) *δ* 7.45 (d, J = 8.5 Hz, 2H), 7.34 (d, J = 4.4 Hz, 4H), 7.30–7.24 (m, 3H), 5.79 (s, 1H), 2.24 (s, 1H).

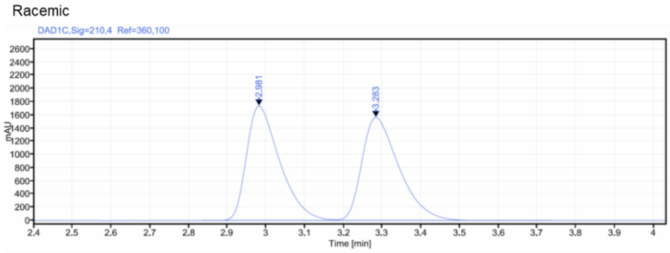

Signal: DAD1C,Sig=210,4 Ref=360,100

RT [min]	Type	Width [min]	Area	Height	Area%	Name
2.981	VV	0.34	10335.25	1738.84	49.82	
3.283	VB	0.56	10409.06	1563.98	50.18	
		Sum	20744.31			

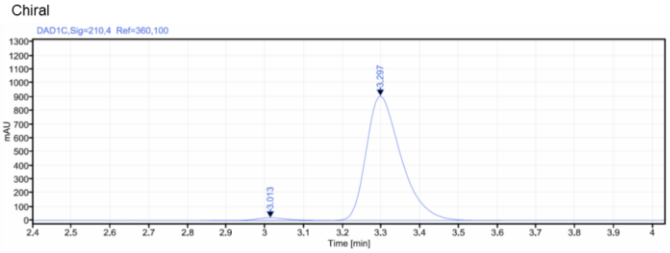

Signal: DAD1C,Sig=210,4 Ref=360,100

RT [min]	Type	Width [min]	Area	Height	Area%	Name
3.013	BV	0.33	107.49	19.86	1.87	
3.297	VB	0.45	5641.80	907.17	98.13	
		Sum	5749.29			

Supplementary Fig. 11 SFC chromatograms of 4e. OJ-3 column, MeOH/CO_2 = 10:90, 1.0 mL/min, 210 nm, t_{minor} = 3.0 min, t_{major} = 3.3 min. $[\alpha]_D^{20}$ +20.0 92% ee (c 1.02 in $CHCl_3$) (*S*) (lit.[19] $[\alpha]_D^{25}$ +19.0 (c 1.00 in $CHCl_3$) 91% ee (*S*)).

(*S*)-phenyl(4-(trifluoromethyl)phenyl)methanol (4f)

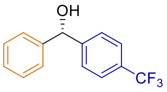

Known compound.[15] **Procedure A**, white solid, 39.3 mg, 78% yield, 90% ee. **Procedure B**, white solid, 37.7 mg, 75% yield, 99% ee. R_f = 0.50

(PE/EA = 4:1 v/v). Elution with PE/EA = 10:1 to 5:1 v/v. ^{1}**H NMR** (500 MHz, Chloroform-*d*) *δ* 7.59 (d, J = 8.2 Hz, 2H), 7.52 (d, J = 8.1 Hz, 2H), 7.36 (d, J = 4.3 Hz, 4H), 7.34–7.26 (m, 1H), 5.89 (d, J = 2.7 Hz, 1H), 2.28 (d, J = 3.1 Hz, 1H).

Racemic | Chiral

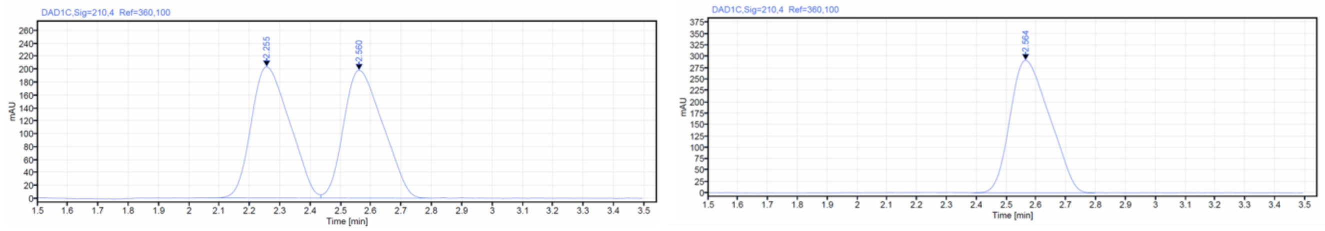

Racemic — Signal: DAD1C,Sig=210,4 Ref=360,100

RT [min]	Type	Width [min]	Area	Height	Area%	Name
2.255	BV	0.37	1777.26	202.93	50.02	
2.560	VB	0.37	1776.03	197.86	49.98	
		Sum	3553.29			

Chiral — Signal: DAD1C,Sig=210,4 Ref=360,100

RT [min]	Type	Width [min]	Area	Height	Area%	Name
2.564	BV	0.42	2682.12	292.17	100.00	
		Sum	2682.12			

Supplementary Fig. 12 SFC chromatograms of 4f. OJ-3 column, MeOH/CO_2 = 7:93, 1.0 mL/min, 210 nm, t_{minor} = 2.3 min, t_{major} = 2.6 min.

(*S*)-phenyl(4-(trifluoromethoxy)phenyl)methanol (4g)

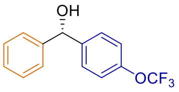

Known compound.[20] **Procedure A**, white solid, 42.1 mg, 78% yield, 91% ee. **Procedure B**, white solid, 41.8 mg, 78% yield, 95% ee. R_f = 0.45 (PE/EA = 6:1 v/v). Elution with PE/EA = 10:1 to 5:1 v/v. ^{1}**H NMR** (500 MHz, Chloroform-*d*) *δ* 7.40 (d, *J* = 8.4 Hz, 2H), 7.35 (d, *J* = 4.4 Hz, 4H), 7.33–7.25 (m, 1H), 7.17 (d, *J* = 7.6 Hz, 2H), 5.83 (d, *J* = 3.4 Hz, 1H), 2.32 (d, *J* = 3.5 Hz, 1H).

Racemic | Chiral

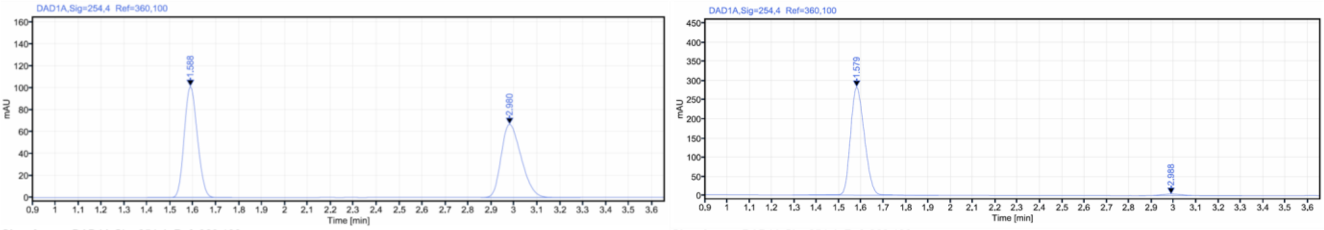

Racemic — Signal: DAD1A,Sig=254,4 Ref=360,100

RT [min]	Type	Width [min]	Area	Height	Area%	Name
1.588	BB	0.37	402.58	101.75	49.94	
2.980	BB	0.43	403.50	66.91	50.06	
		Sum	806.08			

Chiral — Signal: DAD1A,Sig=254,4 Ref=360,100

RT [min]	Type	Width [min]	Area	Height	Area%	Name
1.579	BB	0.65	1224.66	283.81	97.93	
2.988	BB	0.46	25.92	4.31	2.07	
		Sum	1250.58			

Supplementary Fig. 13 SFC chromatograms of 4g-Cr. OJ-3 column, MeOH/CO_2 = 7:93, 1.0 mL/min, 254 nm, t_{minor} = 3.0 min, t_{major} = 1.6 min.

(*S*)-4-(hydroxy(phenyl)methyl)benzoate (4h)

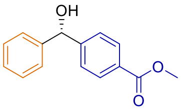

Known compound.[21] **Procedure A**, white solid, 29.1 mg, 60% yield, 87% ee. **Procedure B**, white solid, 28.2 mg, 58% yield, 95% ee. R_f = 0.50 (PE/EA = 4:1 v/v). Elution with PE/EA = 10:1 to 4:1 v/v. ^{1}**H NMR** (500 MHz, Chloroform-*d*) *δ* 7.97 (d, *J* = 8.4 Hz, 2H), 7.44 (d, *J* = 8.3 Hz, 2H), 7.37–7.28 (m, 4H), 7.28–7.23 (m, 1H), 5.84 (s, 1H), 3.87 (s, 3H), 2.68 (s, 1H).

Racemic | Chiral

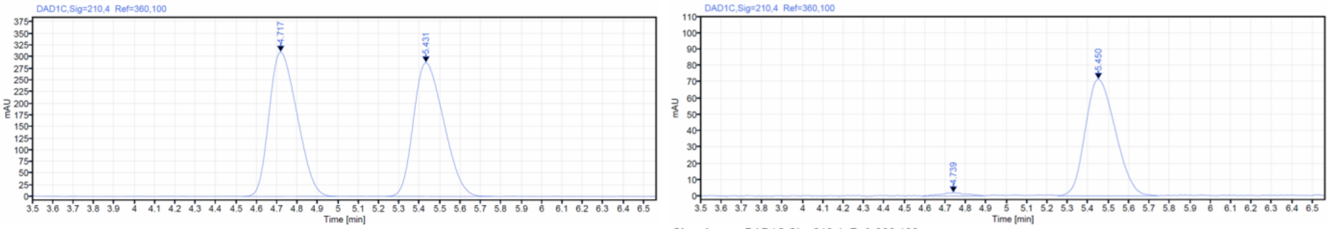

Racemic — Signal: DAD1C,Sig=210,4 Ref=360,100

RT [min]	Type	Width [min]	Area	Height	Area%	Name
4.717	BV	0.46	2959.23	310.10	49.96	
5.431	VV	0.52	2964.24	287.27	50.04	
		Sum	5923.47			

Chiral — Signal: DAD1C,Sig=210,4 Ref=360,100

RT [min]	Type	Width [min]	Area	Height	Area%	Name
4.739	BB	0.30	18.61	2.00	2.47	
5.450	VB	0.49	734.30	71.46	97.53	
		Sum	752.91			

Supplementary Fig. 14 SFC chromatograms of 4h. OJ-3 column, MeOH/CO_2 = 10:90, 1.0 mL/min, 210 nm, t_{minor} = 4.7 min, t_{major} = 5.5 min. $[\alpha]_D^{20}$ +17.6 (c 1.62 in $CHCl_3$) 87% ee (*S*) (lit.[21] $[\alpha]_D^{23}$ +32.0 (c 1.00 in $CHCl_3$) 92% ee (*S*)).

(*S*)-4-(hydroxy(phenyl)methyl)-*N*,*N*-dimethylbenzamide (4i)

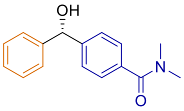

Procedure A, white solid, 19.4 mg, 38% yield, 83% ee. **Procedure B**, white solid, 17.9 mg, 35% yield, 94% ee. R_f = 0.50 (EA). Elution with EA. **^{1}H NMR** (500 MHz, Chloroform-*d*) δ 7.39 (d, *J* = 8.1 Hz, 2H), 7.37–7.30 (m, 6H), 7.26 (t, *J* = 6.9 Hz, 1H), 5.82 (s, 1H), 3.01 (d, *J* = 62.8 Hz, 6H), 2.82 (s, 1H). **^{13}C NMR** (126 MHz, Chloroform-*d*) δ 171.45, 145.36, 143.54, 135.18, 128.50, 127.67, 127.20, 126.59, 126.39, 75.76, 39.57, 35.33. **HRMS** (m/z): $[M+H]^+$ calcd. for $[C_{16}H_{18}NO_2]^+$, 256.1332, found, 256.1336.

Racemic

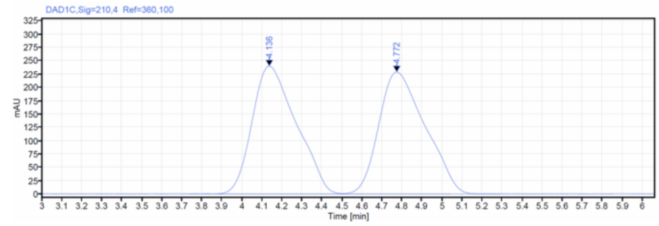

Signal: DAD1C,Sig=210,4 Ref=360,100

RT [min]	Type	Width [min]	Area	Height	Area%	Name
4.136	BV	0.77	3677.79	240.00	49.96	
4.772	VV	0.79	3684.17	228.98	50.04	
		Sum	7361.96			

Chiral

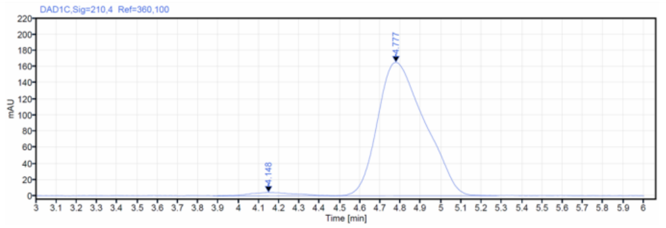

Signal: DAD1C,Sig=210,4 Ref=360,100

RT [min]	Type	Width [min]	Area	Height	Area%	Name
4.148	VV	0.60	73.52	4.41	2.68	
4.777	VV	0.80	2670.27	165.25	97.32	
		Sum	2743.79			

Supplementary Fig. 15 SFC chromatograms of 4i. OJ-3 column, MeOH/CO_2 = 10:90, 1.0 mL/min, 210 nm, t_{minor} = 4.1 min, t_{major} = 4.8 min,.

(*R*)-(4-(dimethylamino)phenyl)(phenyl)methanol (4j)

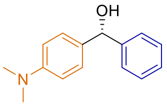

Known compound.[22] **Procedure A**, step (1) with 0.22 mmol **2**, white solid, 31.1 mg, 68% yield, 90% ee. R_f = 0.45 (PE/EA = 4:1 v/v). Elution with PE/EA = 10:1 to 4:1 v/v. **^{1}H NMR** (600 MHz, Chloroform-*d*) δ 7.38 (d, J = 7.3 Hz, 2H), 7.32 (t, J = 7.7 Hz, 2H), 7.25–7.19 (m, 3H), 6.69 (d, J = 8.8 Hz, 2H), 5.77 (s, 1H), 2.92 (s, 6H), 2.10 (s, 1H).

Racemic

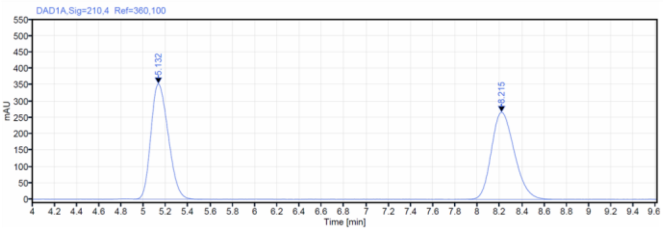

Signal: DAD1A,Sig=210,4 Ref=360,100

RT [min]	Type	Width [min]	Area	Height	Area%	Name
5.132	VV	0.62	3822.72	352.93	49.96	
8.215	VV	0.75	3828.53	266.44	50.04	
		Sum	7651.25			

Chiral

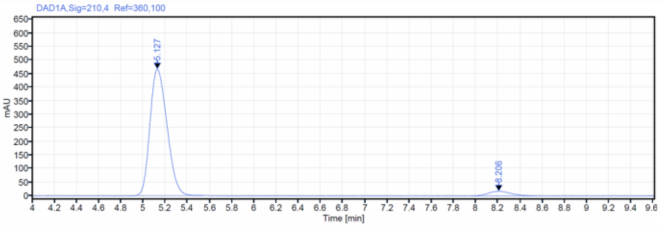

Signal: DAD1A,Sig=210,4 Ref=360,100

RT [min]	Type	Width [min]	Area	Height	Area%	Name
5.127	VV	0.66	4974.89	466.89	95.41	
8.206	BV	0.58	239.46	16.88	4.59	
		Sum	5214.34			

Supplementary Fig. 16 SFC chromatograms of 4j. IH-3 column, MeOH/CO_2 = 10:90, 1.0 mL/min, 210 nm, t_{minor} = 8.2 min, t_{major} = 5.1 min.

(*S*)-benzo[d][1,3]dioxol-5-yl(phenyl)methanol (4k)

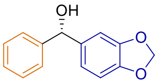

Known compound.[17] **Procedure A**, colorless oil, 36.7 mg, 80% yield, 82% ee. R_f = 0.45 (PE/EA = 4:1 v/v). Elution with PE/EA = 10:1 to 4:1 v/v. **^{1}H NMR** (500 MHz, Chloroform-*d*) δ 7.39–7.30 (m, 4H), 7.29–7.22 (m, 1H), 6.87–6.82 (m, 2H), 6.75 (d, *J* = 8.4 Hz, 1H), 5.91 (q, *J* = 1.4 Hz, 2H), 5.75 (d, *J* = 2.9 Hz, 1H), 2.23 (d, *J* = 3.3 Hz, 1H).

Racemic

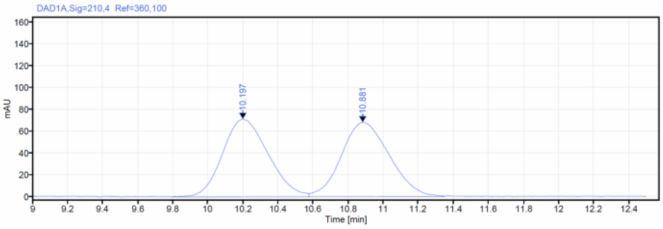

Signal: DAD1A,Sig=210,4 Ref=360,100

RT [min]	Type	Width [min]	Area	Height	Area%	Name
10.197	BV	0.78	1289.46	71.46	49.80	
10.881	VV	0.77	1299.72	68.23	50.20	
		Sum	2589.18			

Chiral

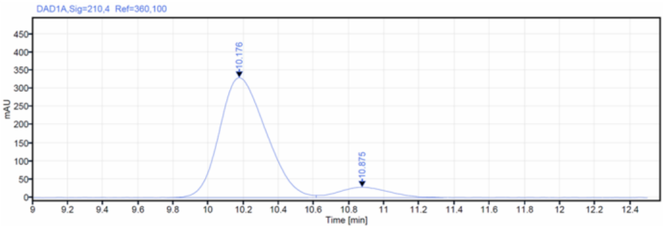

Signal: DAD1A,Sig=210,4 Ref=360,100

RT [min]	Type	Width [min]	Area	Height	Area%	Name
10.176	BV	0.85	6044.72	330.23	91.25	
10.875	VV	0.72	579.57	28.72	8.75	
		Sum	6624.29			

Supplementary Fig. 17 SFC chromatograms of 4k. OD-3 column, MeOH/CO_2 = 10:90, 1.0 mL/min, 210 nm, t_{minor} = 10.9 min, t_{major} = 10.2 min.

(*R*)-(4-fluorophenyl)(3-methoxyphenyl)methanol (4l)

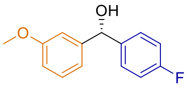

Known compound.[23] **Procedure A**, colorless oil, 35.2 mg, 76% yield, 97% ee. **Procedure B**, colorless oil, 33.8 mg, 73% yield, 91% ee. R_f = 0.45 (PE/EA = 4:1 v/v). Elution with PE/EA = 10:1 to 4:1 v/v. **^{1}H NMR** (600 MHz, Chloroform-*d*) δ 7.33 (dd, J = 8.4, 5.6 Hz, 2H), 7.25 (t, J = 8.1 Hz, 1H), 7.00 (t, J = 8.6 Hz, 2H), 6.94–6.90 (m, 2H), 6.81 (dd, J = 8.2, 2.3 Hz, 1H), 5.78 (s, 1H), 3.78 (s, 3H), 2.31 (s, 1H).

Racemic

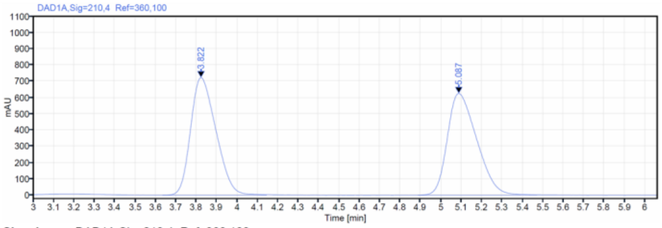

Signal: DAD1A,Sig=210,4 Ref=360,100

RT [min]	Type	Width [min]	Area	Height	Area%	Name
3.822	BV	0.51	6247.25	724.09	49.95	
5.087	VV	0.58	6258.96	626.41	50.05	
		Sum	12506.21			

Chiral

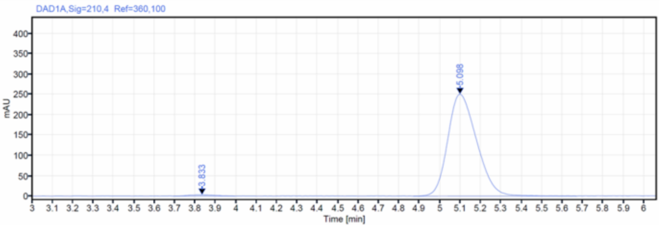

Signal: DAD1A,Sig=210,4 Ref=360,100

RT [min]	Type	Width [min]	Area	Height	Area%	Name
3.833	VV	0.30	30.15	3.32	1.21	
5.098	BB	0.80	2465.77	250.74	98.79	
		Sum	2495.92			

Supplementary Fig. 18 SFC chromatograms of 4l. OD-3 column, MeOH/CO_2 = 10:90, 1.0 mL/min, 210 nm, t_{minor} = 3.8 min, t_{major} = 5.1 min.

(*S*)-(4-chlorophenyl)(4-methoxyphenyl)methanol (4m)

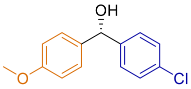

Known compound.[16] **Procedure A**, white solid, 33.0 mg, 66% yield, 94% ee. **Procedure B**, white solid, 32.2 mg, 65% yield, 93% ee. R_f = 0.45 (PE/EA = 4:1 v/v). Elution with PE/EA = 10:1 to 4:1 v/v. **^{1}H NMR** (500 MHz, Chloroform-*d*) δ 7.33–7.28 (m, 4H), 7.25 (d, J = 8.0 Hz, 2H), 6.87 (d, J = 8.8 Hz, 2H), 5.78 (s, 1H), 3.79 (s, 3H), 2.14 (s, 1H).

Racemic

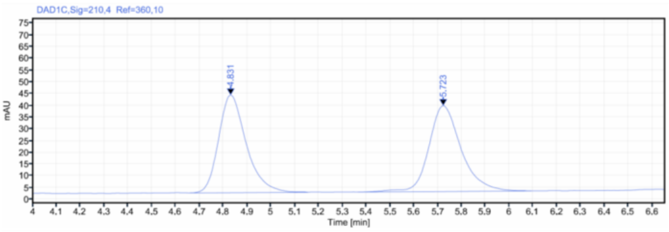

Signal: DAD1C,Sig=210,4 Ref=360,10

RT [min]	Type	Width [min]	Area	Height	Area%	Name
4.831	BV	0.49	335.87	41.63	49.42	
5.723	BV	0.73	343.81	36.56	50.58	
		Sum	679.68			

Chiral

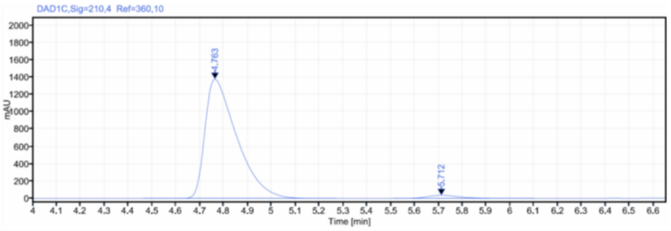

Signal: DAD1C,Sig=210,4 Ref=360,10

RT [min]	Type	Width [min]	Area	Height	Area%	Name
4.763	BV	0.81	12756.02	1377.62	97.39	
5.712	BV	0.59	342.07	36.97	2.61	
		Sum	13098.09			

Supplementary Fig. 19 SFC chromatograms of 4m. AD-3 column, MeOH/CO_2 = 10:90, 1.0 mL/min, 210 nm, t_{minor} = 5.7 min, t_{major} = 4.8 min. $[\alpha]_D^{20}$ +27.8 (c 1.61 in $CHCl_3$) 94% ee (*S*) (lit.[16] $[\alpha]_D^{20}$ +16.6 (c 0.73 in $CHCl_3$) 53% ee (*S*)).

(*S*)-(4-bromophenyl)(4-methoxyphenyl)methanol (4n)

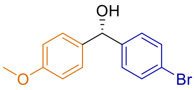

Known compound.[24] **Procedure A**, white solid, 34.3 mg, 59% yield, 95% ee. **Procedure B**, white solid, 34.2 mg, 59% yield, 91% ee. R_f = 0.45 (PE/EA = 4:1 v/v). Elution with PE/EA = 10:1 to 4:1 v/v. **^{1}H NMR** (500 MHz, Chloroform-*d*) δ 7.44 (d, J = 8.5 Hz, 2H), 7.27–7.21 (m, 4H), 6.86 (d, J = 8.8 Hz, 2H), 5.74 (s, 1H), 3.78 (s, 3H), 2.22 (s, 1H).

Racemic

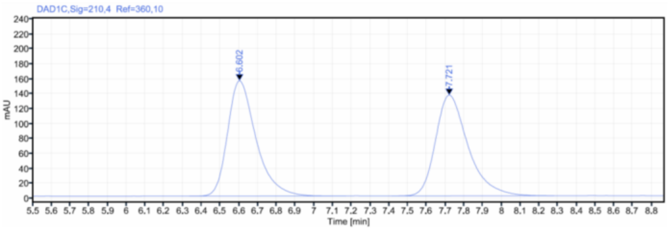

Signal: DAD1C,Sig=210,4 Ref=360,10

RT [min]	Type	Width [min]	Area	Height	Area%	Name
6.602	BV	0.83	1624.69	154.34	49.99	
7.721	BV	0.87	1625.40	134.84	50.01	
		Sum	3250.09			

Chiral

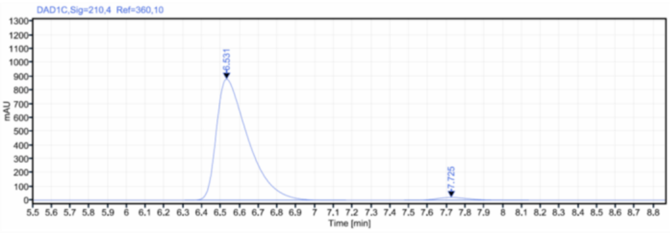

Signal: DAD1C,Sig=210,4 Ref=360,10

RT [min]	Type	Width [min]	Area	Height	Area%	Name
6.531	VV	0.86	10280.66	880.51	97.65	
7.725	VV	0.66	247.71	20.76	2.35	
		Sum	10528.37			

Supplementary Fig. 20 SFC chromatograms of 4n. AD-3 column, MeOH/CO_2 = 10:90, 1.0 mL/min, 210 nm, t_{minor} = 7.7 min, t_{major} = 6.5 min.

(*R*)-(4-methoxyphenyl)(4-(trifluoromethyl)phenyl)methanol (4o)

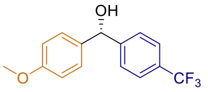

Known compound.[25] **Procedure A**, white solid, 40.9 mg, 73% yield, 94% ee. **Procedure B**, white solid, 40.5 mg, 72% yield, 96% ee. R_f = 0.45 (PE/EA = 4:1 v/v). Elution with PE/EA = 10:1 to 4:1 v/v. **^{1}H NMR** (500 MHz, Chloroform-*d*) δ 7.58 (d, *J* = 8.2 Hz, 2H), 7.48 (d, *J* = 8.5 Hz, 2H), 7.24 (d, *J* = 8.5 Hz, 2H), 6.86 (d, *J* = 8.8 Hz, 2H), 5.82 (s, 1H), 3.78 (s, 3H), 2.35 (s, 1H).

Racemic

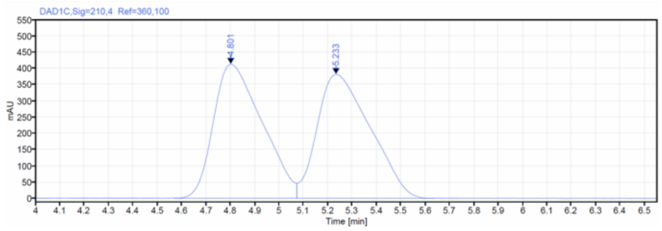

Signal: DAD1C,Sig=210,4 Ref=360,100

RT [min]	Type	Width [min]	Area	Height	Area%	Name
4.801	VV	0.50	5746.61	413.28	49.84	
5.233	VV	0.57	5782.65	382.15	50.16	
		Sum	11529.26			

Chiral

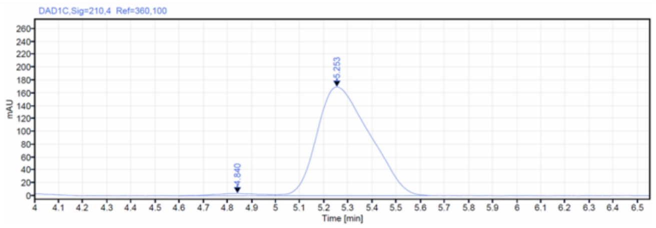

Signal: DAD1C,Sig=210,4 Ref=360,100

RT [min]	Type	Width [min]	Area	Height	Area%	Name
4.840	BV	0.40	47.11	3.62	1.81	
5.253	VB	0.64	2556.47	169.28	98.19	
		Sum	2603.58			

Supplementary Fig. 21 SFC chromatograms of 4o. OJ-3 column, MeOH/CO_2 = 5:95, 1.0 mL/min, 210 nm, t_{minor} = 4.8 min, t_{major} = 5.3 min.

(*S*)-(4-methoxyphenyl)(4-(trifluoromethoxy)phenyl)methanol (4p)

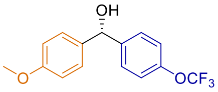

Procedure A, white solid, 45.1 mg, 76% yield, 93% ee. **Procedure B**, white solid, 44.7 mg, 75% yield, 95% ee. R_f = 0.45 (PE/EA = 4:1 v/v). Elution with PE/EA = 10:1 to 4:1 v/v. **^{1}H NMR** (500 MHz, Chloroform-*d*) δ 7.39 (d, *J* = 8.5 Hz, 2H), 7.26 (d, *J* = 8.6 Hz, 2H), 7.17 (d, *J* = 8.1 Hz, 2H), 6.87 (d, *J* = 8.7 Hz, 2H), 5.79 (s, 1H), 3.79 (s, 3H), 2.26 (s, 1H). **^{13}C NMR** (126 MHz, Chloroform-*d*) δ 159.28, 148.37, 142.59, 135.68, 127.91, 127.74, 120.87, 120.45 (q, *J* = 257.0 Hz), 114.02, 75.10, 55.26. **^{19}F NMR** (565 MHz, Chloroform-*d*) δ -57.85. **HRMS-APCI** (m/z): $[M-OH]^+$ calcd. for $[C_{15}H_{12}F_3O_2]^+$, 281.0784, found, 281.0788.

Racemic

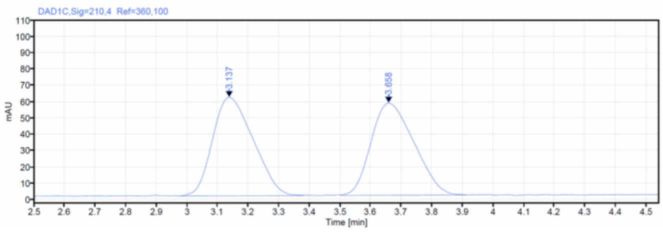

Signal: DAD1C,Sig=210,4 Ref=360,100

RT [min]	Type	Width [min]	Area	Height	Area%	Name
3.137	BV	0.41	555.34	60.38	50.19	
3.658	VB	0.41	551.04	56.59	49.81	
		Sum	1106.38			

Chiral

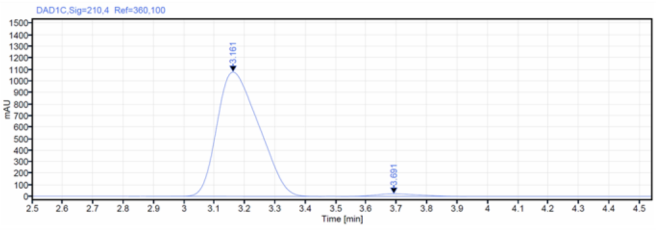

Signal: DAD1C,Sig=210,4 Ref=360,100

RT [min]	Type	Width [min]	Area	Height	Area%	Name
3.161	BV	0.50	10256.46	1078.28	97.84	
3.691	BV	0.41	226.84	22.30	2.16	
		Sum	10483.31			

Supplementary Fig. 22 SFC chromatograms of 4p-Cr. OJ-3 column, MeOH/CO_2 = 10:90, 1.0 mL/min, 210 nm, t_{minor} = 3.7 min, t_{major} = 3.2 min.

(*S*)-(4-((tert-butyldimethylsilyl)oxy)phenyl)(4-chlorophenyl)methanol (4q)

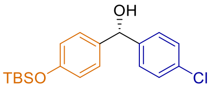

Procedure A, colorless oil, 31.6 mg, 45% yield, 82% ee. R_f = 0.55 (PE/EA = 6:1 v/v). Elution with PE/EA = 10:1 to 5:1 v/v. **^{1}H NMR** (500 MHz, Chloroform-*d*) δ 7.29 (s, 4H), 7.17 (d, *J* = 8.2 Hz, 2H), 6.79 (d, *J* = 8.6 Hz, 2H), 5.73 (d, *J* = 3.3 Hz, 1H), 2.25 (d, *J* = 3.5 Hz, 1H), 0.97 (s, 9H), 0.18 (s, 6H). **^{13}C NMR** (126 MHz, Chloroform-*d*) δ 155.35, 142.40, 136.27, 133.06, 128.47, 127.85, 127.78, 120.11, 75.19, 25.62, 18.15, -4.45. **HRMS-ESI** (m/z): $[M-OH]^+$ calcd. for $[C_{19}H_{24}ClOSi]^+$, 331.1279, found, 331.1284.

Racemic

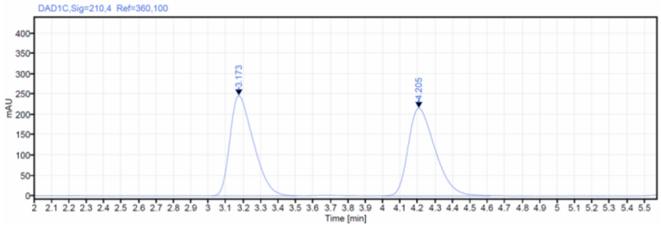

RT [min]	Type	Width [min]	Area	Height	Area%	Name
3.173	VV	0.49	2309.01	246.81	49.85	
4.205	VV	0.63	2323.17	216.14	50.15	
		Sum	4632.19			

Signal: DAD1C,Sig=210,4 Ref=360,100

Chiral

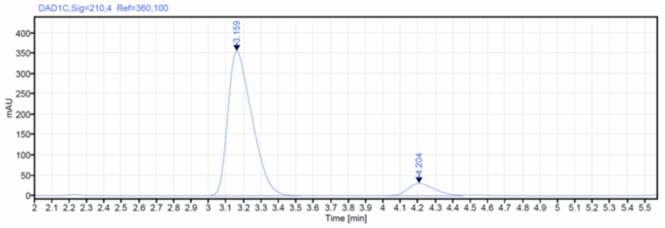

RT [min]	Type	Width [min]	Area	Height	Area%	Name
3.159	VV	0.53	3420.64	356.03	91.24	
4.204	BV	0.45	328.61	30.49	8.76	
		Sum	3749.25			

Signal: DAD1C,Sig=210,4 Ref=360,100

Supplementary Fig. 23 SFC chromatograms of 4q. AD-3 column, MeOH/CO_2 = 10:90, 1.0 mL/min, 254 nm, t_{minor} = 4.2 min, t_{major} = 3.2 min.

(*S*)-(4-(dimethylamino)phenyl)(4-methoxyphenyl)methanol (4r)

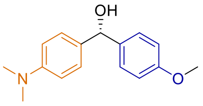

Known compound.[22] **Procedure A**, step (1) with 0.22 mmol **2**, white solid, 37.3 mg, 72% yield, 87% ee. R_f = 0.25 (PE/EA = 4:1 v/v). Elution with PE/EA = 6:1 to 3:1 v/v. **^{1}H NMR** (600 MHz, Chloroform-*d*) δ 7.29 (d, *J* = 8.4 Hz, 2H), 7.21 (d, *J* = 8.6 Hz, 2H), 6.86 (d, *J* = 8.8 Hz, 2H), 6.70 (d, *J* = 8.8 Hz, 2H), 5.74 (d, *J* = 2.6 Hz, 1H), 3.79 (s, 3H), 2.93 (s, 6H), 2.02 (d, *J* = 3.5 Hz, 1H).

Racemic

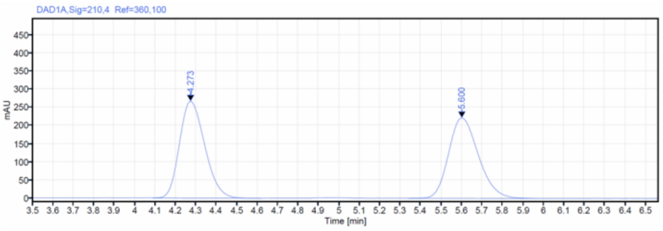

Signal: DAD1A,Sig=210,4 Ref=360,100

RT [min]	Type	Width [min]	Area	Height	Area%	Name
4.273	VB	0.53	2289.65	266.85	50.42	
5.600	VV	0.63	2251.63	221.79	49.58	
		Sum	4541.28			

Chiral

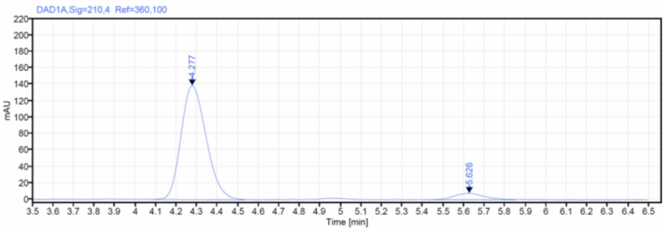

Signal: DAD1A,Sig=210,4 Ref=360,100

RT [min]	Type	Width [min]	Area	Height	Area%	Name
4.277	BV	0.46	1180.07	138.76	93.68	
5.626	BV	0.44	79.60	7.83	6.32	
		Sum	1259.67			

Supplementary Fig. 24 SFC chromatograms of 4r. IH-3 column, MeOH/CO_2 = 10:90, 1.0 mL/min, 210 nm, t_{minor} = 5.6 min, t_{major} = 4.3 min.

methyl (*R*)-4-((4-(dimethylamino)phenyl)(hydroxy)methyl)benzoate (4s)

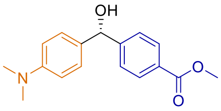

Known compound.[22] **Procedure A**, step (1) with 0.22 mmol **2**, pale yellow solid, 44.1 mg, 77% yield, 87% ee. R_f = 0.25 (PE/EA = 4:1 v/v). Elution with PE/EA = 6:1 to 3:1 v/v. **^{1}H NMR** (600 MHz, Chloroform-*d*) δ 7.99 (d, J = 8.1 Hz, 2H), 7.47 (d, J = 8.1 Hz, 2H), 7.18 (d, J = 8.5 Hz, 2H), 6.68 (d, J = 8.5 Hz, 2H), 5.80 (s, 1H), 3.89 (s, 3H), 2.93 (s, 6H), 2.20 (s, 1H).

Racemic

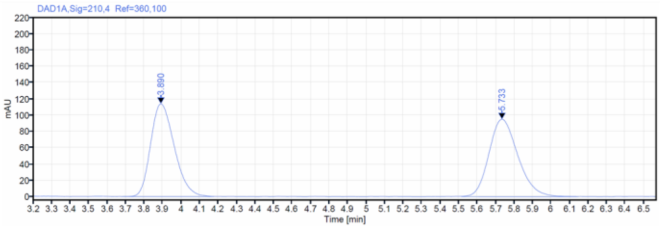

Signal: DAD1A,Sig=210,4 Ref=360,100

RT [min]	Type	Width [min]	Area	Height	Area%	Name
3.890	BV	0.49	1010.43	113.90	49.49	
5.733	VV	0.63	1031.32	95.04	50.51	
		Sum	2041.75			

Chiral

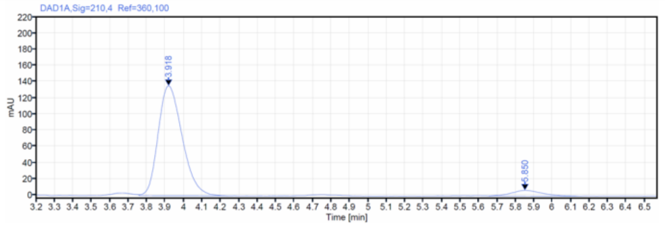

Signal: DAD1A,Sig=210,4 Ref=360,100

RT [min]	Type	Width [min]	Area	Height	Area%	Name
3.918	VB	0.48	1227.44	135.80	93.97	
5.850	VB	0.49	78.74	7.09	6.03	
		Sum	1306.18			

Supplementary Fig. 25 SFC chromatograms of 4s. IH-3 column, MeOH/CO_2 = 15:85, 1.0 mL/min, 210 nm, t_{minor} = 5.8 min, t_{major} = 3.9 min.

(*S*)-(4-fluorophenyl)(p-tolyl)methanol (4t)

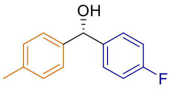

Known compound.[26] **Procedure A**, 32.8 mg, white solid, 76% yield, 87% ee. **Procedure B**, 31.5 mg, white solid, 73% yield, 94% ee. R_f = 0.55 (PE/EA = 4:1 v/v). Elution with PE/EA = 10:1 to 4:1 v/v. **^{1}H NMR** (500 MHz, Chloroform-*d*) δ 7.33 (dd, *J* = 8.3, 5.4 Hz, 2H), 7.23 (d, *J* = 8.1 Hz,

2H), 7.14 (d, J = 7.6 Hz, 2H), 7.00 (t, J = 8.7 Hz, 2H), 5.78 (d, J = 3.4 Hz, 1H), 2.33 (s, 3H), 2.21 (d, J = 3.5 Hz, 1H).

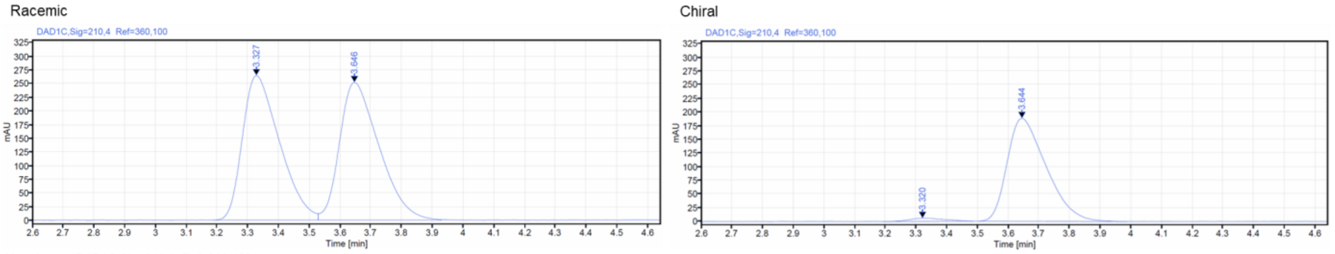

Racemic — Signal: DAD1C,Sig=210,4 Ref=360,100

RT [min]	Type	Width [min]	Area	Height	Area%	Name
3.327	BV	0.34	2216.53	264.73	49.65	
3.646	VV	0.40	2247.40	252.10	50.35	
		Sum	4463.93			

Chiral — Signal: DAD1C,Sig=210,4 Ref=360,100

RT [min]	Type	Width [min]	Area	Height	Area%	Name
3.320	BB	0.29	47.78	6.08	2.79	
3.644	BV	0.48	1663.92	188.90	97.21	
		Sum	1711.70			

Supplementary Fig. 26 SFC chromatograms of 4t. AD-3 column, MeOH/CO_2 = 10:90, 1.0 mL/min, 210 nm, t_{minor} = 3.3 min, t_{major} = 3.6 min.

(*S*)-o-tolyl(2-(trifluoromethyl)phenyl)methanol (**4u**)

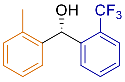

Procedure A, step (2) at room temperature, colorless oil, 44.1 mg, 83% yield, 99% ee. R_f = 0.55 (PE/EA = 6:1 v/v). Elution with PE/EA = 10:1 to 6:1 v/v. **^{1}H NMR** (500 MHz, Chloroform-*d*) δ 7.69 (d, J = 7.3 Hz, 1H), 7.53 – 7.44 (m, 2H), 7.44 – 7.36 (m, 2H), 7.27 – 7.18 (m, 2H), 7.17 – 7.12 (m, 1H), 6.39 (d, J = 2.5 Hz, 1H), 2.27 (d, J = 3.5 Hz, 1H), 2.14 (s, 3H). **^{13}C NMR** (126 MHz, Chloroform-*d*) δ 141.24, 140.14, 135.38, 132.27, 130.59, 129.21, 128.11 (q, J = 30.3 Hz), 127.89, 127.71, 126.18, 126.10 (q, J = 5.9 Hz), 125.87, 124.53 (q, J = 274.1 Hz), 68.60, 19.11. **GCMS-EI** (m/z): $[M]^+$ calcd. for $[C_{15}H_{13}F_3O]^+$, 266.1, found, 266.1.

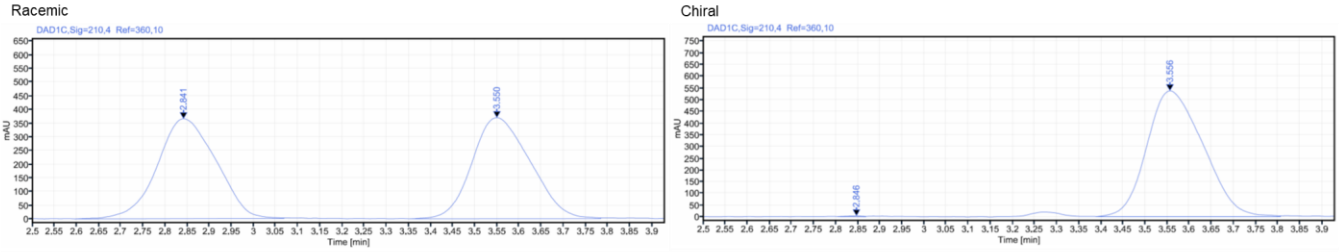

Racemic — Signal: DAD1C,Sig=210,4 Ref=360,10

RT [min]	Type	Width [min]	Area	Height	Area%	Name
2.841	BV	0.47	3362.96	365.17	49.97	
3.550	BV	0.43	3367.42	369.62	50.03	
		Sum	6730.38			

Chiral — Signal: DAD1C,Sig=210,4 Ref=360,10

RT [min]	Type	Width [min]	Area	Height	Area%	Name
2.846	VV	0.06	9.53	3.54	0.20	
3.556	BV	0.42	4717.89	536.20	99.80	
		Sum	4727.42			

Supplementary Fig. 27 SFC chromatograms of 4u. OD-3 column, MeOH/CO_2 = 10:90, 1.0 mL/min, 210 nm, t_{minor} = 2.8 min, t_{major} = 3.6 min.

(*S*)-(3-fluoro-5-methoxyphenyl)(4-methoxyphenyl)methanol (**4v**)

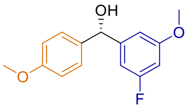

Procedure A, colorless oil, 38.7 mg, 74% yield, 82% ee. **Procedure B**, colorless oil, 36.5 mg, 70% yield, 95% ee. R_f = 0.40 (PE/EA = 4:1 v/v). Elution with PE/EA = 10:1 to 4:1 v/v. **^{1}H NMR** (500 MHz, Chloroform-*d*) δ 7.25 (d, J = 8.5 Hz, 2H), 6.86 (d, J = 8.8 Hz, 2H), 6.75 – 6.71 (m, 1H), 6.70 – 6.65 (m, 1H), 6.49 (dt, J = 10.5, 2.3 Hz, 1H), 5.71 (d, J = 3.1 Hz, 1H), 3.78 (s, 3H), 3.76 (s, 3H), 2.27 (d, J = 3.4 Hz, 1H). **^{13}C NMR** (126 MHz, Chloroform-*d*) δ 163.58 (d, J = 245.1 Hz), 160.86 (d, J = 11.3 Hz), 159.25, 147.14 (d, J = 8.8 Hz), 135.50, 127.91, 113.98, 107.77 (d, J = 2.6 Hz), 105.52 (d, J = 22.6 Hz), 100.34 (d, J = 25.3 Hz), 75.31 (d, J = 2.3 Hz), 55.50, 55.26. **^{19}F NMR** (471 MHz, $CDCl_3$) δ -111.37. **HRMS-APCI** (m/z): $[M\text{-}OH]^+$ calcd. for $[C_{15}H_{14}FO_2]^+$, 245.0972, found, 245.0987.

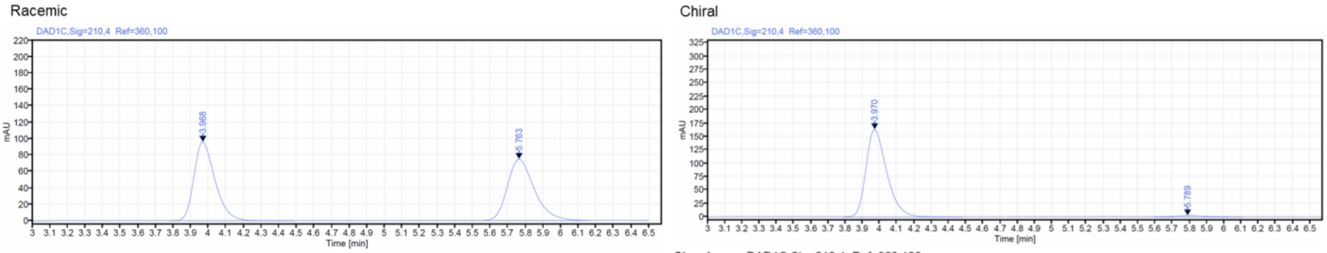

Racemic — Signal: DAD1C,Sig=210,4 Ref=360,100

RT [min]	Type	Width [min]	Area	Height	Area%	Name
3.968	VV	0.71	795.57	95.86	50.06	
5.763	BV	0.68	793.56	74.76	49.94	
		Sum	1589.13			

Chiral — Signal: DAD1C,Sig=210,4 Ref=360,100

RT [min]	Type	Width [min]	Area	Height	Area%	Name
3.970	VV	0.69	1376.98	163.87	97.56	
5.789	VV	0.51	34.39	2.67	2.44	
		Sum	1411.37			

Supplementary Fig. 28 SFC chromatograms of 4v. AD-3 column, MeOH/CO_2 = 10:90, 1.5 mL/min, 210 nm, t_{minor} = 5.8 min, t_{major} = 4.0 min.

(*S*)-naphthalen-2-yl(5,6,7,8-tetrahydronaphthalen-2-yl)methanol (**4w**)

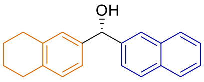

Procedure A, 48.9 mg, white solid, 85% yield, 84% ee. R_f = 0.55 (PE/EA = 4:1 v/v). Elution with PE/EA = 10:1 to 4:1 v/v. **^{1}H NMR** (500 MHz, Chloroform-*d*) δ 7.90 (s, 1H), 7.85–7.82 (m, 1H), 7.81–7.76 (m, 2H), 7.49–7.40 (m, 3H), 7.12–7.08 (m, 2H), 7.02 (d, *J* = 8.4 Hz, 1H), 5.92 (d, *J* = 3.3 Hz, 1H), 2.73 (d, *J* = 3.2 Hz, 4H), 2.28 (d, *J* = 3.5 Hz, 1H), 1.76 (dt, *J* = 6.7, 3.5 Hz, 4H). **^{13}C NMR** (126 MHz, Chloroform-*d*) δ 141.28, 140.84, 137.36, 136.71, 133.25, 132.80, 129.32, 128.19, 128.05, 127.63, 127.38, 126.08, 125.82, 124.78, 124.74, 123.92, 76.27, 29.43, 29.12, 23.14, 23.12. **HRMS-APCI** (m/z): $[M\text{-}OH]^+$ calcd. for $[C_{21}H_{19}]^+$, 271.1481, found, 271.1482.

Racemic | Chiral

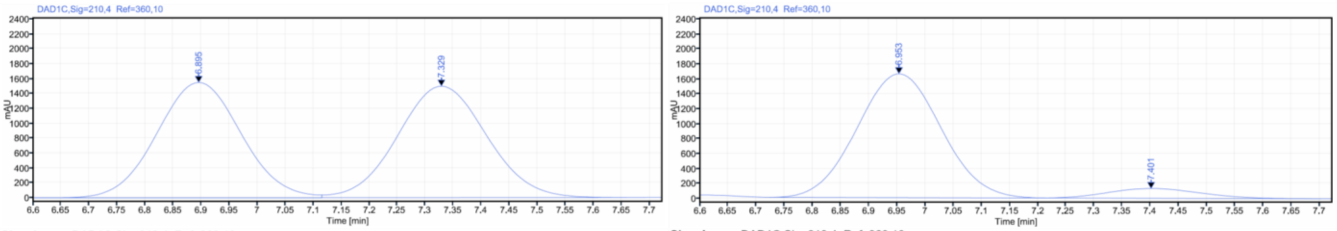

Racemic — Signal: DAD1C,Sig=210,4 Ref=360,10

RT [min]	Type	Width [min]	Area	Height	Area%	Name
6.895	BV	0.51	16230.94	1548.56	49.43	
7.329	VV	0.54	16602.75	1497.61	50.57	
		Sum	32833.69			

Chiral — Signal: DAD1C,Sig=210,4 Ref=360,10

RT [min]	Type	Width [min]	Area	Height	Area%	Name
6.953	BV	0.49	17253.64	1657.60	92.02	
7.401	VB	0.49	1496.89	131.34	7.98	
		Sum	18750.52			

Supplementary Fig. 29 SFC chromatograms of 4w. IC-3 column, MeOH/CO_2 = 10:90, 1.0 mL/min, 210 nm, t_{minor} = 7.4 min, t_{major} = 6.9 min.

(R)-(4-chlorophenyl)(o-tolyl)methanol (**4x**)

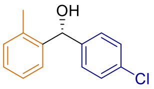

Known compound.[27] **Procedure A**, white solid, 29.7 mg, 64% yield, 74% ee. R_f = 0.55 (PE/EA = 4:1 v/v). Elution with PE/EA = 10:1 to 4:1 v/v. **^{1}H NMR** (500 MHz, Chloroform-d) δ 7.45 (dd, *J* = 7.3, 1.6 Hz, 1H), 7.32–7.25 (m, 4H), 7.22 (qd, *J* = 7.2, 1.7 Hz, 2H), 7.15 (dd, *J* = 7.2, 1.5 Hz, 1H), 5.98 (s, 1H), 2.25 (s, 3H), 2.13 (s, 1H).

Racemic | Chiral

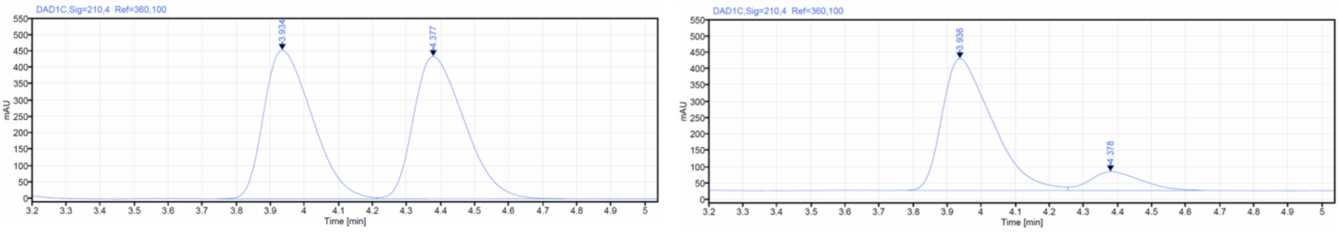

Racemic — Signal: DAD1C,Sig=210,4 Ref=360,100

RT [min]	Type	Width [min]	Area	Height	Area%	Name
3.934	BV	0.47	4482.30	454.35	49.91	
4.377	VB	0.55	4498.35	435.28	50.09	
		Sum	8980.65			

Chiral — Signal: DAD1C,Sig=210,4 Ref=360,100

RT [min]	Type	Width [min]	Area	Height	Area%	Name
3.936	BV	0.51	4125.45	403.22	87.29	
4.378	VV	0.41	600.85	57.59	12.71	
		Sum	4726.30			

Supplementary Fig. 30 SFC chromatograms of 4x. AD-3 column, MeOH/CO_2 = 10:90, 1.0 mL/min, 210 nm, t_{minor} = 4.4 min, t_{major} = 3.9 min.

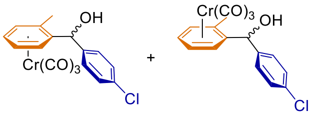

Procedure A**, 4x-Cr**, 50.23:49.77, >20:1 dr and 2.9:1 dr. (Racemate from $NaBH_4$ reduction, 50.43:49.58, 1.2:1 dr and 1:1.2 dr)

Racemic | Chiral

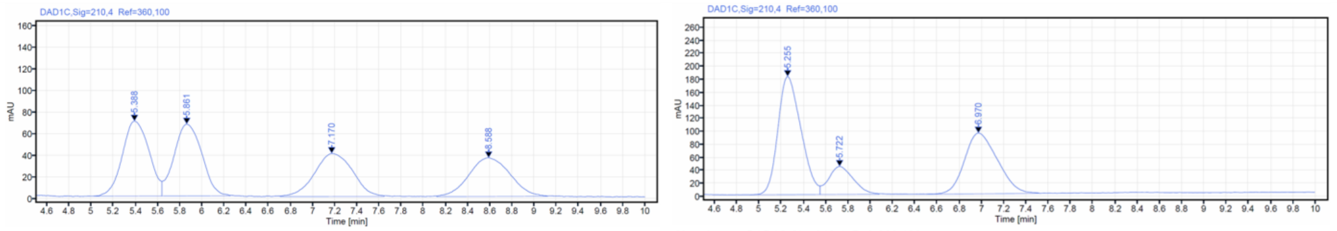

Racemic — Signal: DAD1C,Sig=210,4 Ref=360,100

RT [min]	Type	Width [min]	Area	Height	Area%	Name
5.388	BV	0.62	1169.30	69.46	27.78	
5.861	VV	0.62	1160.41	66.52	27.57	
7.170	VV	0.94	953.31	39.91	22.65	
8.588	VV	1.00	926.45	35.84	22.01	
		Sum	4209.47			

Chiral — Signal: DAD1C,Sig=210,4 Ref=360,100

RT [min]	Type	Width [min]	Area	Height	Area%	Name
5.255	BV	0.63	2613.59	181.62	50.23	
5.722	VV	0.53	666.02	43.12	12.80	
6.970	BB	1.03	1923.38	93.36	36.97	
		Sum	5202.98			

Supplementary Fig. 31 SFC chromatograms of 4x-Cr. AS-3 column, MeOH/CO2 = 7:93, 1.0 mL/min, 210 nm, t_{minor}= 8.5 min, t_{major} = 5.3 min and t_{minor}= 5.7 min, t_{major} = 7.0 min.

When **1x** was directly subjected to the same hydrogenation conditions, the enantioselectivity was much lower (28% ee).

Racemic

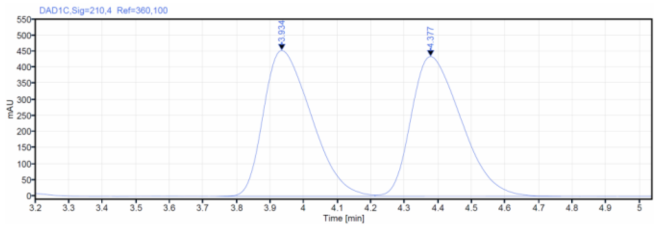

Signal: DAD1C,Sig=210,4 Ref=360,100

RT [min]	Type	Width [min]	Area	Height	Area%	Name
3.934	BV	0.47	4482.30	454.35	49.91	
4.377	VB	0.55	4498.35	435.28	50.09	
		Sum	8980.65			

Chiral

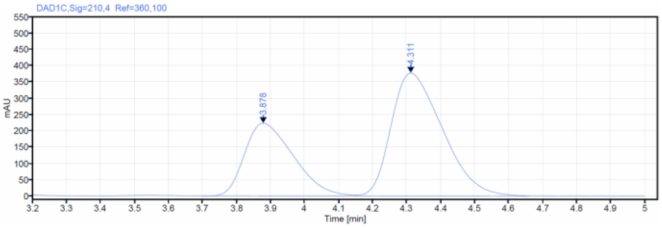

Signal: DAD1C,Sig=210,4 Ref=360,100

RT [min]	Type	Width [min]	Area	Height	Area%	Name
3.878	BV	0.45	2216.99	222.54	35.98	
4.311	VV	0.51	3945.02	377.23	64.02	
		Sum	6162.00			

Supplementary Fig. 32 SFC chromatograms of 4x (directly hydrogenation from 1x). AD-3 column, MeOH/CO_2 = 10:90, 1.0 mL/min, 210 nm, t_{major} = 3.9 min, t_{minor} = 4.4 min.

The kinetic resolution of **1x-Cr** can be achieved by shortening the reaction time. After 15 minutes of reaction at 0 °C, the conversion of the reaction was 45%, and two pair of diastereomers (**4x-Cr**, 91.37:8.63) with > 20:1 dr and 1.6:1 dr were obtained respectively. The recovered **1x-Cr** was also obtained with 68% ee. After removing the Cr(CO)3 unit, we obtained the free alcohol **4x** with 92% ee.

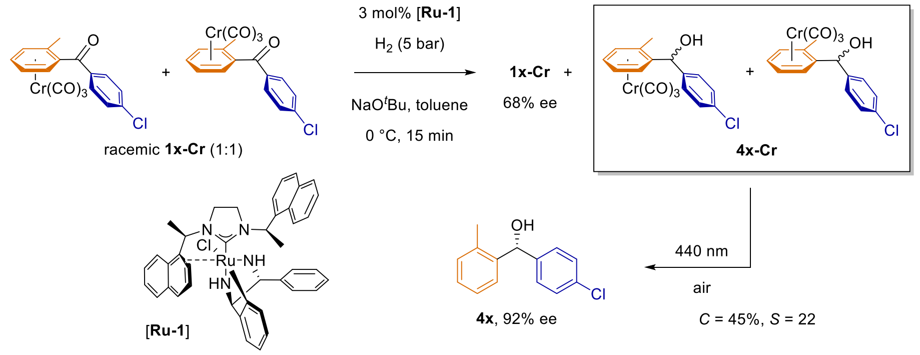

Supplementary Fig. 33 Kinetic resolution of 1x-Cr. Conversion (*C*). *S* factor (*S*) = ln[(1 − *C*) (1 − ee$_s$)]/ln[(1 − *C*) (1 + ee$_s$)].

Racemic

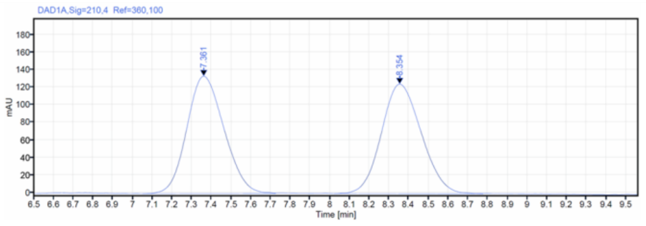

Signal: DAD1A,Sig=210,4 Ref=360,100

RT [min]	Type	Width [min]	Area	Height	Area%	Name
7.361	BV	0.67	1753.70	133.72	49.88	
8.354	BV	0.75	1761.83	124.85	50.12	
		Sum	3515.54			

Chiral

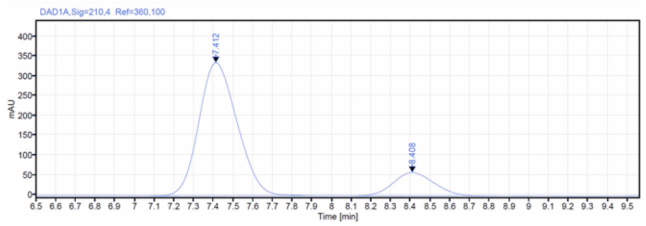

Signal: DAD1A,Sig=210,4 Ref=360,100

RT [min]	Type	Width [min]	Area	Height	Area%	Name
7.412	BB	0.83	4514.99	335.75	84.26	
8.408	BV	0.66	843.57	59.02	15.74	
		Sum	5358.55			

Supplementary Fig. 34 SFC chromatograms of recovered 1x-Cr (kinetic resolution of 1x-Cr). OD-3 column, MeOH/CO_2 = 5:95, 1.0 mL/min, 210 nm, t_{major} = 7.4 min, t_{minor} = 8.4 min.

Racemic

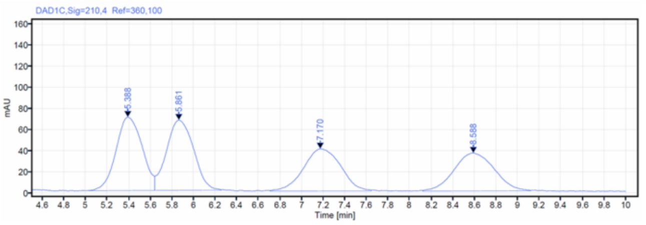

Signal: DAD1C,Sig=210,4 Ref=360,100

RT [min]	Type	Width [min]	Area	Height	Area%	Name
5.388	BV	0.62	1169.30	69.46	27.78	
5.861	VV	0.62	1160.41	66.52	27.57	
7.170	VV	0.94	953.31	39.91	22.65	
8.588	VV	1.00	926.45	35.84	22.01	
		Sum	4209.47			

Chiral

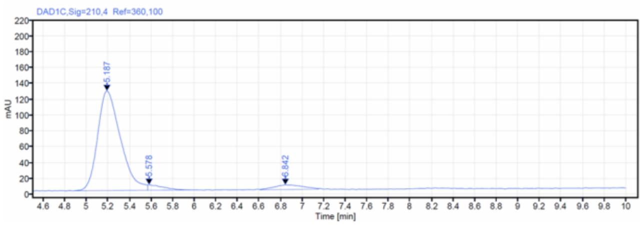

Signal: DAD1C,Sig=210,4 Ref=360,100

RT [min]	Type	Width [min]	Area	Height	Area%	Name
5.187	BV	0.69	1850.98	125.76	91.37	
5.578	MB m	0.16	67.45	6.87	3.33	
6.842	VV	0.55	107.45	5.82	5.30	
		Sum	2025.88			

Supplementary Fig. 35 SFC chromatograms of 4x-Cr (kinetic resolution of 1x-Cr). AS-3 column, MeOH/CO_2 = 7:93, 1.0 mL/min, 210 nm, t_{minor}= 8.5 min, t_{major} = 5.2 min and t_{minor}= 5.6 min, t_{major} = 6.8 min. (Racemate from $NaBH_4$ reduction, 50.43:49.58, 1.2:1 dr and 1:1.2 dr)

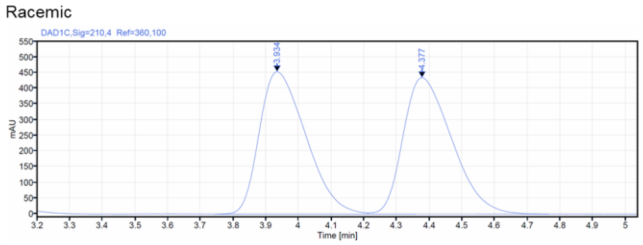

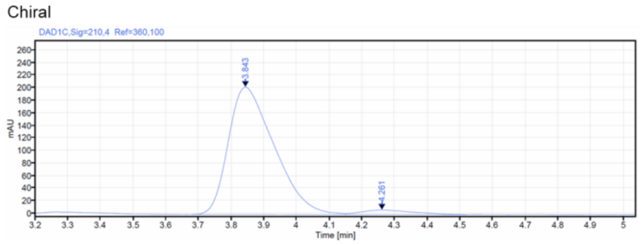

Signal: DAD1C,Sig=210,4 Ref=360,100

RT [min]	Type	Width [min]	Area	Height	Area%	Name
3.934	BV	0.47	4482.30	454.35	49.91	
4.377	VB	0.55	4498.35	435.28	50.09	
		Sum	8980.65			

Signal: DAD1C,Sig=210,4 Ref=360,100

RT [min]	Type	Width [min]	Area	Height	Area%	Name
3.843	BV	0.47	1970.40	202.62	96.30	
4.261	VV	0.37	75.70	7.28	3.70	
		Sum	2046.10			

Supplementary Fig. 36 SFC chromatograms of 4x (kinetic resolution of 1x-Cr). AD-3 column, MeOH/CO_2 = 10:90, 1.0 mL/min, 210 nm, t_{major} = 3.8 min, t_{minor} = 4.3 min.

(*S*)-phenyl(3-(trifluoromethyl)phenyl)methanol (4y)

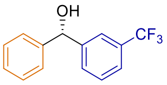

Known compound.[28] **Procedure B**, white solid, 34.9 mg, 69% yield, 96% ee. R_f = 0.55 (PE/EA = 4:1 v/v). Elution with PE/EA = 10:1 to 4:1 v/v. **^{1}H NMR** (500 MHz, Chloroform-*d*) δ 7.70 (s, 1H), 7.53 (t, J = 8.0 Hz, 2H), 7.44 (t, J = 7.7 Hz, 1H), 7.36 (d, J = 4.4 Hz, 4H), 7.34–7.26 (m, 1H), 5.87 (d, J = 3.4 Hz, 1H), 2.34 (d, J = 3.5 Hz, 1H).

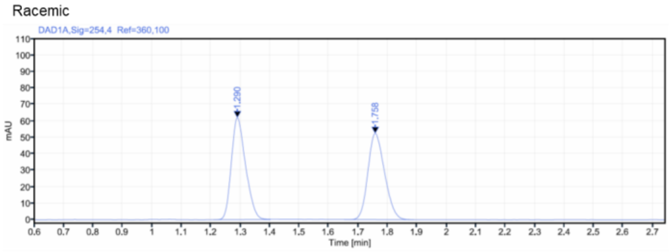

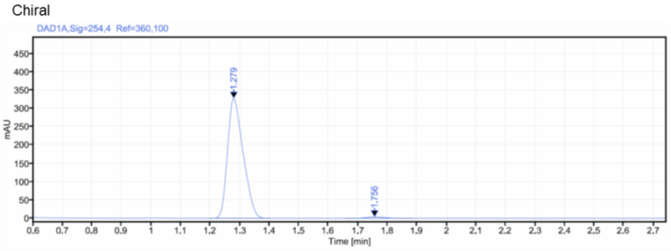

Signal: DAD1A,Sig=254,4 Ref=360,100

RT [min]	Type	Width [min]	Area	Height	Area%	Name
1.290	BV	0.19	208.42	62.04	50.24	
1.758	BB	0.24	206.44	52.48	49.76	
		Sum	414.86			

Signal: DAD1A,Sig=254,4 Ref=360,100

RT [min]	Type	Width [min]	Area	Height	Area%	Name
1.279	BV	0.21	1200.82	327.62	98.49	
1.756	VV	0.23	18.36	4.23	1.51	
		Sum	1219.18			

Supplementary Fig. 37 SFC chromatograms of 4y-Cr. OD-3 column, MeOH/CO_2 = 7:93, 1.0 mL/min, 254 nm, t_{minor} = 1.8 min, t_{major} = 1.3 min.

(*S*)-4-(hydroxy(phenyl)methyl)phenyl trifluoromethanesulfonate (1z)

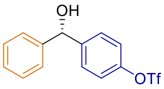

Procedure B, white solid, 51.6 mg, 78% yield, 94% ee. R_f = 0.45 (PE/EA = 4:1 v/v). Elution with PE/EA = 10:1 to 4:1 v/v. **^{1}H NMR** (500 MHz, Chloroform-d) δ 7.46 (d, J = 8.4 Hz, 2H), 7.39–7.27 (m, 5H), 7.23 (d, J = 8.8 Hz, 2H), 5.84 (d, J = 3.2 Hz, 1H), 2.35 (d, J = 3.4 Hz, 1H). **^{13}C NMR** (126 MHz, Chloroform-*d*) δ 148.68, 144.10, 143.03, 128.79, 128.28, 128.13, 126.59, 121.27, 118.71 (q, J = 320.7 Hz), 75.38. **^{19}F NMR** (471 MHz, Chloroform-d) δ -72.84. **HRMS** (m/z): $[M-OH]^+$ calcd. for $[C_{14}H_{10}F_3O_3S]^+$, 315.0297 found, 315.0308.

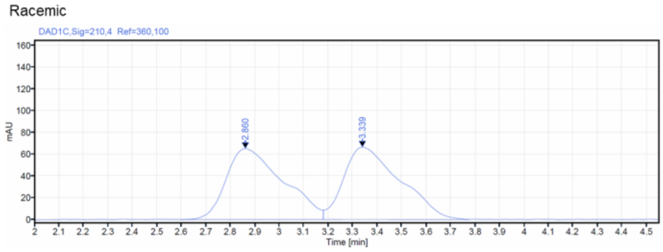

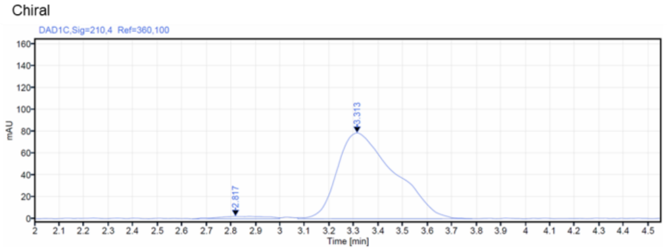

Signal: DAD1C,Sig=210,4 Ref=360,100

RT [min]	Type	Width [min]	Area	Height	Area%	Name
2.860	BV	0.58	1052.69	65.26	49.66	
3.339	VV	0.59	1066.91	66.61	50.34	
		Sum	2119.60			

Signal: DAD1C,Sig=210,4 Ref=360,100

RT [min]	Type	Width [min]	Area	Height	Area%	Name
2.817	BV	0.36	33.92	2.42	2.59	
3.313	VB	0.71	1275.08	78.81	97.41	
		Sum	1309.00			

Supplementary Fig. 38 SFC chromatograms of 4z. AD-3 column, MeOH/CO_2 = 5:95, 1.0 mL/min, 210 nm, t_{minor} = 2.8 min, t_{major} = 3.3 min.

(*S*)-(3,4-dichlorophenyl)(phenyl)methanol (4aa)

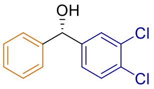

Known compound.[16] **Procedure B**, white solid, 40.4 mg, 80% yield, 97% ee. R_f = 0.50 (PE/EA = 4:1 v/v). Elution with PE/EA = 10:1 to 4:1 v/v. **^{1}H NMR** (500 MHz, Chloroform-*d*) δ 7.47 (d, J = 1.8 Hz, 1H), 7.38–7.26 (m, 6H), 7.15 (dd, J = 8.3, 1.8 Hz, 1H), 5.71 (s, 1H), 2.53 (s, 1H).

Racemic

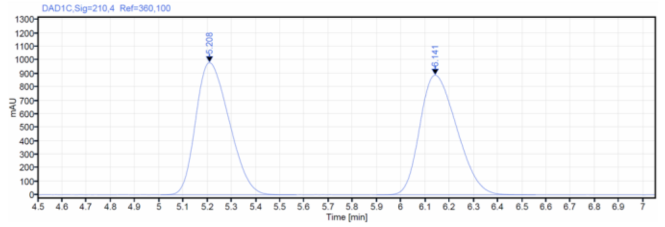

Signal: DAD1C,Sig=210,4 Ref=360,100

RT [min]	Type	Width [min]	Area	Height	Area%	Name
5.208	VV	0.56	9386.00	981.96	49.92	
6.141	BB	0.66	9414.70	889.01	50.08	
		Sum	18800.70			

Chiral

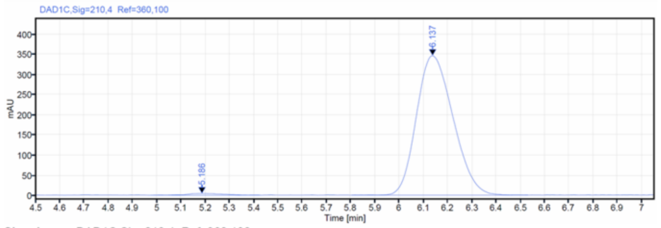

Signal: DAD1C,Sig=210,4 Ref=360,100

RT [min]	Type	Width [min]	Area	Height	Area%	Name
5.186	BB	0.45	45.52	4.31	1.24	
6.137	BB	0.73	3626.13	345.94	98.76	
		Sum	3671.65			

Supplementary Fig. 39 SFC chromatograms of 4aa. OJ-3 column, MeOH/CO_2 = 10:90, 1.0 mL/min, 210 nm, t_{minor} = 5.2 min, t_{major} = 6.1 min.

(*S*)-naphthalen-2-yl(phenyl)methanol (**4ab**)

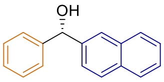

Known compound.[15] **Procedure B**, white solid, 35.1 mg, 75% yield, 92% ee. R_f = 0.50 (PE/EA = 4:1 v/v). Elution with PE/EA = 10:1 to 4:1 v/v. **^{1}H NMR** (500 MHz, Chloroform-*d*) δ 7.88 (s, 1H), 7.84–7.76 (m, 3H), 7.49–7.43 (m, 2H), 7.43–7.40 (m, 3H), 7.35–7.31 (m, 2H), 7.28–7.24 (m, 1H), 5.98 (d, J = 3.2 Hz, 1H), 2.36 (d, J = 3.5 Hz, 1H).

Racemic

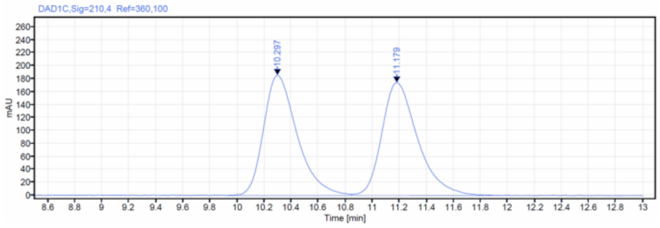

Signal: DAD1C,Sig=210,4 Ref=360,100

RT [min]	Type	Width [min]	Area	Height	Area%	Name
10.297	BV	0.92	3205.84	185.69	49.93	
11.179	VV	1.11	3215.07	174.15	50.07	
		Sum	6420.92			

Chiral

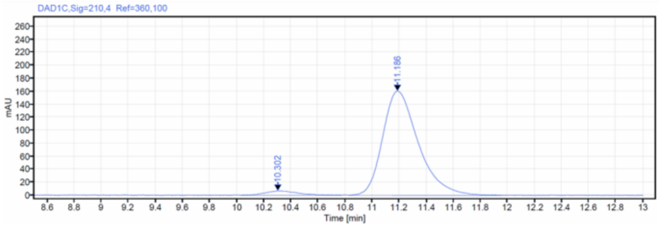

Signal: DAD1C,Sig=210,4 Ref=360,100

RT [min]	Type	Width [min]	Area	Height	Area%	Name
10.302	VV	0.75	120.47	7.06	3.93	
11.186	VV	1.13	2946.42	160.50	96.07	
		Sum	3066.89			

Supplementary Fig. 40 SFC chromatograms of 4ab. AD-3 column, MeOH/CO_2 = 10:90, 1.0 mL/min, 210 nm, t_{minor} = 10.3 min, t_{major} = 11.2 min.

(*S*)-(4-fluorophenyl)(4-vinylphenyl)methanol (**4ac**)

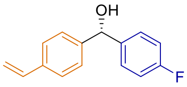

Procedure B, white solid, 28.1 mg, 62% yield, 93% ee. R_f = 0.55 (PE/EA = 4:1 v/v). Elution with PE/EA = 10:1 to 4:1 v/v. **^{1}H NMR** (500 MHz, Chloroform-*d*) δ 7.38 (d, J = 8.2 Hz, 2H), 7.34–7.29 (m, 4H), 7.01 (t, J = 8.7 Hz, 2H), 6.69 (dd, J = 17.6, 10.9 Hz, 1H), 5.80 (d, J = 2.4 Hz, 1H), 5.73 (dd, J = 17.6, 0.8 Hz, 1H), 5.24 (dd, J = 10.9, 0.8 Hz, 1H), 2.26 (d, J = 3.2 Hz, 1H). **^{13}C NMR** (126 MHz, Chloroform-*d*) δ 162.16 (d, J = 245.9 Hz), 143.14, 139.44 (d, J = 3.1 Hz), 137.07, 136.31, 128.19 (d, J = 8.1 Hz), 126.62, 126.40, 115.30 (d, J = 21.5 Hz), 114.09, 75.35. **^{19}F NMR** (471 MHz, $CDCl_3$) δ -114.95. **GCMS-EI** (m/z): $[M]^+$ calcd. for $[C_{15}H_{13}FO]^+$, 228.1, found, 228.1.

Racemic

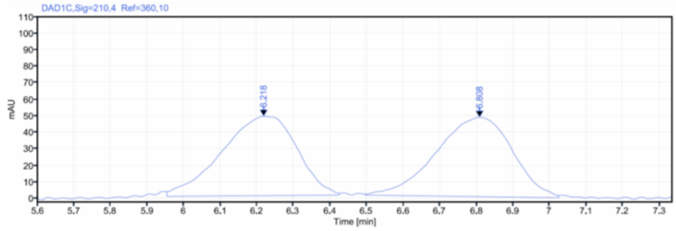

Signal: DAD1C,Sig=210,4 Ref=360,10

RT [min]	Type	Width [min]	Area	Height	Area%	Name
6.218	VV	0.47	685.72	48.05	49.71	
6.808	VV	0.53	693.68	48.01	50.29	
		Sum	1379.40			

Chiral

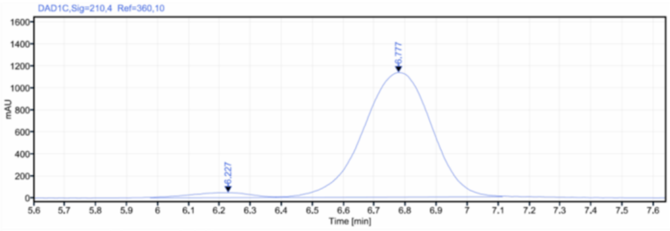

Signal: DAD1C,Sig=210,4 Ref=360,10

RT [min]	Type	Width [min]	Area	Height	Area%	Name
6.227	VV	0.41	619.94	45.23	3.44	
6.777	VV	0.73	17384.63	1133.96	96.56	
		Sum	18004.57			

Supplementary Fig. 41 SFC chromatograms of 4ac. IC-3 column, MeOH/CO_2 = 10:90, 1.0 mL/min, 202 nm, t_{minor} = 6.2 min, t_{major} = 6.8 min.

(1-(4-fluorophenyl)vinyl)benzene chromium tricarbonyl (3a-Cr)

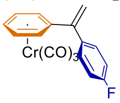

Yellow solid. 54.7 mg, 82% yield. The regioisomeric ratio was > 10:1. R_f = 0.40 (PE/EA = 20:1 v/v). Elution with PE/EA = 30:1 to 20:1 v/v. **^{1}H NMR** (500 MHz, Chloroform-*d*) δ 7.35 (t, J = 6.9 Hz, 2H), 7.07 (t, J = 8.5 Hz, 2H), 5.69 (s, 1H), 5.50–5.25 (m, 6H). **^{13}C NMR** (126 MHz, Chloroform-*d*) δ 232.55,162.73 (d, J = 247.8 Hz), 144.40, 135.45 (d, J = 3.4 Hz), 130.15 (d, J = 8.1 Hz), 117.31, 115.39 (d, J = 21.6 Hz), 108.45, 93.25, 92.54, 91.28. **^{19}F NMR** (471 MHz, Chloroform-*d*) δ -113.48. **HRMS-APCI** (m/z): $[M]^+$ calcd. for $[C_{17}H_{12}CrFO_3]^+$, 335.0170, found, 335.0166.

(*S*)-1-fluoro-4-(1-phenylethyl)benzene (5a)

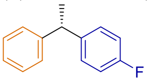

Known compound.[12] **Procedure A**, colorless oil, 32.7 mg, 82% yield, 79% ee. R_f = 0.80 (PE). Elution with PE. **^{1}H NMR** (500 MHz, Chloroform-*d*) δ 7.28 (t, J = 7.5 Hz, 1H), 7.22–7.14 (m, 3H), 6.96 (t, J = 8.4 Hz, 1H), 4.13 (q, J = 7.1 Hz, 1H), 1.62 (d, J = 7.2 Hz, 2H).

Racemic

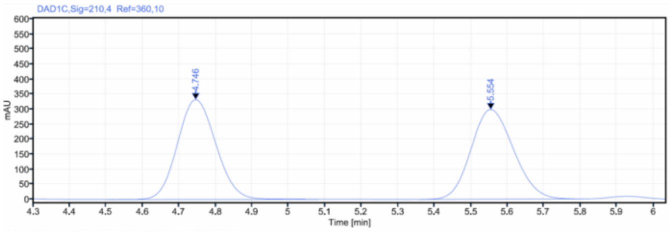

Signal: DAD1C,Sig=210,4 Ref=360,10

RT [min]	Type	Width [min]	Area	Height	Area%	Name
4.746	BV	0.58	2444.56	332.95	50.23	
5.554	VB	0.45	2422.29	298.97	49.77	
		Sum	4866.85			

Chiral

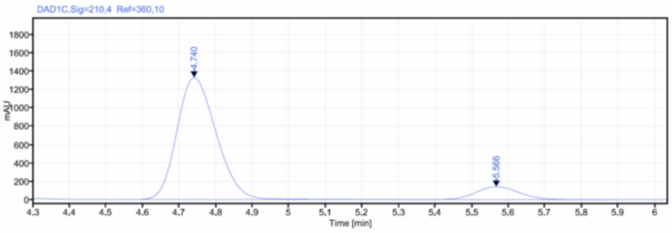

Signal: DAD1C,Sig=210,4 Ref=360,10

RT [min]	Type	Width [min]	Area	Height	Area%	Name
4.740	BV	0.83	9935.50	1325.54	89.55	
5.566	VV	0.51	1159.01	139.75	10.45	
		Sum	11094.51			

Supplementary Fig. 42 SFC chromatograms of 5a-Cr. OJ-3 column, MeOH/CO_2 = 7:93, 1.0 mL/min, 210 nm, t_{minor} = 5.6 min, t_{major} = 4.7 min.

(1-(4-chlorophenyl)vinyl)benzene chromium tricarbonyl (3b-Cr)

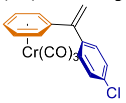

Yellow solid. 56.7 mg, 81% yield. The regioisomeric ratio was > 10:1. R_f = 0.40 (PE/EA = 20:1 v/v). Elution with PE/EA = 30:1 to 20:1 v/v. **^{1}H NMR** (500 MHz, Chloroform-d) δ 7.35 (d, J = 8.3 Hz, 2H), 7.32 (d, J = 8.1 Hz, 2H), 5.70 (s, 1H), 5.46 (s, 1H), 5.41 (d, J = 6.1 Hz, 2H), 5.37 (d, J = 5.7 Hz, 1H), 5.32 (t, J = 5.8 Hz, 2H). **^{13}C NMR** (126 MHz, Chloroform-*d*) δ 232.48, 144.34, 137.87, 134.33, 129.74, 128.66, 117.59, 108.13, 93.22, 92.53, 91.24. **HRMS-APCI** (m/z): $[M]^+$ calcd. for $[C_{17}H_{12}ClCrO_3]^+$, 350.9875, found, 350.9886.

(*S*)-1-chloro-4-(1-phenylethyl)benzene (5b)

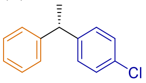

Known compound.[12] **Procedure A**, colorless oil, 35.0 mg, 81% yield, 73% ee. R_f = 0.80 (PE). Elution with PE. **^{1}H NMR** (500 MHz, Chloroform-*d*) δ 7.28 (t, J = 7.6 Hz, 1H), 7.26–7.22 (m, 1H), 7.21–7.17 (m, 2H), 7.14 (d, J = 8.4 Hz, 1H), 4.12 (q, J = 7.2 Hz, 1H), 1.61 (d, J = 7.2 Hz, 1H).

The enantiomeric excess was determined by SFC analysis:

Racemic

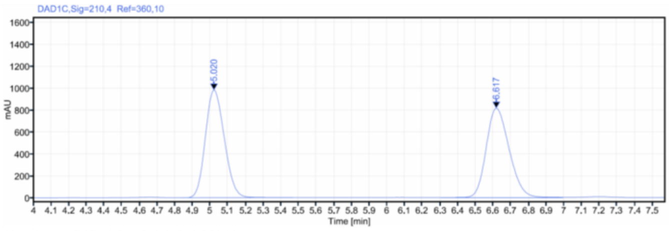

Signal: DAD1C,Sig=210,4 Ref=360,10

RT [min]	Type	Width [min]	Area	Height	Area%	Name
5.020	BV	0.63	7311.06	982.69	49.89	
6.617	BV	0.69	7344.38	815.78	50.11	
		Sum	14655.44			

Chiral

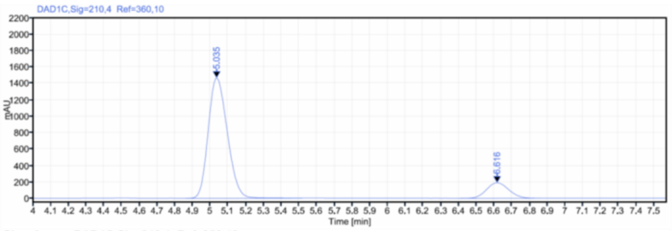

Signal: DAD1C,Sig=210,4 Ref=360,10

RT [min]	Type	Width [min]	Area	Height	Area%	Name
5.035	BV	0.66	10968.29	1467.31	86.88	
6.616	BV	0.46	1656.79	190.05	13.12	
		Sum	12625.08			

Supplementary Fig. 43 SFC chromatograms of 5b. OJ-3 column, MeOH/CO_2 = 7:93, 1.0 mL/min, 210 nm, t_{minor} = 6.6 min, t_{major} = 5.0 min.

(1-(4-(trifluoromethyl)phenyl)vinyl)benzene chromium tricarbonyl (3c-Cr)

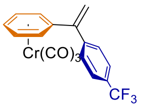

Yellow solid. 62.5 mg, 81% yield. The regioisomeric ratio was > 10:1. R_f = 0.40 (PE/EA = 20:1 v/v). Elution with PE/EA = 30:1 to 20:1 v/v. **^{1}H NMR** (500 MHz, Chloroform-*d*) δ 7.65 (d, J = 7.9 Hz, 2H), 7.51 (d, J = 8.0 Hz, 2H), 5.78 (s, 1H), 5.51 (s, 1H), 5.37 (d, J = 24.5 Hz, 5H). **^{13}C NMR** (126 MHz, Chloroform-*d*) δ 232.46, 144.47, 143.14, 130.56 (q, J = 32.5 Hz), 128.93, 125.56 (q, J = 3.9 Hz), 124.09 (q, J = 272.2 Hz), 118.52, 107.61, 93.13, 92.62, 91.30. **^{19}F NMR** (565 MHz, Chloroform-*d*) δ -62.64. **HRMS-APCI** (m/z): $[M]^+$ calcd. for $[C_{18}H_{12}CrF_3O_3]^+$, 385.0138, found, 385.0142.

(*S*)-1-(1-phenylethyl)-4-(trifluoromethyl)benzene (**5c**)

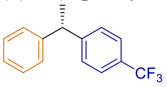

Known compound.[12] **Procedure A**, colorless oil, 40.7 mg, 81% yield, 77% ee. R_f = 0.80 (PE). Elution with PE. **^{1}H NMR** (500 MHz, Chloroform-*d*) δ 7.31–7.24 (m, 2H), 7.24–7.12 (m, 5H), 6.95 (t, J = 8.7 Hz, 2H), 4.12 (q, J = 7.2 Hz, 1H), 1.61 (d, J = 7.2 Hz, 3H).

Racemic

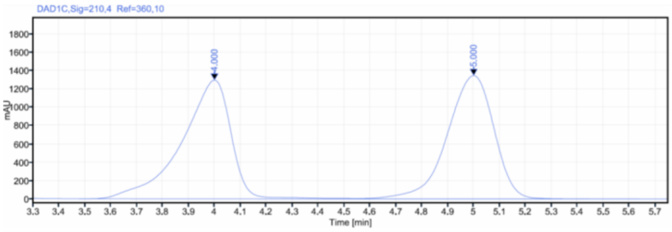

Signal: DAD1C,Sig=210,4 Ref=360,10

RT [min]	Type	Width [min]	Area	Height	Area%	Name
4.000	BV	1.08	17100.62	1294.50	50.39	
5.000	VB	1.08	16835.01	1344.72	49.61	
		Sum	33935.64			

Chiral

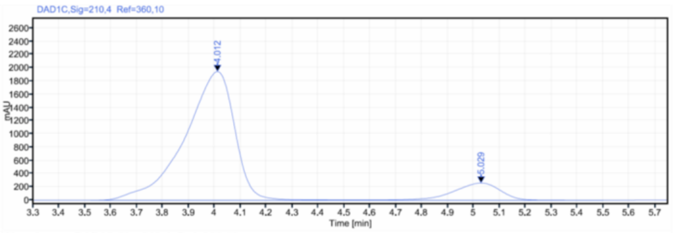

Signal: DAD1C,Sig=210,4 Ref=360,10

RT [min]	Type	Width [min]	Area	Height	Area%	Name
4.012	BV	1.08	25881.70	1944.69	88.77	
5.029	VB	0.90	3273.93	260.13	11.23	
		Sum	29155.63			

Supplementary Fig. 44 SFC chromatograms of 5c-Cr. OJ-3 column, MeOH/CO_2 = 7:93, 1.0 mL/min, 210 nm, t_{minor} = 5.0 min, t_{major} = 4.0 min.

2-(1-(2-(trifluoromethyl)phenyl)vinyl)toluene chromium tricarbonyl (3d-Cr)

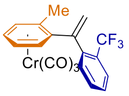

Yellow solid. 67.5 mg, 85% yield. The regioisomeric ratio was > 10:1. R_f = 0.45 (PE/EA = 20:1 v/v). Elution with PE/EA = 30:1 to 20:1 v/v. **^{1}H NMR** (500 MHz, Chloroform-*d*) δ 7.68 (d, J = 7.7 Hz, 1H), 7.56 (t, J = 7.4 Hz, 1H), 7.43 (t, J = 7.8 Hz, 2H), 5.80 (s, 1H), 5.67 (s, 1H), 5.53–5.38 (m, 2H), 5.11 (t, J = 6.1 Hz, 2H), 2.13 (s, 3H). **^{13}C NMR** (126 MHz, Chloroform-*d*) δ 232.98, 142.44, 139.93, 132.13, 131.86, 128.20, 127.87 (q, J = 30.2 Hz), 126.57 (q, J = 5.6 Hz), 125.12, 123.92 (q, J = 274.0 Hz), 111.10, 109.36, 98.03, 94.87, 92.05, 88.13, 20.35. **^{19}F NMR** (565 MHz, Chloroform-*d*) δ -57.00. **HRMS-APCI** (m/z): $[M]^+$ calcd. for $[C_{19}H_{14}CrF_3O_3]^+$, 399.0295, found, 399.0286.

(*S*)-1-methyl-2-(1-(2-(trifluoromethyl)phenyl)ethyl)benzene (**5d**)

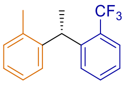

Procedure A, step (2) at room temperature, colorless oil, 45.1 mg, 85% yield, 99% ee. R_f = 0.80 (PE). Elution with PE. **^{1}H NMR** (500 MHz, Chloroform-*d*) δ 7.65 (d, J = 7.6 Hz, 1H), 7.42–7.34 (m, 2H), 7.29–7.24 (m, 2H), 7.25–7.22 (m, 1H), 7.16 (td, J = 7.4, 1.2 Hz, 1H), 7.12 (d, J = 7.4 Hz, 2H), 4.68 (q, J = 7.0 Hz, 1H), 2.10 (s, 3H), 1.62 (d, J = 7.0 Hz, 3H). **^{13}C NMR** (126 MHz, Chloroform-*d*) δ 145.39 (q, J = 1.7 Hz), 142.86, 136.67, 131.98, 130.74, 129.04, 127.94 (q, J = 29.6 Hz), 126.63, 126.45, 126.11 (q, J = 6.1 Hz), 125.96, 125.76, 124.79 (q, J = 274.1 Hz), 37.35, 22.57, 19.73. **^{19}F NMR** (471 MHz, Chloroform-*d*) δ -58.95. **GCMS-EI** (m/z): $[M]^+$ calcd. for $[C_{16}H_{15}F_3]^+$, 264.1, found, 264.1.

The enantiomeric excess was determined by HPLC analysis:

Racemic

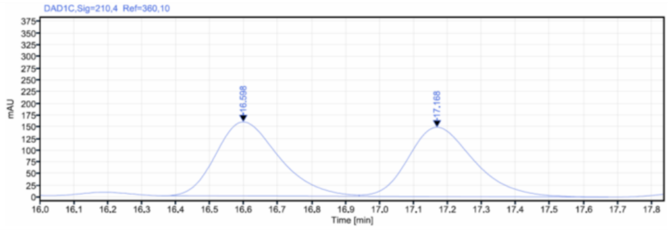

Signal: DAD1C,Sig=210,4 Ref=360,10

RT [min]	Type	Width [min]	Area	Height	Area%	Name
16.598	BV	0.58	2050.20	158.16	50.78	
17.168	VB	0.73	1987.17	148.11	49.22	
		Sum	4037.38			

Chiral

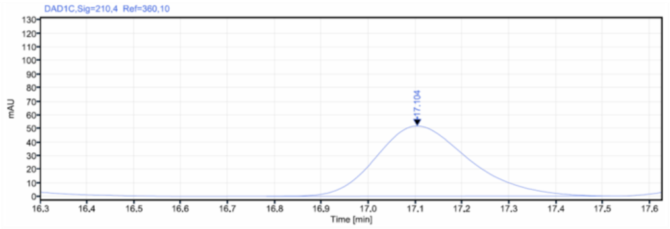

Signal: DAD1C,Sig=210,4 Ref=360,10

RT [min]	Type	Width [min]	Area	Height	Area%	Name
17.104	VB	0.73	712.39	51.70	100.00	
		Sum	712.39			

Supplementary Fig. 45 SFC chromatograms of 5d-Cr. IH-3 + IBN-3 column, MeOH/hexane = 2:98, 1.0 mL/min, 210 nm, t_{minor} = 16.6 min, t_{major} = 17.1 min.

4 Computational Studies

Density Functional Theory (DFT) calculation of the thermodynamic data was conducted with Gaussian 16 C.01[29] and ORCA 5.0.3[30, 31] Structures were first optimized at PBE0-D3(BJ)/def2SVP[32-35] level with SMD[36] implicit solvent model using toluene as solvent in Gaussian. Vibrational analysis was done at the optimization level, with the result that no imaginary vibration frequency for substrates and products, and only one imaginary frequency for the transition states were given. Free energy correction of the optimized structures was obtained by directly reading from the output file of vibrational analysis. The transition states were further checked by instinct reaction coordinates calculation. Single point energy was calculated in ORCA, at PWPB95-D3(BJ)/def2-QZVPP[32, 34, 35, 37] level, still using SMD implicit solvent model and toluene as solvent. The free energies were obtained by directly summing of the single point energy and the free energy correction. (Cartesian coordinates of optimized structure see Supplementary Data 1)

To get structural and energetical understanding of the impact to rotation of aryl–carbonyl C–C bond with or without $Cr(CO)_3$ ligation, relaxed scan of dihedral angles calculation were taken at the optimization level. The relaxed scan of dihedral angles calculation proceeded with 6° stepsize, and 30 steps. The output electronic energy was directly used. (For detailed calculation data, see Supplementary Data 2)

To give explanation of the enantioselectivity of the hydrogenation, and what role that the coordinated $Cr(CO)_3$ part play, Independent gradient model based on Hirshfeld partition (IGMH)[38] analysis of the transition states were taken. This analysis was taken with Multiwfn[39], by using the wavefunction after optimization from the .fchk file (transformed from .chk). Molecular structure and IGMH isosurface were drawn with VMD[40], using the gird data in the .cub file generated by Multiwfn. Atomic charge population and the conceptual DFT calculation were also conducted with Multiwfn.

Supplementary Table 1 Distortion and interaction energies (kcal/mol) at PBE0-D3(BJ)/def2SVP level.

Entry	TS	$\Delta E_{dist\text{-}sub}$	$\Delta E_{dist\text{-}cat}$	ΔE_{dist}	ΔE_{int}
**1**	**TS-3**	9.0	3.8	12.8	-13.4
**2**	**TS-4**	6.9	2.5	9.4	-12.2

We also used activation strain model[41, 42] to analyze activation barriers that determine reaction rates. the transition state energy depends upon ΔE_{dist} and ΔE_{int}, By definition, $\Delta E^{\ddagger} = \Delta E_{dist} + \Delta E_{int}$; therefore, since ΔE_{int} and $\Delta E_{dist\text{-}cat}$ are approximately the same, as in **TS-3** and **TS-4**, the distortion energies of the substrate become the determining factor, which is consistent with the results of elaxed scan of dihedral angels calculation.

Supplementary Table 2 Atomic charge population.

Entry	Atom	Hirshfeld Charge	ADCH Charge	CM5 Charge
**1**	C1 (**1a**)	0.1520	0.2304	0.1853
**2**	O1 (**1a**)	-0.2507	-0.3179	-0.3009
**3**	C1 (**1a-Cr**)	0.1591	0.2505	0.1910
**4**	O1 (**1a-Cr**)	-0.2382	-0.2961	-0.2894

Atomic charge population was a powerful tool for providing quantities information for increased electron-withdrawing effect by $Cr(CO)_3$ η^6-coordination, we calculated various atomic charge population including Hirshfeld atomic charge[43], ADCH atomic charge[44] and CM5 atomic charge[45]. And the result shown that the raise of positive atomic charge after $(CrCO)_3$ η^6-coordination.

Supplementary Table 3 Vertical electron affinity and localized Fukui indexes (f+) of 1a and 1a-Cr.

Entry	Compound	VEA (eV)	f+	
**1**	**1a**	0.6520	C1	0.0386
			O1	0.1225
**2**	**1a-Cr**	0.9468	C1	0.1019
			O1	0.1154

Despite of the atomic charge, orbital factor (interaction between d orbitals of Cr center and the π system) should be one of another concern too. So, we further performed the conceptual density functional analysis[46] to provide information from another aspect. Result shown that the raise of vertical electron affinity of **1a-Cr** and C1 localized f+ to provide information for electron withdrawing effect provided by η^6-coordination of $Cr(CO)_3$.

5 NMR Spectra of Compounds

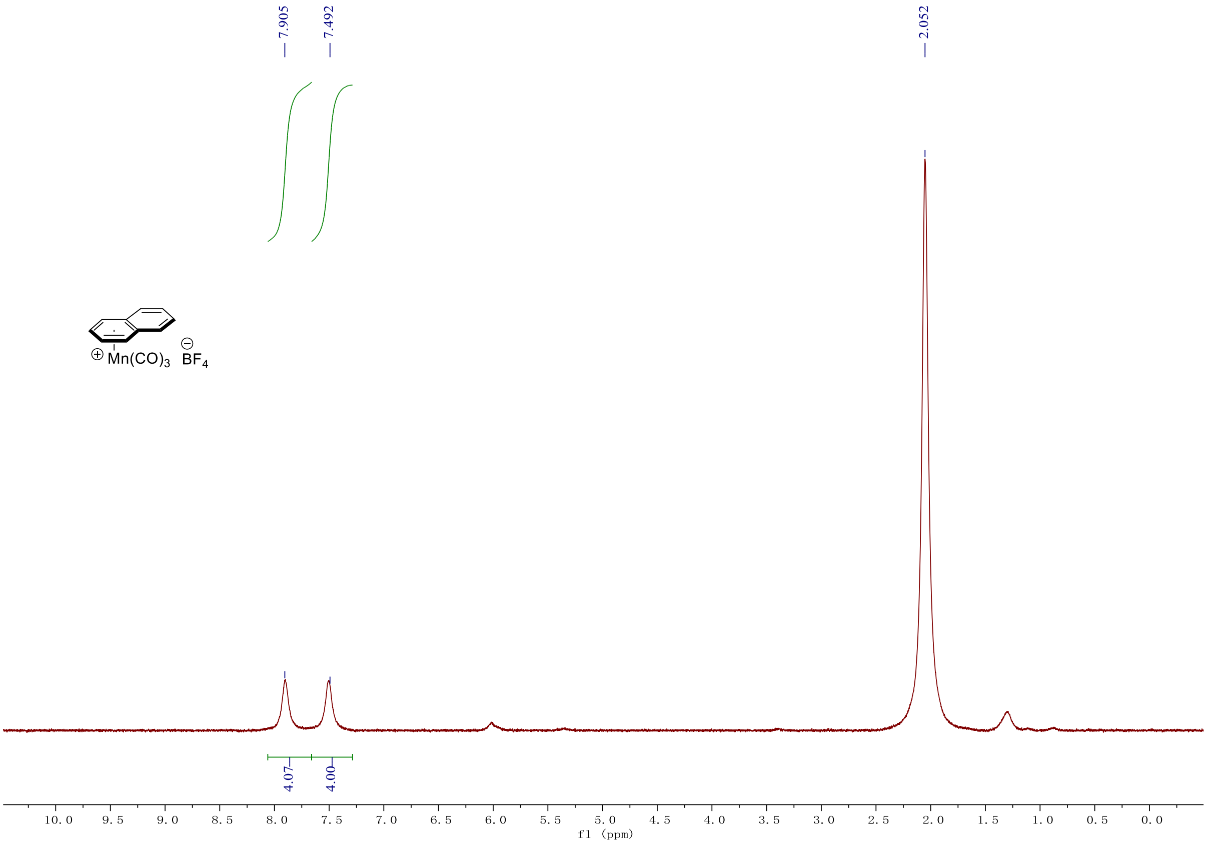

Supplementary Fig. 46 ^{1}H NMR (500 MHz, Acetone-d_6) of [Mn(CO)$_3$(naphthalene)]BF$_4$.

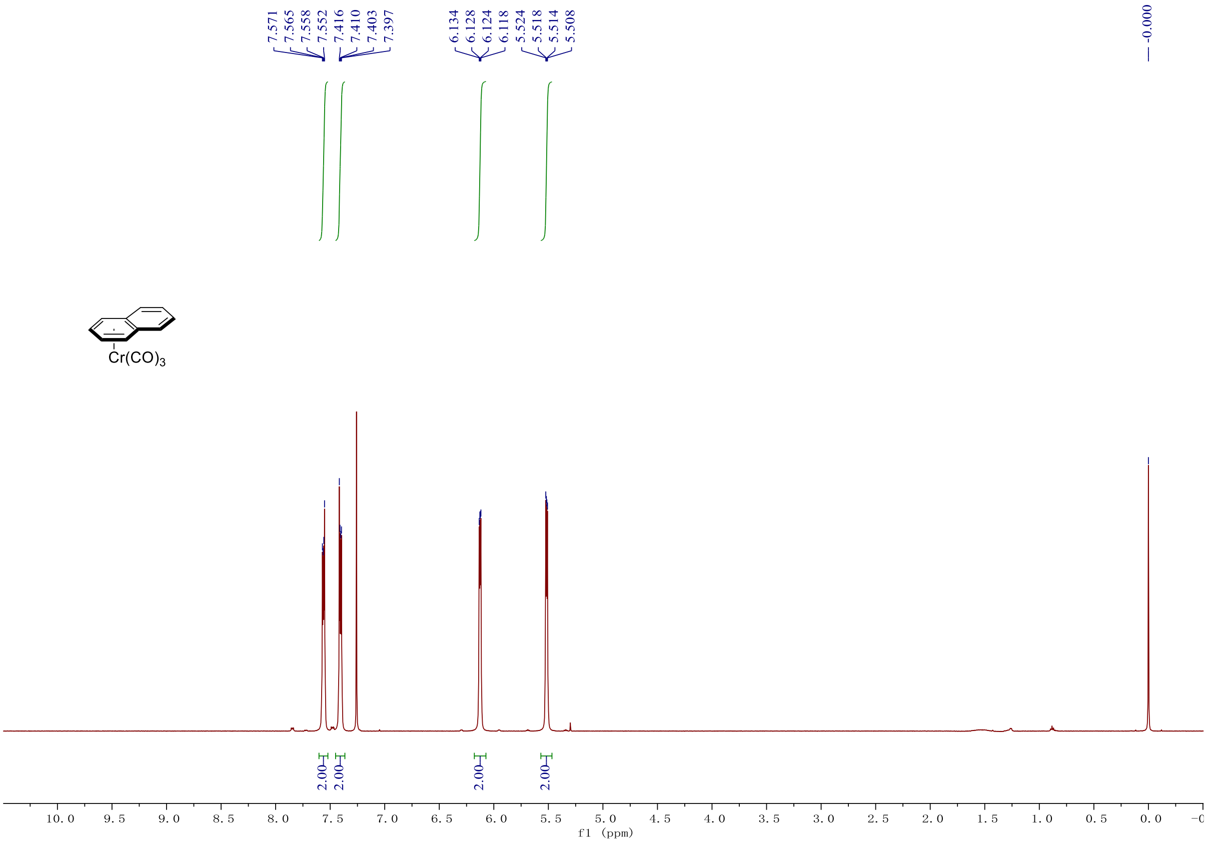

Supplementary Fig. 47 ^{1}H NMR (500 MHz, Chloroform-d) of Cr(CO)$_3$(naphthalene).

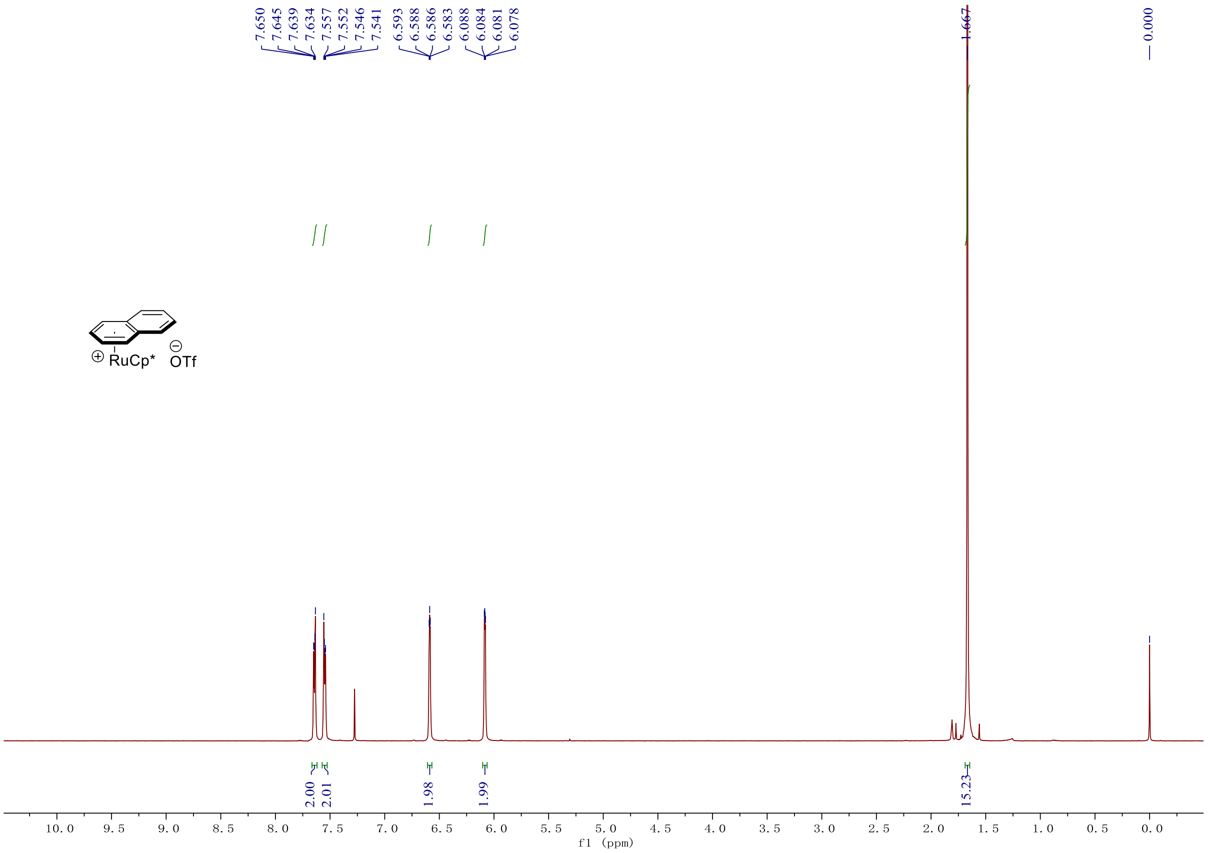

Supplementary Fig. 48 ^{1}H NMR (600 MHz, Chloroform-*d*) of [Cp*Ru(naphthalene)]OTf.

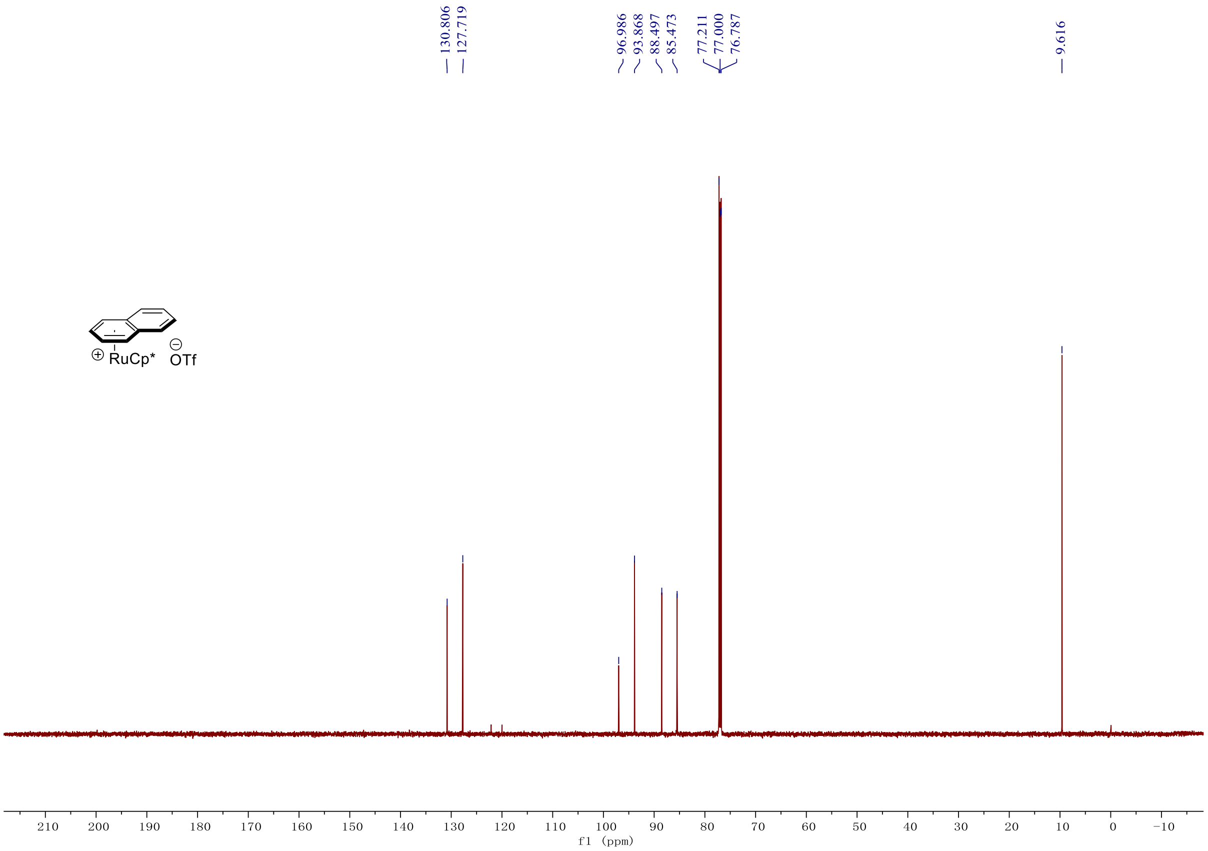

Supplementary Fig. 49 ^{13}C NMR (151 MHz, Chloroform-*d*) of [Cp*Ru(naphthalene)]OTf.

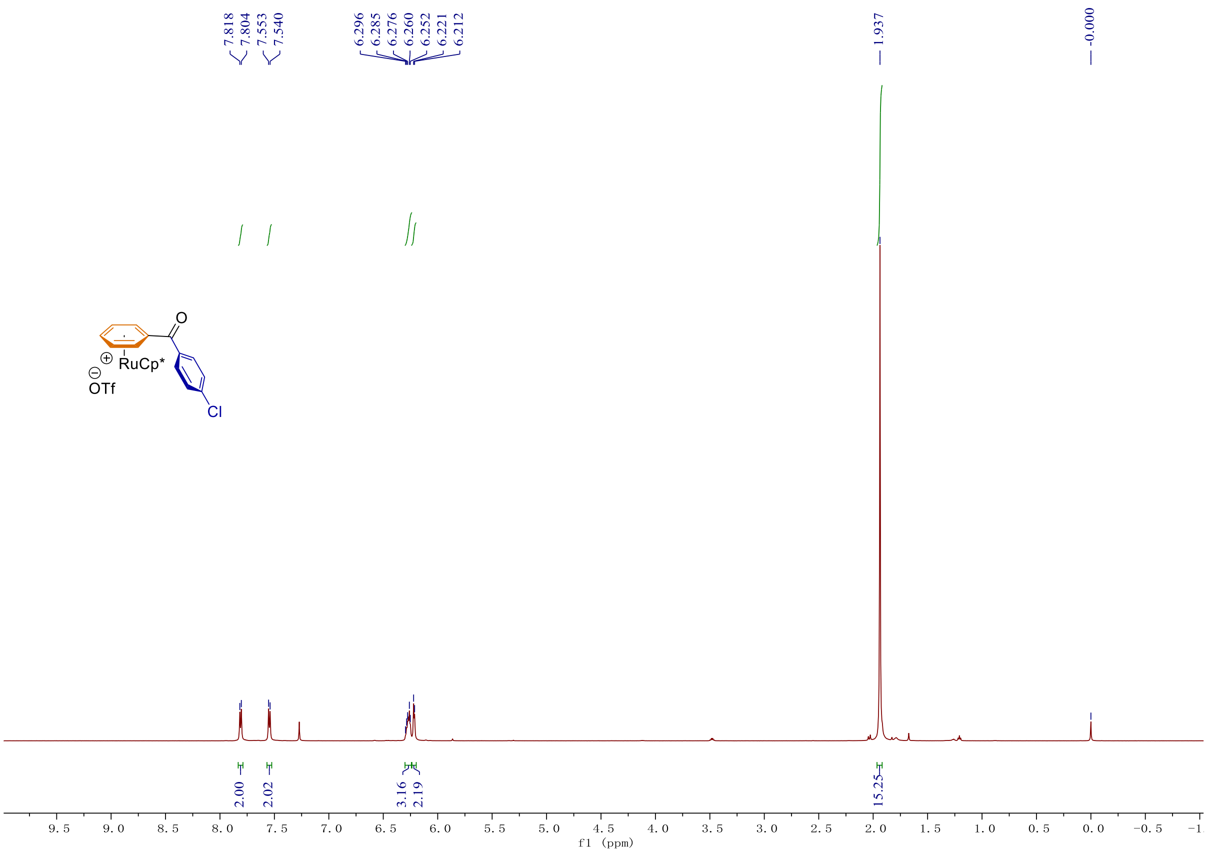

Supplementary Fig. 50 ^{1}H NMR (600 MHz, Chloroform-*d*) of [Cp*Ru(4-chlorobenzoylbenzene)]OTf (1a-Ru).

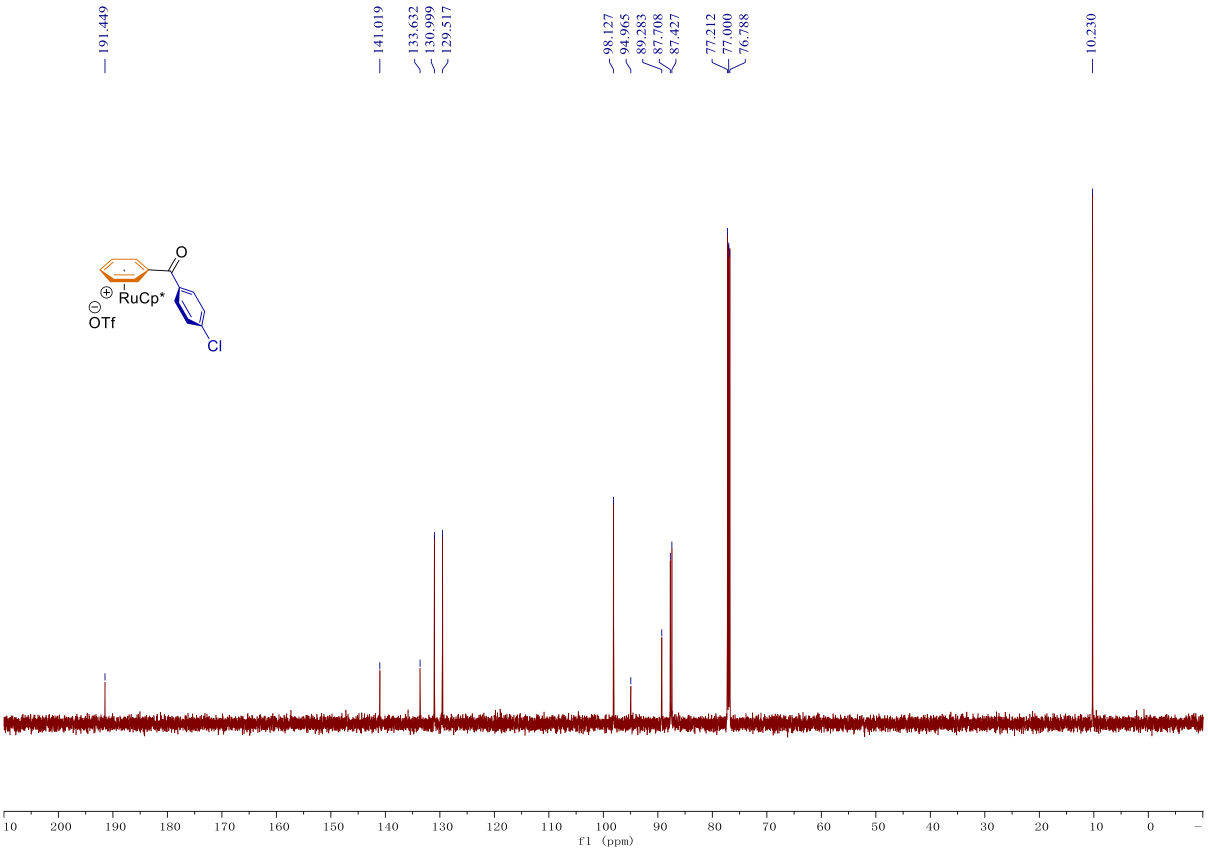

Supplementary Fig. 51 ^{13}C NMR (151 MHz, Chloroform-*d*) of [Cp*Ru(4-chlorobenzoylbenzene)]OTf (1a-Ru).

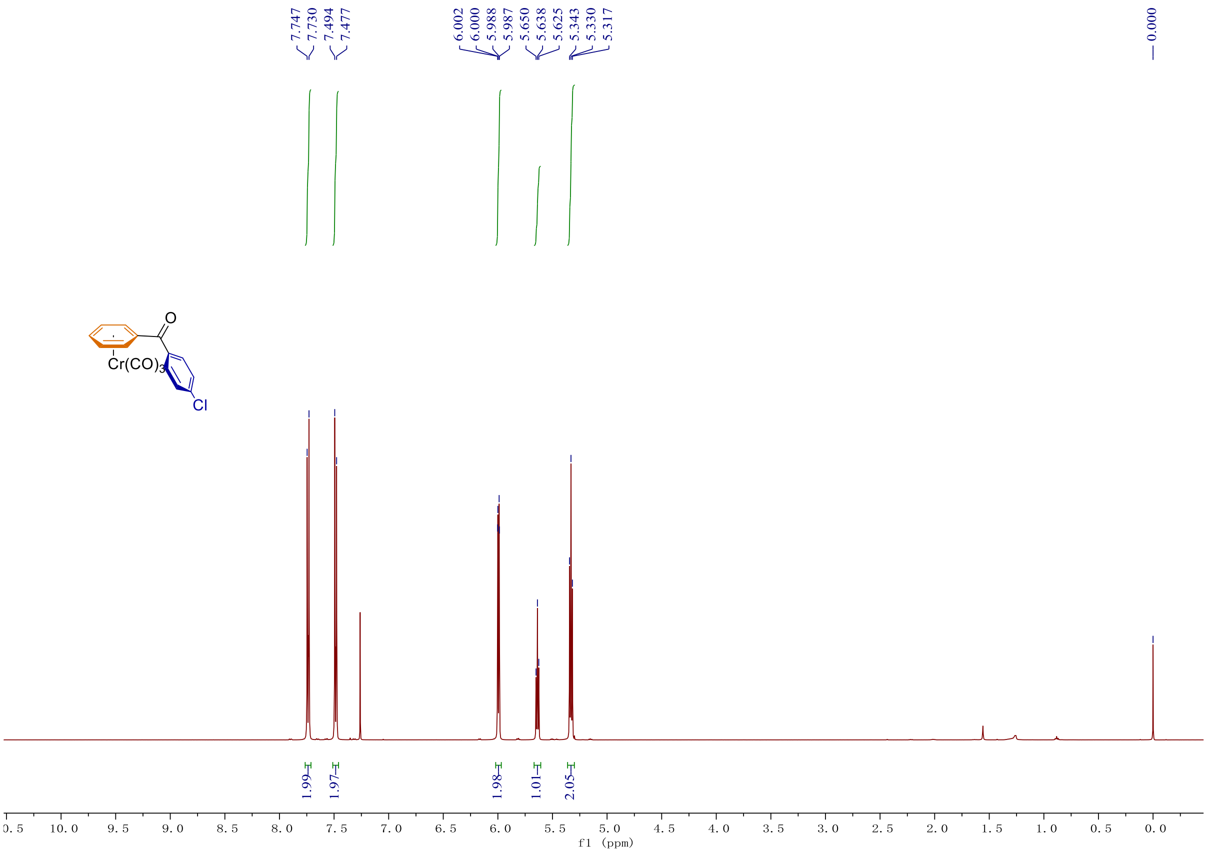

Supplementary Fig. 52 ^{1}H NMR (500 MHz, Chloroform-*d*) of 4-chlorobenzoylbenzene chromium tricarbonyl (1a-Cr).

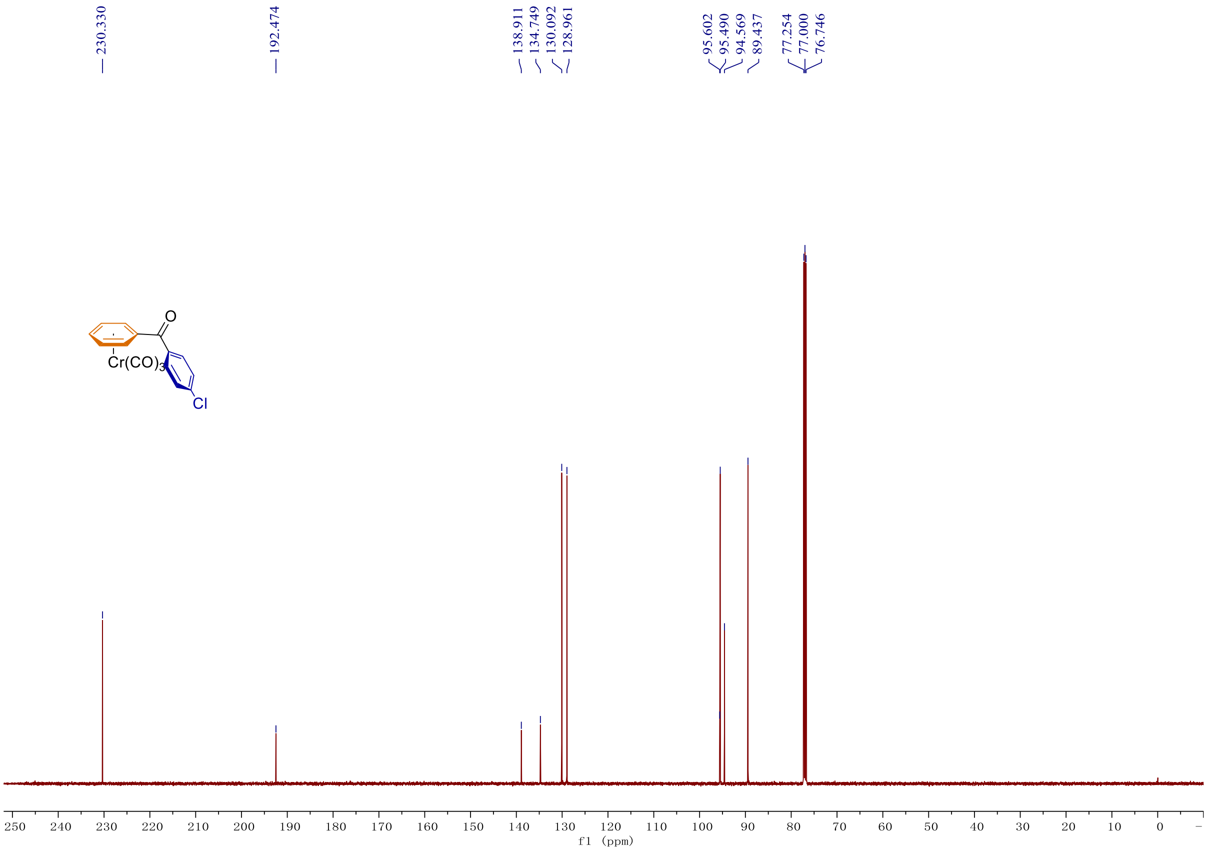

Supplementary Fig. 53 ^{13}C NMR (126 MHz, Chloroform-*d*) of 4-chlorobenzoylbenzene chromium tricarbonyl (1a-Cr).

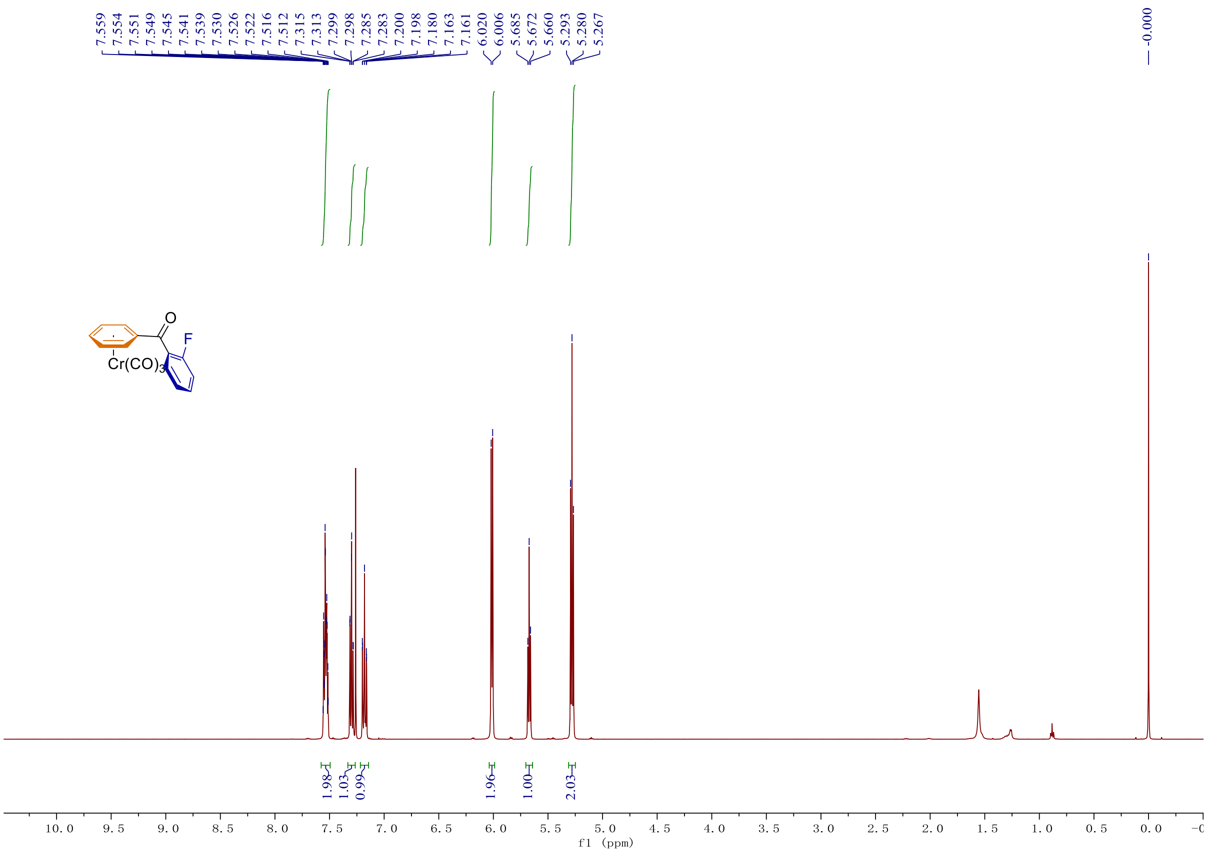

Supplementary Fig. 54 ^{1}H NMR (500 MHz, Chloroform-*d*) of 2-fluorobenzoylbenzene chromium tricarbonyl (s1-Cr).

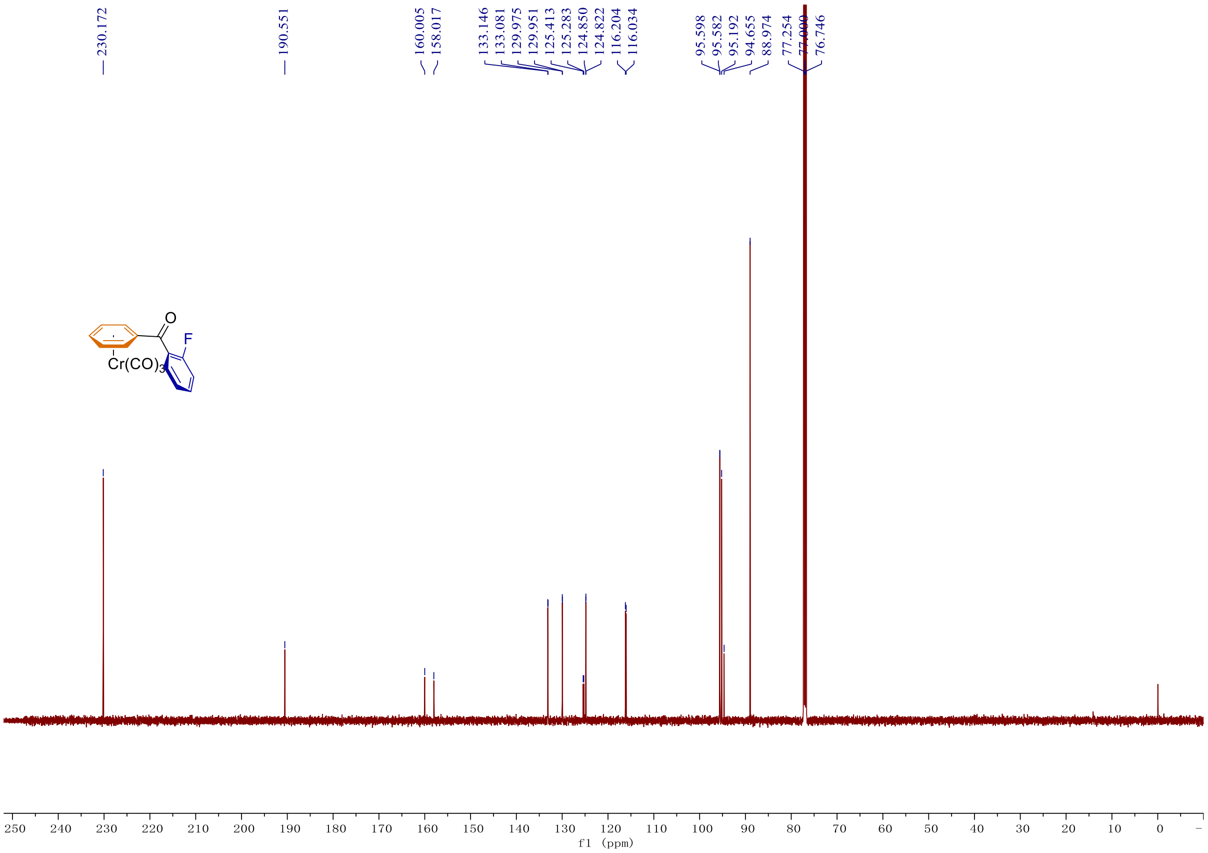

Supplementary Fig. 55 ^{13}C NMR (126 MHz, Chloroform-*d*) of 2-fluorobenzoylbenzene chromium tricarbonyl (s1-Cr).

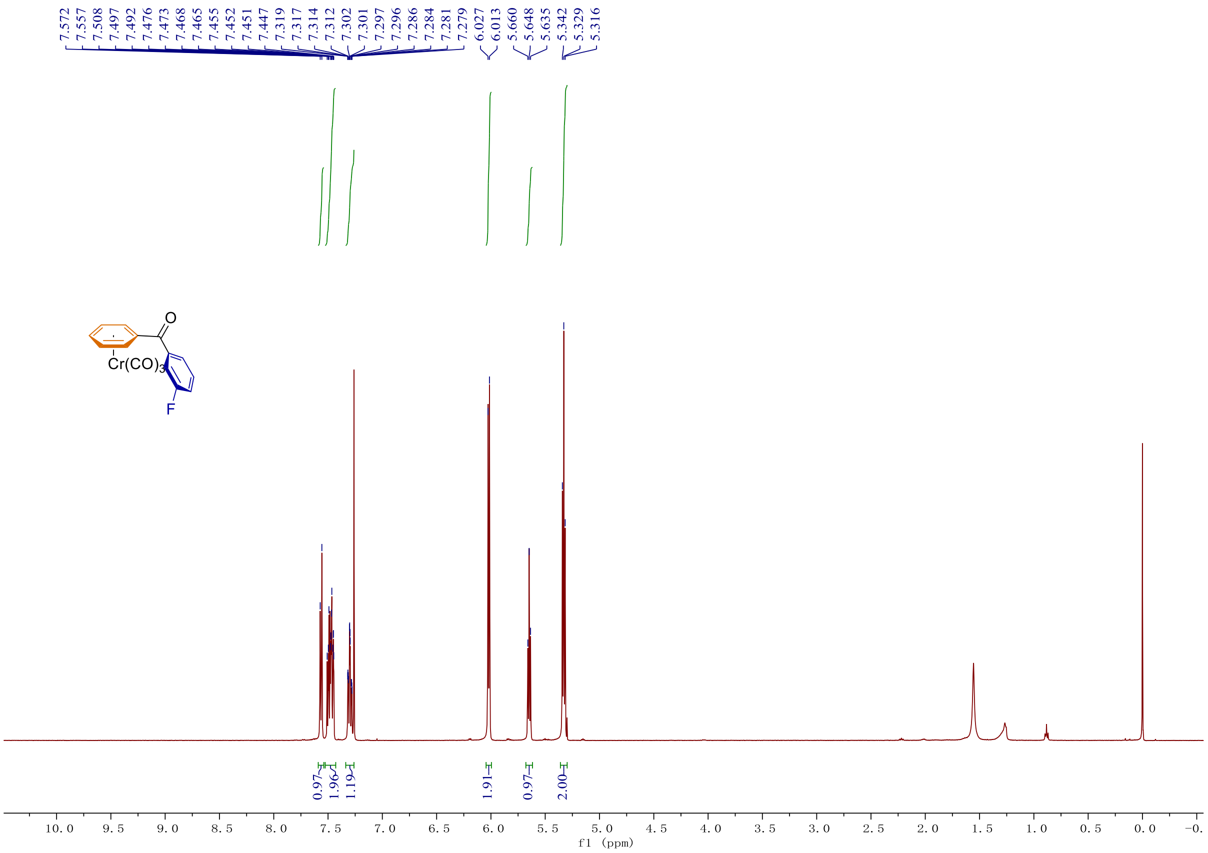

Supplementary Fig. 56 ^{1}H NMR (500 MHz, Chloroform-*d*) of 3-fluorobenzoylbenzene chromium tricarbonyl (1c-Cr).

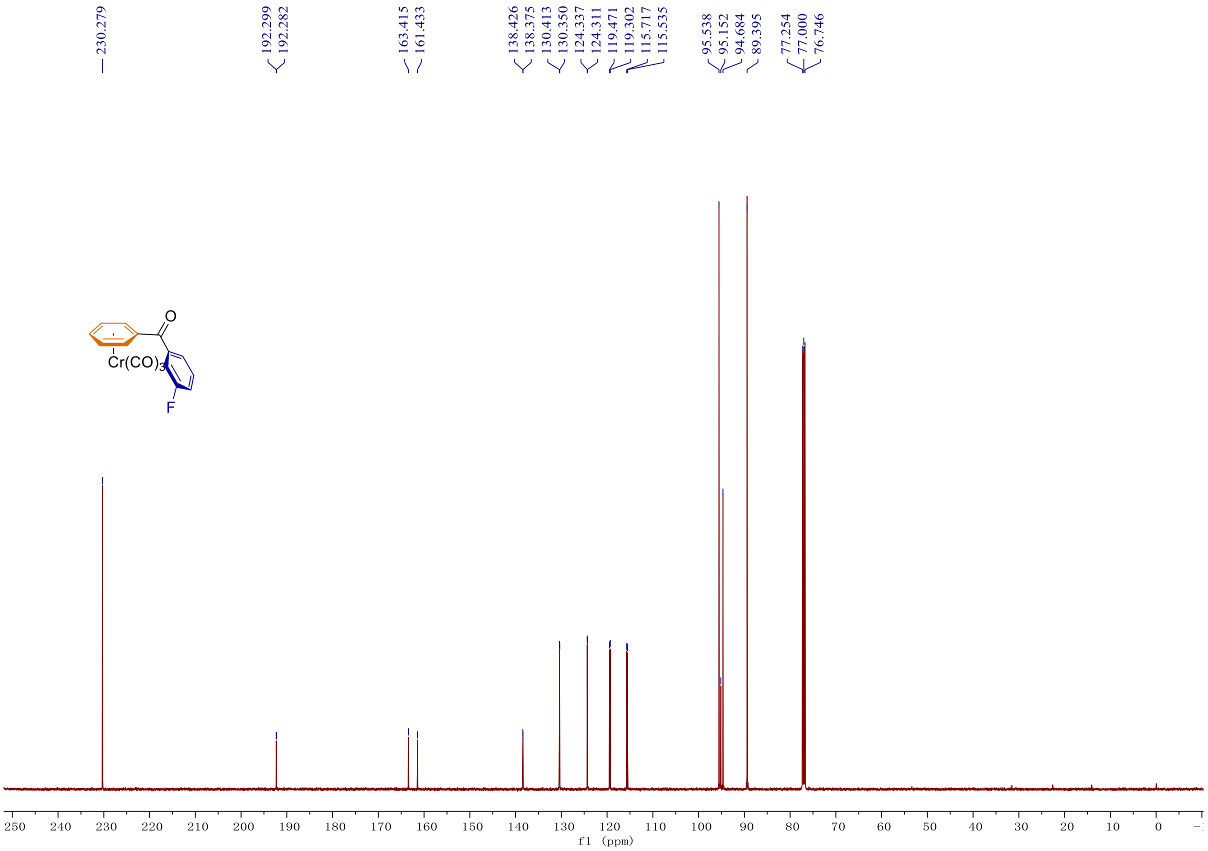

Supplementary Fig. 57 ^{13}C NMR (126 MHz, Chloroform-*d*) of 3-fluorobenzoylbenzene chromium tricarbonyl (1c-Cr).

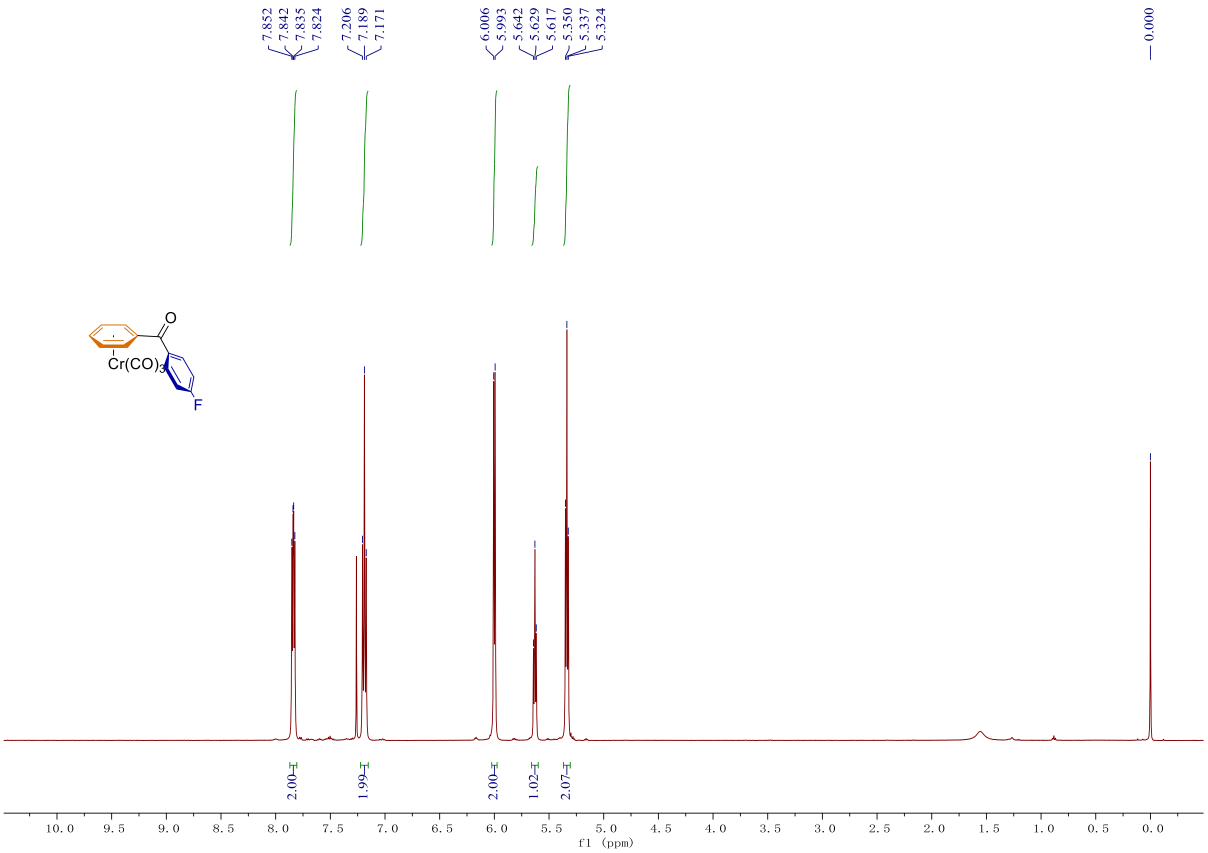

Supplementary Fig. 58 ^{1}H NMR (500 MHz, Chloroform-*d*) of 4-fluorobenzoylbenzene chromium tricarbonyl (1d-Cr).

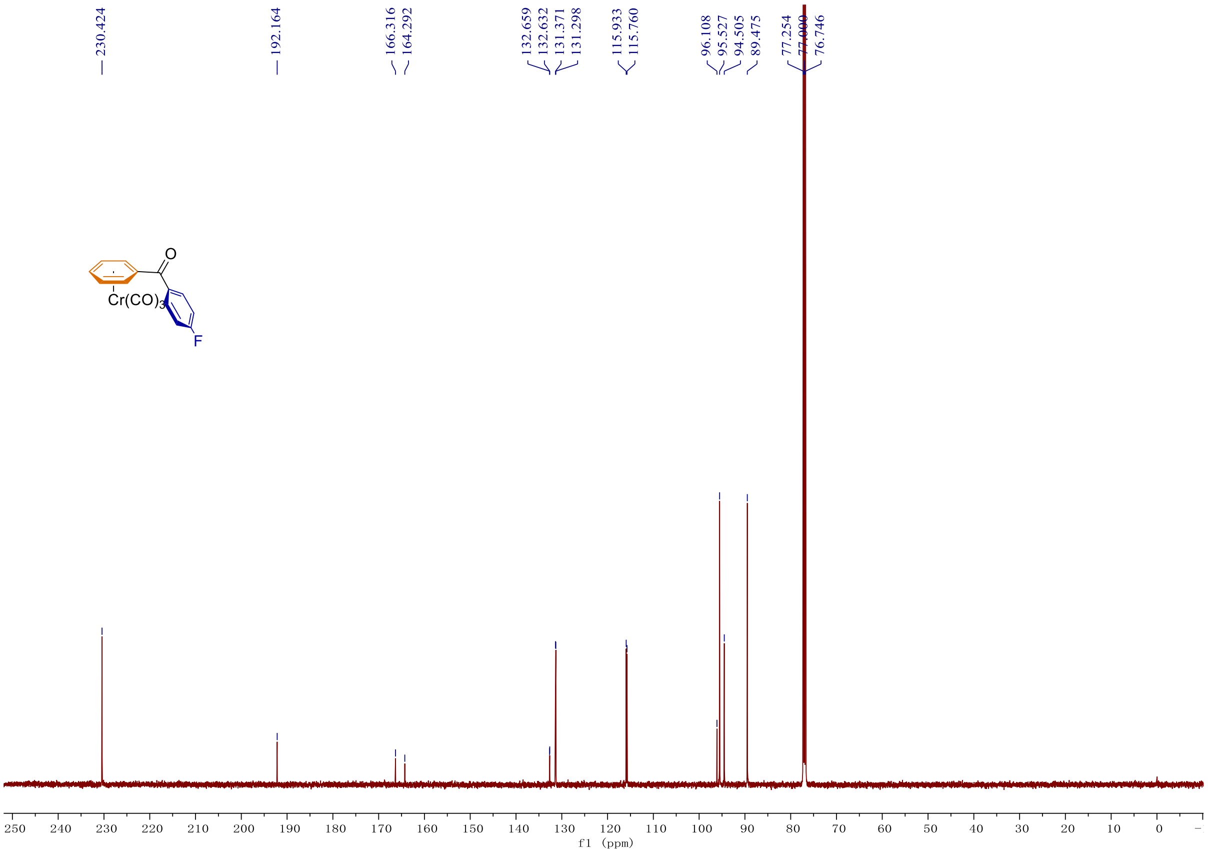

Supplementary Fig. 59 ^{13}C NMR (126 MHz, Chloroform-*d*) of 4-fluorobenzoylbenzene chromium tricarbonyl (1d-Cr).

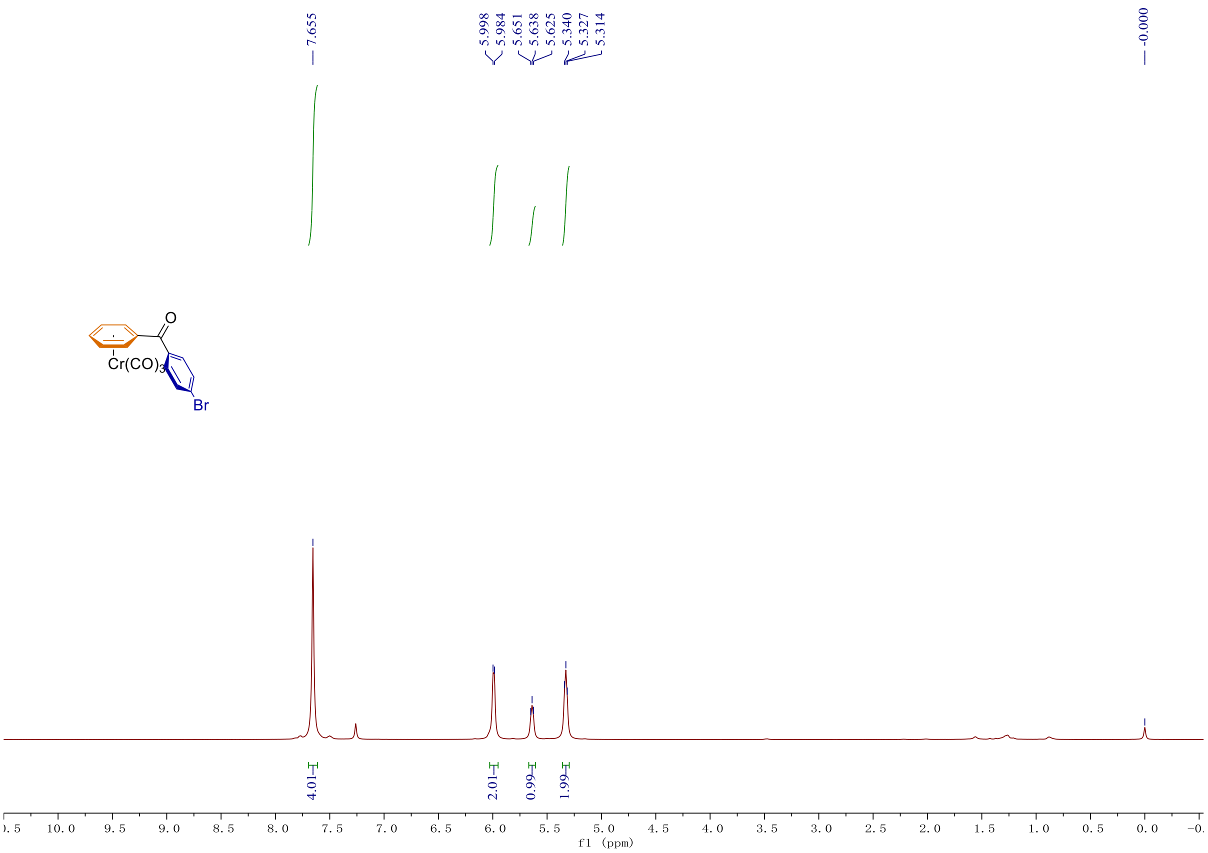

Supplementary Fig. 60 ^{1}H NMR (500 MHz, Chloroform-*d*) of 4-bromobenzoylbenzene chromium tricarbonyl (1e-Cr).

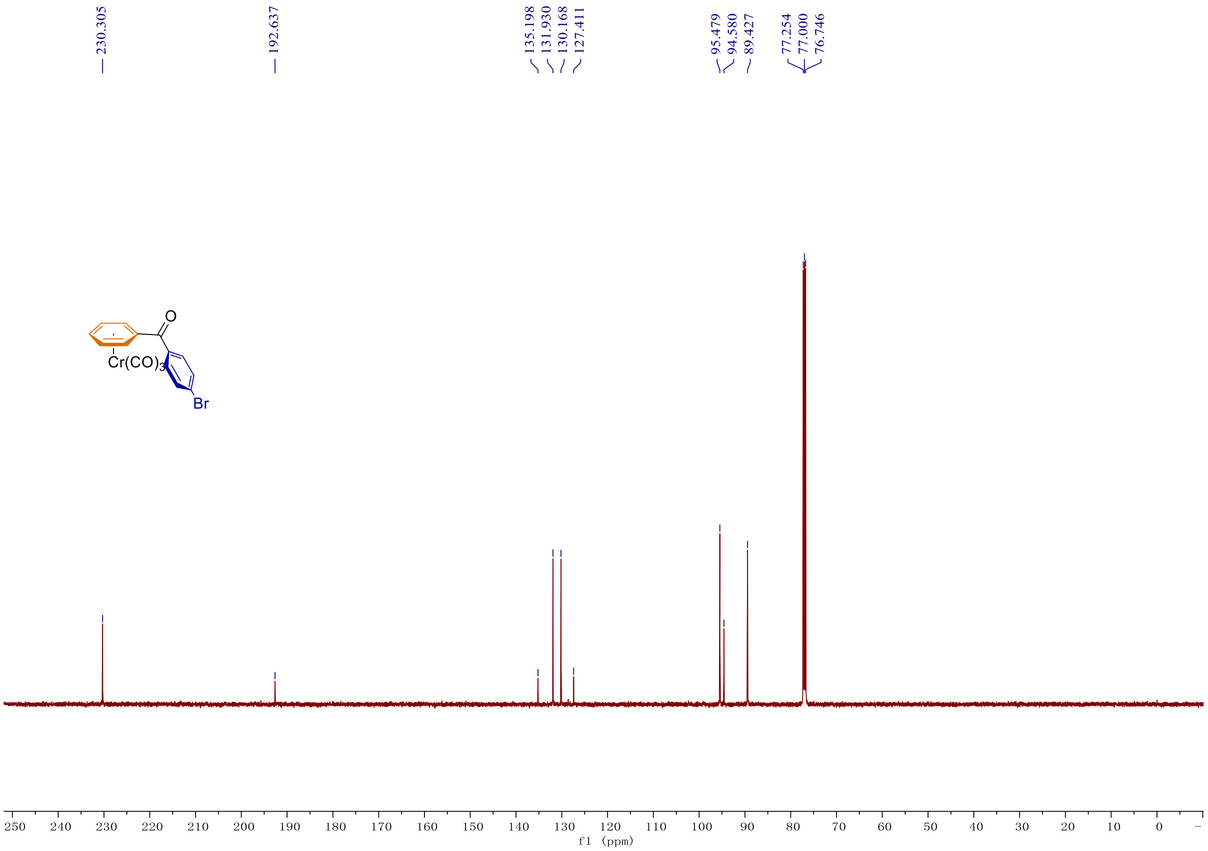

Supplementary Fig. 61 ^{13}C NMR (126 MHz, Chloroform-*d*) of 4-bromobenzoylbenzene chromium tricarbonyl (1e-Cr).

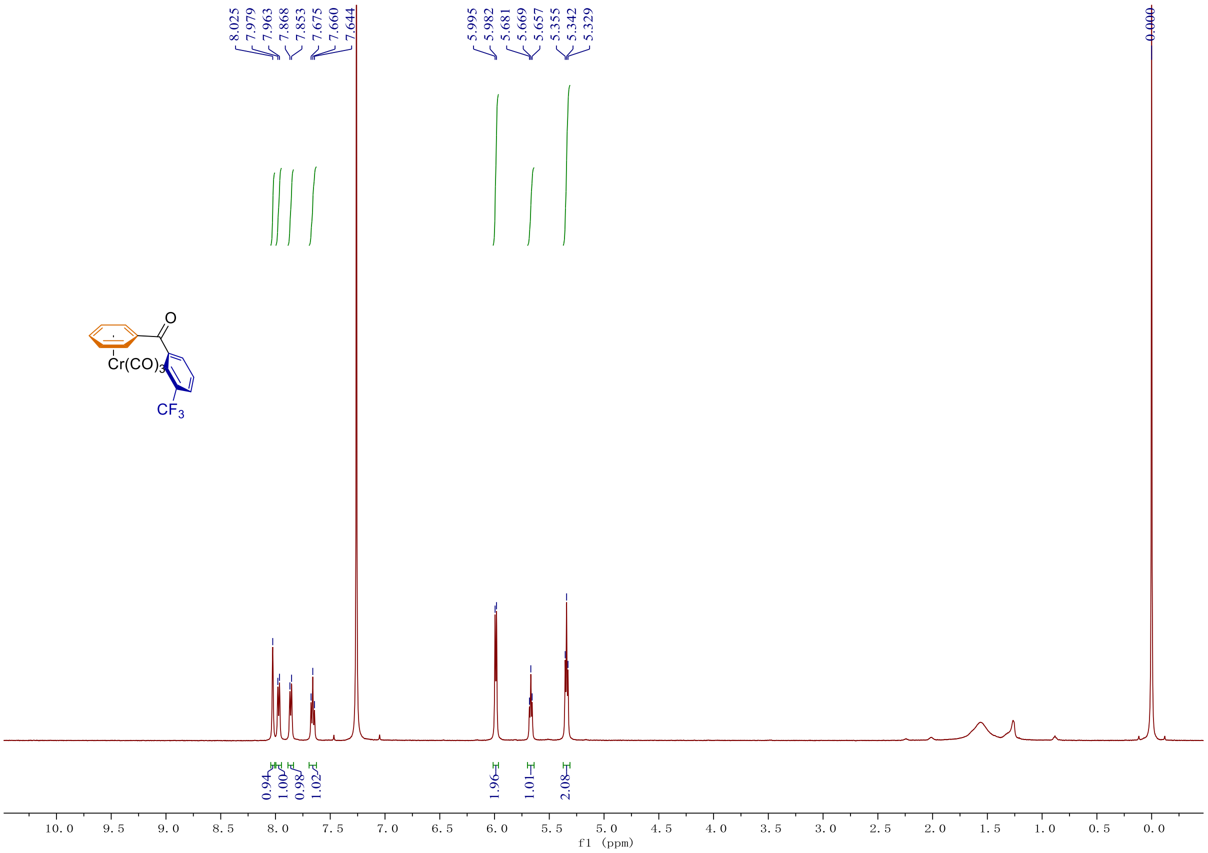

Supplementary Fig. 62 ^{1}H NMR (500 MHz, Chloroform-*d*) of 3-(trifluoromethyl)benzoylbenzene chromium tricarbonyl (1y-Cr).

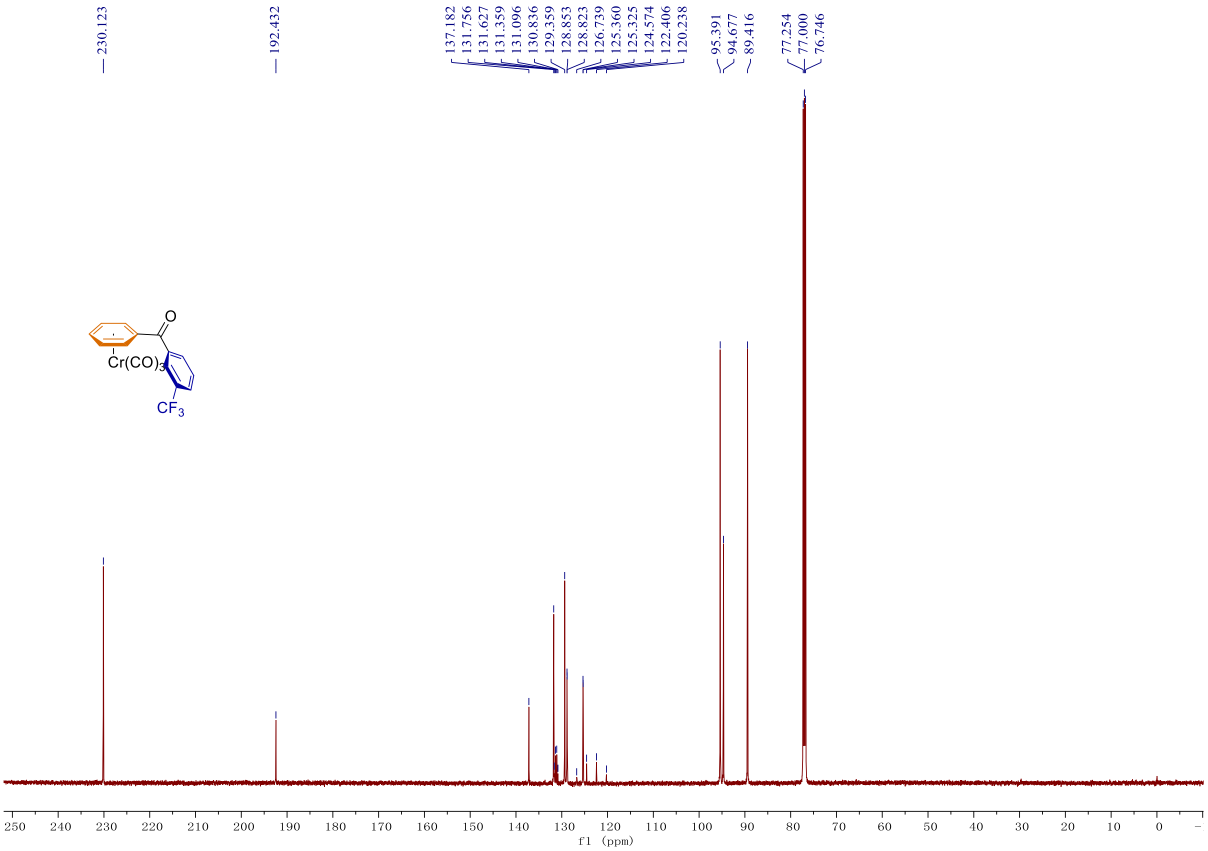

Supplementary Fig. 63 ^{13}C NMR (126 MHz, Chloroform-*d*) of 3-(trifluoromethyl)benzoylbenzene chromium tricarbonyl (1y-Cr).

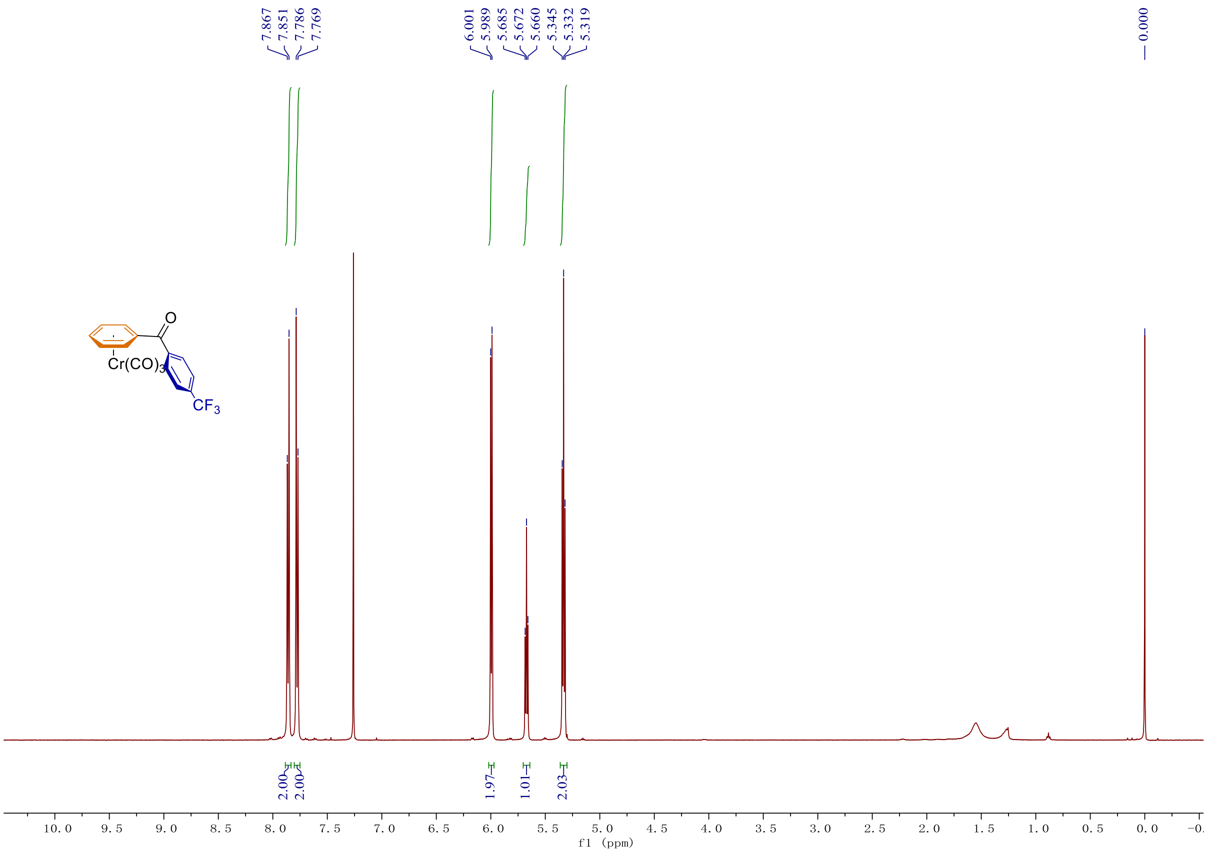

Supplementary Fig. 64 ^{1}H NMR (500 MHz, Chloroform-*d*) of 4-(trifluoromethyl)benzoylbenzene chromium tricarbonyl (1f-Cr).

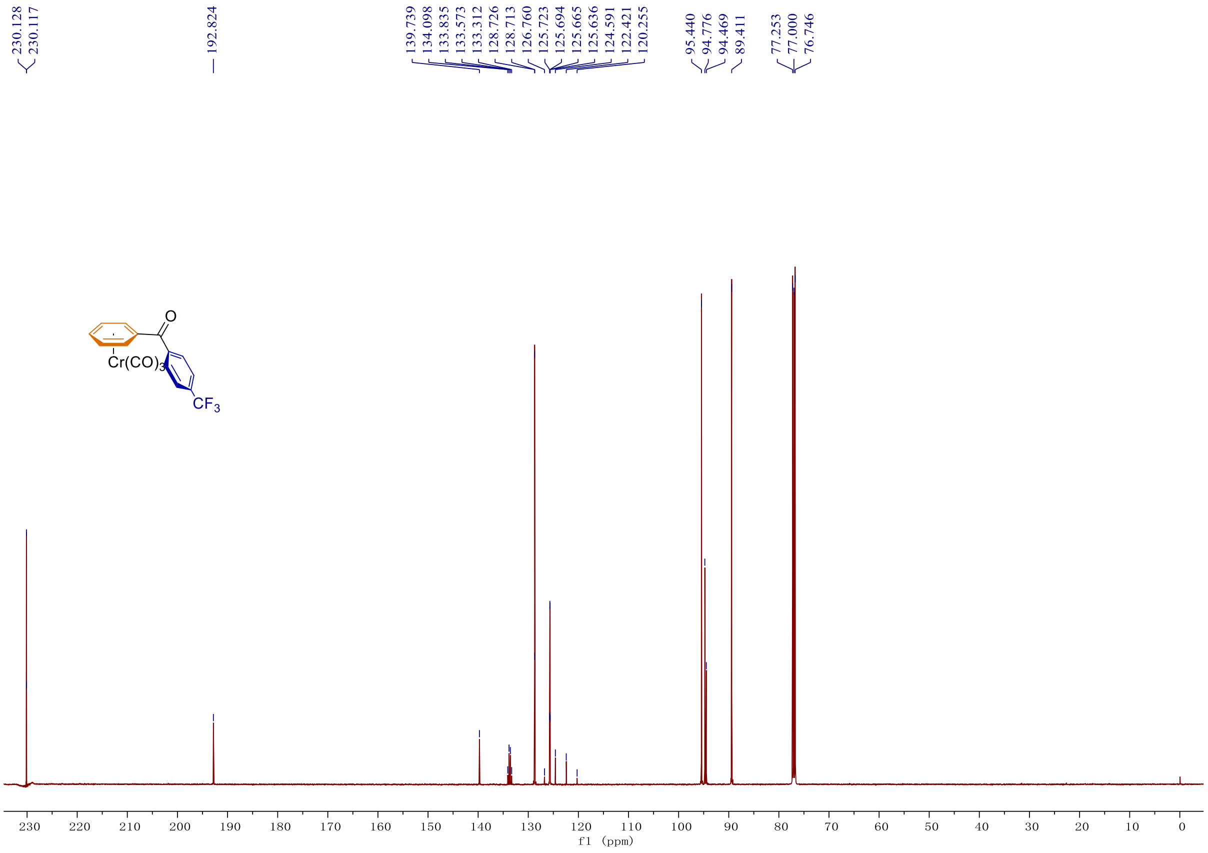

Supplementary Fig. 65 ^{13}C NMR (126 MHz, Chloroform-*d*) of 4-(trifluoromethyl)benzoylbenzene chromium tricarbonyl (1f-Cr).

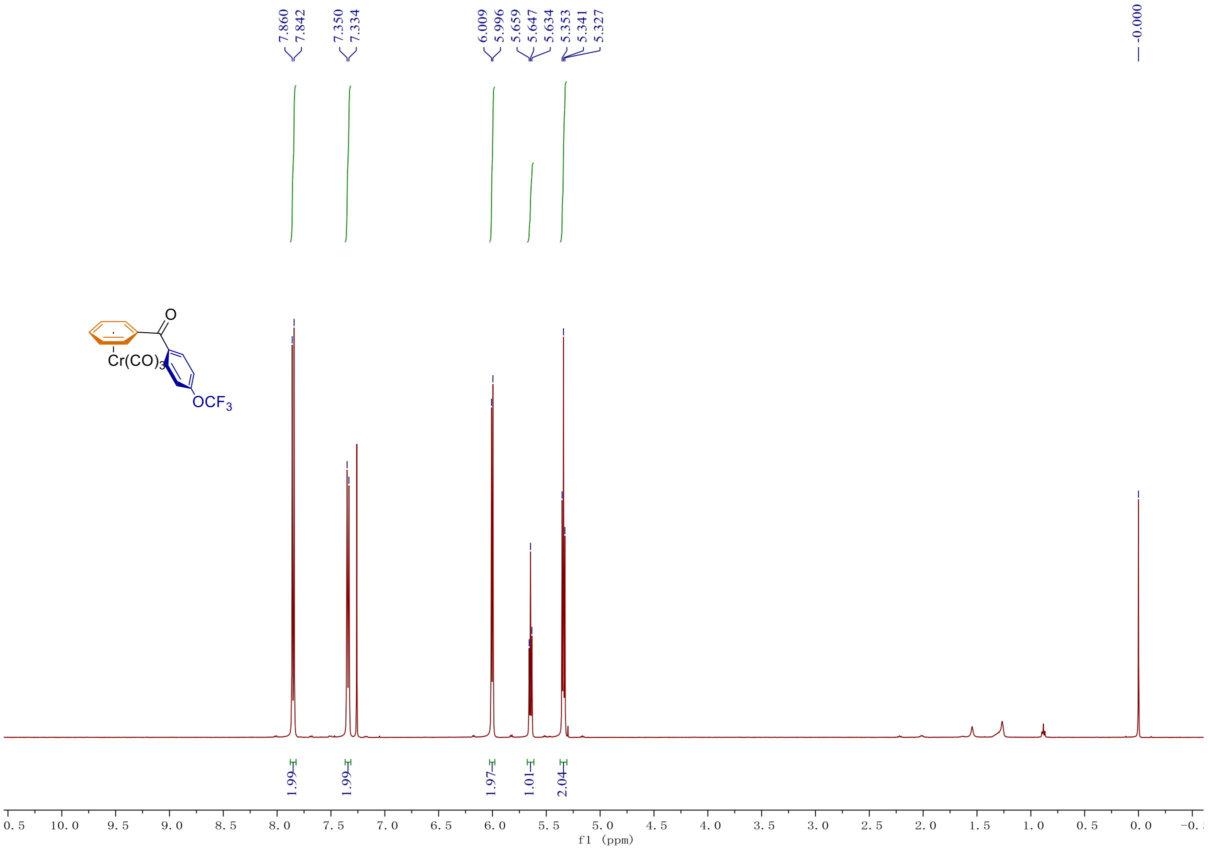

Supplementary Fig. 66 ^{1}H NMR (500 MHz, Chloroform-*d*) of 4-(trifluoromethyloxy)benzoylbenzene chromium tricarbonyl (1g-Cr).

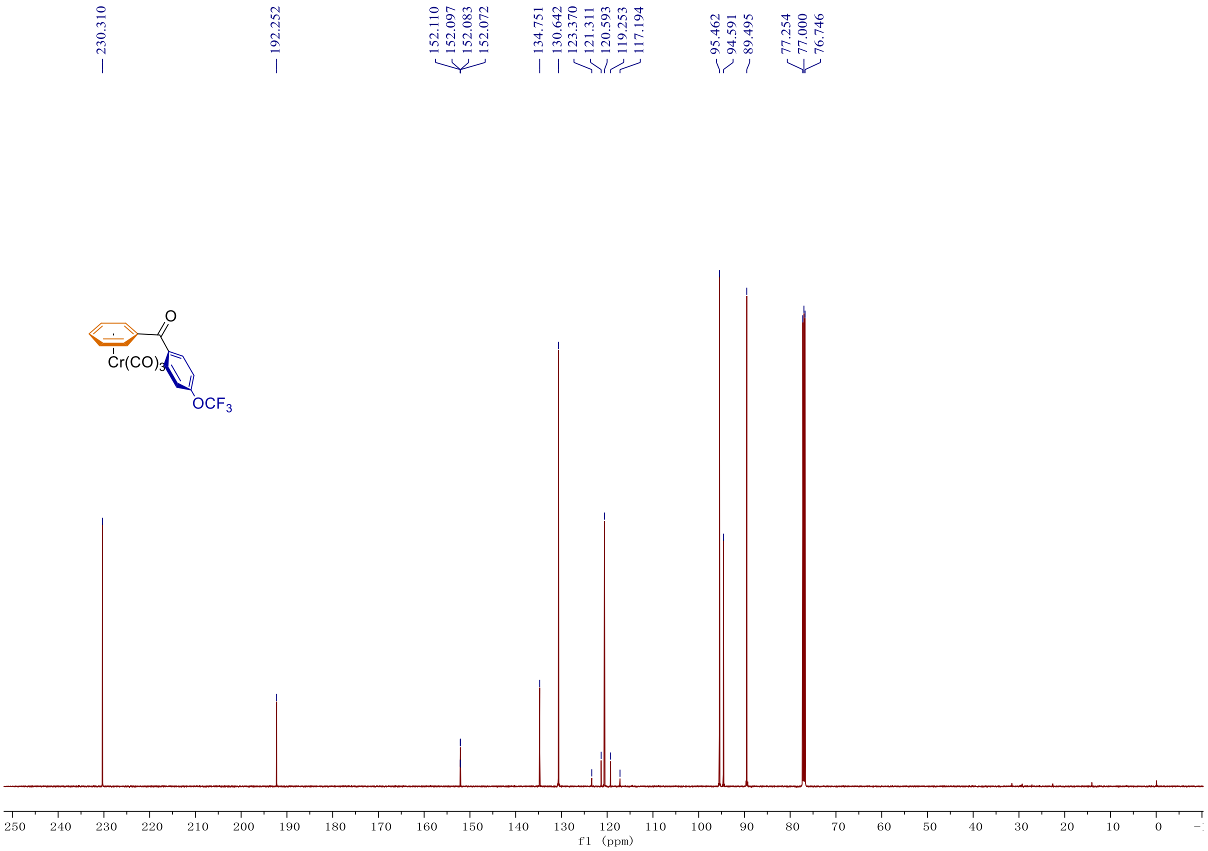

Supplementary Fig. 67 ^{13}C NMR (126 MHz, Chloroform-*d*) of 4-(trifluoromethyloxy)benzoylbenzene chromium tricarbonyl (1g-Cr).

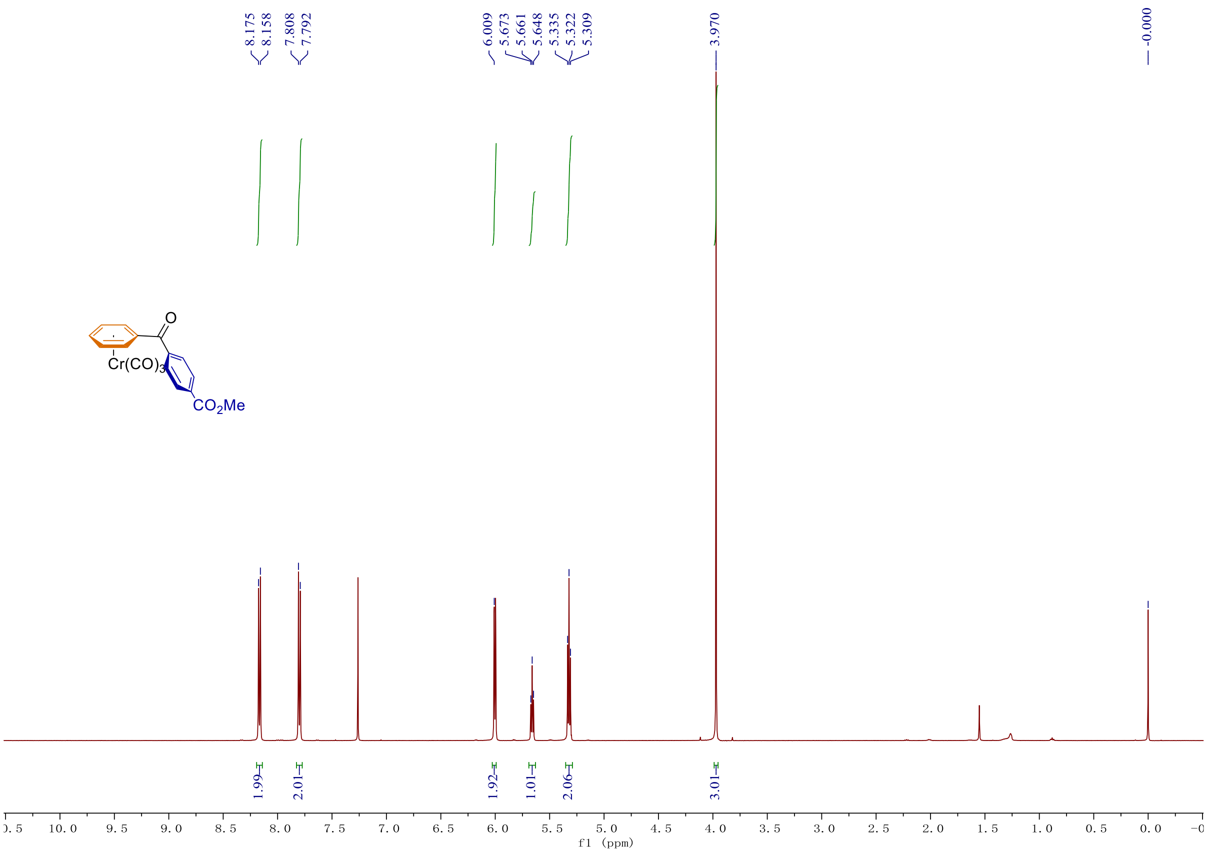

Supplementary Fig. 68 ^{1}H NMR (500 MHz, Chloroform-*d*) of 4-(methoxycarbonyl)benzoylbenzene chromium tricarbonyl (1h-Cr).

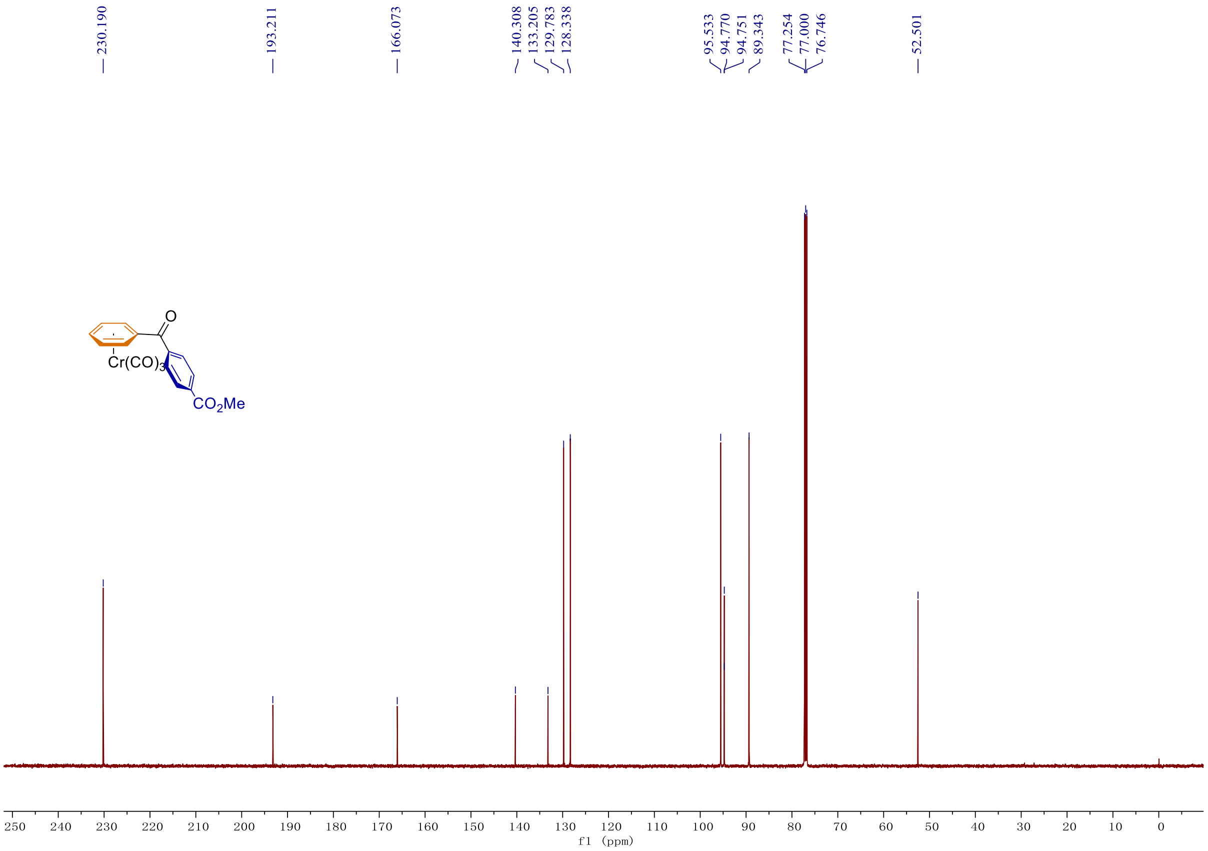

Supplementary Fig. 69 ^{13}C NMR (126 MHz, Chloroform-*d*) of 4-(methoxycarbonyl)benzoylbenzene chromium tricarbonyl (1h-Cr).

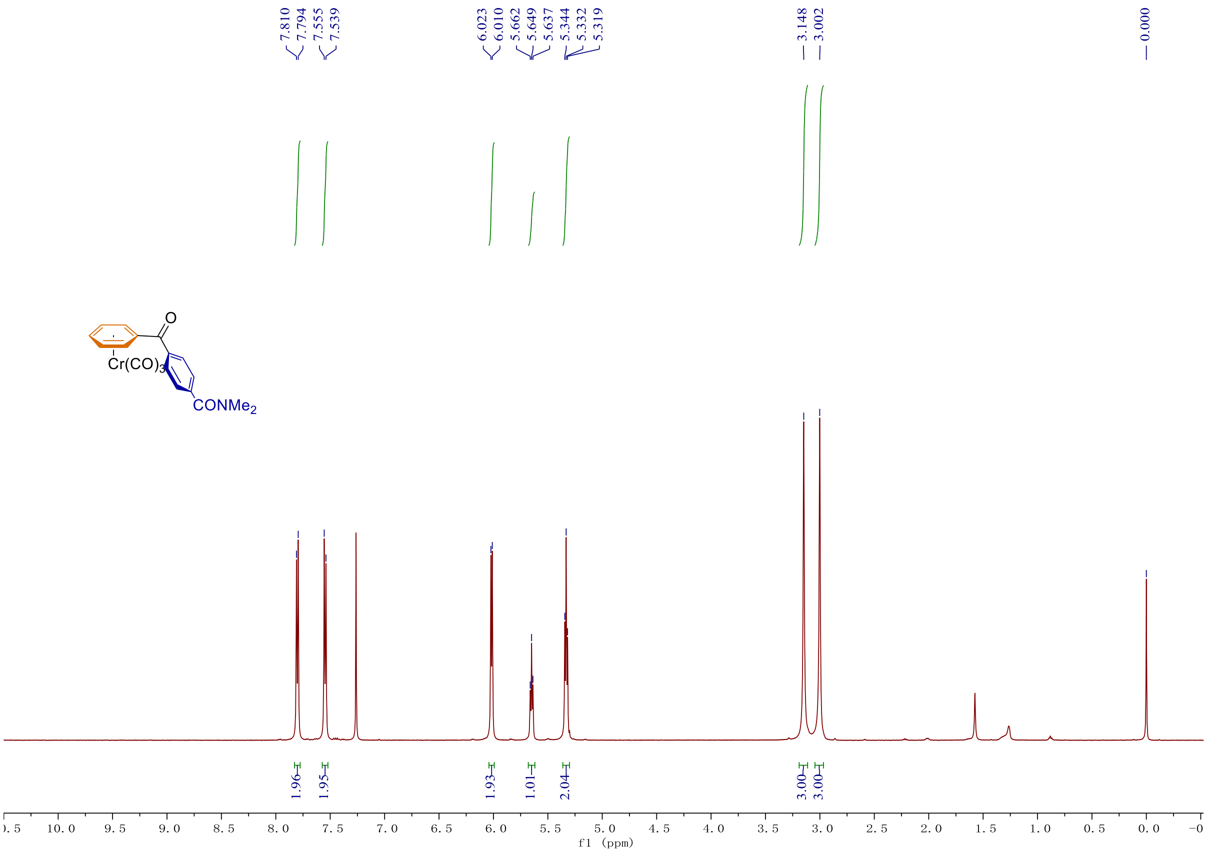

Supplementary Fig. 70 ^{1}H NMR (500 MHz, Chloroform-*d*) of 4-(dimethylcarbamoyl)benzoylbenzene chromium tricarbonyl (1i-Cr).

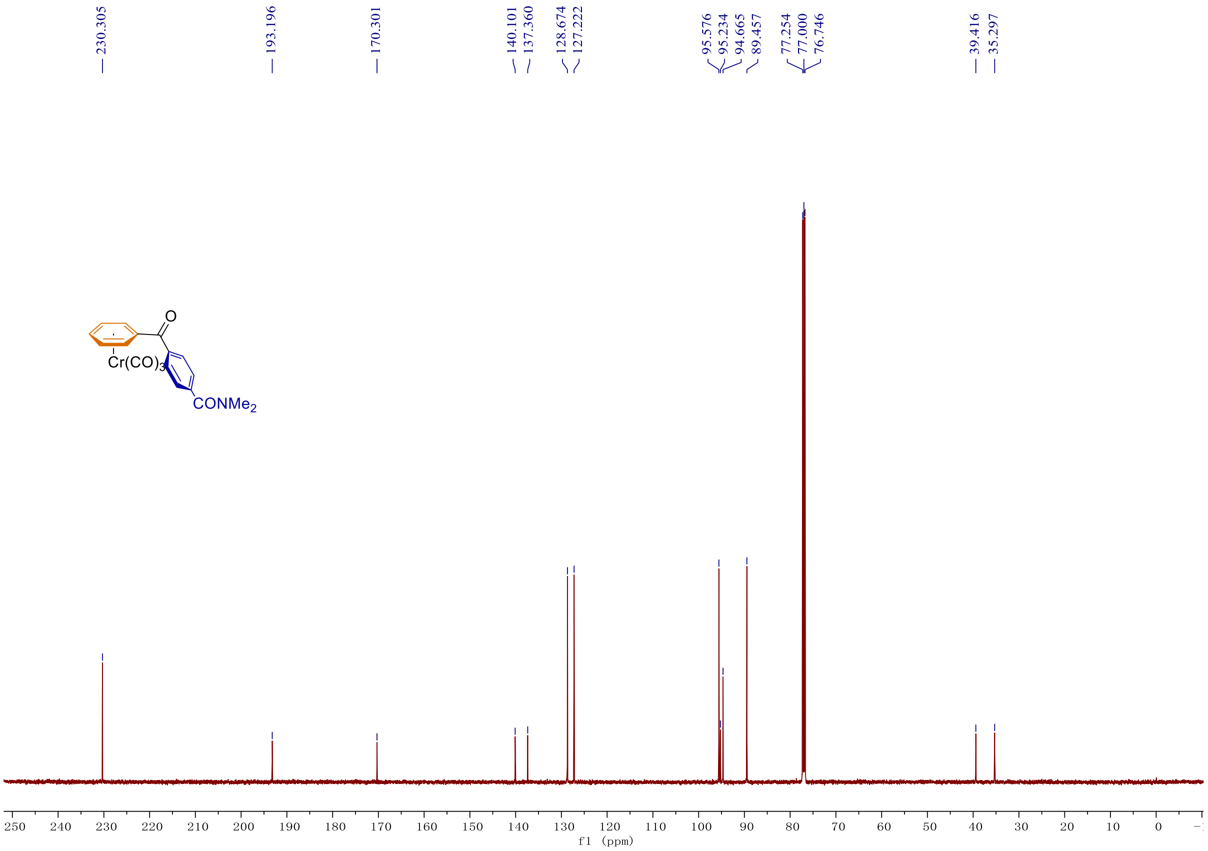

Supplementary Fig. 71 ^{13}C NMR (126 MHz, Chloroform-*d*) of 4-(dimethylcarbamoyl)benzoylbenzene chromium tricarbonyl (1i-Cr).

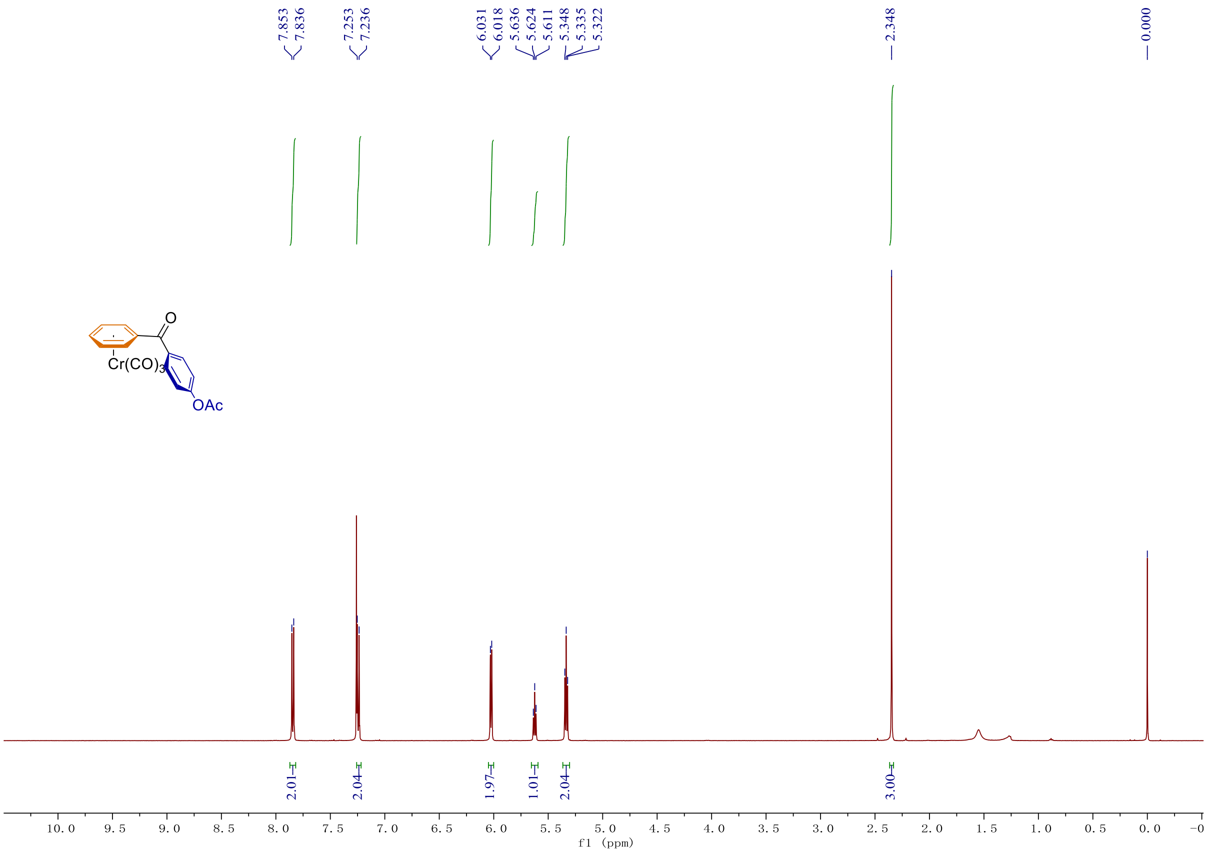

Supplementary Fig. 72 ^{1}H NMR (500 MHz, Chloroform-*d*) of 4-(acetoxy)benzoylbenzene chromium tricarbonyl (s2-Cr).

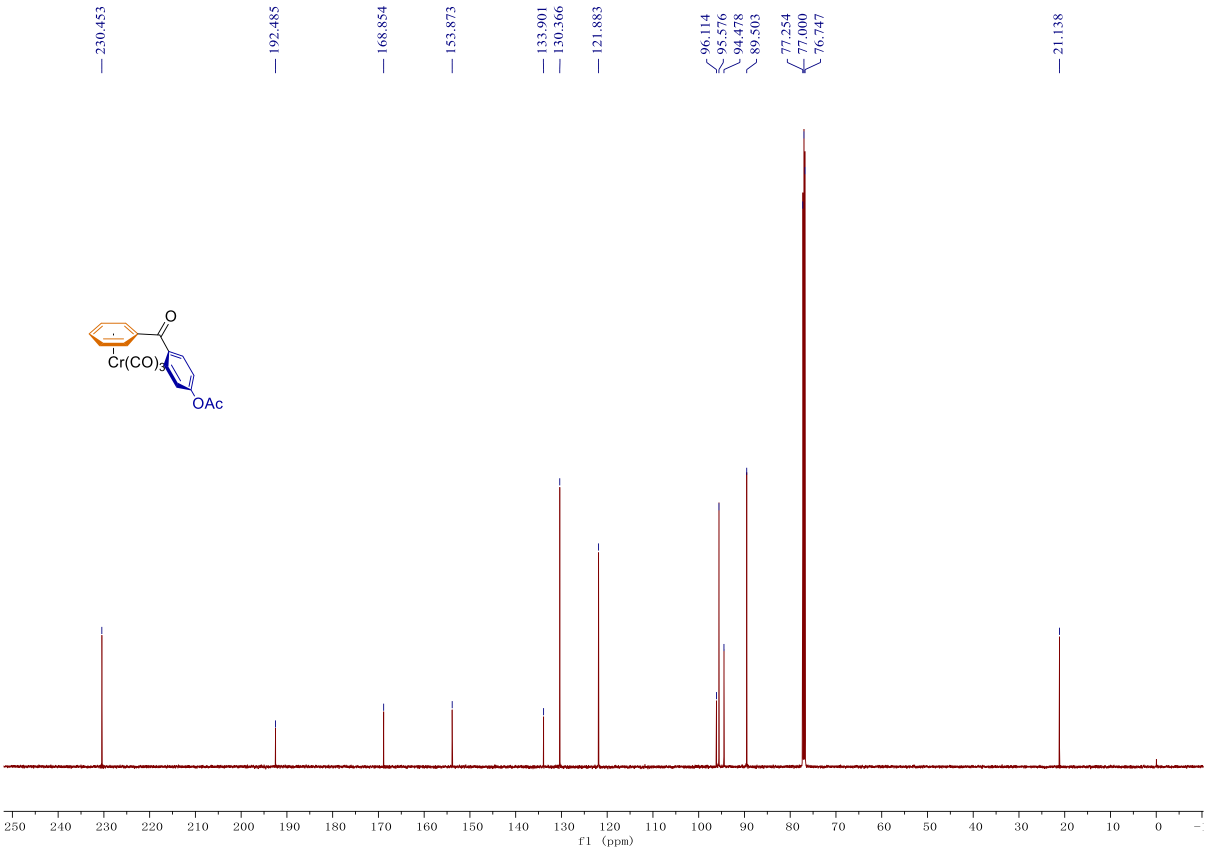

Supplementary Fig. 73 ^{13}C NMR (126 MHz, Chloroform-*d*) of 4-(acetoxy)benzoylbenzene chromium tricarbonyl (s2-Cr).

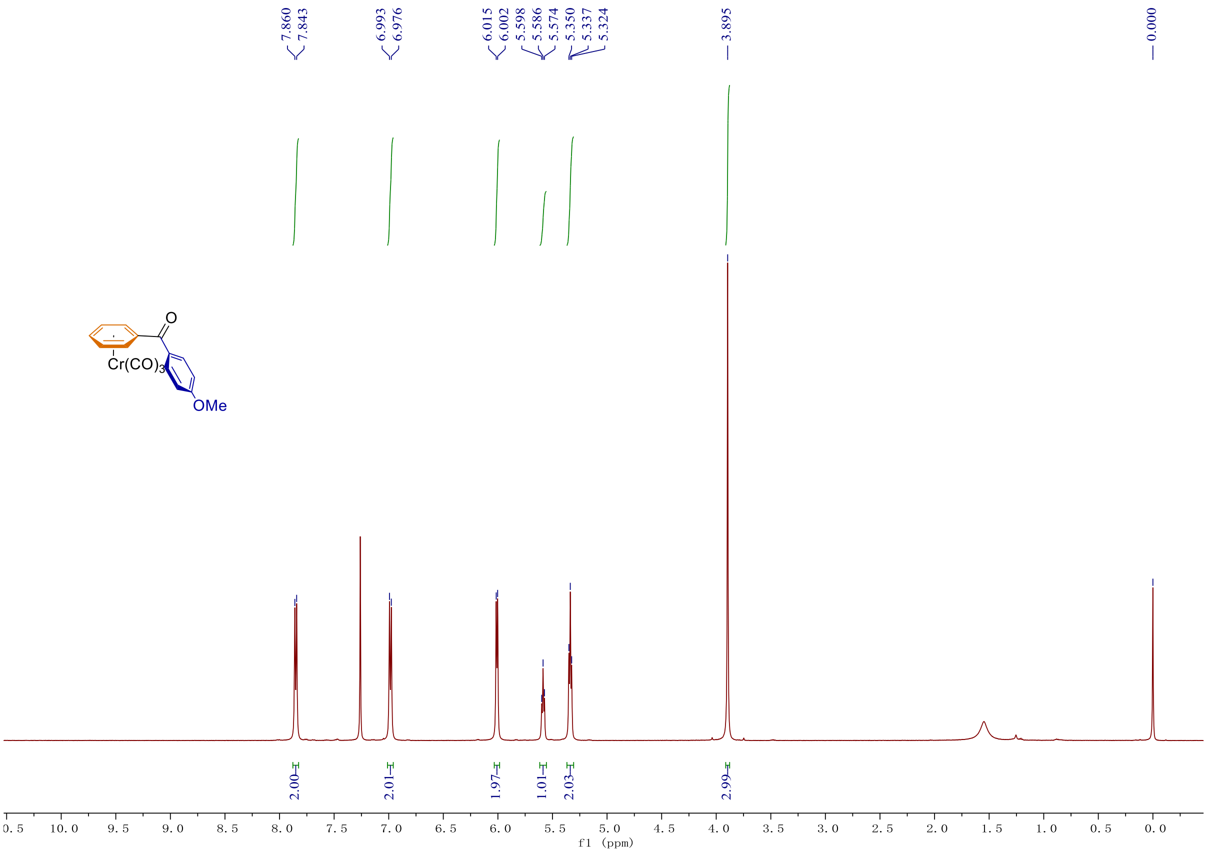

Supplementary Fig. 74 ^{1}H NMR (500 MHz, Chloroform-*d*) of 4-(methoxy)benzoylbenzene chromium tricarbonyl (1b-Cr).

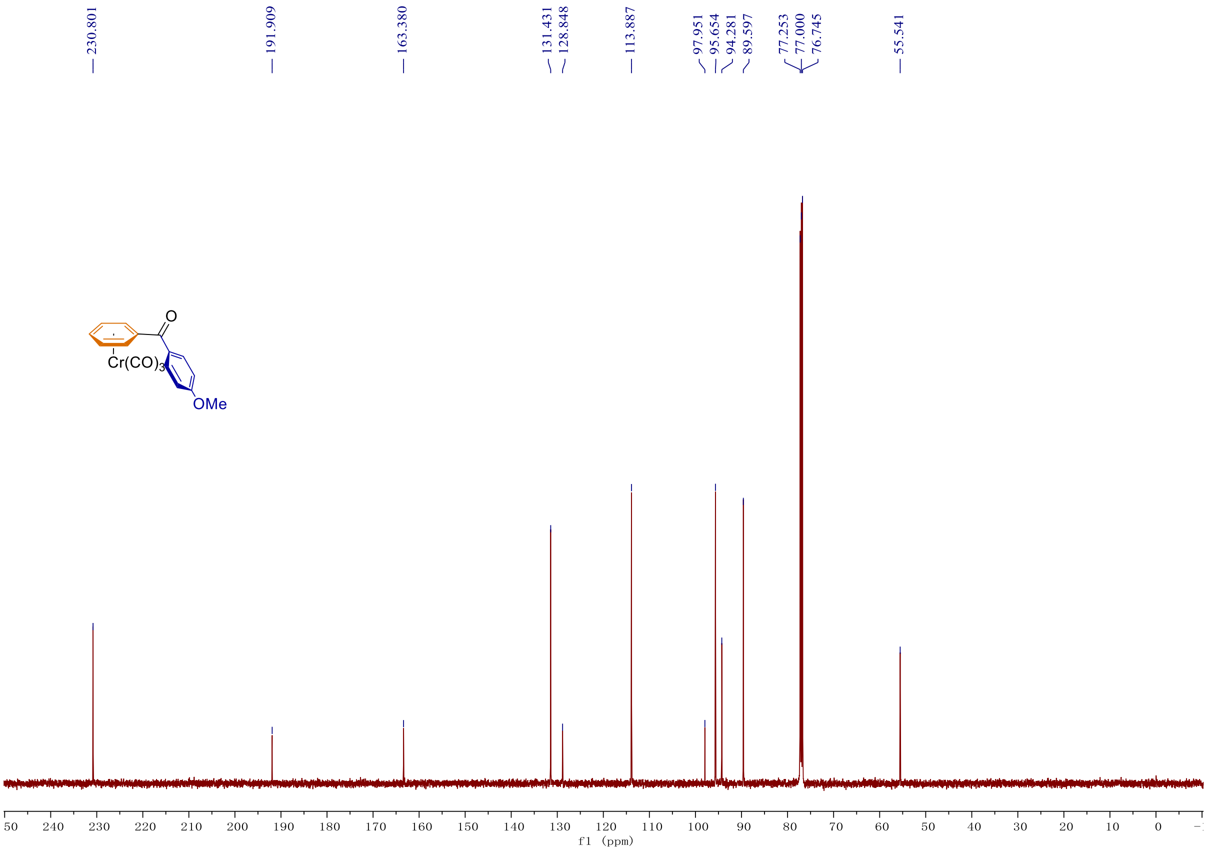

Supplementary Fig. 75 ^{13}C NMR (126 MHz, Chloroform-*d*) of 4-(methoxy)benzoylbenzene chromium tricarbonyl (1b-Cr).

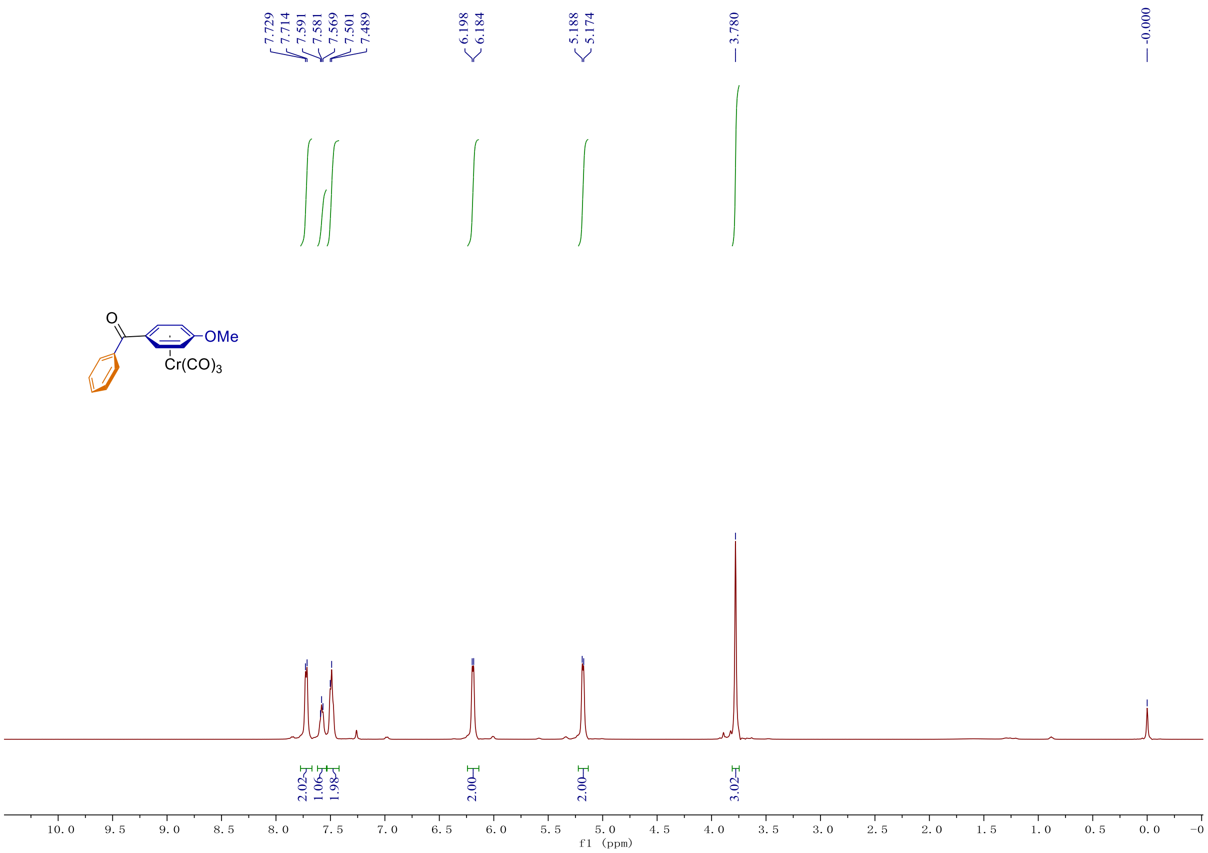

Supplementary Fig. 76 ^{1}H NMR (500 MHz, Chloroform-*d*) of 4-benzoylanisole chromium tricarbonyl (1b-Cr').

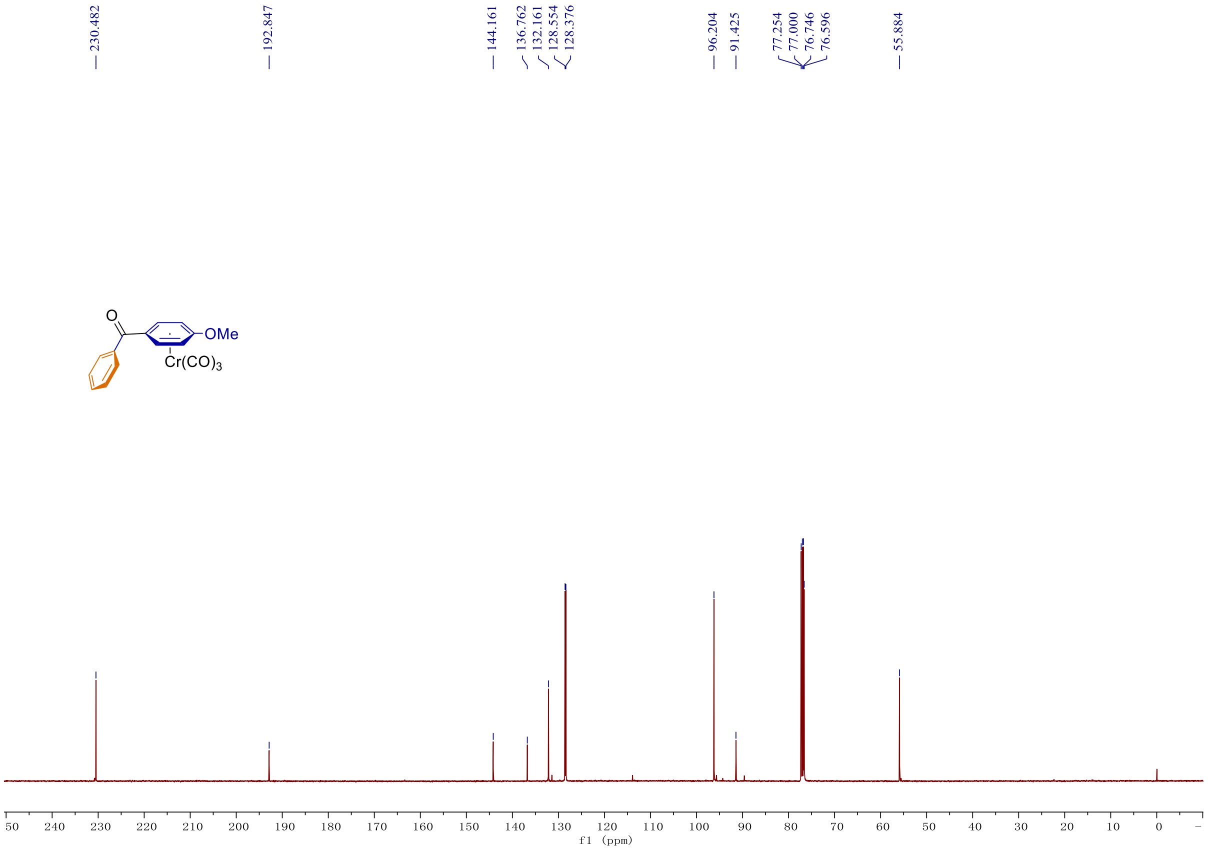

Supplementary Fig. 77 ^{13}C NMR (126 MHz, Chloroform-*d*) of 4-benzoylanisole chromium tricarbonyl (1b-Cr').

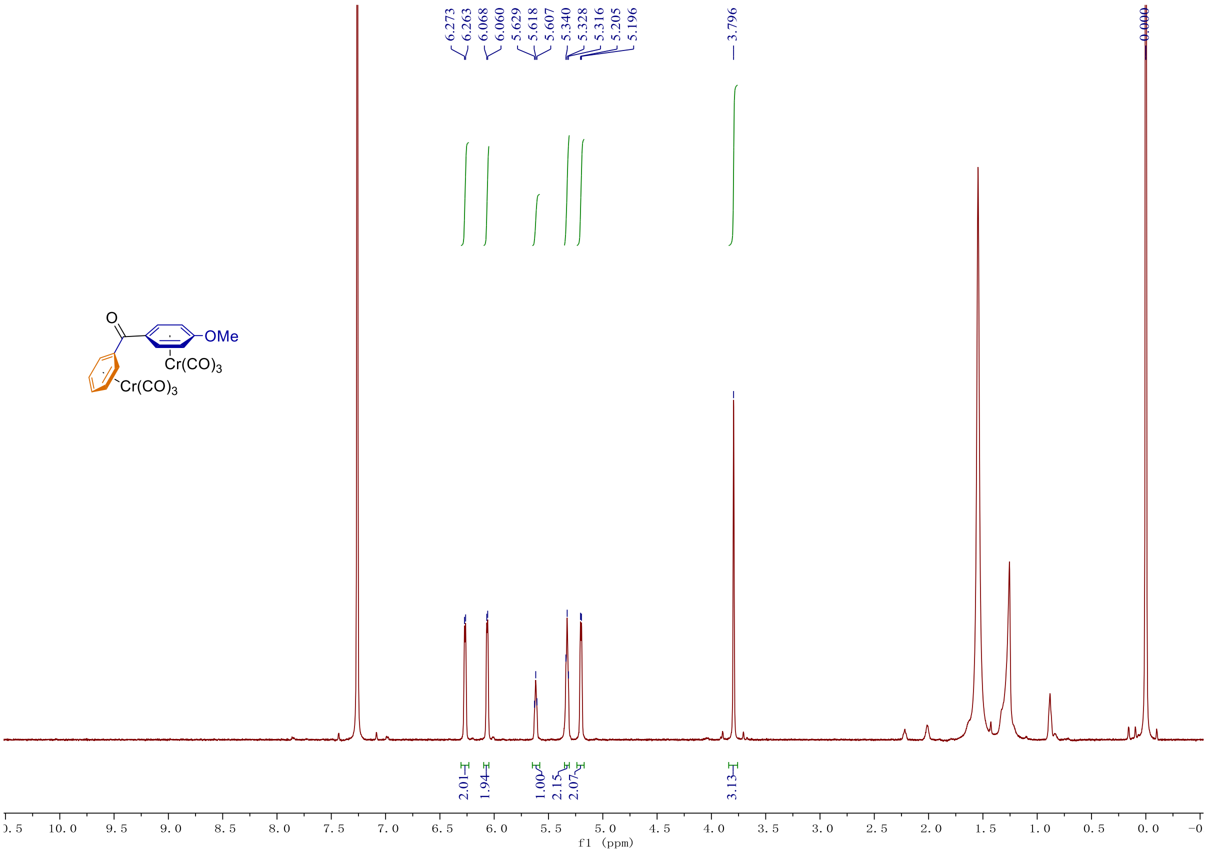

Supplementary Fig. 78 ^{1}H NMR (600 MHz, Chloroform-*d*) of (4-methoxyphenyl chromium tricarbonyl)(phenyl chromium tricarbonyl)methanone (1b-diCr).

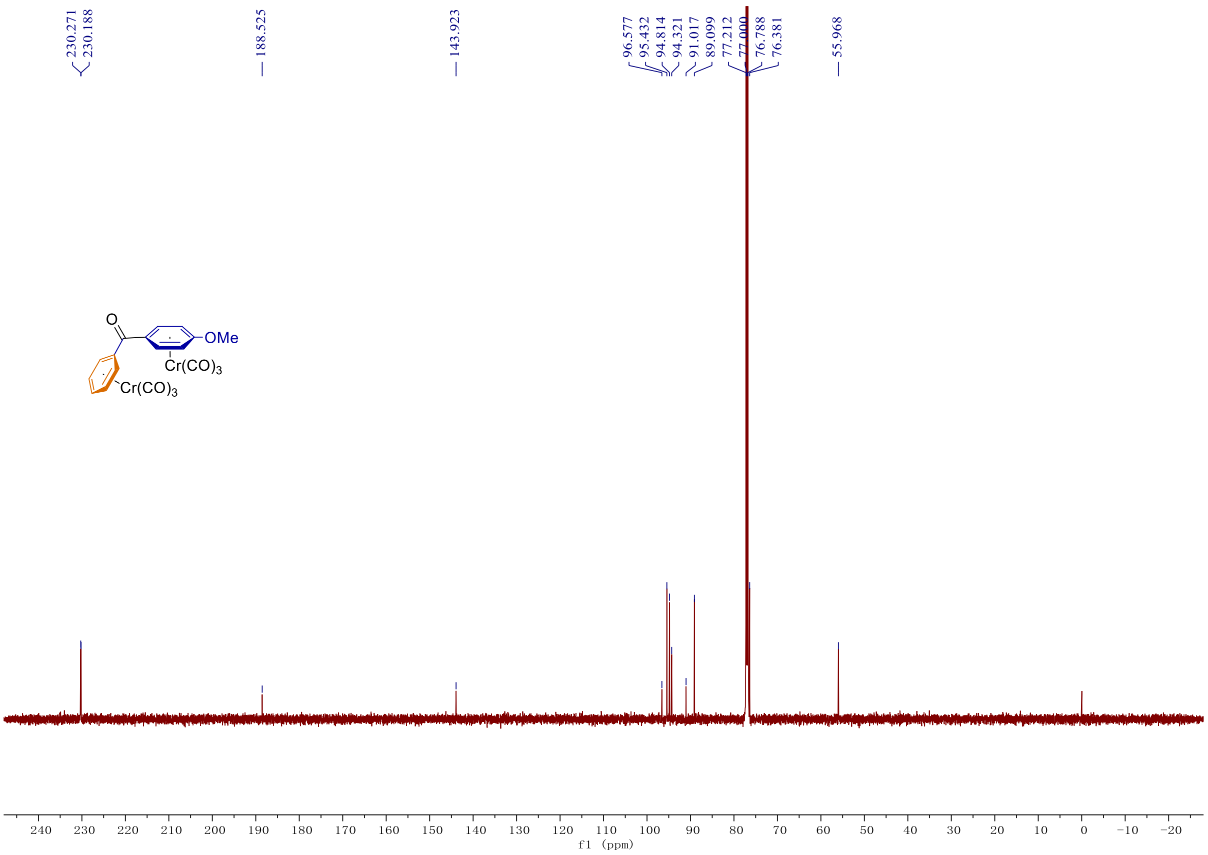

Supplementary Fig. 79 ^{13}C NMR (151 MHz, Chloroform-*d*) of (4-methoxyphenyl chromium tricarbonyl)(phenyl chromium tricarbonyl)methanone (1b-diCr).

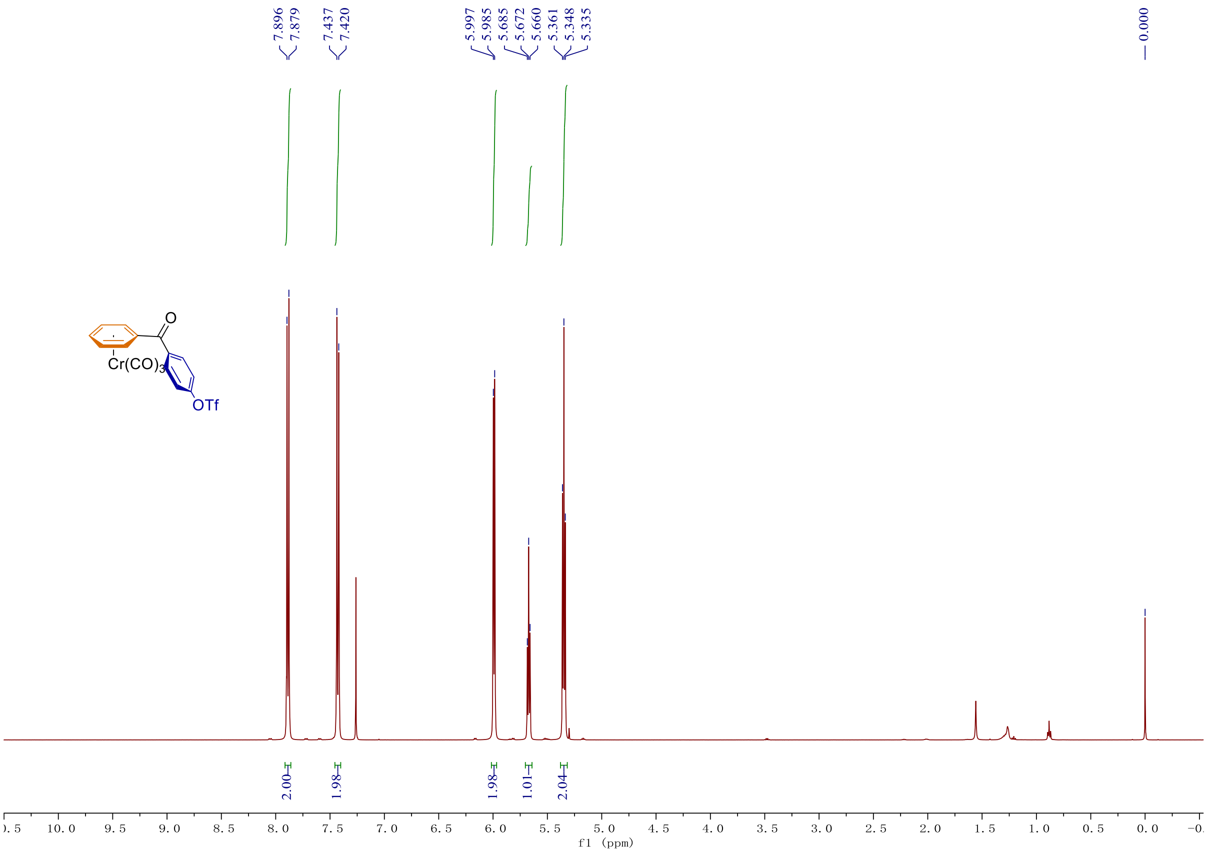

Supplementary Fig. 80 ^{1}H NMR (500 MHz, Chloroform-*d*) of 4-(((trifluoromethyl)sulfonyl)oxy)benzoylbenzene chromium tricarbonyl (1z-Cr).

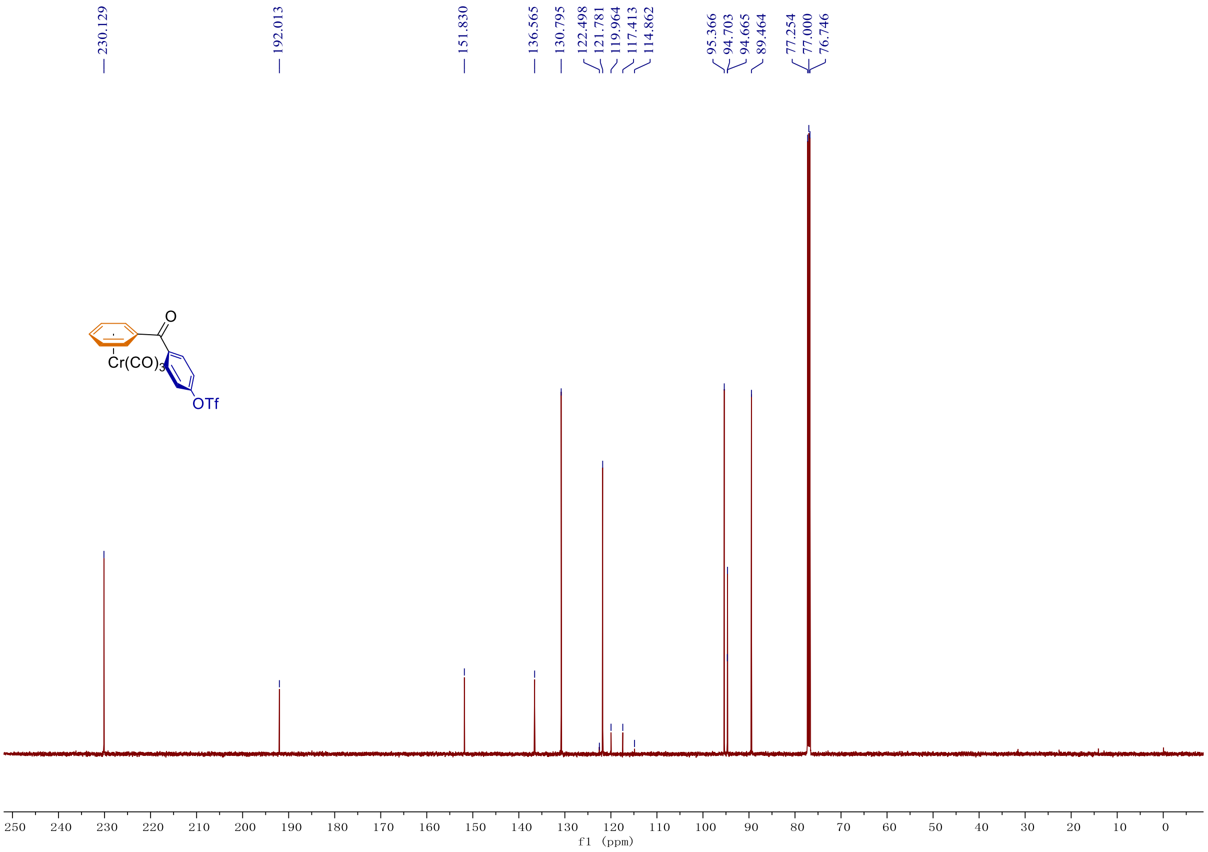

Supplementary Fig. 81 ^{13}C NMR (126 MHz, Chloroform-*d*) of 4-(((trifluoromethyl)sulfonyl)oxy)benzoylbenzene chromium tricarbonyl (1z-Cr).

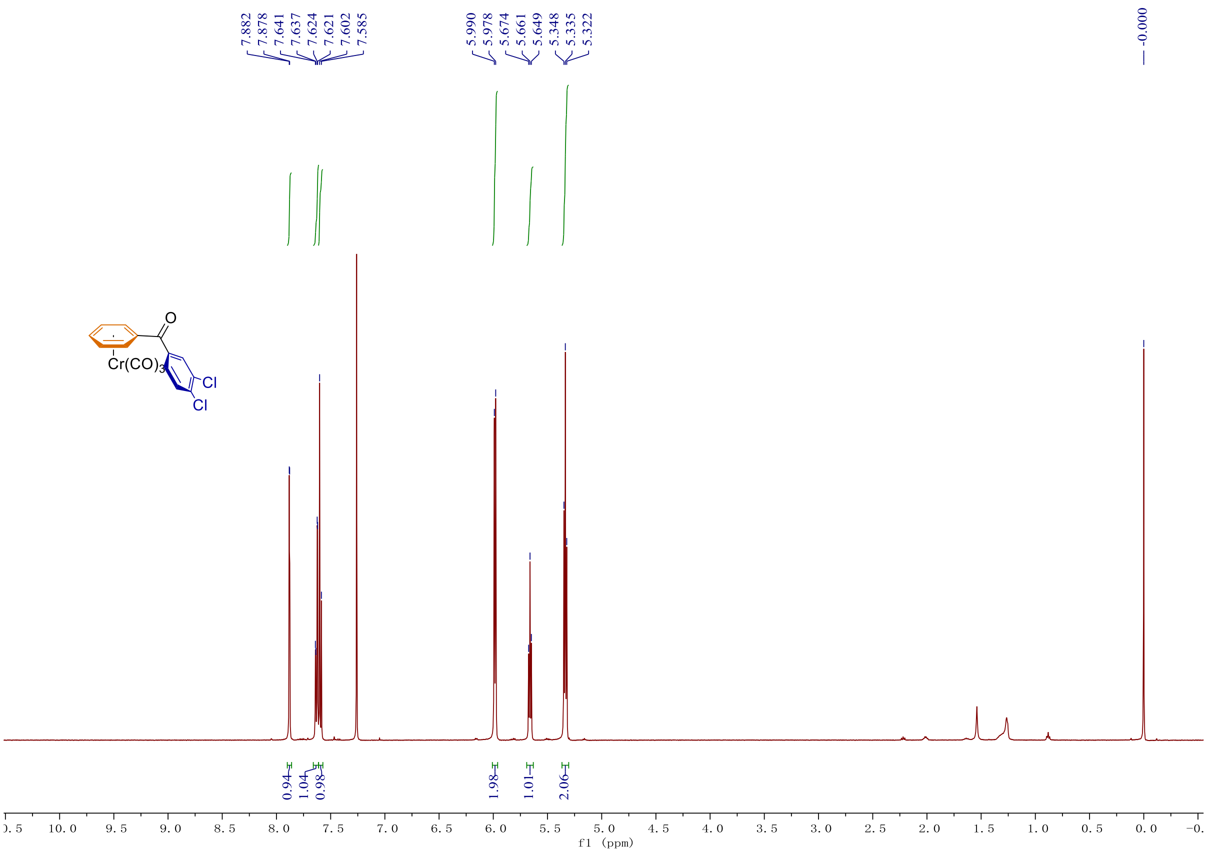

Supplementary Fig. 82 ^{1}H NMR (500 MHz, Chloroform-*d*) of 3,4-dichlorobenzoylbenzene chromium tricarbonyl (1aa-Cr).

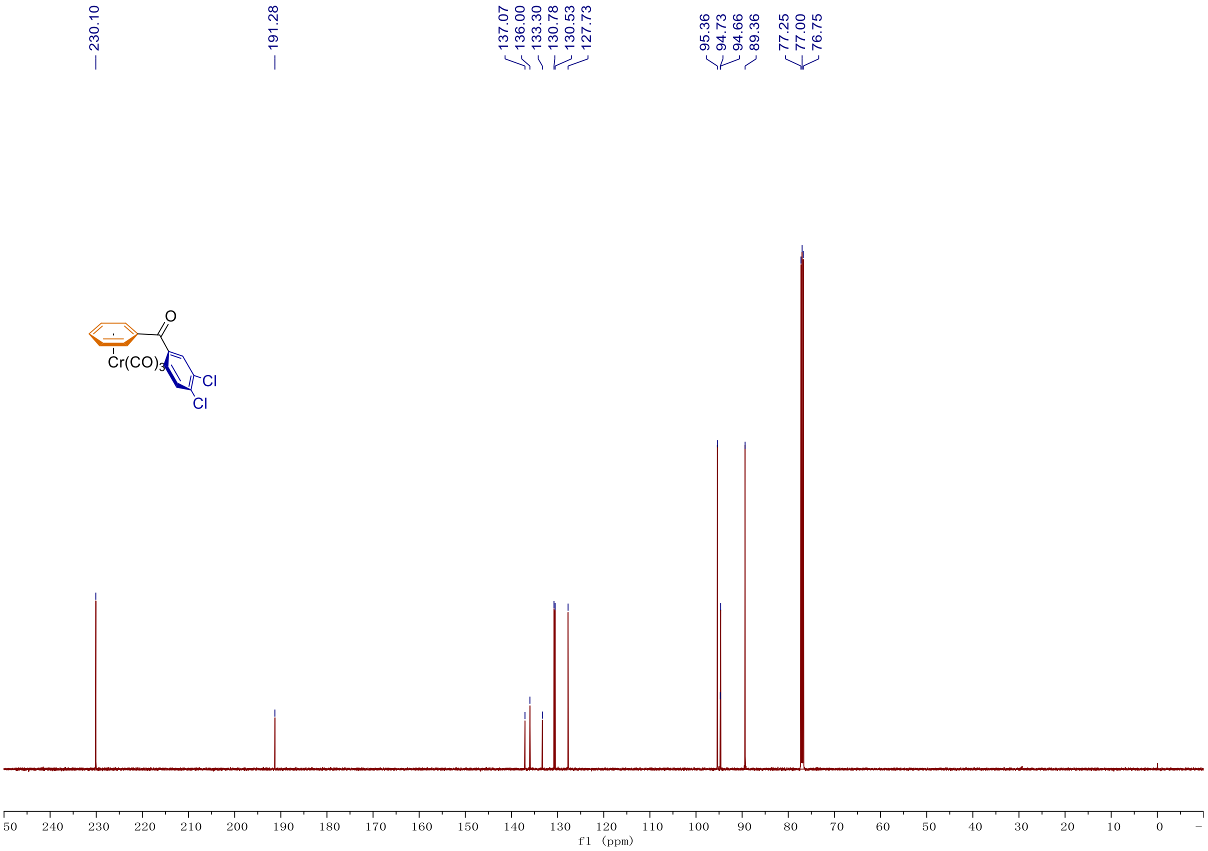

Supplementary Fig. 83 ^{13}C NMR (126 MHz, Chloroform-*d*) of 3,4-dichlorobenzoylbenzene chromium tricarbonyl (1aa-Cr).

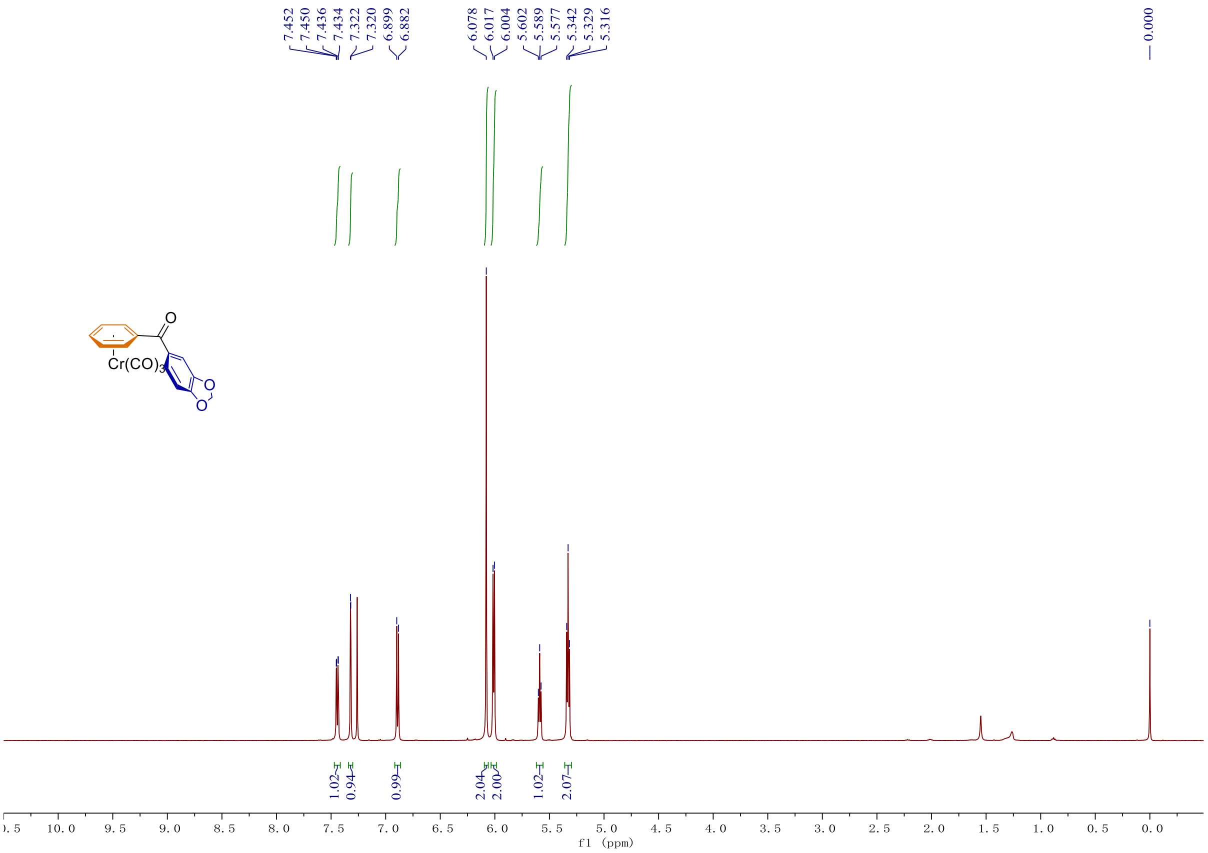

Supplementary Fig. 84 ^{1}H NMR (500 MHz, Chloroform-*d*) of (benzo[d][1,3]dioxole-5-carbonyl)benzene chromium tricarbonyl (1k-Cr).

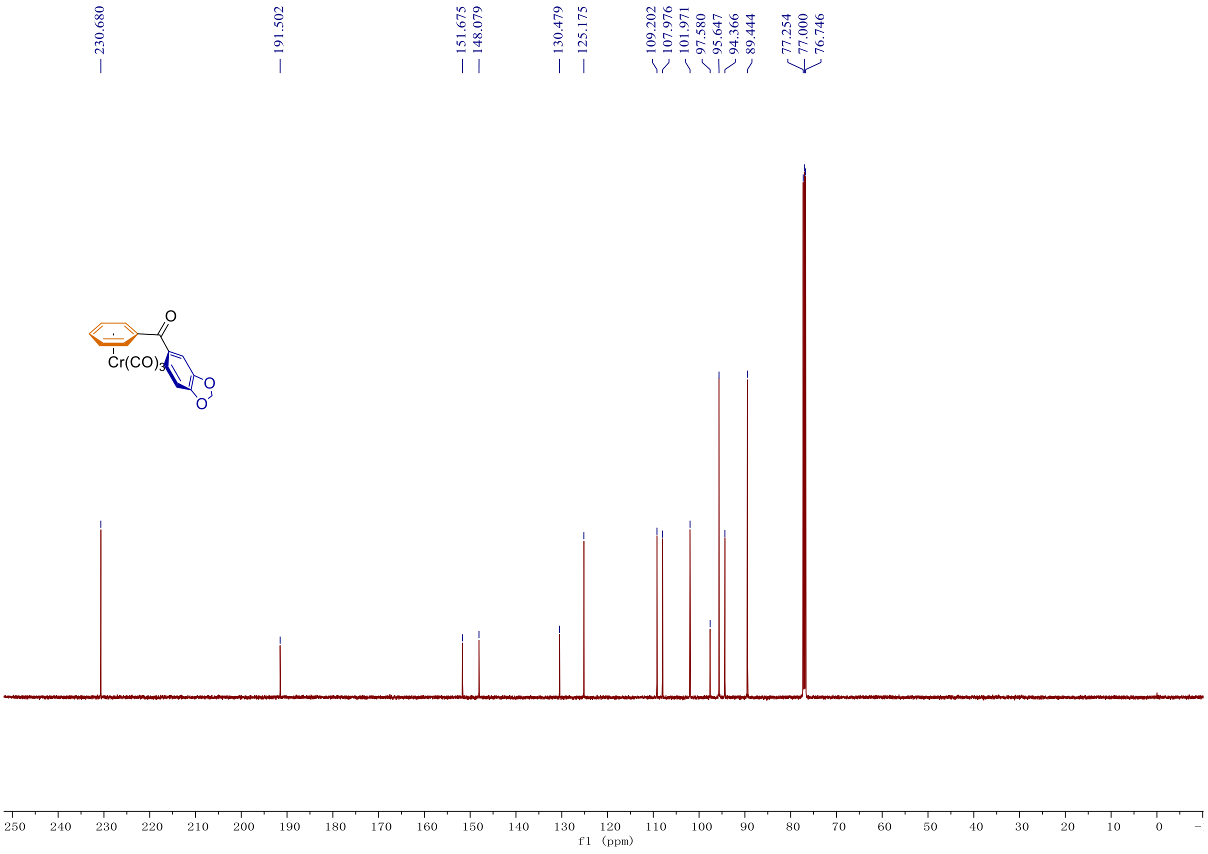

Supplementary Fig. 85 ^{13}C NMR (126 MHz, Chloroform-*d*) of (benzo[d][1,3]dioxole-5-carbonyl)benzene chromium tricarbonyl (1k-Cr).

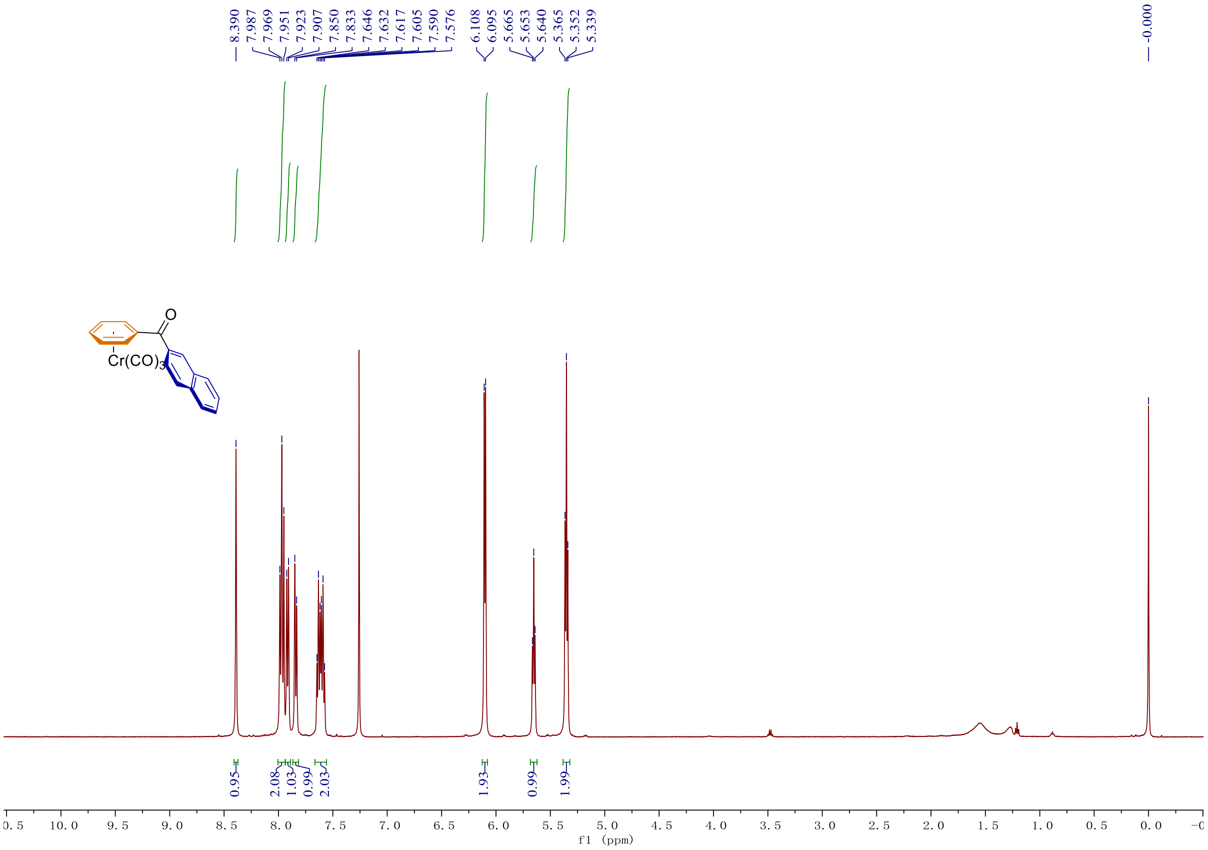

Supplementary Fig. 86 ^{1}H NMR (500 MHz, Chloroform-*d*) of 2-naphthoylbenzene chromium tricarbonyl (1ab-Cr).

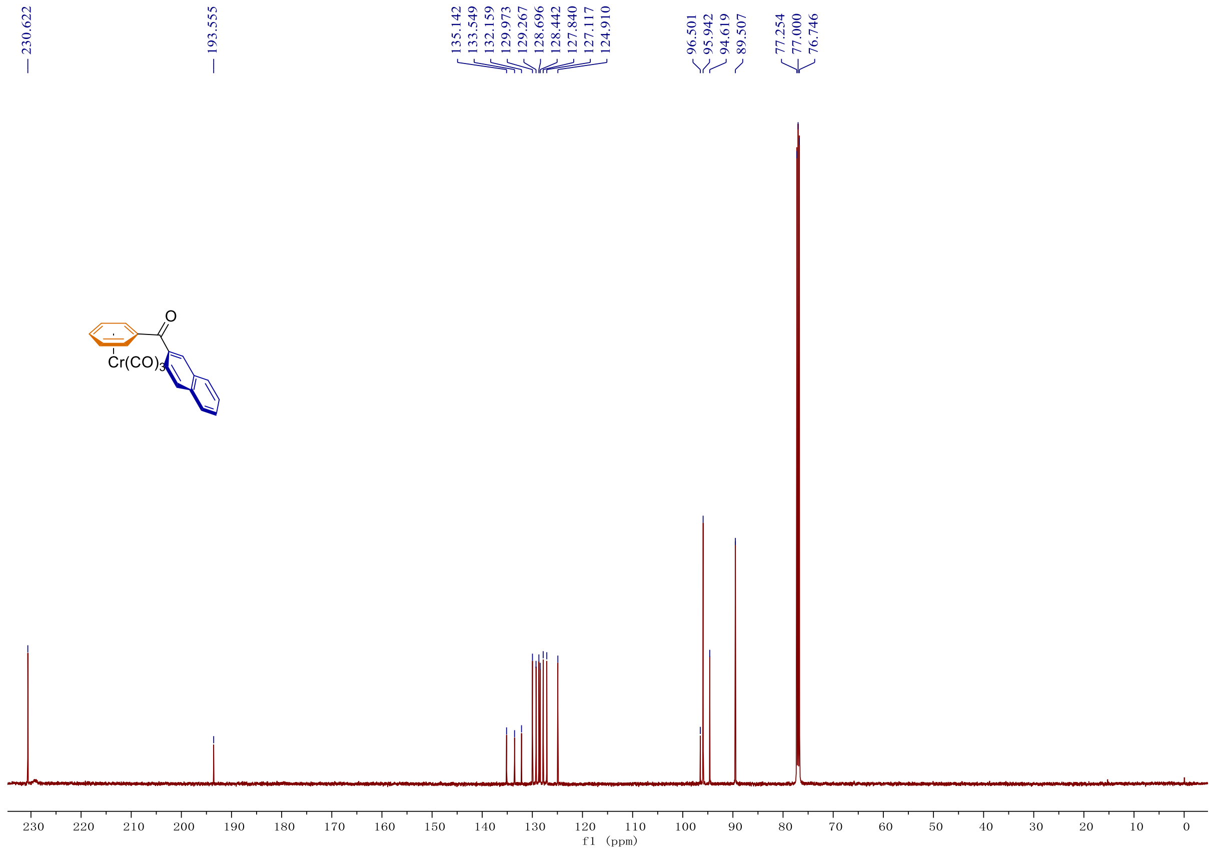

Supplementary Fig. 87 ^{13}C NMR (126 MHz, Chloroform-*d*) of 2-naphthoylbenzene chromium tricarbonyl (1ab-Cr).

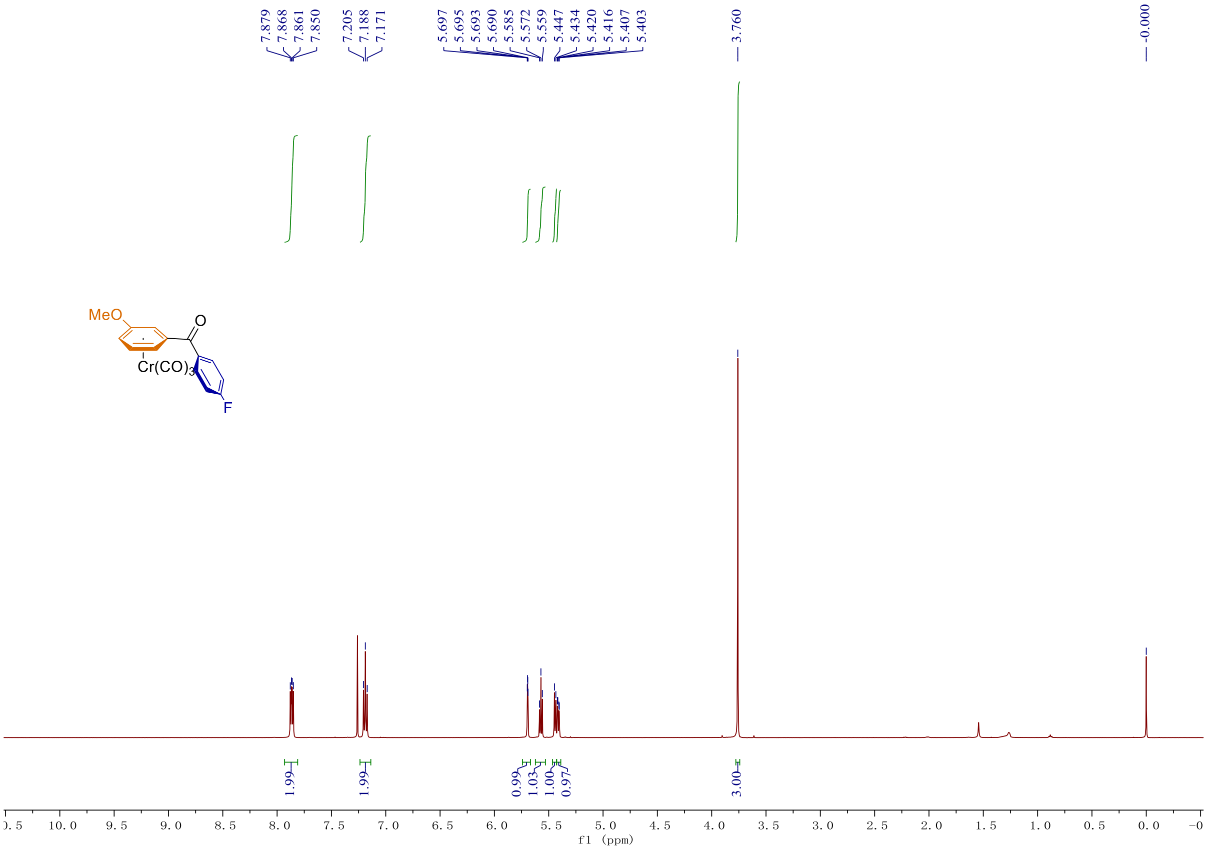

Supplementary Fig. 88 ^{1}H NMR (500 MHz, Chloroform-*d*) of 3-(4-fluorobenzoyl)anisole chromium tricarbonyl (1l-Cr).

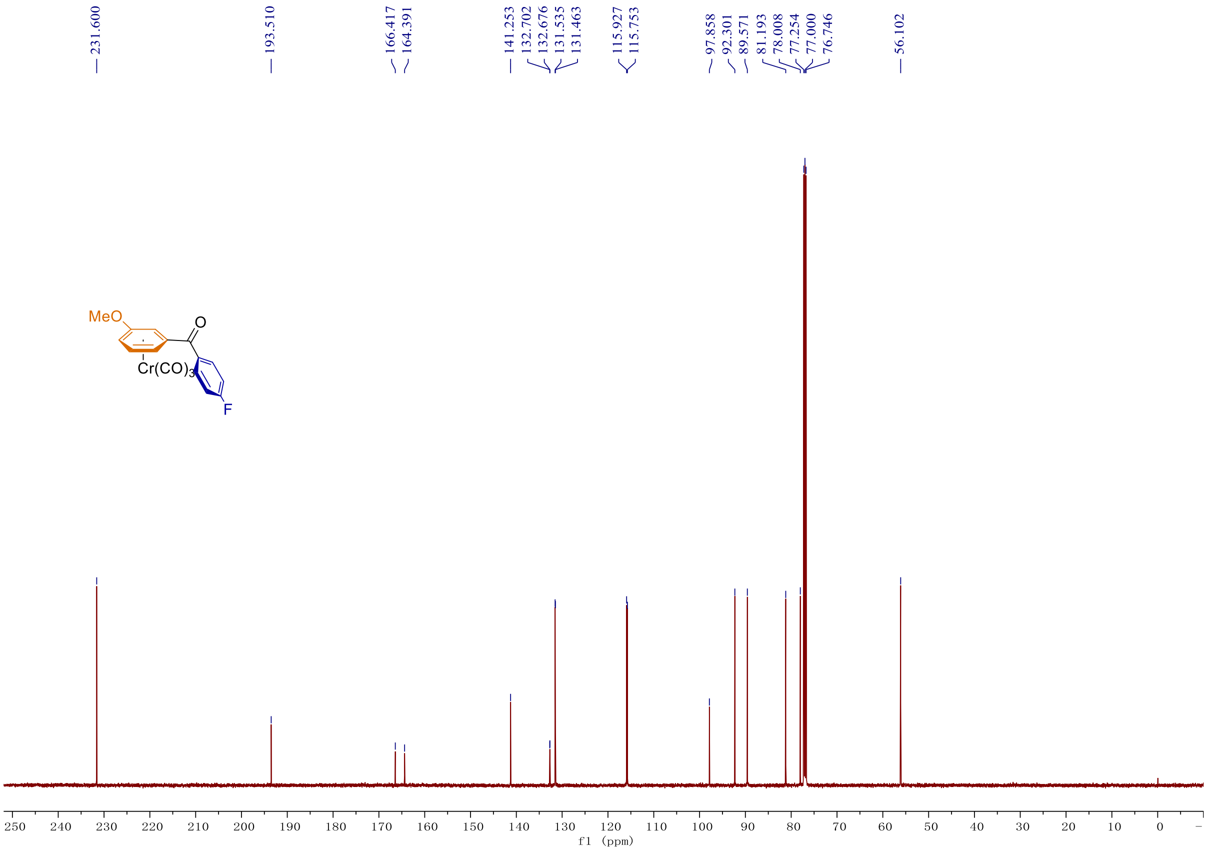

Supplementary Fig. 89 ^{13}C NMR (126 MHz, Chloroform-*d*) of 3-(4-fluorobenzoyl)anisole chromium tricarbonyl (1l-Cr).

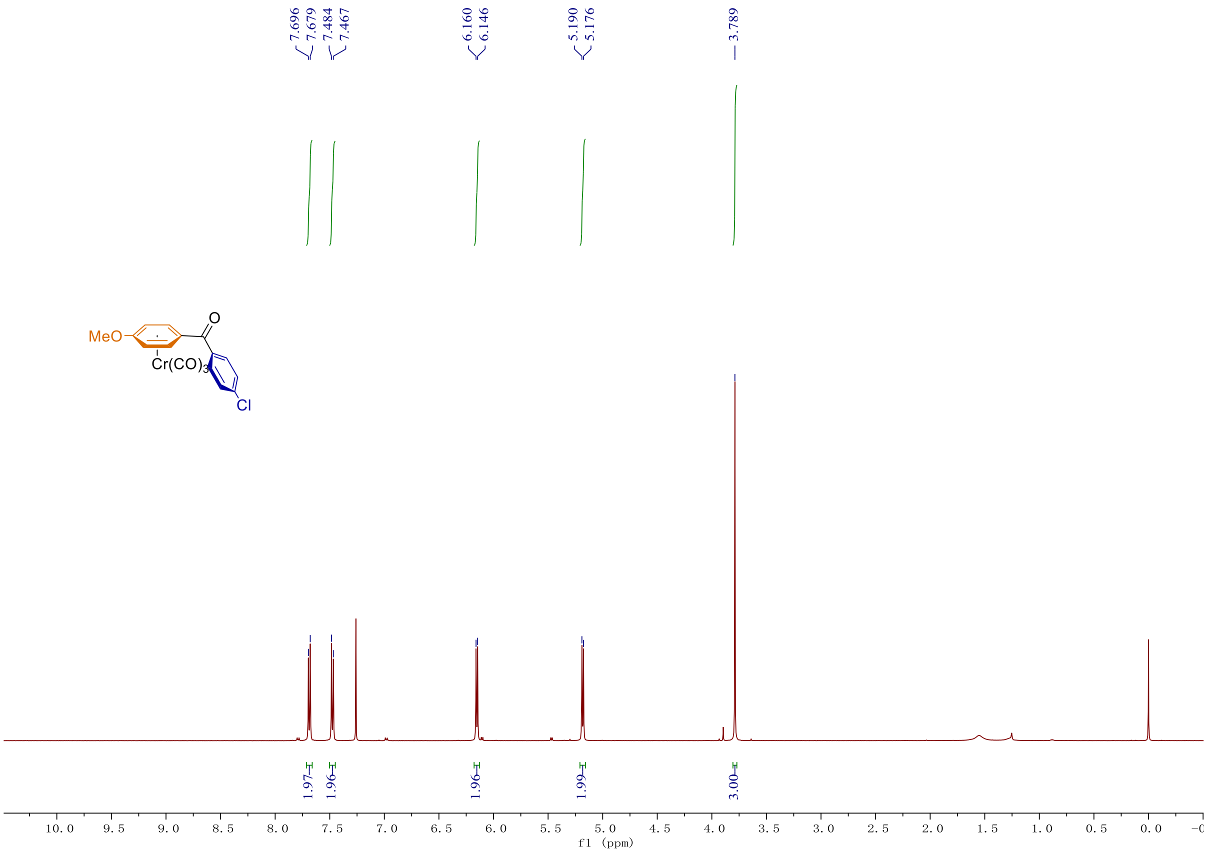

Supplementary Fig. 90 ^{1}H NMR (500 MHz, Chloroform-*d*) of 4-(4-chlorobenzoyl)anisole chromium tricarbonyl (1m-Cr).

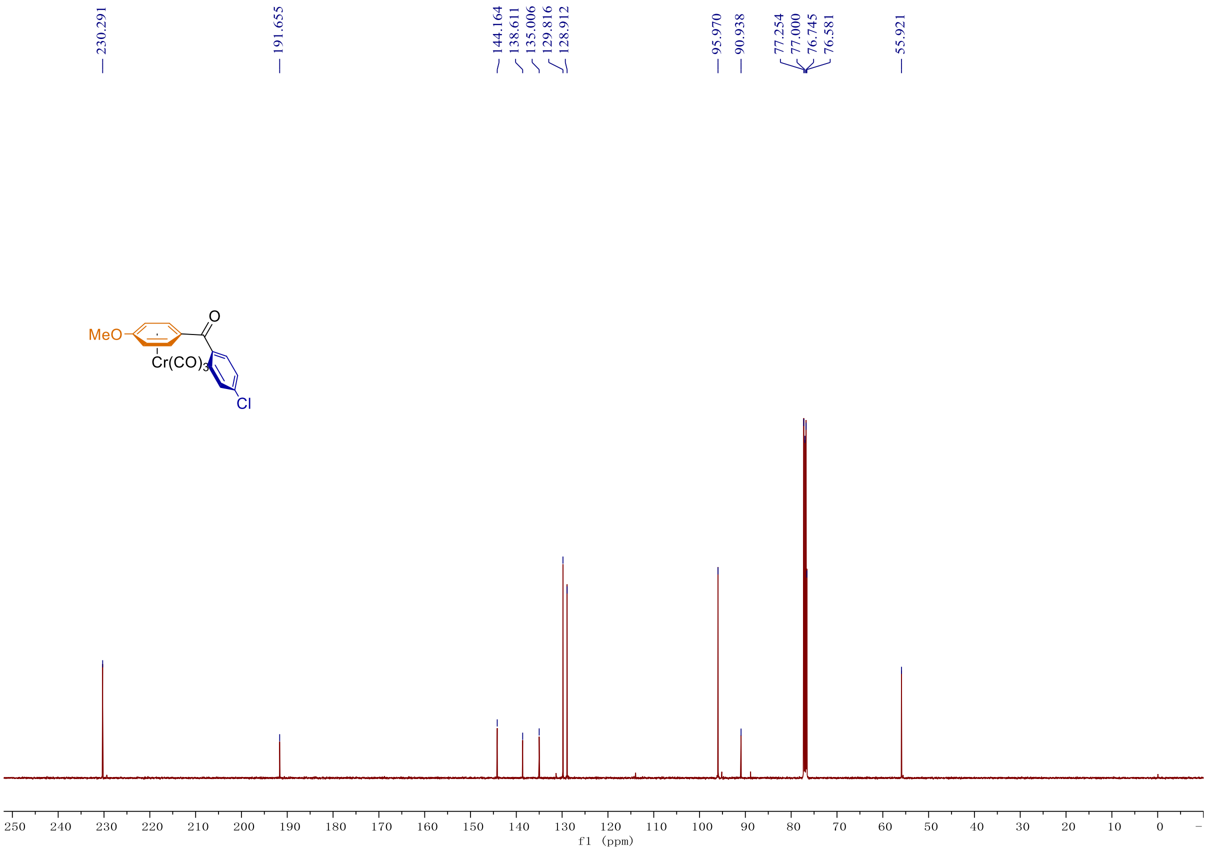

Supplementary Fig. 91 ^{13}C NMR (126 MHz, Chloroform-*d*) of 4-(4-chlorobenzoyl)anisole chromium tricarbonyl (1m-Cr).

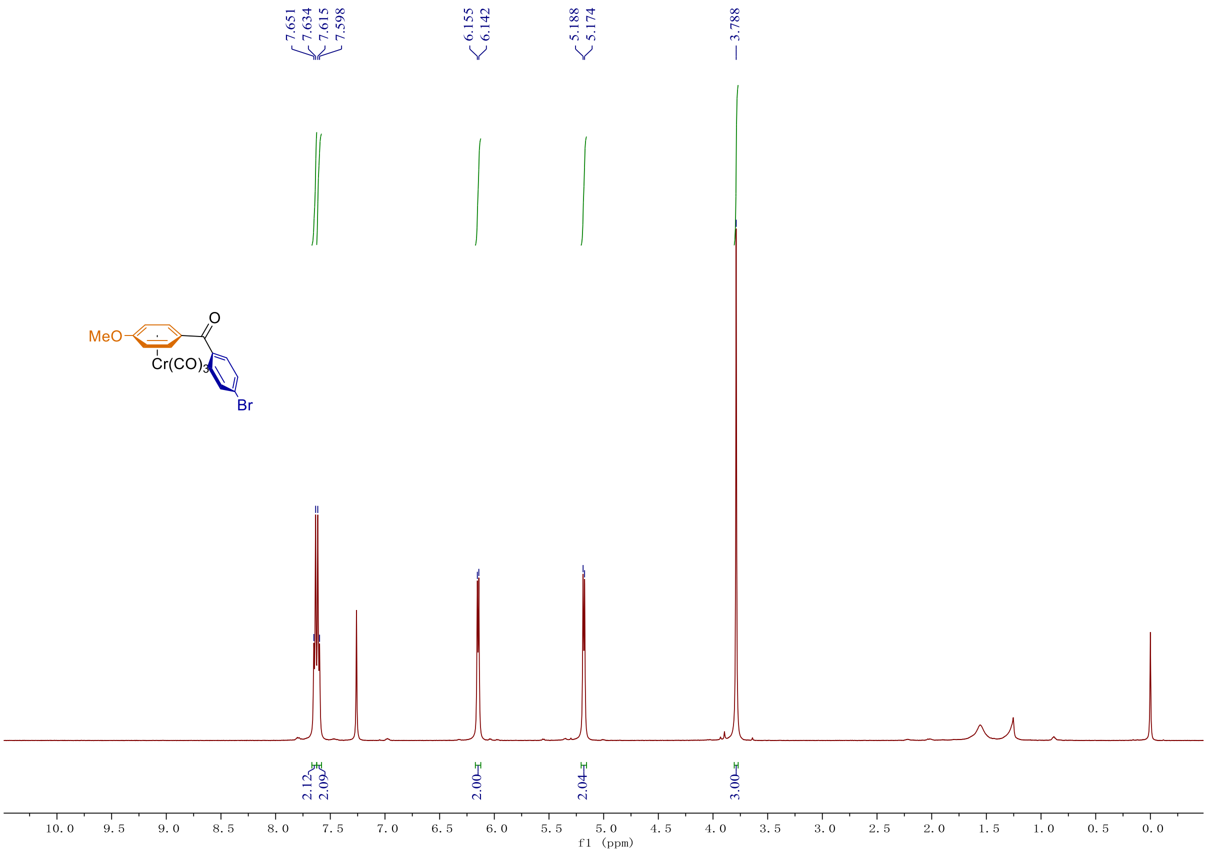

Supplementary Fig. 92 ^{1}H NMR (500 MHz, Chloroform-*d*) of 4-(4-bromobenzoyl)anisole chromium tricarbonyl (1n-Cr).

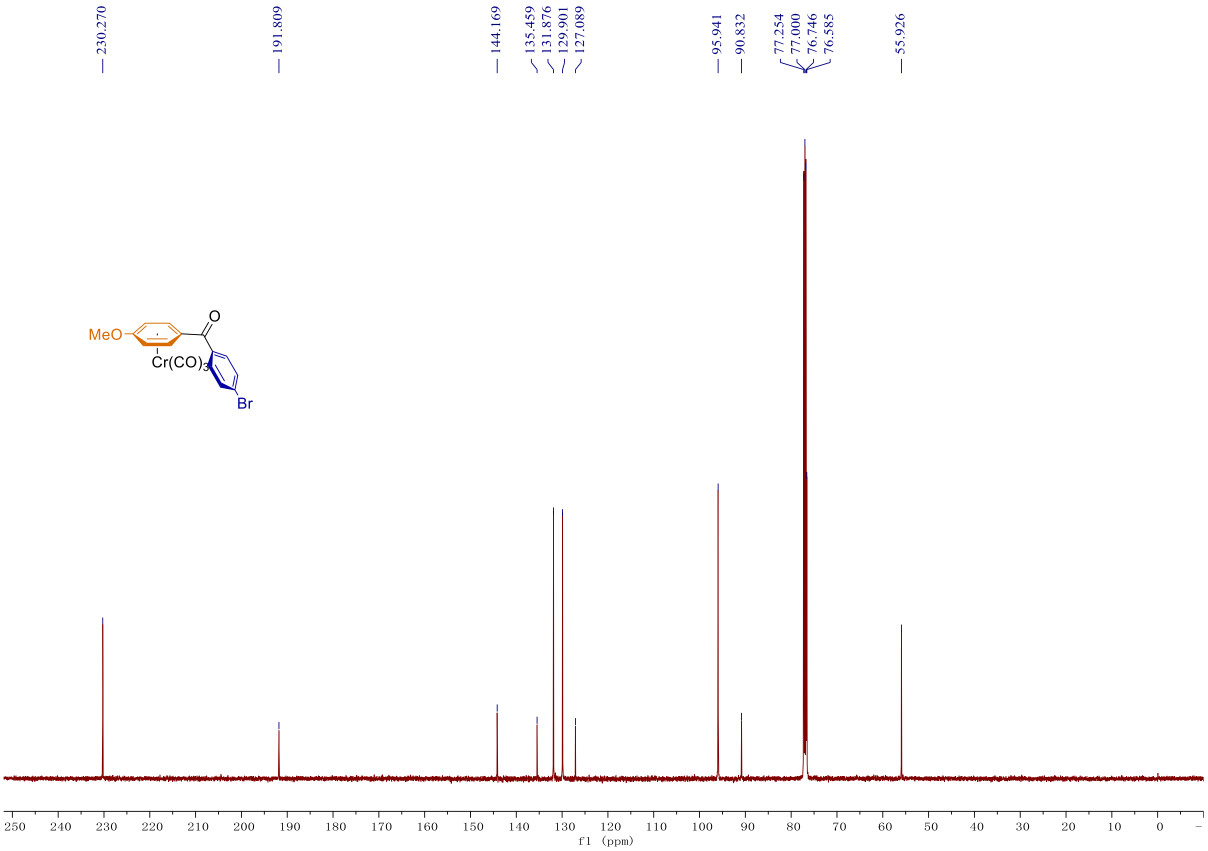

Supplementary Fig. 93 ^{13}C NMR (126 MHz, Chloroform-*d*) of 4-(4-bromobenzoyl)anisole chromium tricarbonyl (1n-Cr).

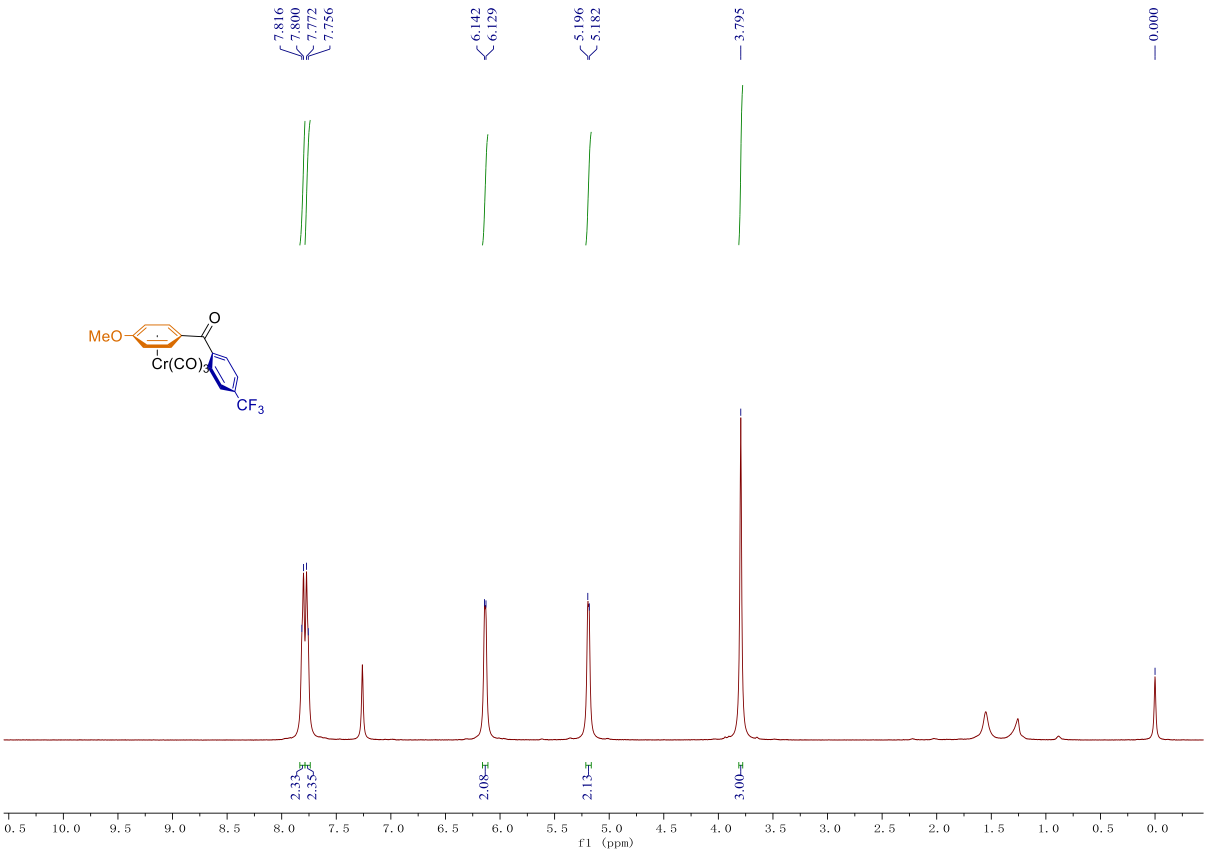

Supplementary Fig. 94 ^{1}H NMR (500 MHz, Chloroform-*d*) of 4-(4-(trifluomethyl)benzoyl)anisole chromium tricarbonyl (1o-Cr).

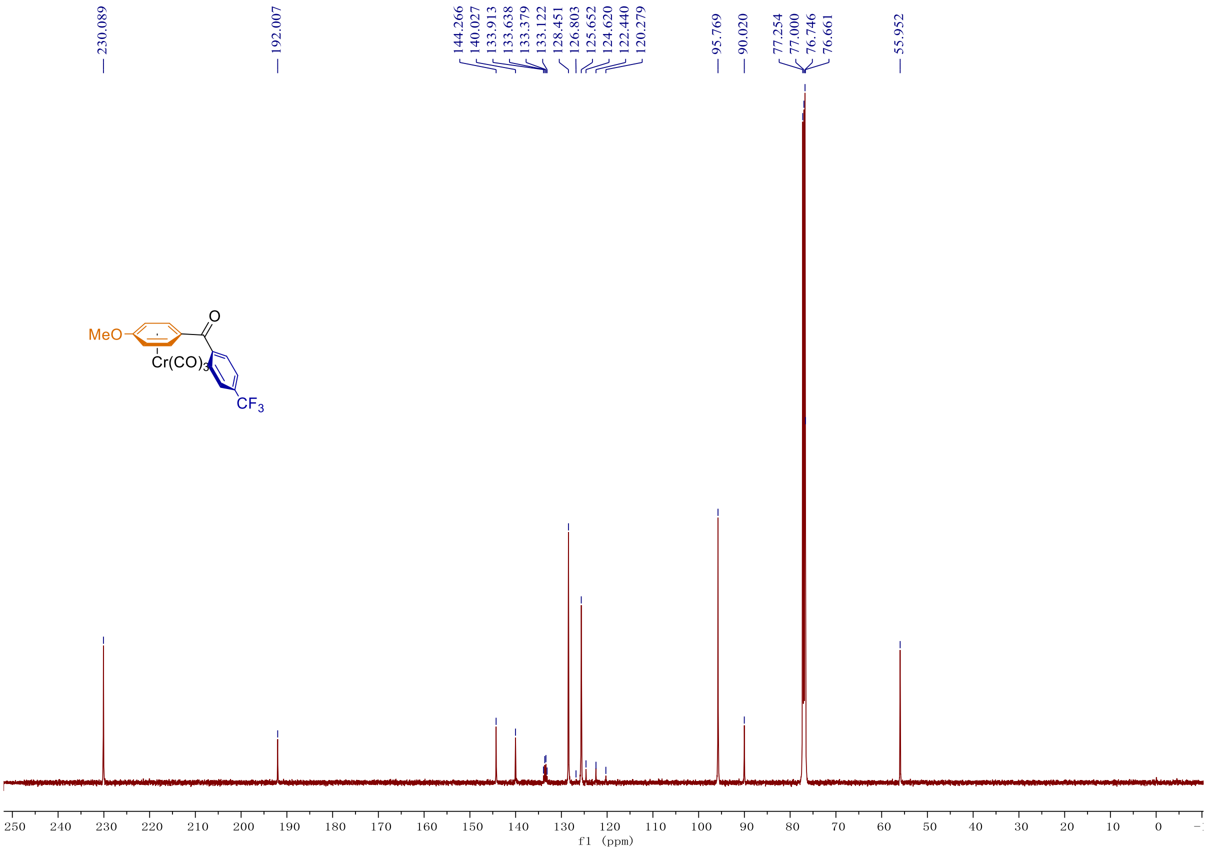

Supplementary Fig. 95 ^{13}C NMR (126 MHz, Chloroform-*d*) of 4-(4-(trifluomethyl)benzoyl)anisole chromium tricarbonyl (1o-Cr).

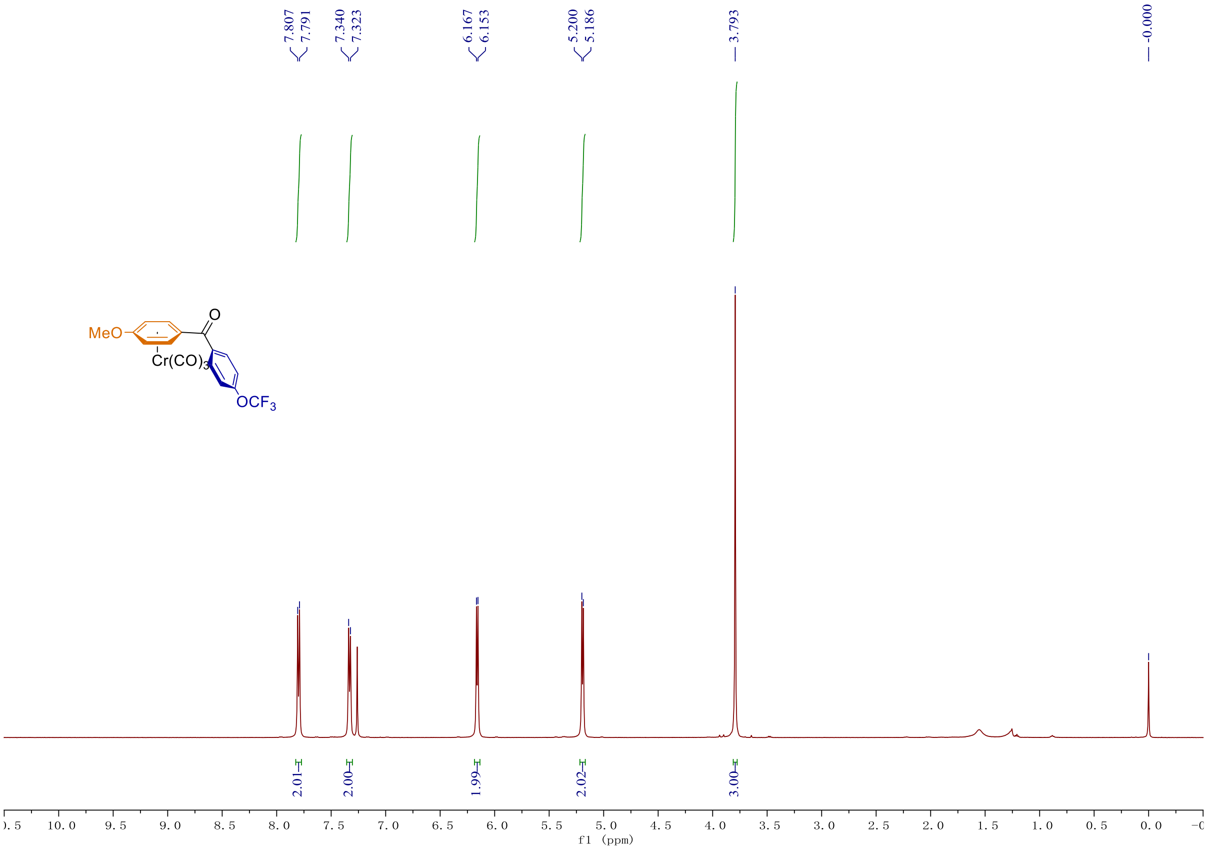

Supplementary Fig. 96 ^{1}H NMR (500 MHz, Chloroform-*d*) of 4-(4-(trifluoromethoxy)benzoyl)anisole chromium tricarbonyl (1p-Cr).

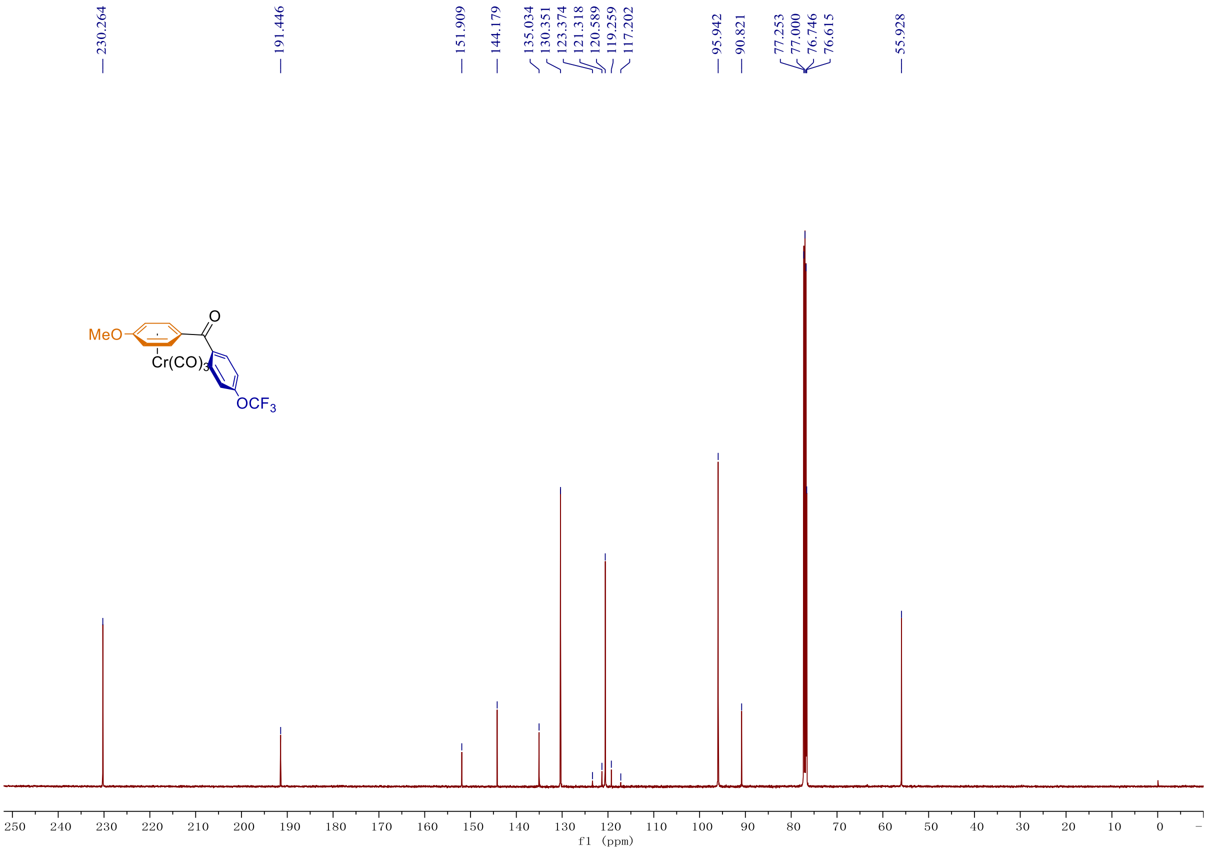

Supplementary Fig. 97 ^{13}C NMR (126 MHz, Chloroform-*d*) of 4-(4-(trifluoromethoxy)benzoyl)anisole chromium tricarbonyl (1p-Cr).

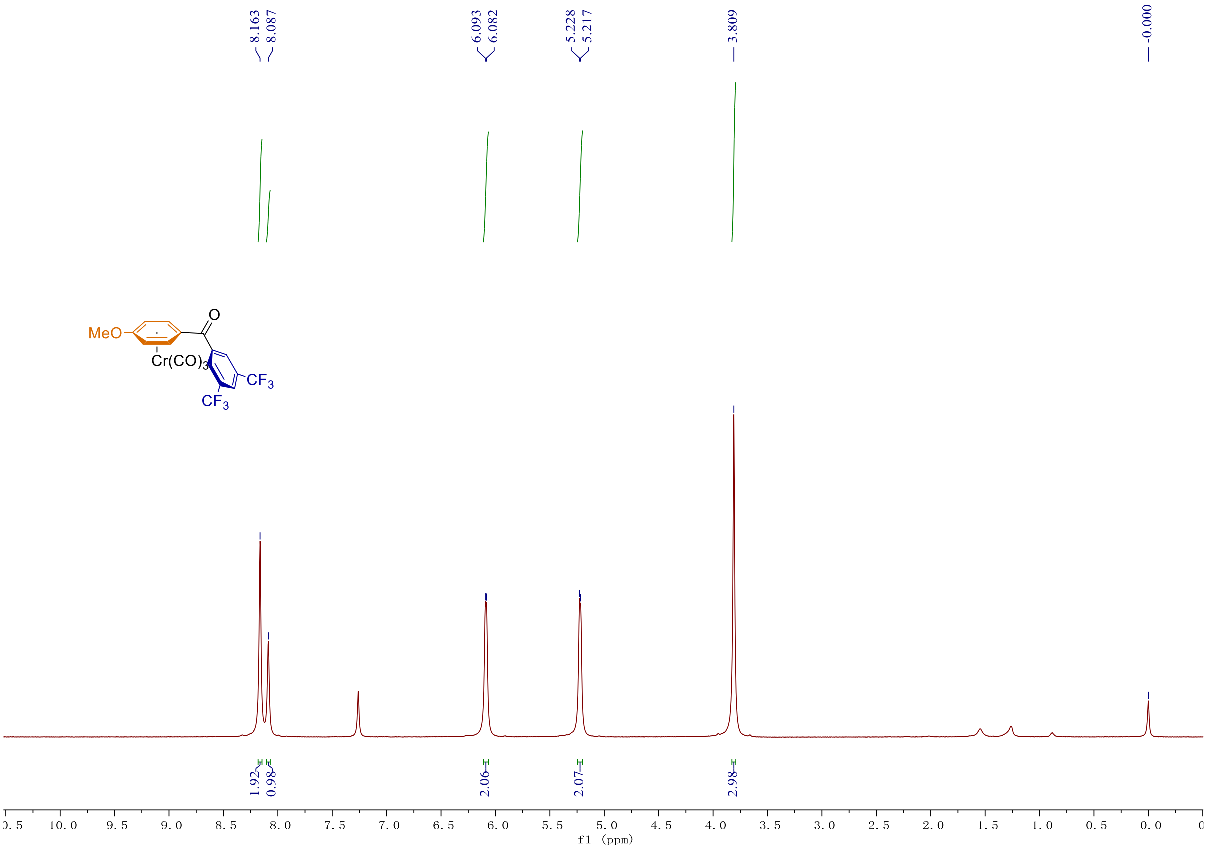

Supplementary Fig. 98 ^{1}H NMR (500 MHz, Chloroform-*d*) of 4-(3,5-bis(trifluomethyl)benzoyl)anisole chromium tricarbonyl (s3-Cr).

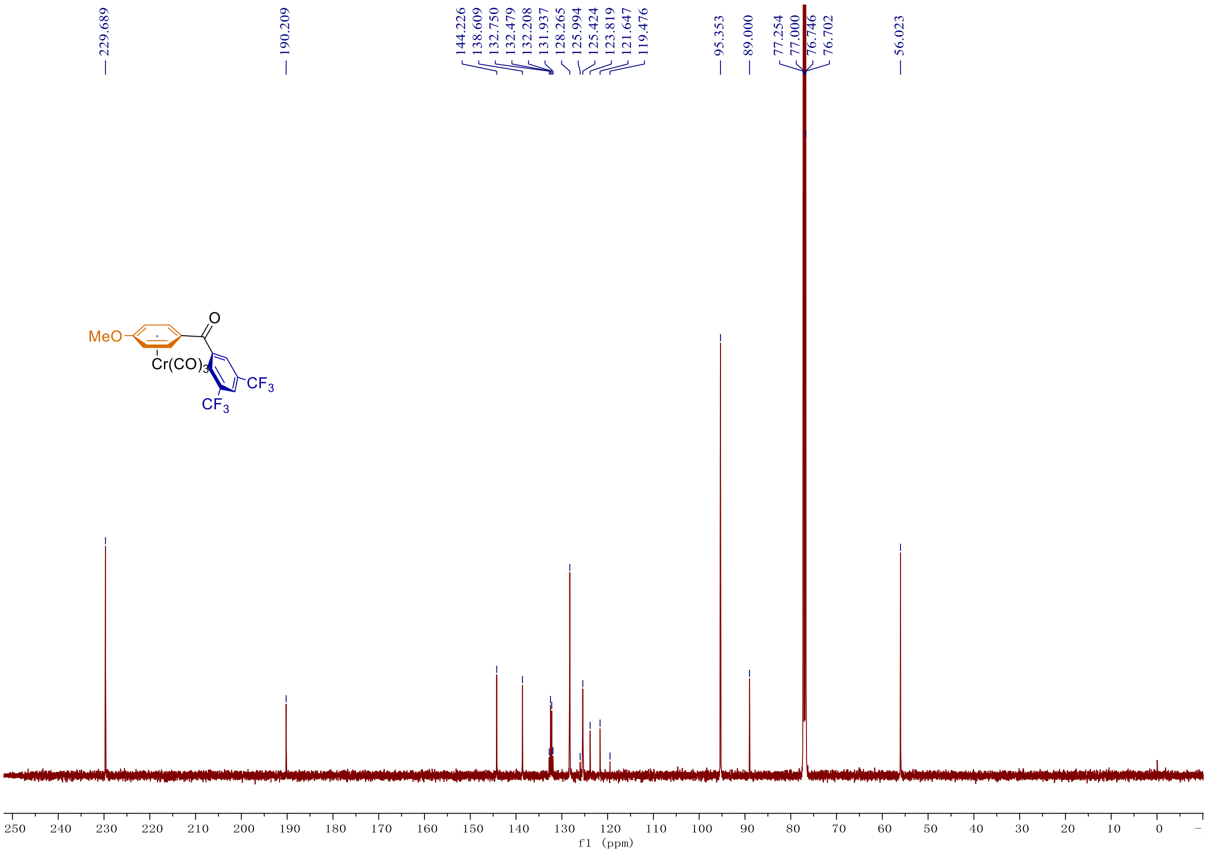

Supplementary Fig. 99 ^{13}C NMR (126 MHz, Chloroform-*d*) of 4-(3,5-bis(trifluomethyl)benzoyl)anisole chromium tricarbonyl (s3-Cr).

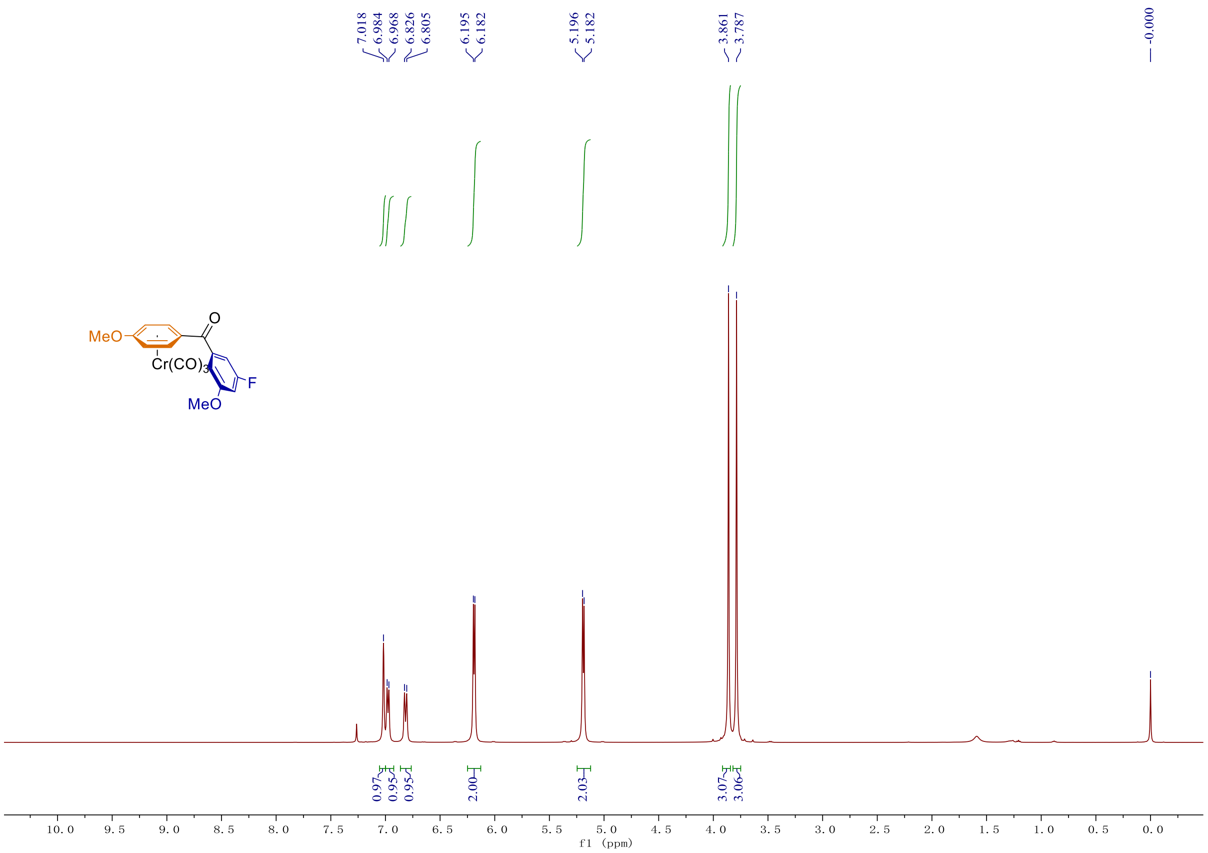

Supplementary Fig. 100 ^{1}H NMR (500 MHz, Chloroform-*d*) of 4-(3-methoxy-5-fluorobenzoyl)anisole chromium tricarbonyl (1v-Cr).

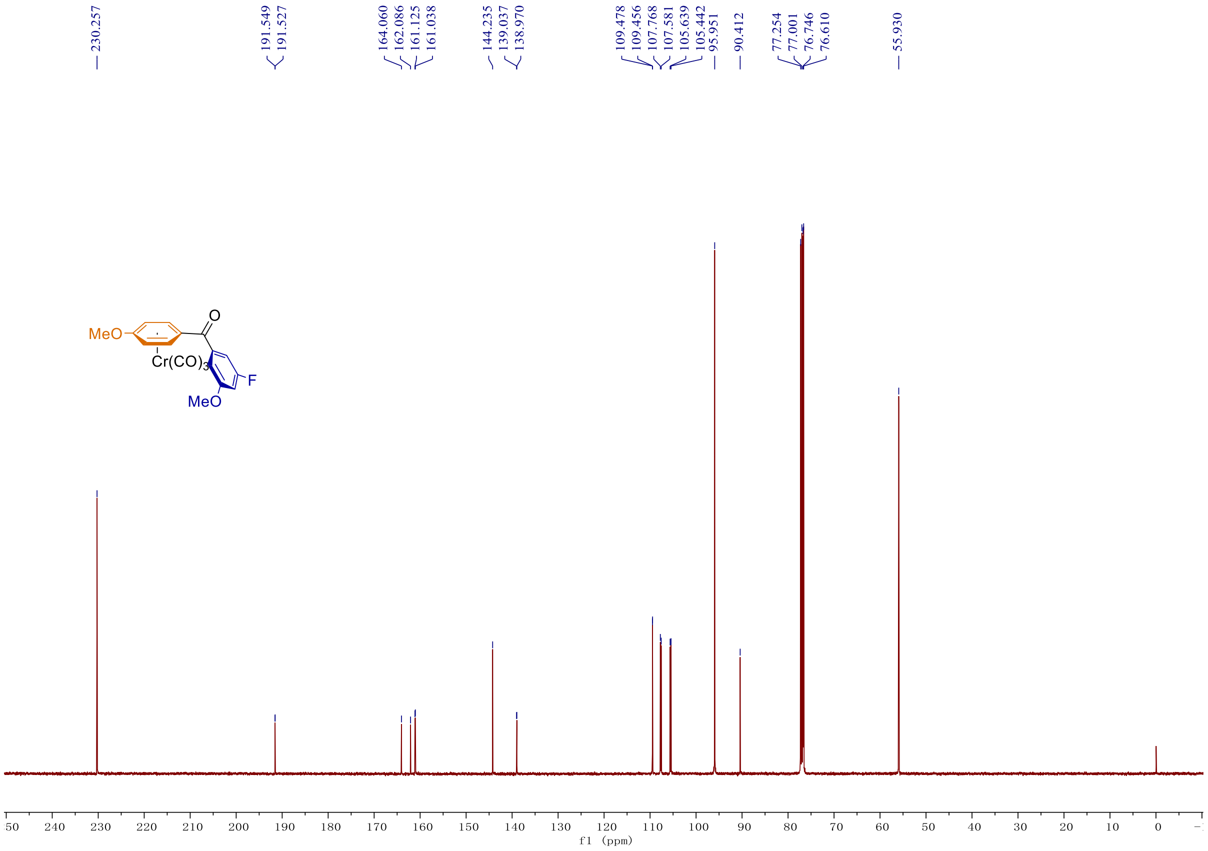

Supplementary Fig. 101 ^{13}C NMR (126 MHz, Chloroform-*d*) of 4-(3-methoxy-5-fluorobenzoyl)anisole chromium tricarbonyl (1v-Cr).

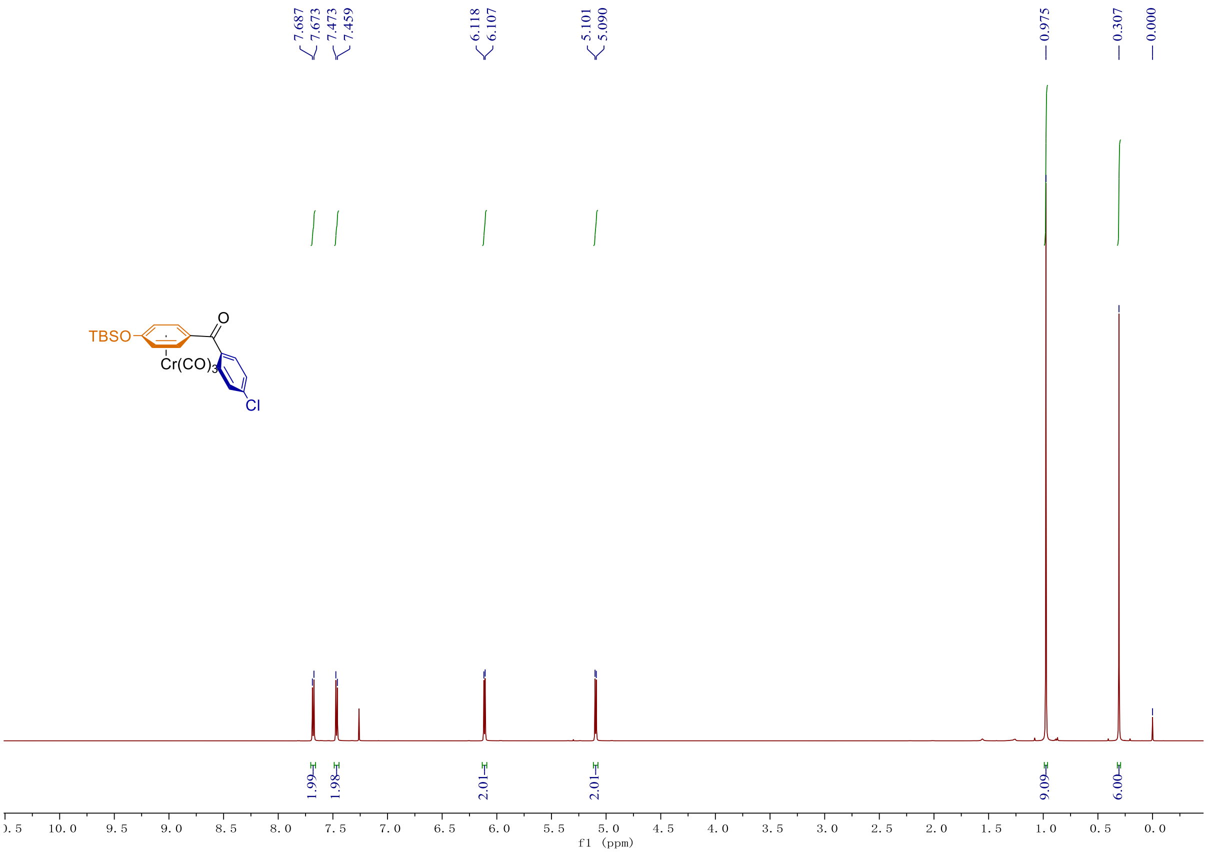

Supplementary Fig. 102 ^{1}H NMR (600 MHz, Chloroform-*d*) of 4-(4-chlorobenzoyl)(((tert-butyldimethylsilyl)oxy)benzene) chromium tricarbonyl (1q-Cr).

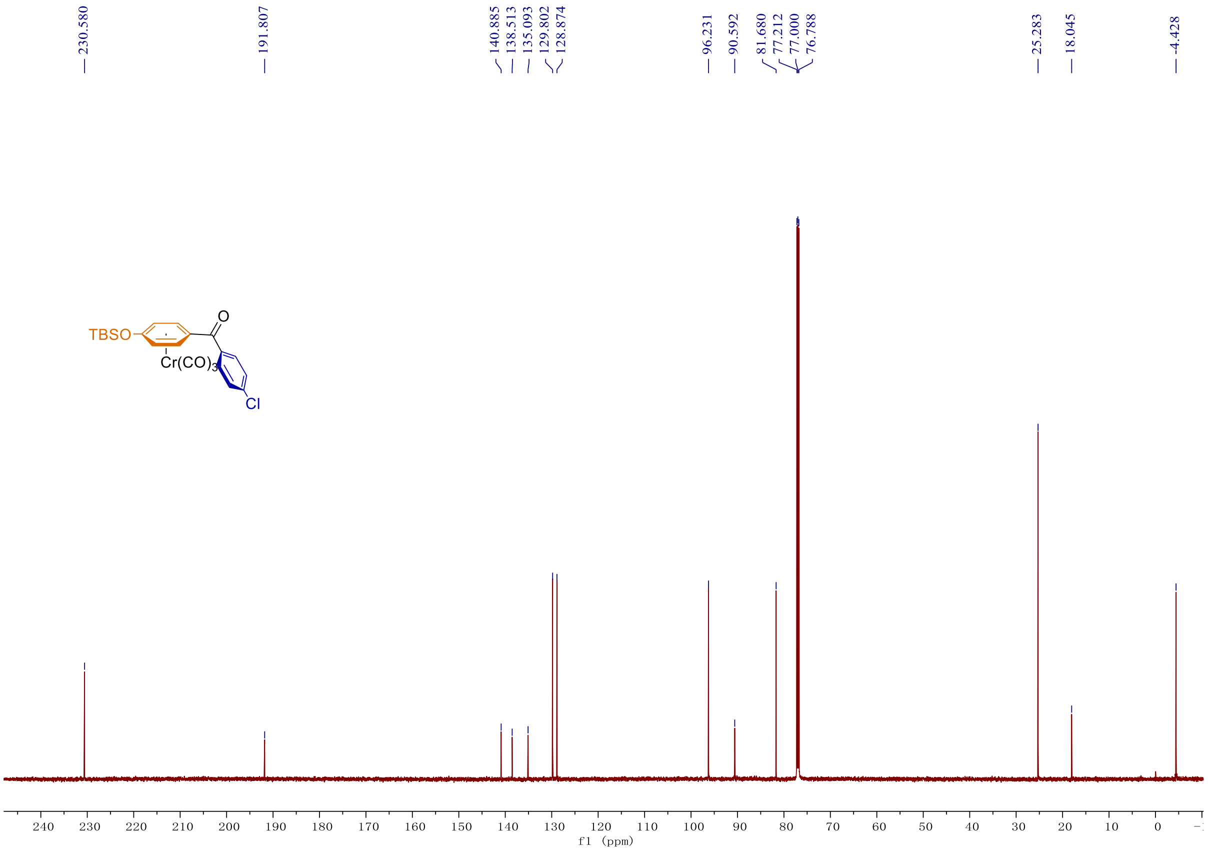

Supplementary Fig. 103 ^{13}C NMR (151 MHz, Chloroform-*d*) of 4-(4-chlorobenzoyl)(((tert-butyldimethylsilyl)oxy)benzene) chromium tricarbonyl (1q-Cr).

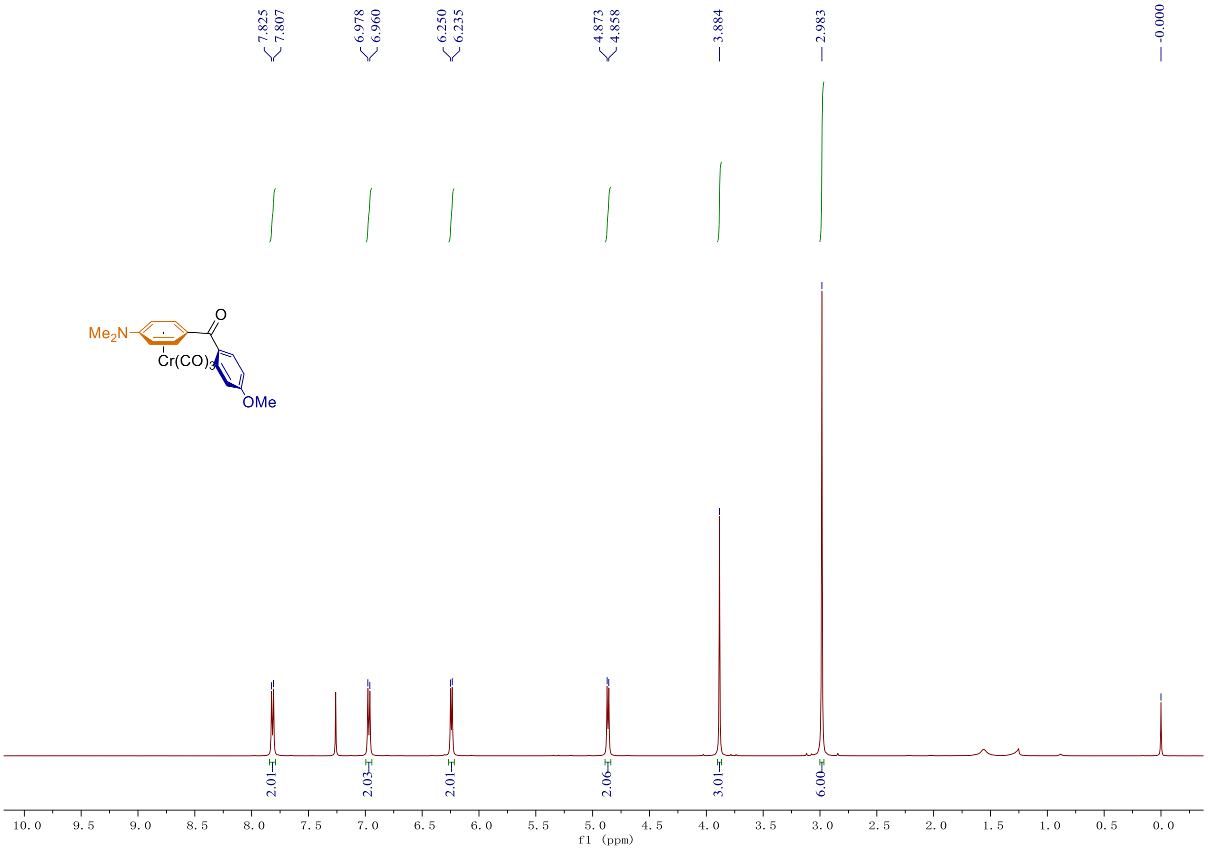

Supplementary Fig. 104 ^{1}H NMR (500 MHz, Chloroform-*d*) of 4-(4-methoxybenzoyl)nn-dimethylaniline chromium tricarbonyl (1r-Cr).

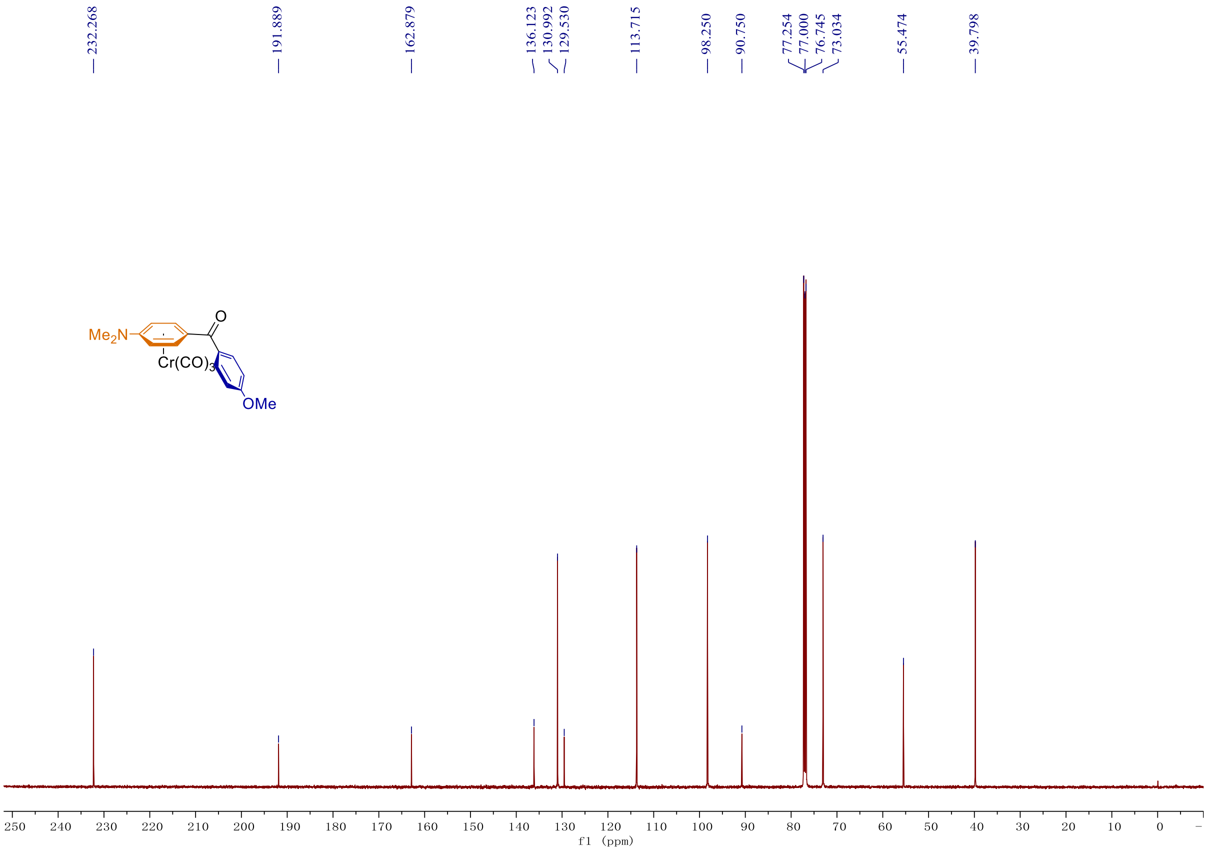

Supplementary Fig. 105 ^{13}C NMR (126 MHz, Chloroform-*d*) of 4-(4-methoxybenzoyl)nn-dimethylaniline chromium tricarbonyl (1r-Cr).

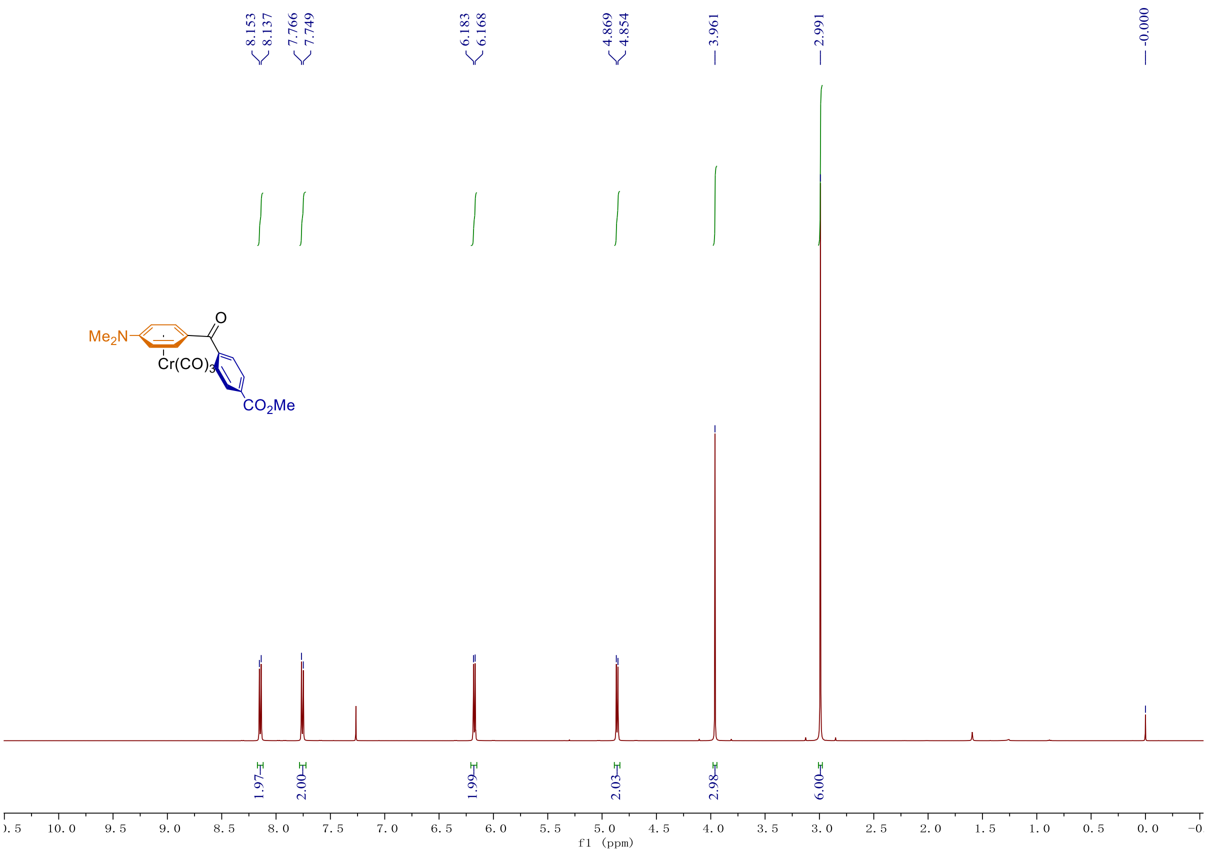

Supplementary Fig. 106 ^{1}H NMR (500 MHz, Chloroform-*d*) of 4-(4-methoxycarbonyl)nn-dimethylaniline chromium tricarbonyl (1s-Cr).

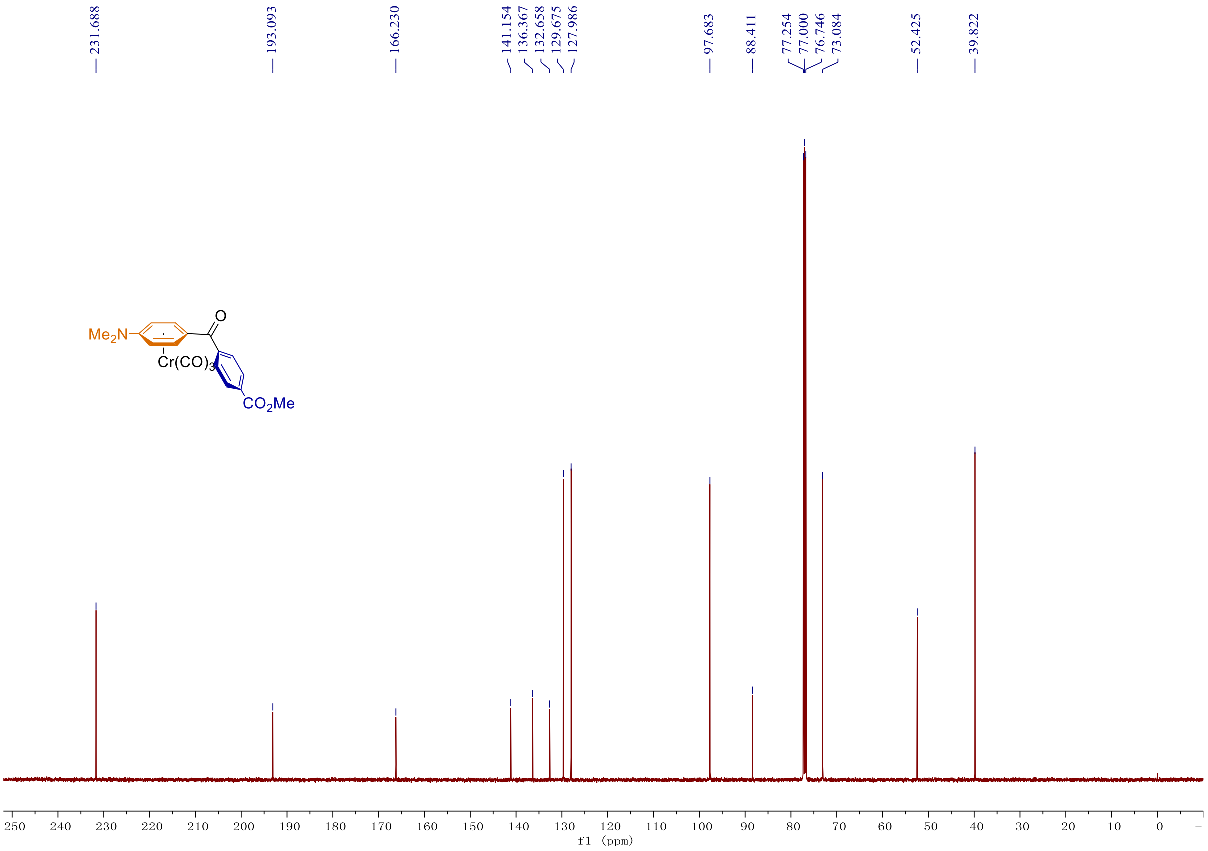

Supplementary Fig. 107 ^{13}C NMR (126 MHz, Chloroform-*d*) of 4-(4-methoxycarbonyl)nn-dimethylaniline chromium tricarbonyl (1s-Cr).

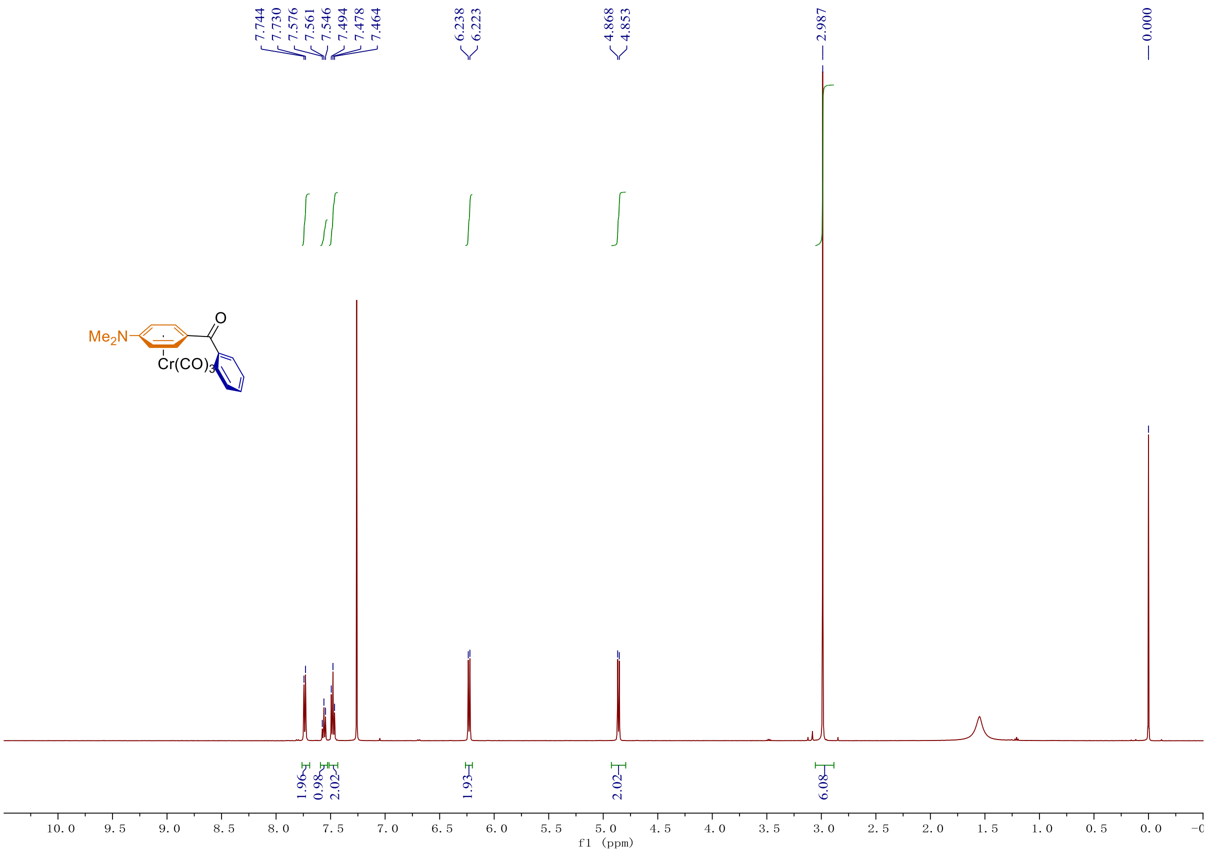

Supplementary Fig. 108 ^{1}H NMR (500 MHz, Chloroform-*d*) of 4-(benzoyl)nn-dimethylaniline chromium tricarbonyl (1j-Cr).

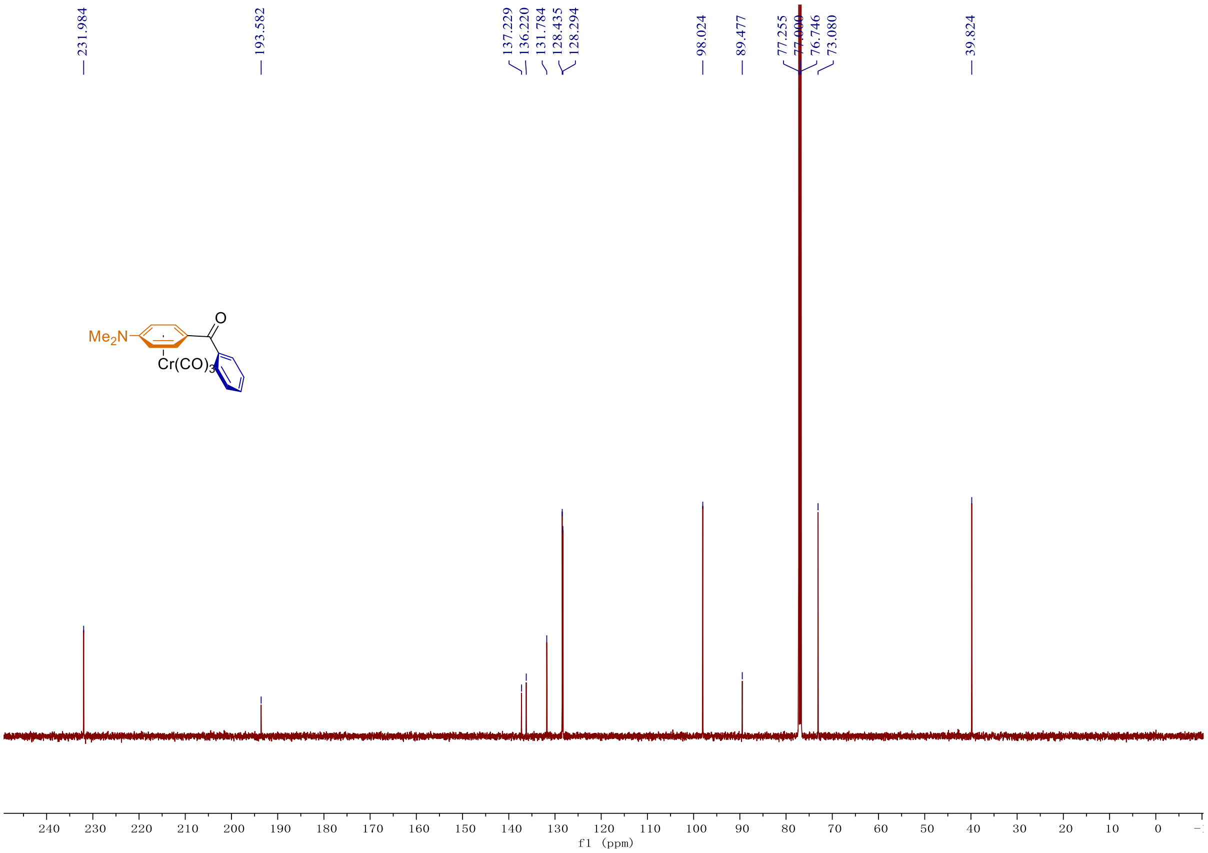

Supplementary Fig. 109 ^{13}C NMR (126 MHz, Chloroform-*d*) of 4-(benzoyl)nn-dimethylaniline chromium tricarbonyl (1j-Cr).

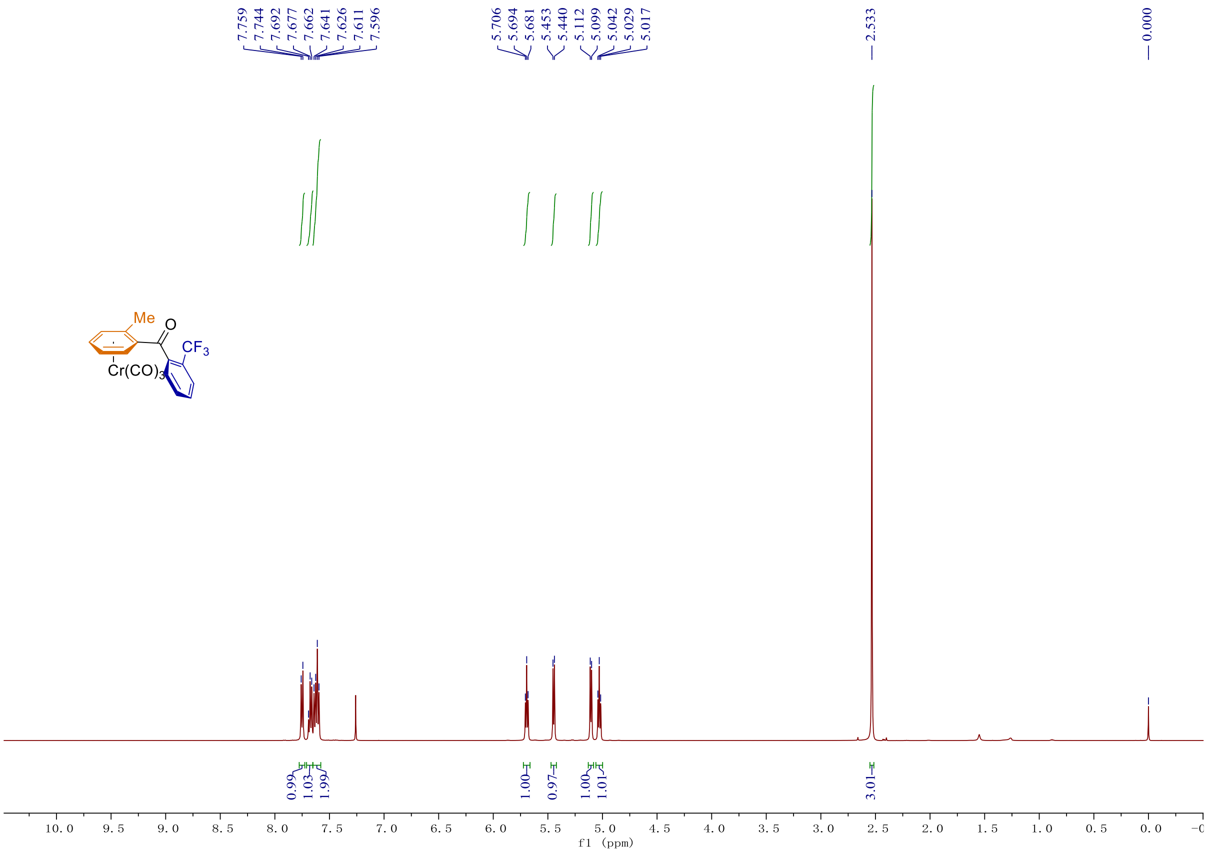

Supplementary Fig. 110 ^{1}H NMR (500 MHz, Chloroform-*d*) of 2-(2-(trifluoromethyl)benzoyl)toluene chromium tricarbonyl (1u-Cr).

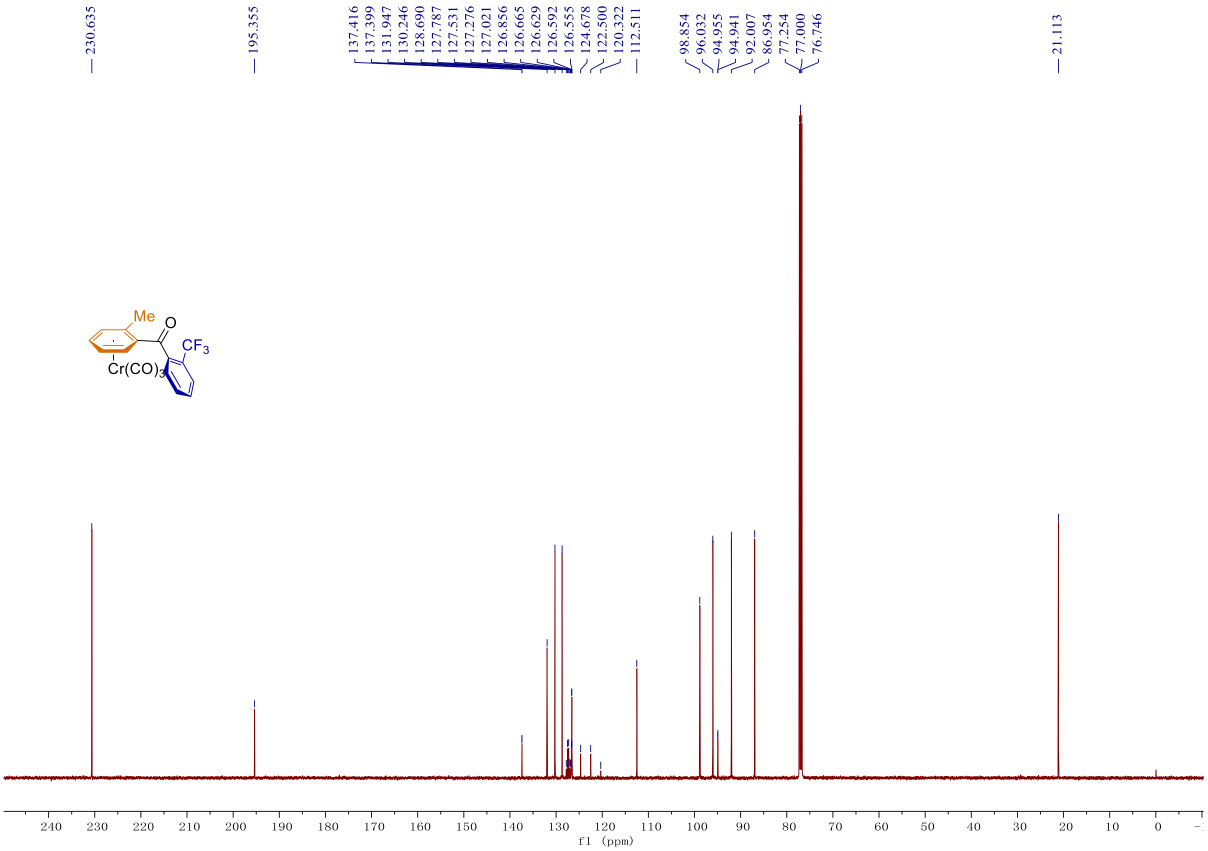

Supplementary Fig. 111 ^{13}C NMR (126 MHz, Chloroform-*d*) of 2-(2-(trifluoromethyl)benzoyl)toluene chromium tricarbonyl (1u-Cr).

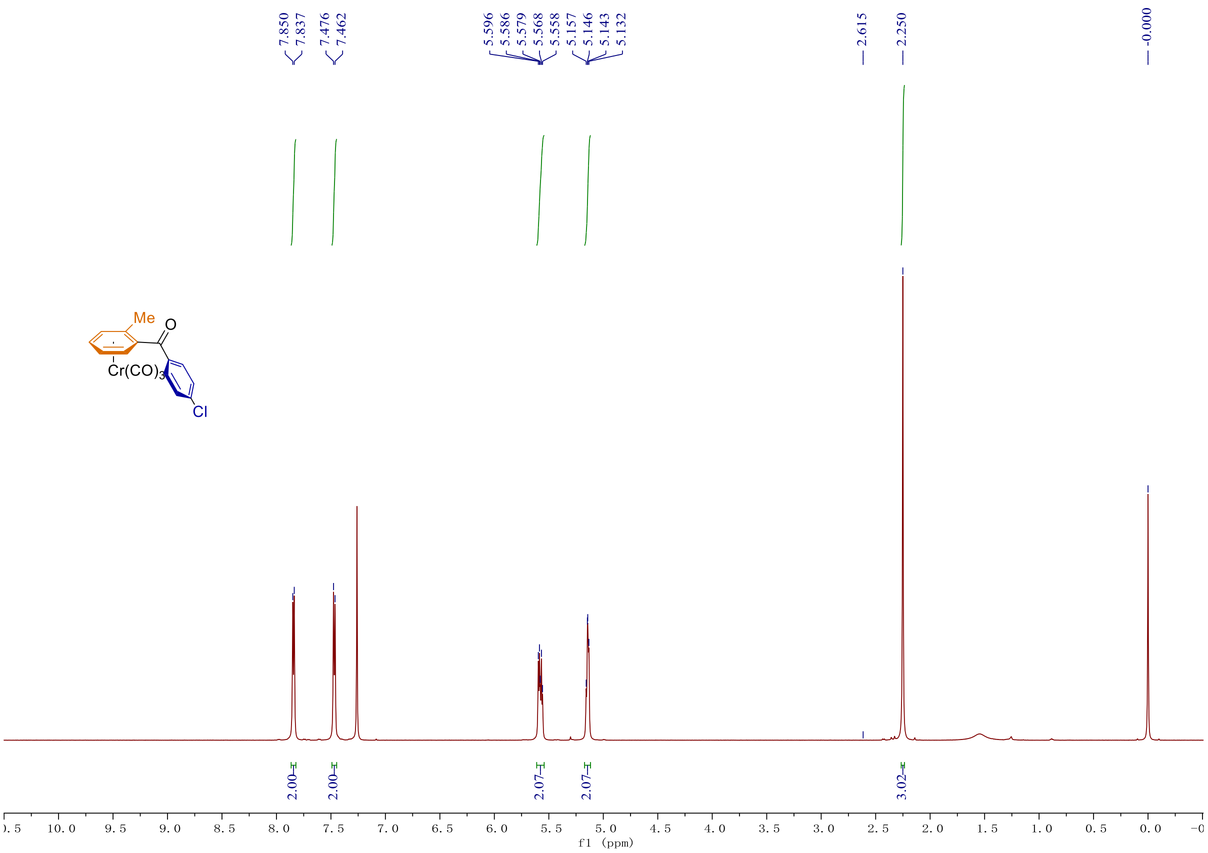

Supplementary Fig. 112 ^{1}H NMR (600 MHz, Chloroform-*d*) of 2-(4-chlorobenzoyl)toluene chromium tricarbonyl (1x-Cr).

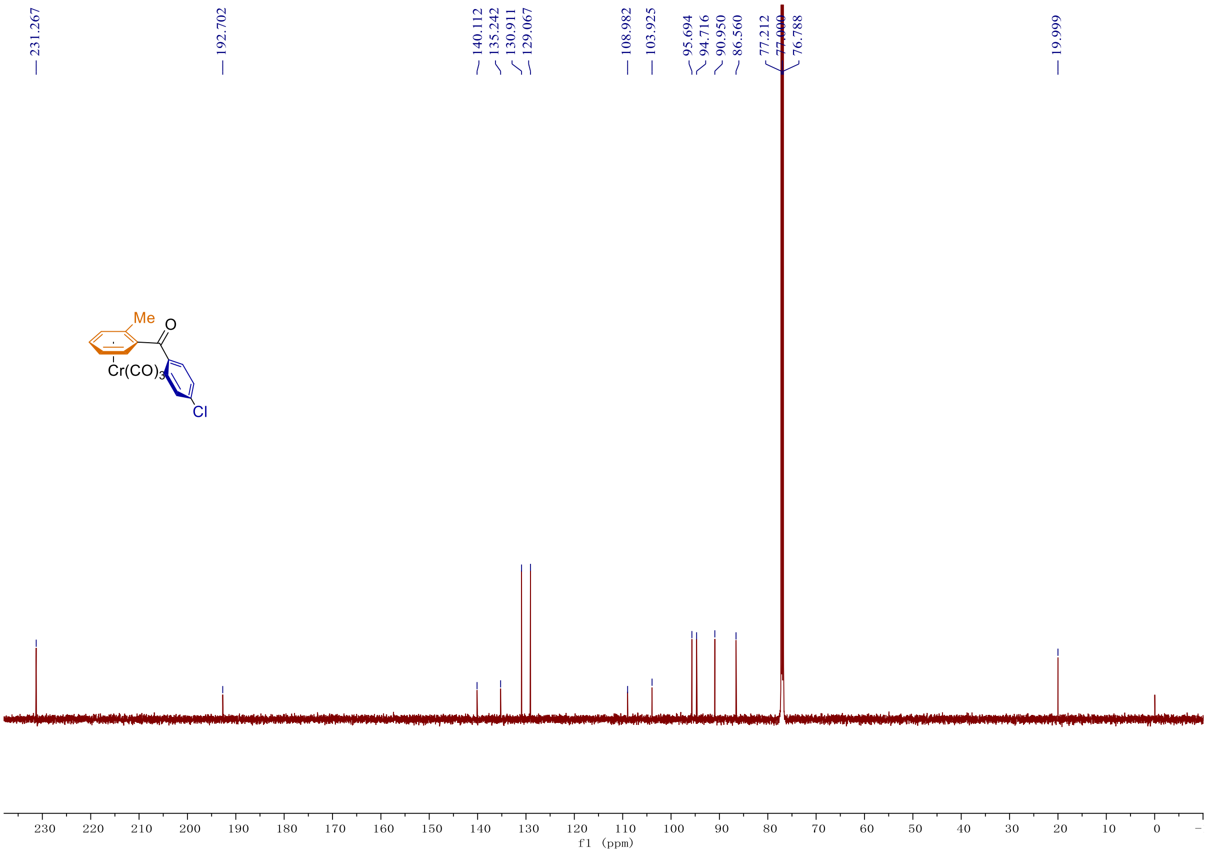

Supplementary Fig. 113 ^{13}C NMR (151 MHz, Chloroform-*d*) of 2-(4-chlorobenzoyl)toluene chromium tricarbonyl (1x-Cr).

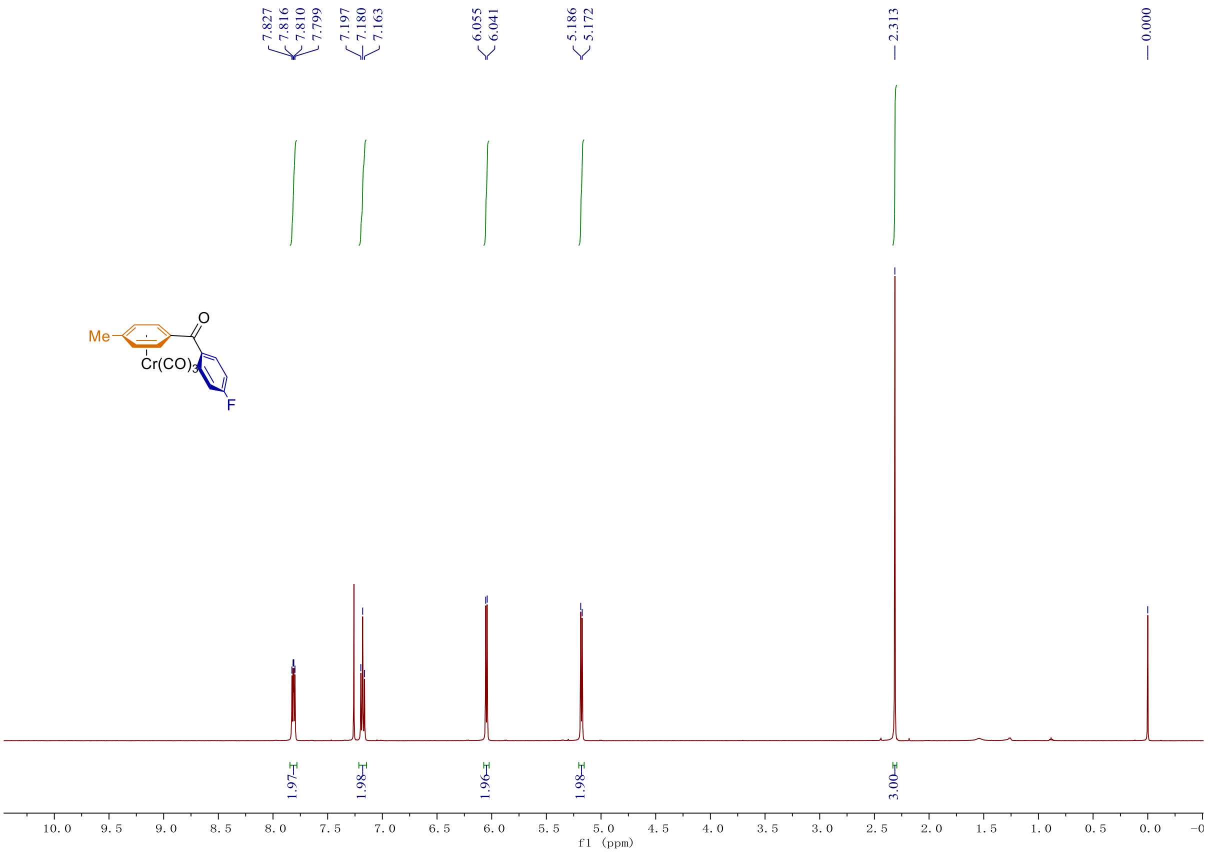

Supplementary Fig. 114 ^{1}H NMR (500 MHz, Chloroform-*d*) of 4-(4-fluorobenzoyl)toluene chromium tricarbonyl (1t-Cr).

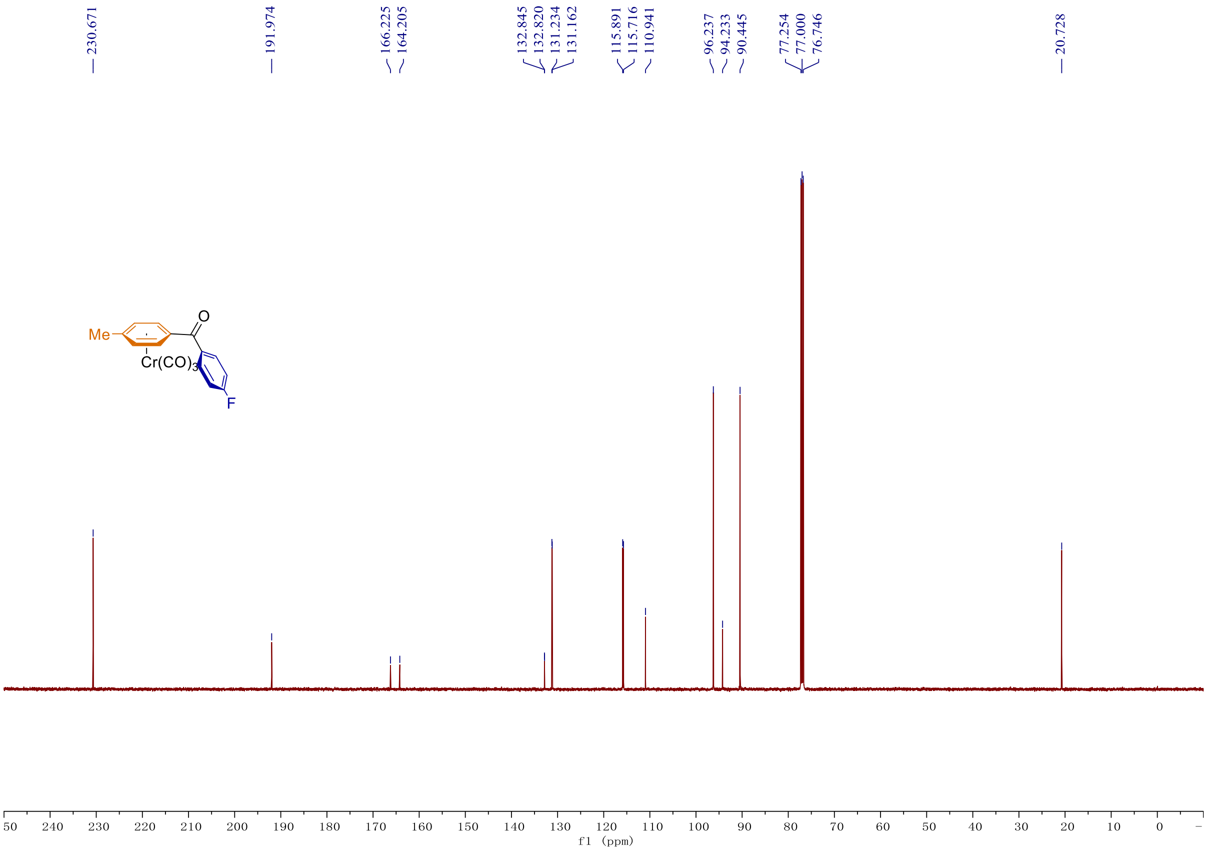

Supplementary Fig. 115 ^{13}C NMR (126 MHz, Chloroform-*d*) of 4-(4-fluorobenzoyl)toluene chromium tricarbonyl (1t-Cr).

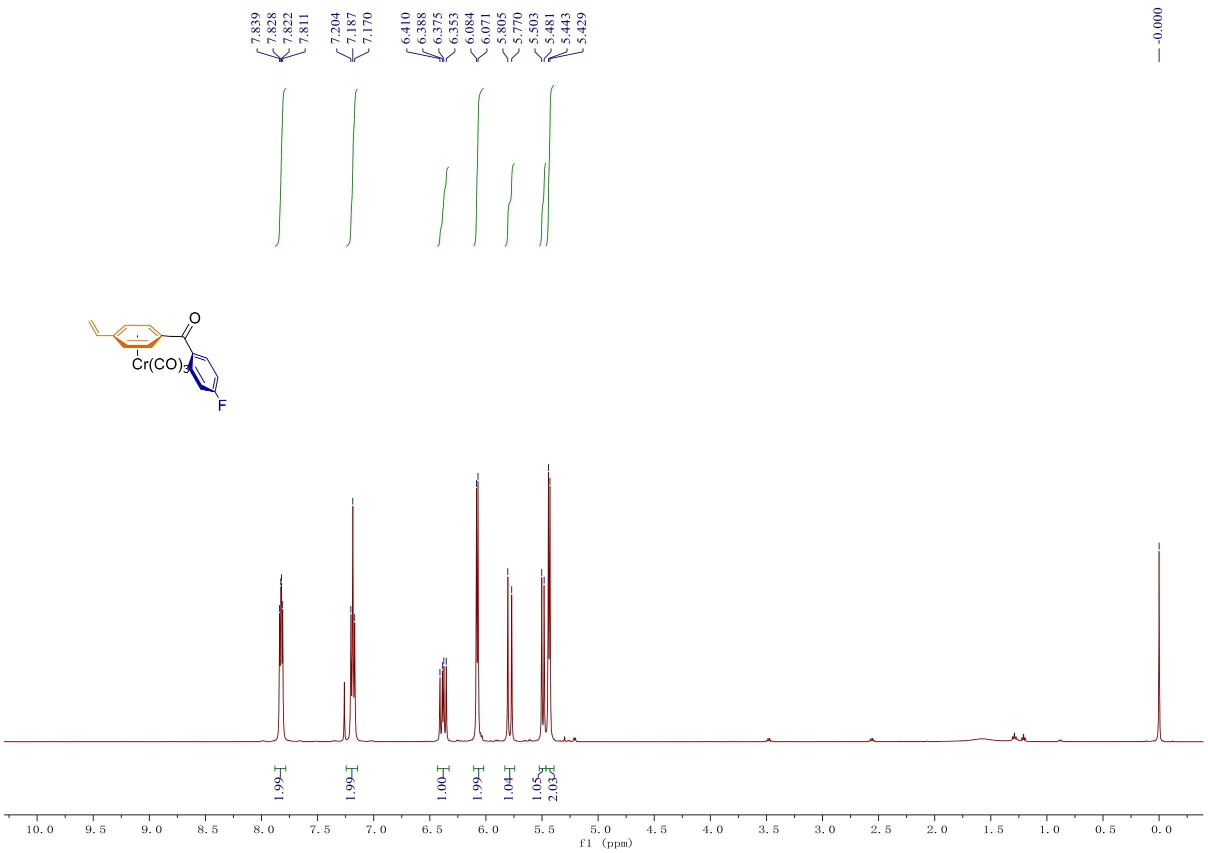

Supplementary Fig. 116 ^{1}H NMR (500 MHz, Chloroform-*d*) of 4-(4-fluorobenzoyl)styrene chromium tricarbonyl (1ac-Cr).

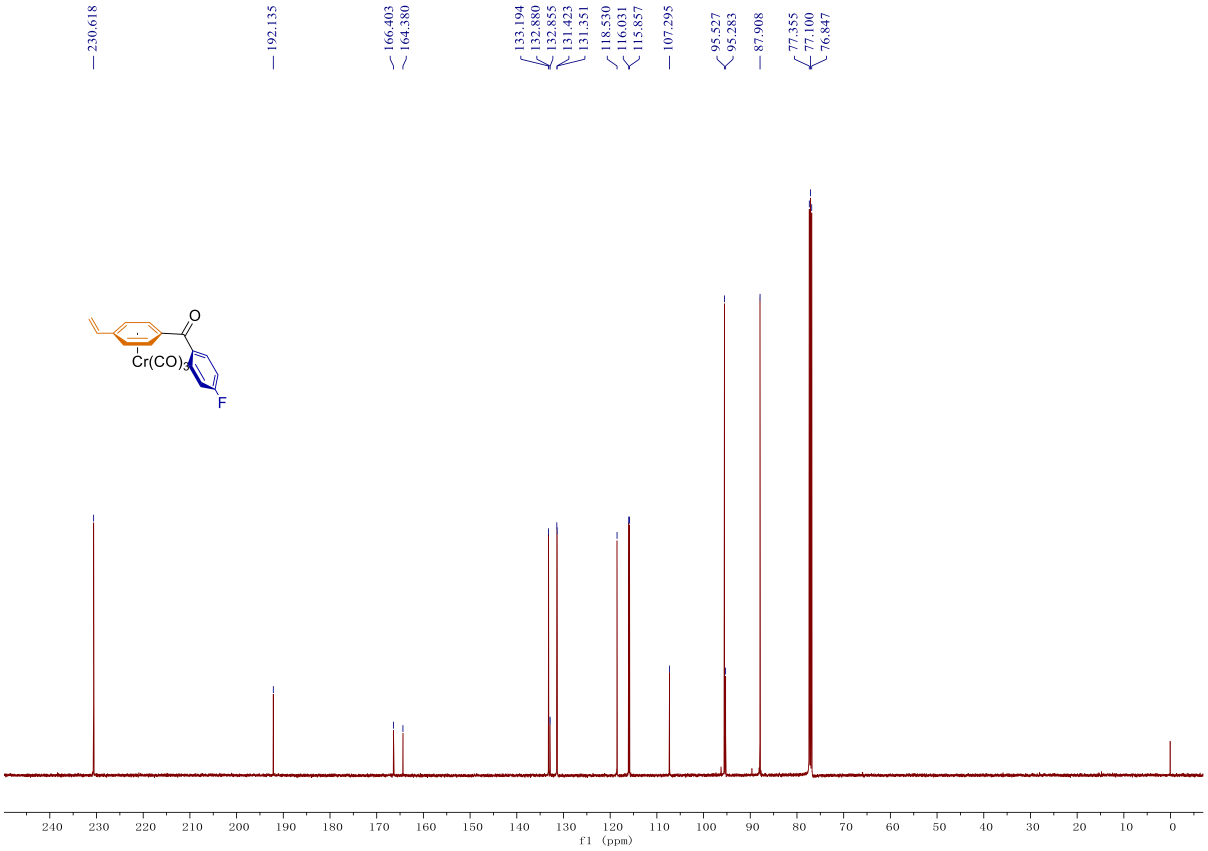

Supplementary Fig. 117 ^{13}C NMR (126 MHz, Chloroform-*d*) of 4-(4-fluorobenzoyl)styrene chromium tricarbonyl (1ac-Cr).

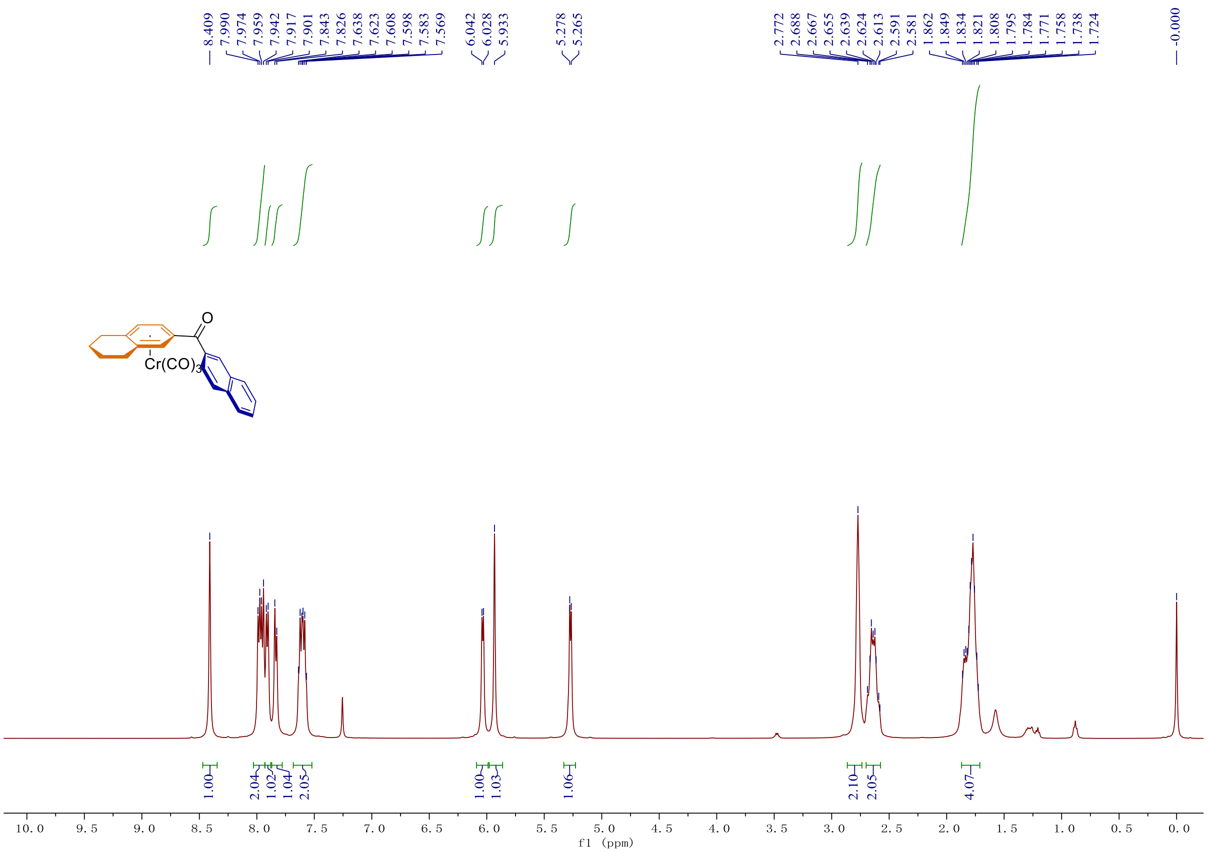

Supplementary Fig. 118 ^{1}H NMR (500 MHz, Chloroform-*d*) of 2-(2-naphthoyl)(5,6,7,8-tetrahydronaphthalene) chromium tricarbonyl (1w-Cr).

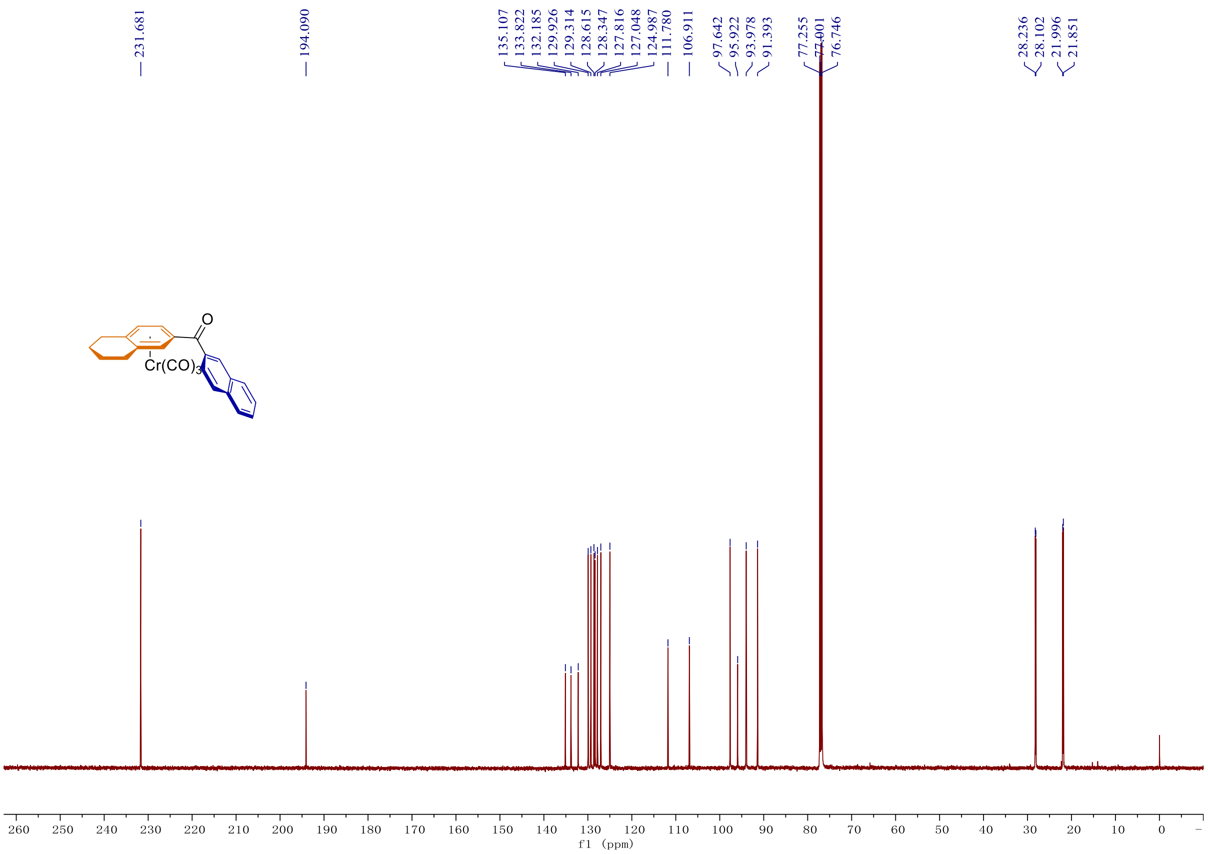

Supplementary Fig. 119 ^{13}C NMR (126 MHz, Chloroform-*d*) of 2-(2-naphthoyl)(5,6,7,8-tetrahydronaphthalene) chromium tricarbonyl (1w-Cr).

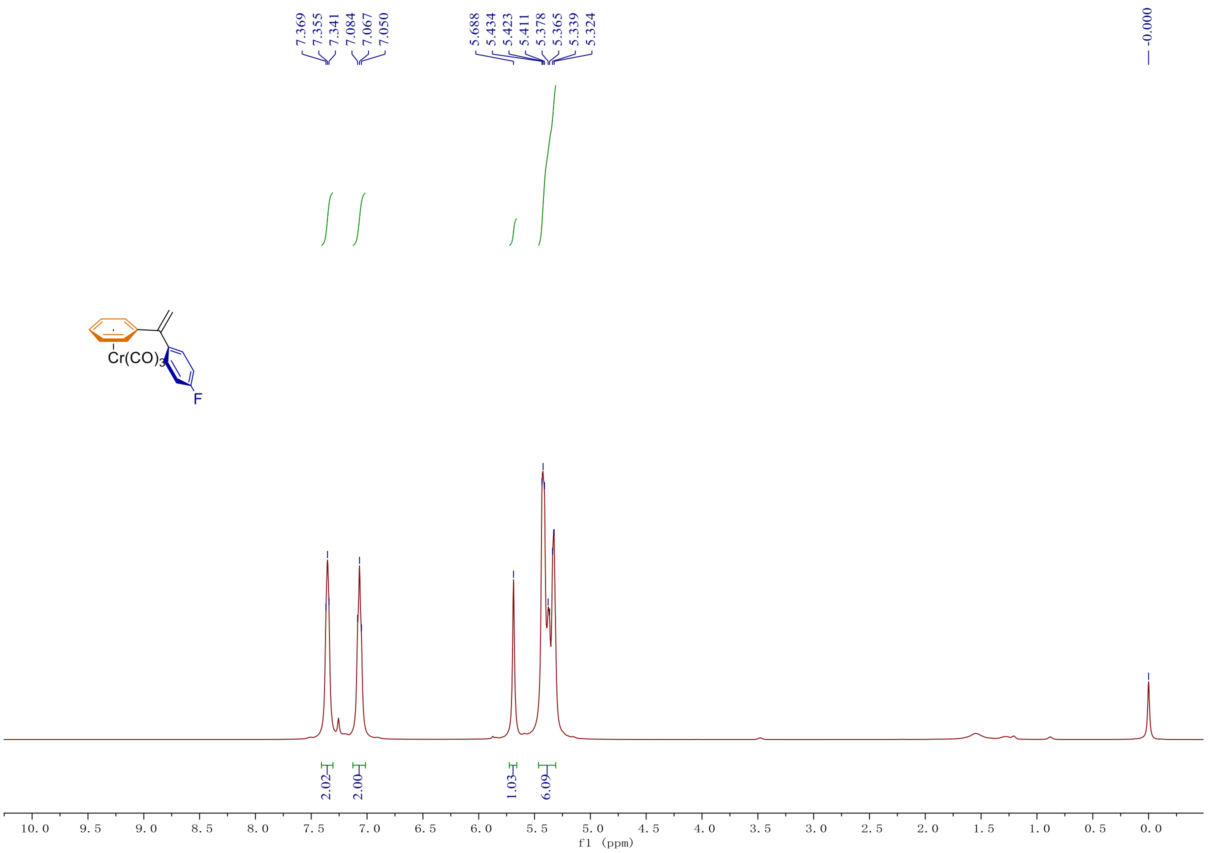

Supplementary Fig. 120 ^{1}H NMR (500 MHz, Chloroform-*d*) of (1-(4-fluorophenyl)vinyl)benzene chromium tricarbonyl (3a-Cr).

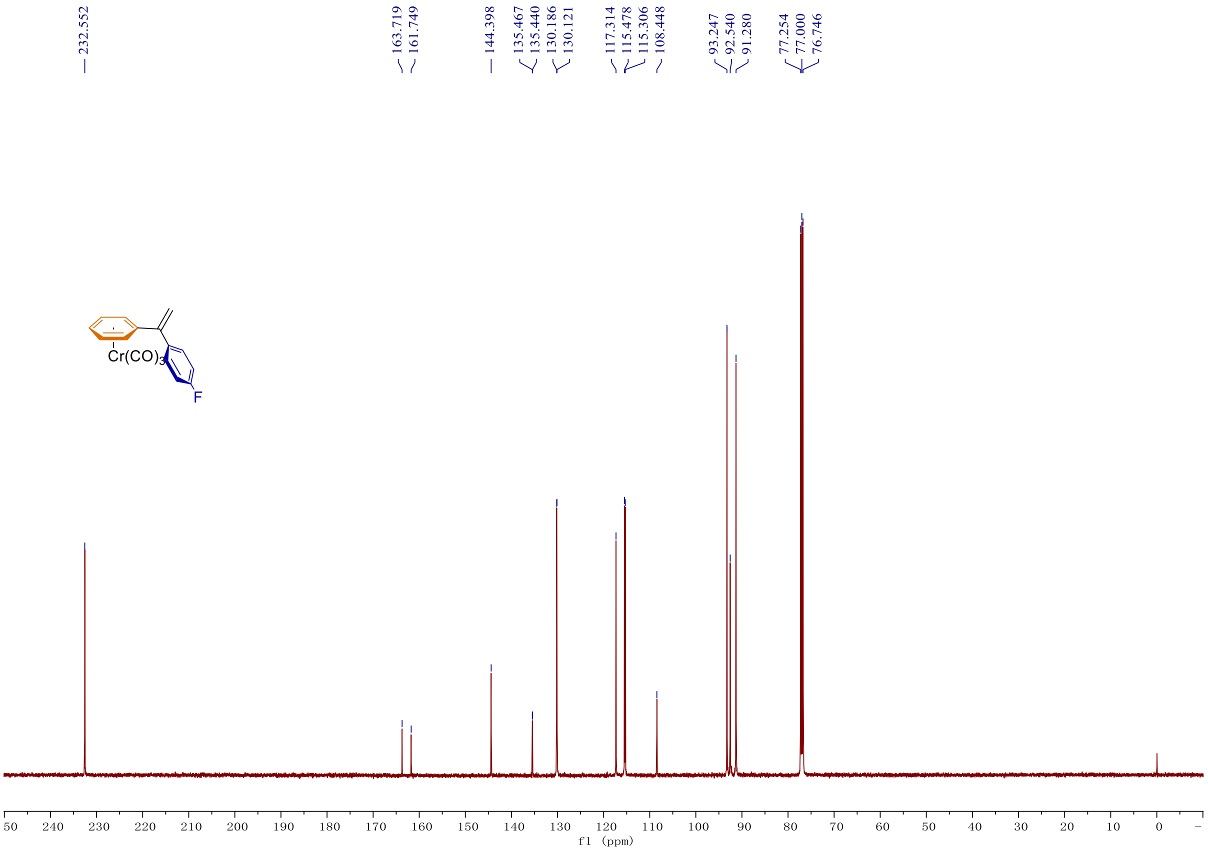

Supplementary Fig. 121 ^{13}C NMR (126 MHz, Chloroform-*d*) of (1-(4-fluorophenyl)vinyl)benzene chromium tricarbonyl (3a-Cr).

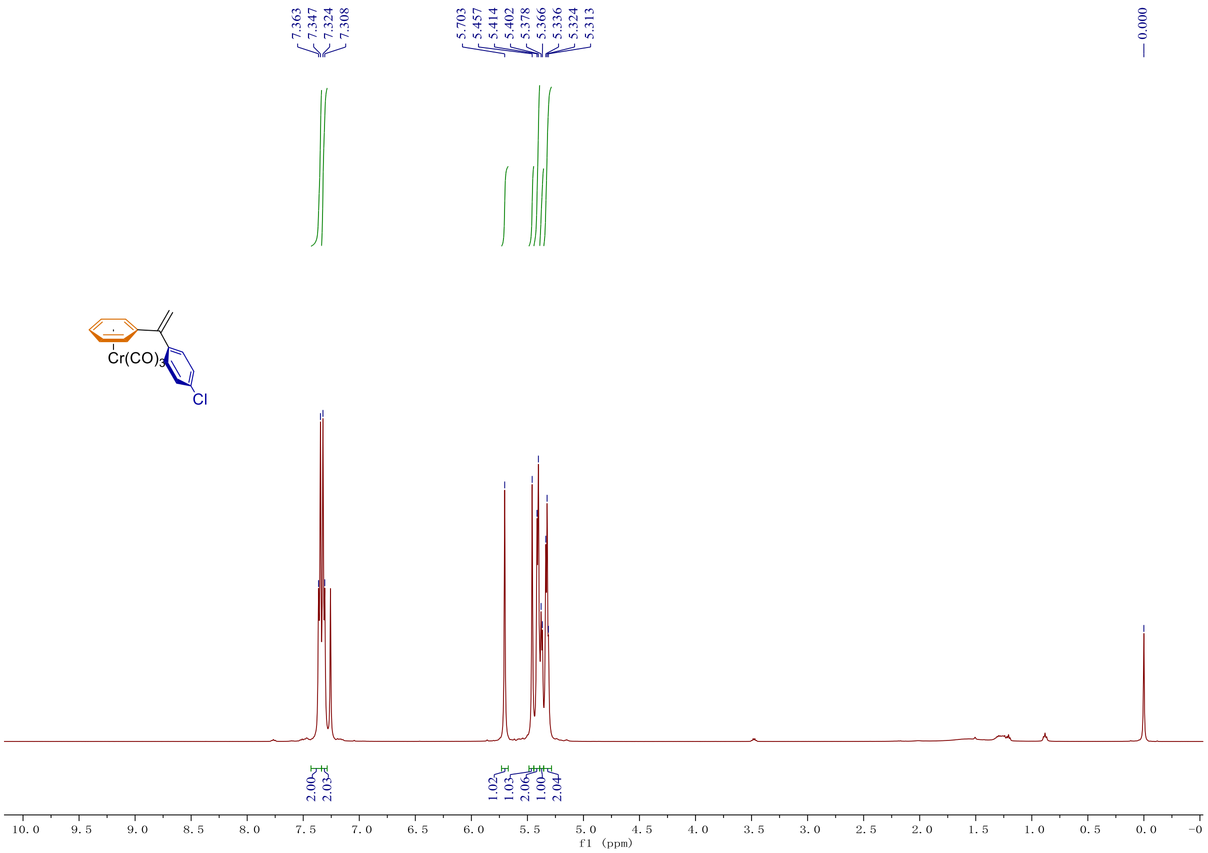

Supplementary Fig. 122 ^{1}H NMR (500 MHz, Chloroform-*d*) of (1-(4-chlorophenyl)vinyl)benzene chromium tricarbonyl (3b-Cr).

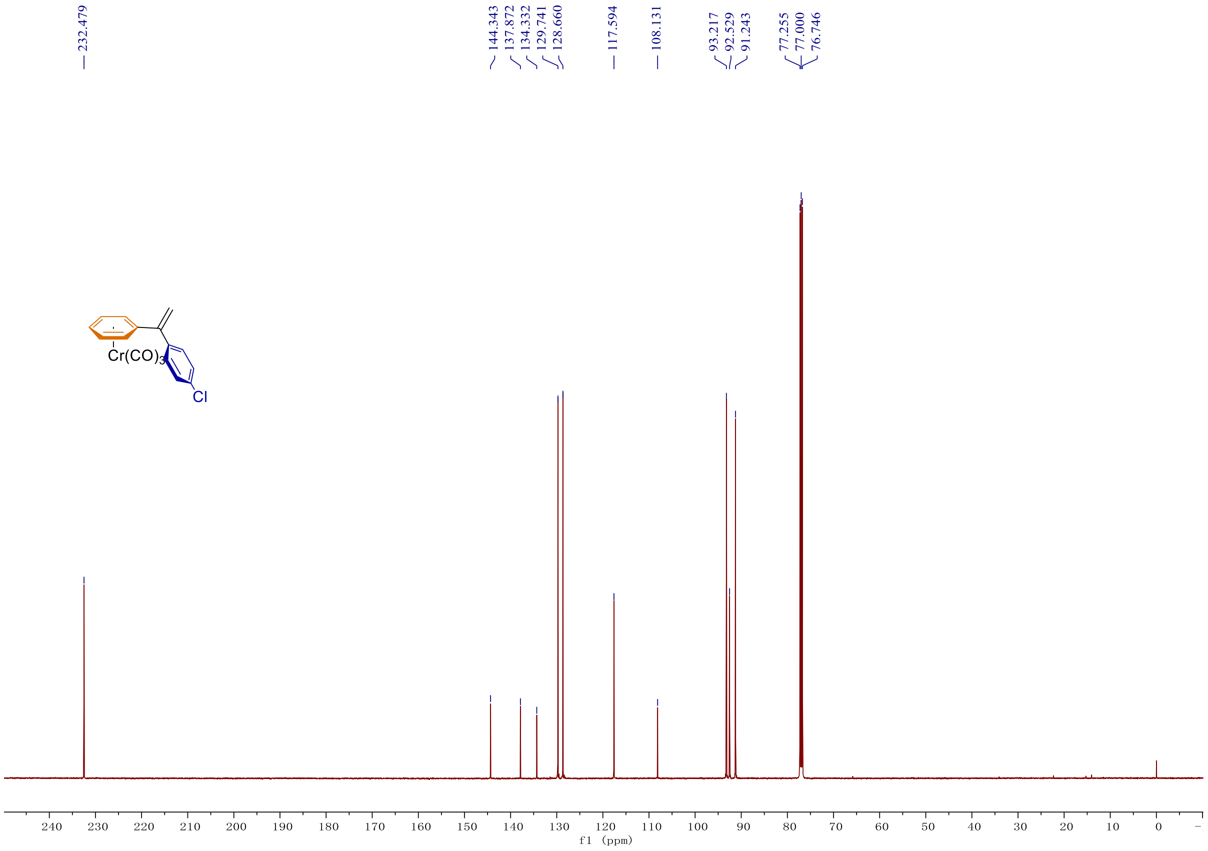

Supplementary Fig. 123 ^{13}C NMR (126 MHz, Chloroform-*d*) of (1-(4-chlorophenyl)vinyl)benzene chromium tricarbonyl (3b-Cr).

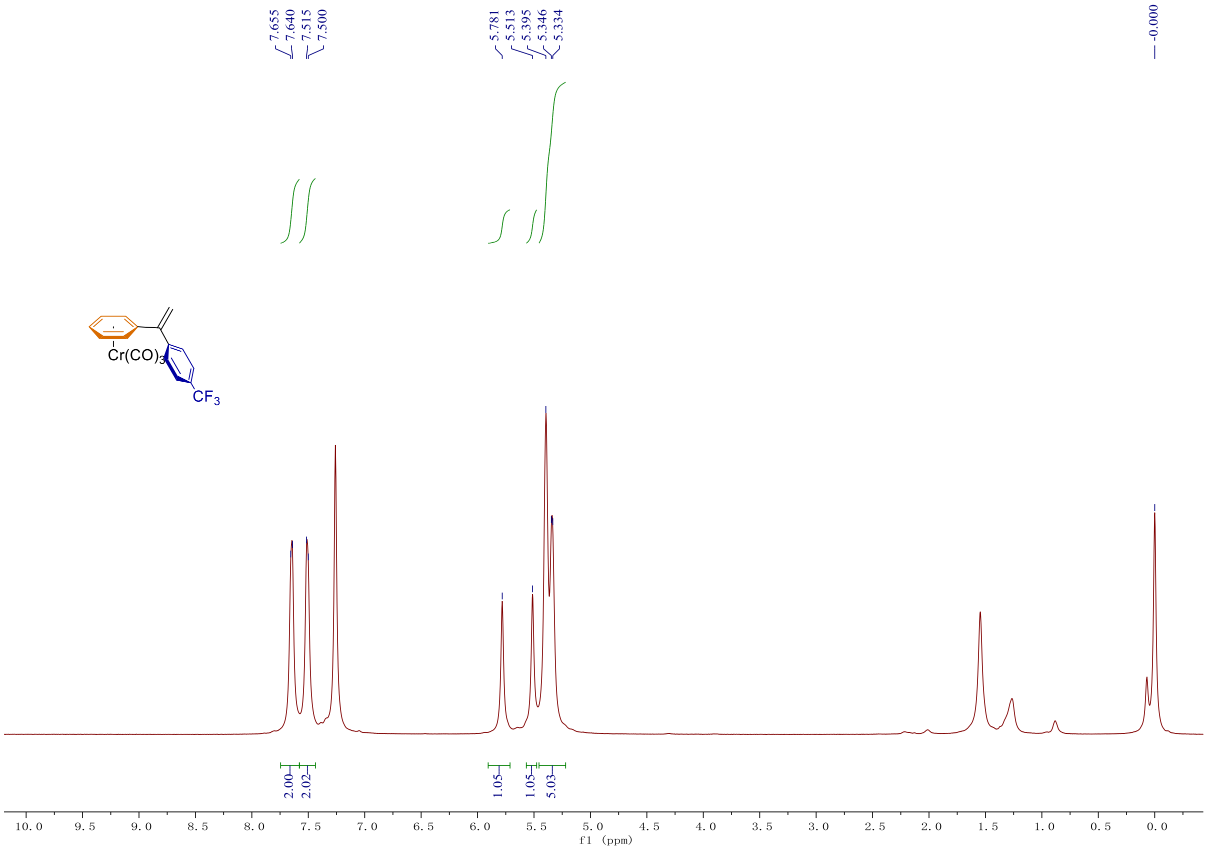

Supplementary Fig. 124 ^{1}H NMR (500 MHz, Chloroform-*d*) of (1-(4-(trifluoromethyl)phenyl)vinyl)benzene chromium tricarbonyl (3c-Cr).

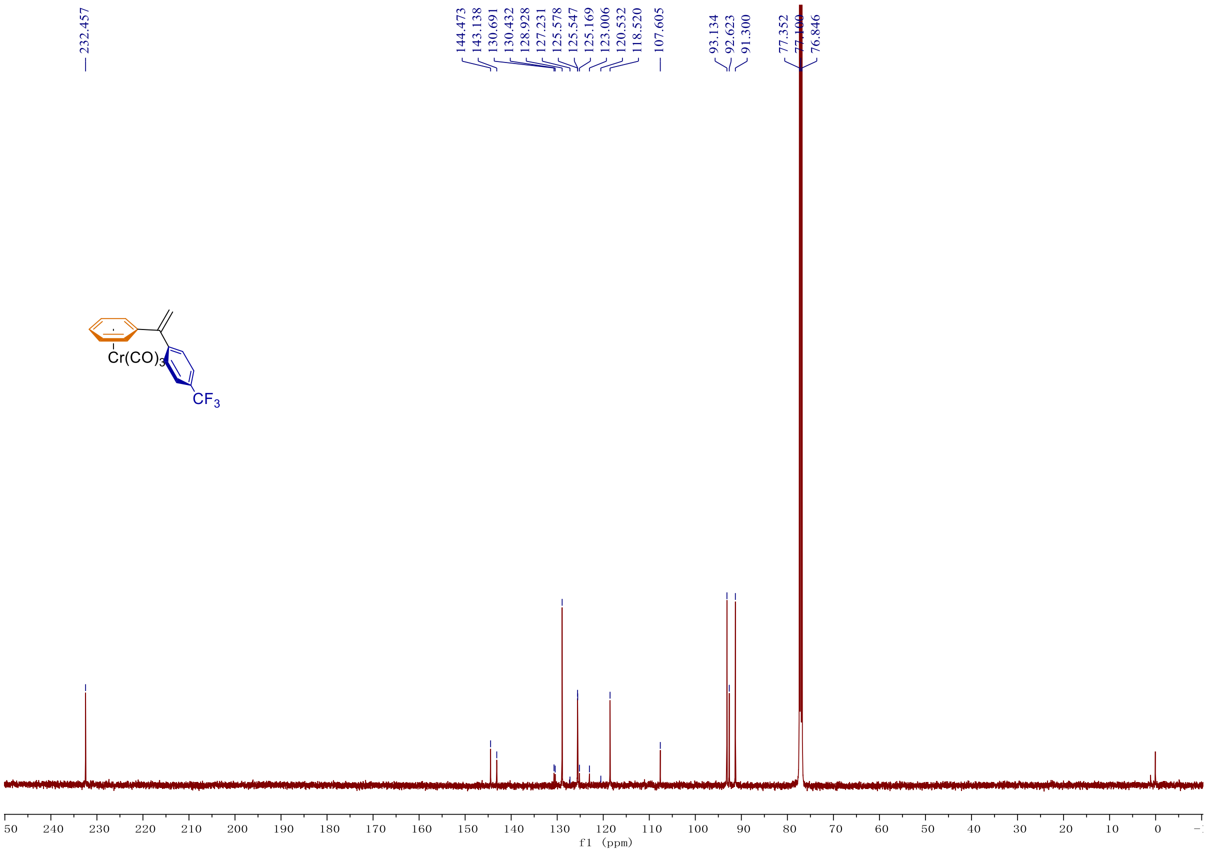

Supplementary Fig. 125 ^{13}C NMR (126 MHz, Chloroform-*d*) of (1-(4-(trifluoromethyl)phenyl)vinyl)benzene chromium tricarbonyl (3c-Cr).

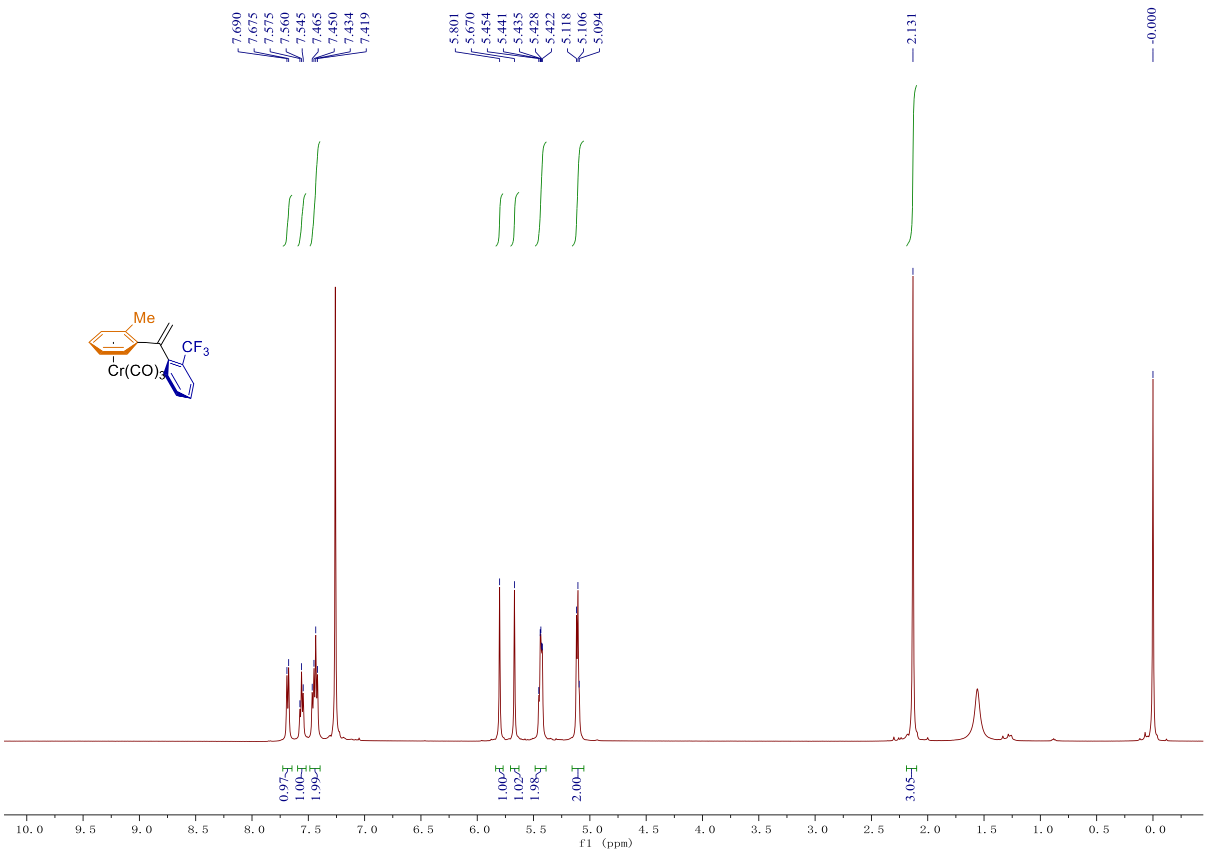

Supplementary Fig. 126 ^{1}H NMR (500 MHz, Chloroform-*d*) of 2-(1-(2-(trifluoromethyl)phenyl)vinyl)toluene chromium tricarbonyl (3d-Cr).

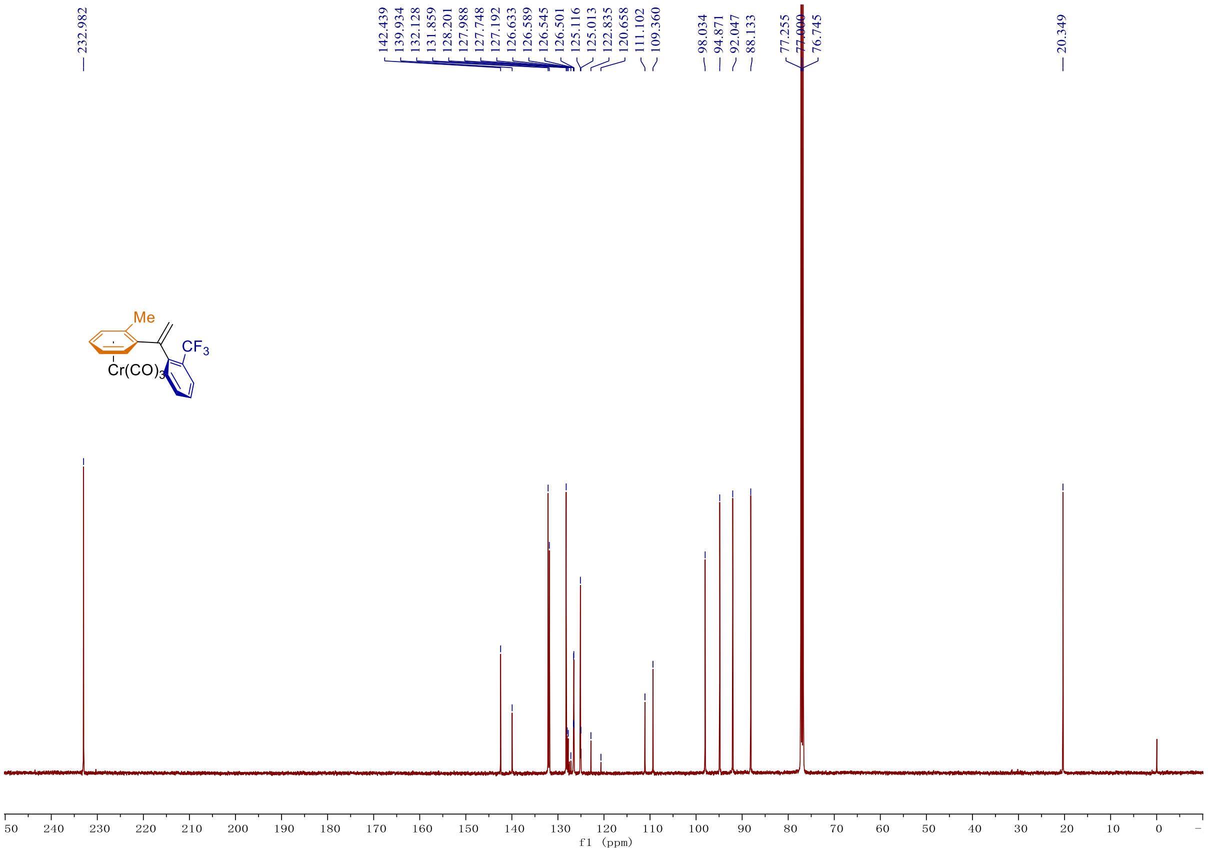

Supplementary Fig. 127 ^{13}C NMR (126 MHz, Chloroform-*d*) of 2-(1-(2-(trifluoromethyl)phenyl)vinyl)toluene chromium tricarbonyl (3d-Cr).

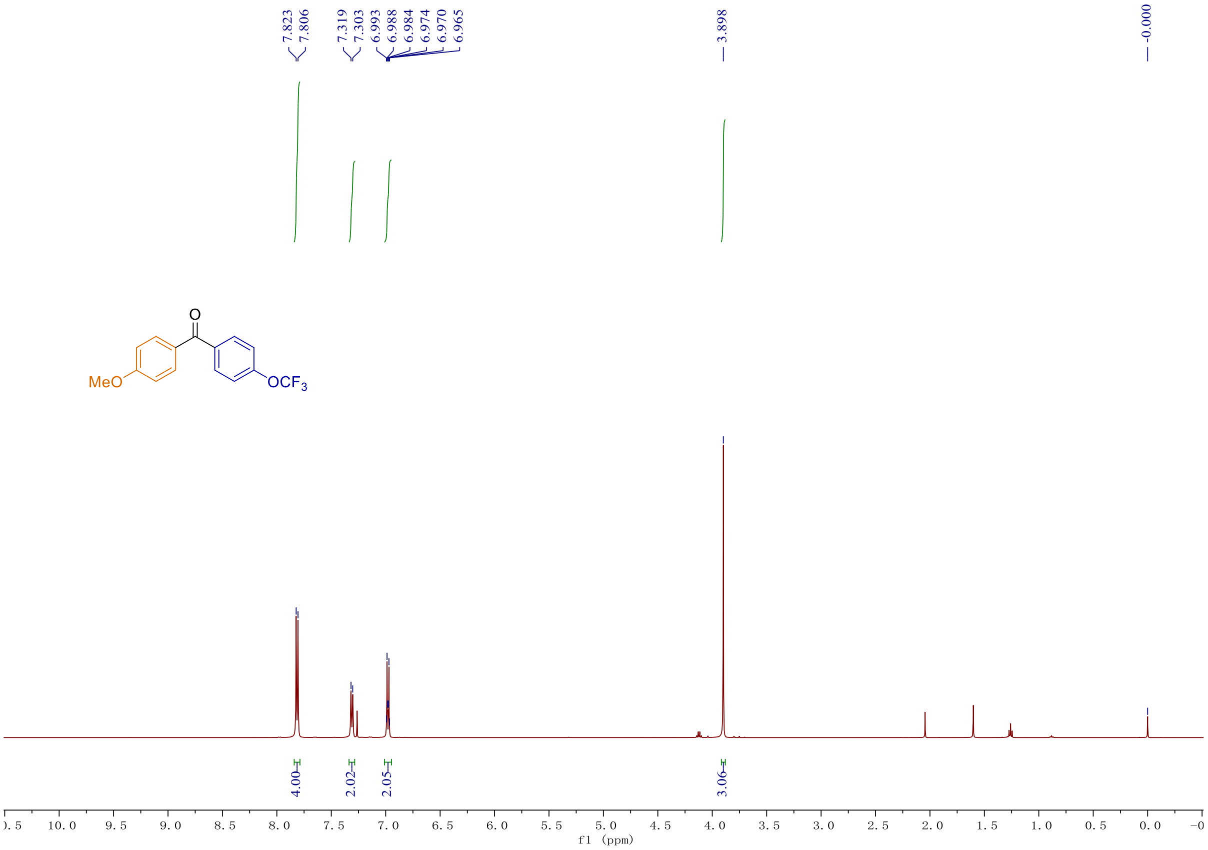

Supplementary Fig. 128 ^{1}H NMR (500 MHz, Chloroform-*d*) of (4-methoxyphenyl)(4-(trifluoromethoxy)phenyl)methanone (1p).

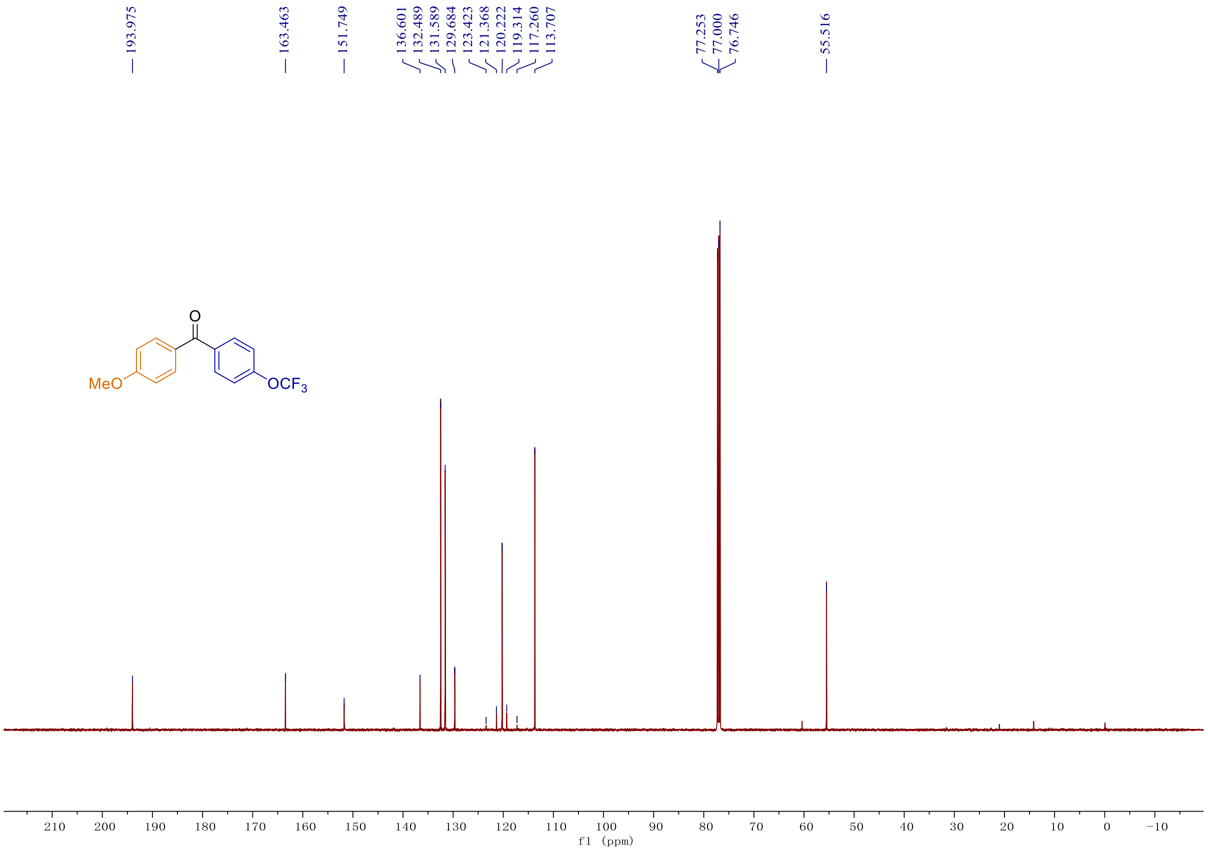

Supplementary Fig. 129 ^{13}C NMR (126 MHz, Chloroform-*d*) of (4-methoxyphenyl)(4-(trifluoromethoxy)phenyl)methanone (1p).

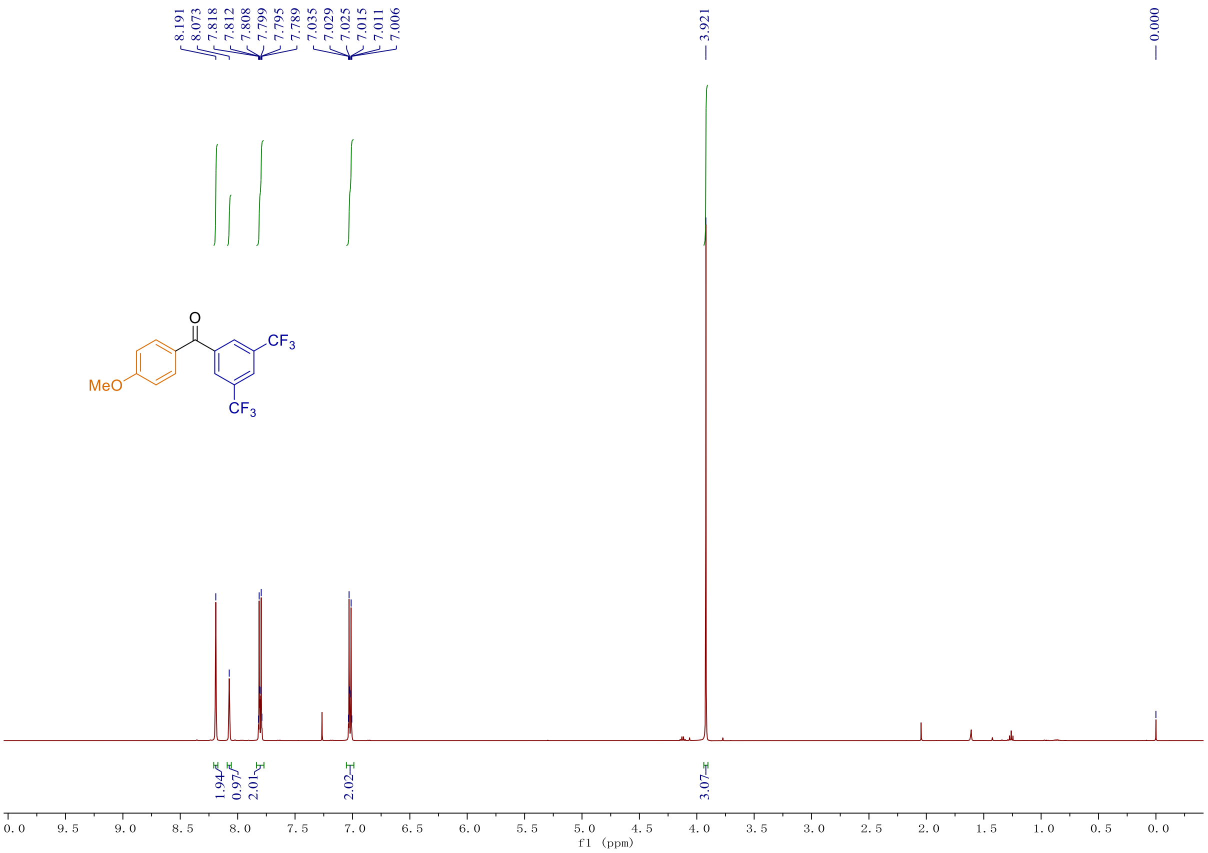

Supplementary Fig. 130 ^{1}H NMR (500 MHz, Chloroform-*d*) of (3,5-bis(trifluoromethyl)phenyl)(4-methoxyphenyl)methanone (s3).

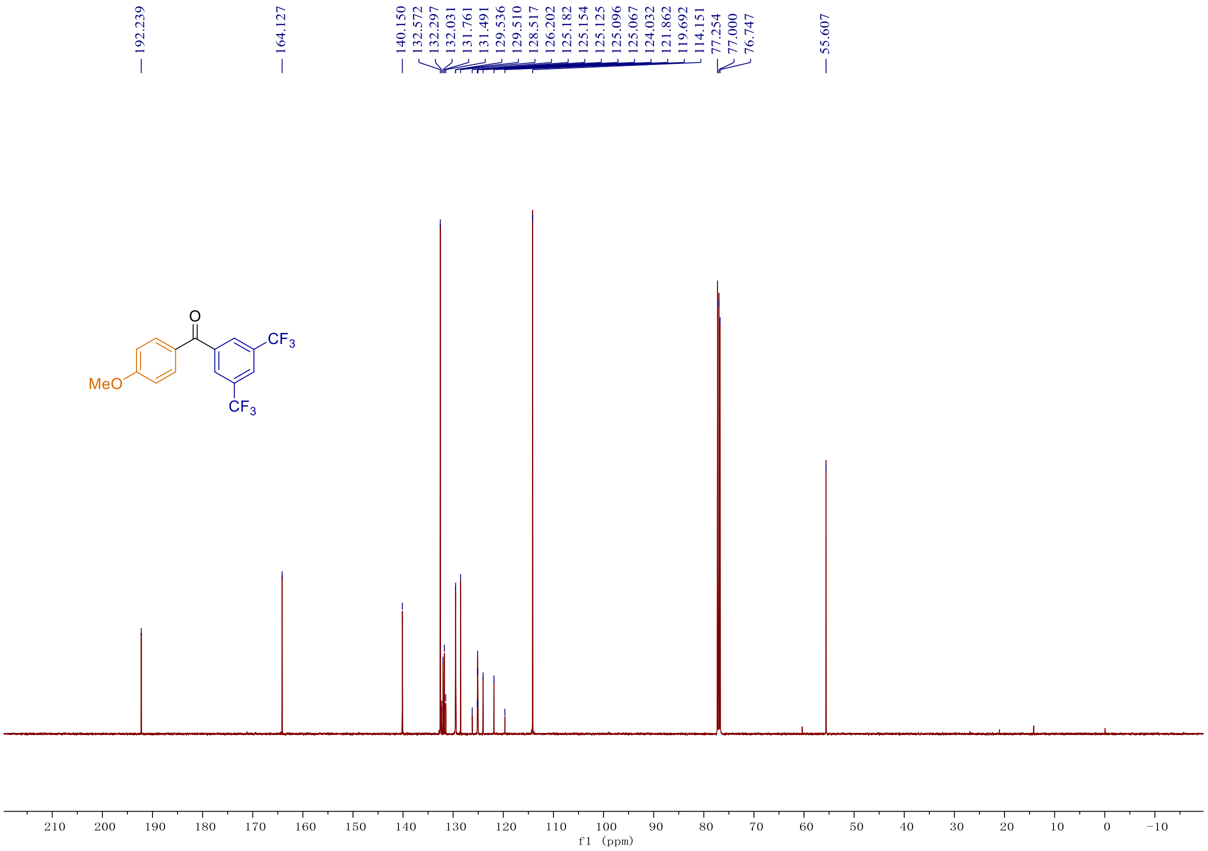

Supplementary Fig. 131 ^{13}C NMR (126 MHz, Chloroform-*d*) of (3,5-bis(trifluoromethyl)phenyl)(4-methoxyphenyl)methanone (s3).

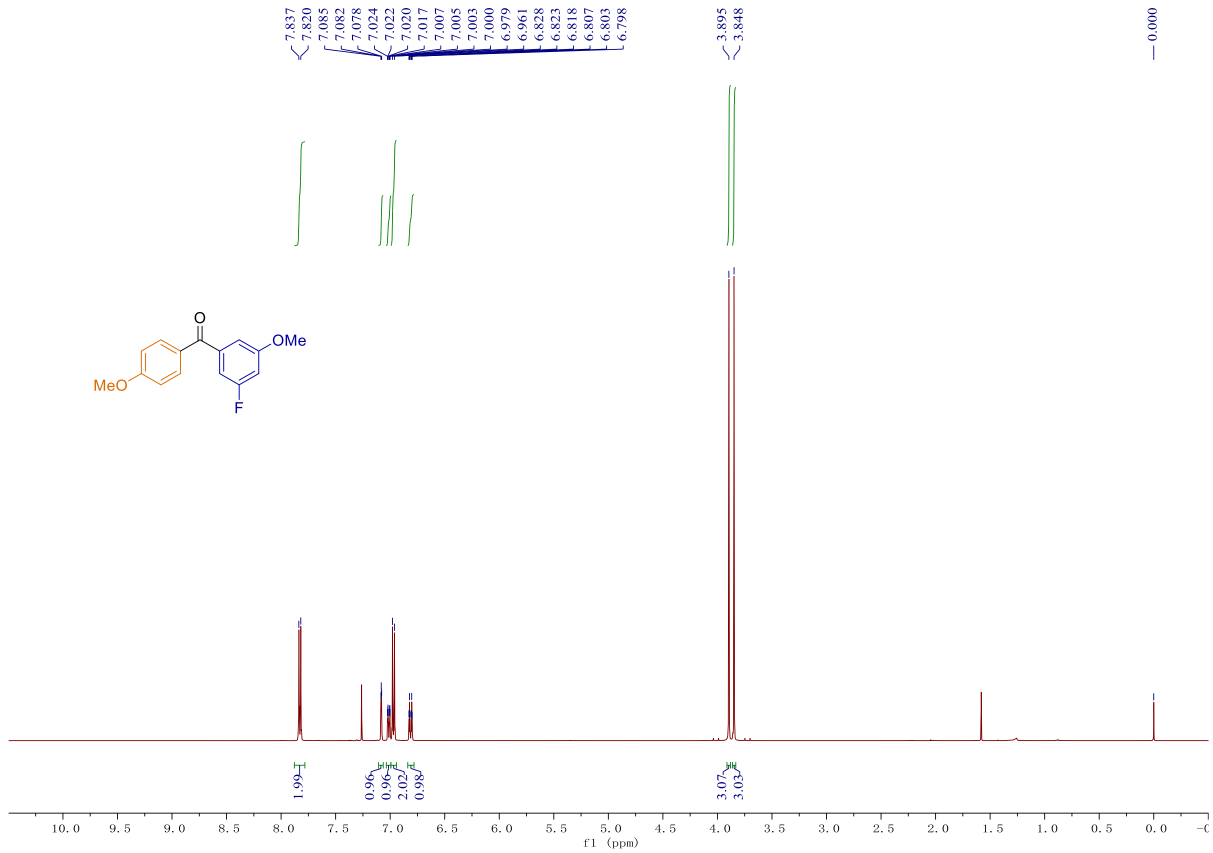

Supplementary Fig. 132 ^{1}H NMR (500 MHz, Chloroform-*d*) of (3-fluoro-5-methoxyphenyl)(4-methoxyphenyl)methanone (1v).

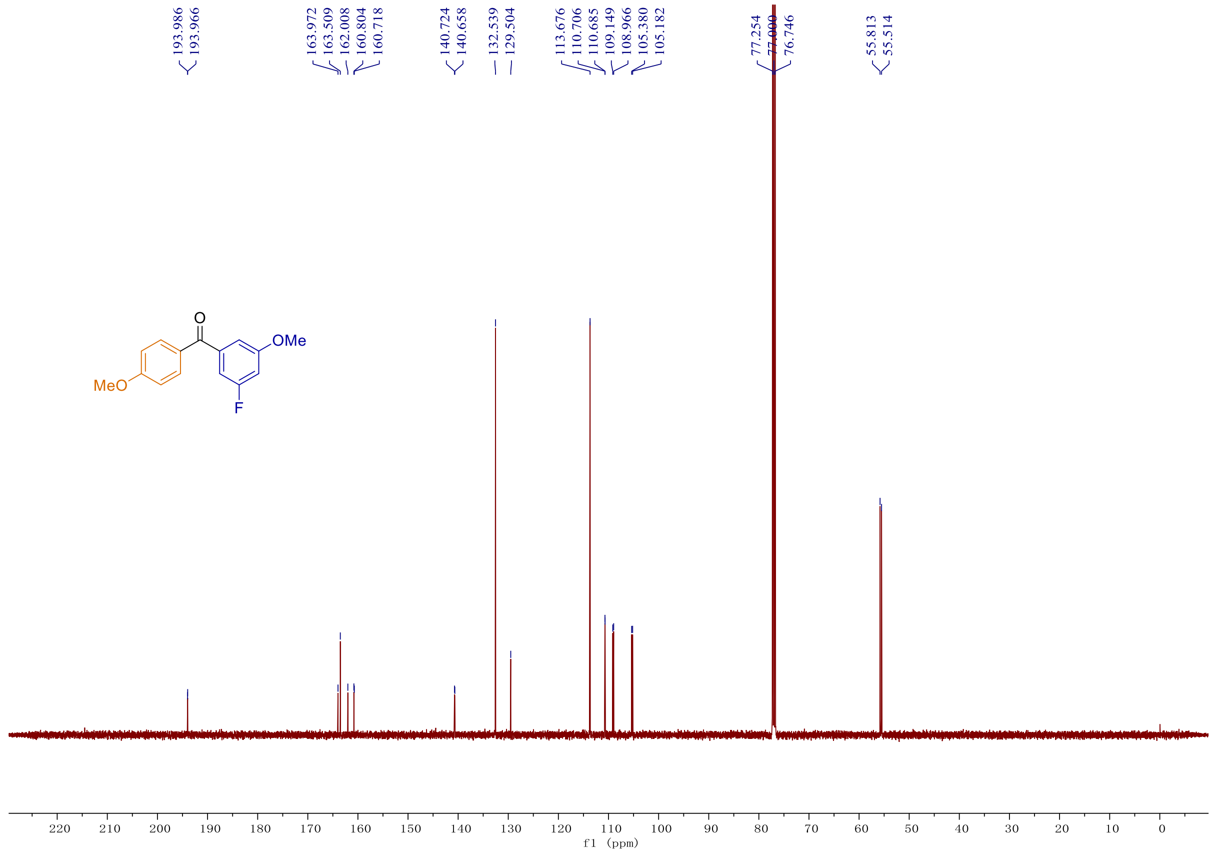

Supplementary Fig. 133 ^{13}C NMR (126 MHz, Chloroform-*d*) of (3-fluoro-5-methoxyphenyl)(4-methoxyphenyl)methanone (1v).

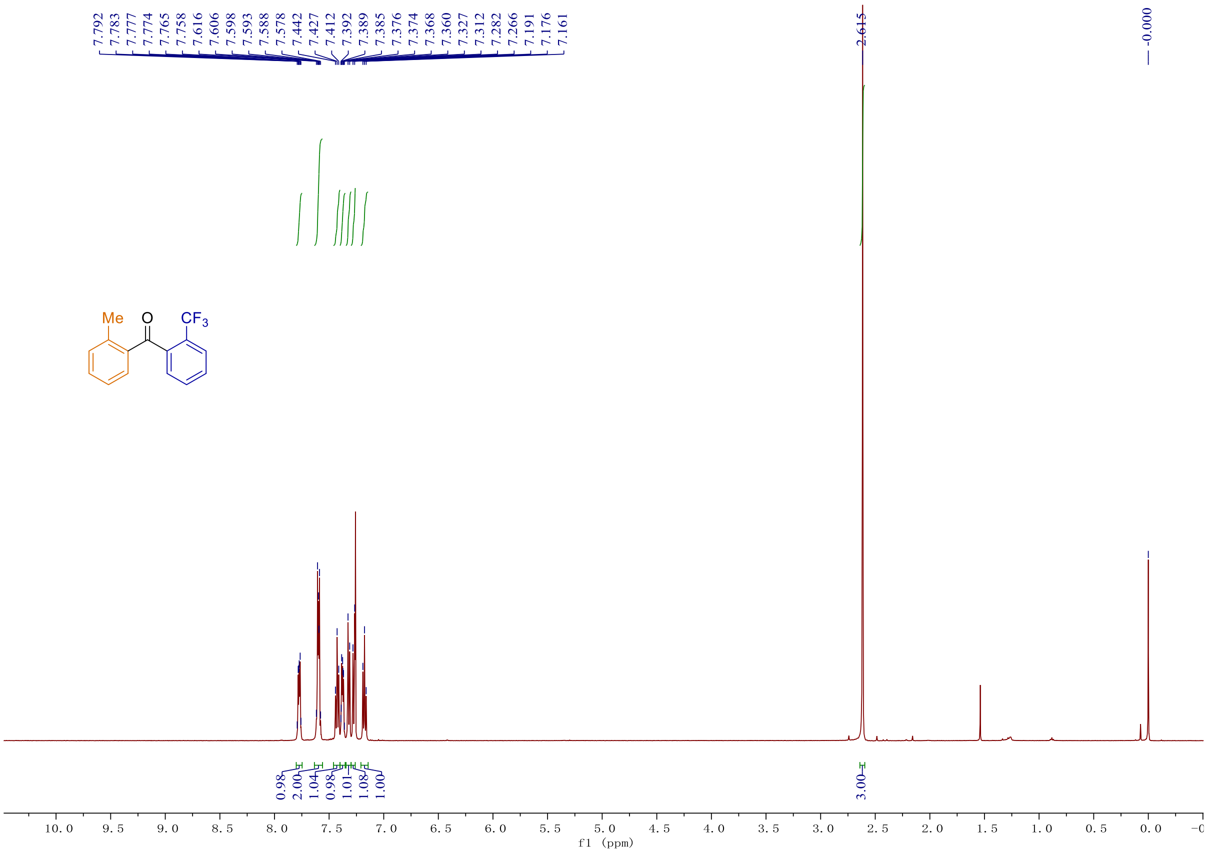

Supplementary Fig. 134 ^{1}H NMR (500 MHz, Chloroform-*d*) of *o*-tolyl(2-(trifluoromethyl)phenyl)methanone (1u).

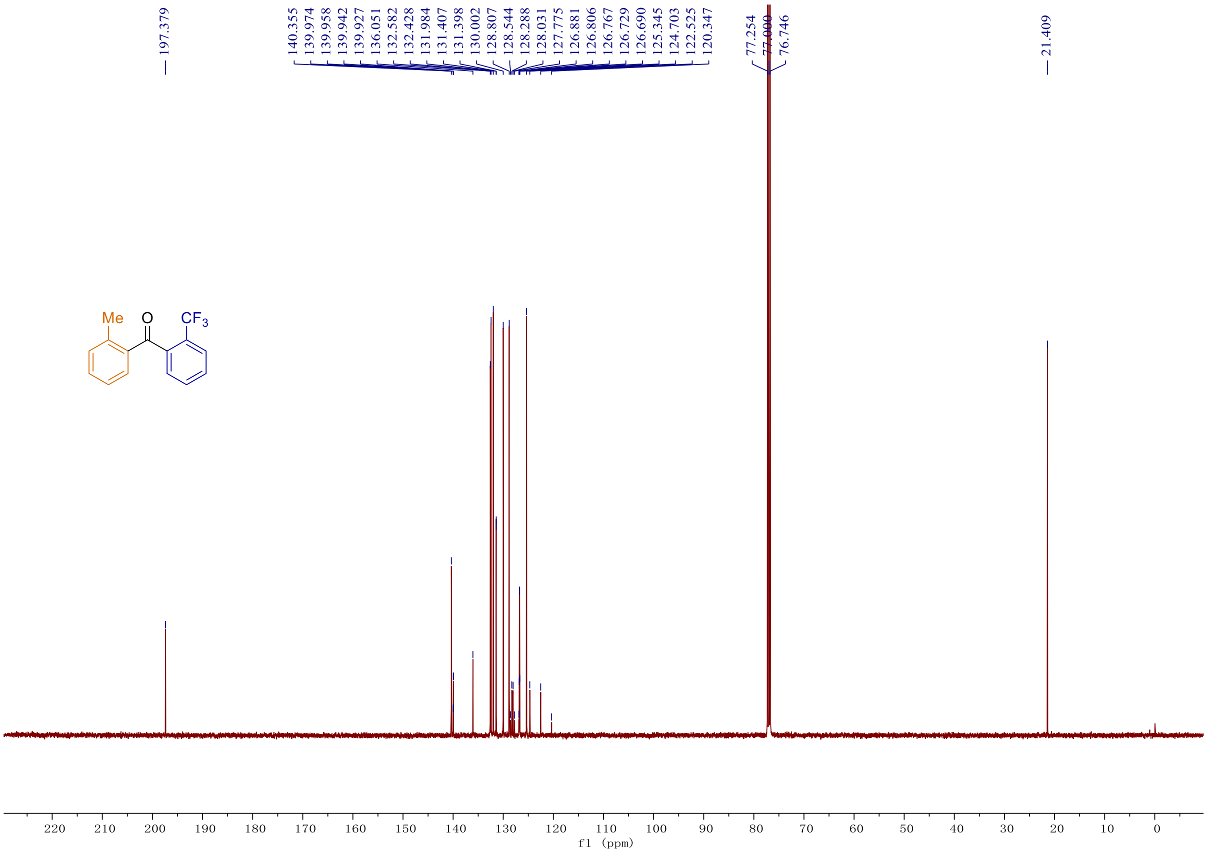

Supplementary Fig. 135 ^{13}C NMR (126 MHz, Chloroform-*d*) of *o*-tolyl(2-(trifluoromethyl)phenyl)methanone (1u).

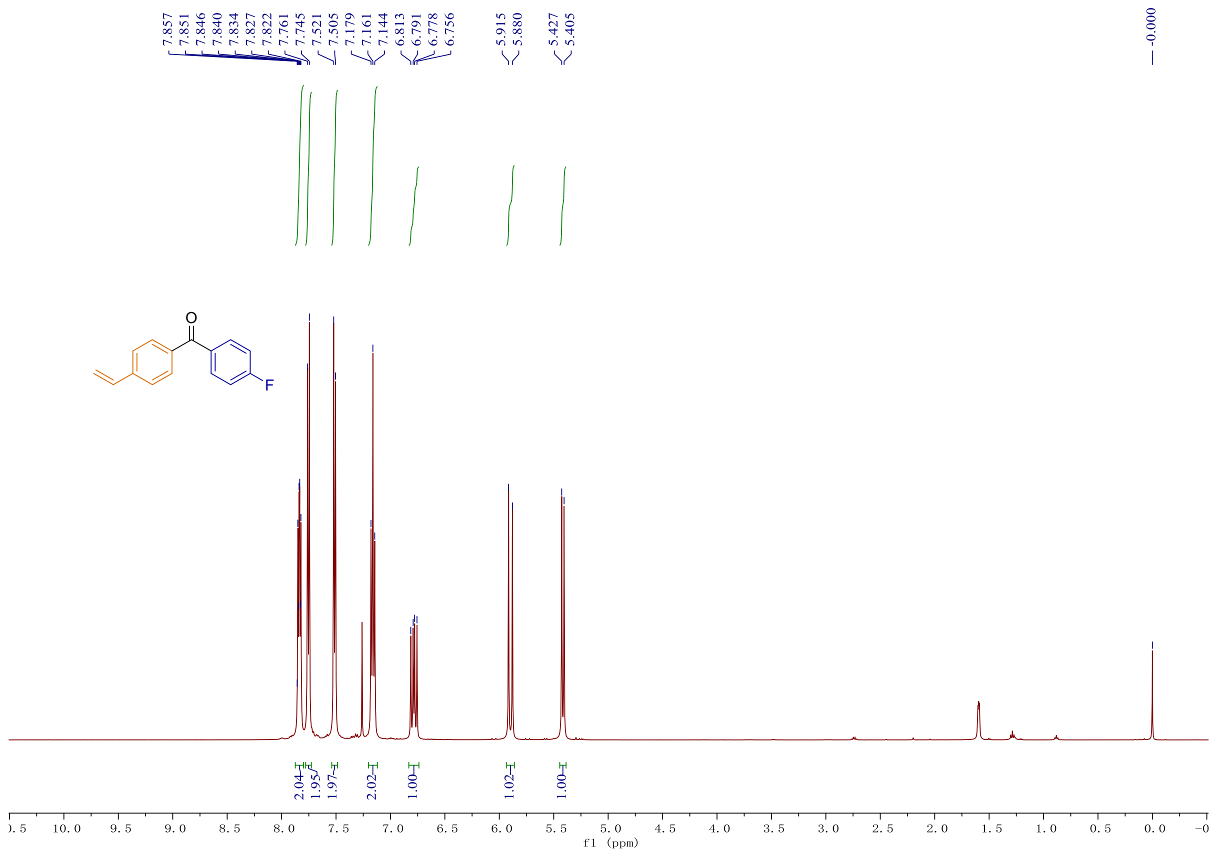

Supplementary Fig. 136 ^{1}H NMR (500 MHz, Chloroform-*d*) of (4-fluorophenyl)(4-vinylphenyl)methanone (1ac).

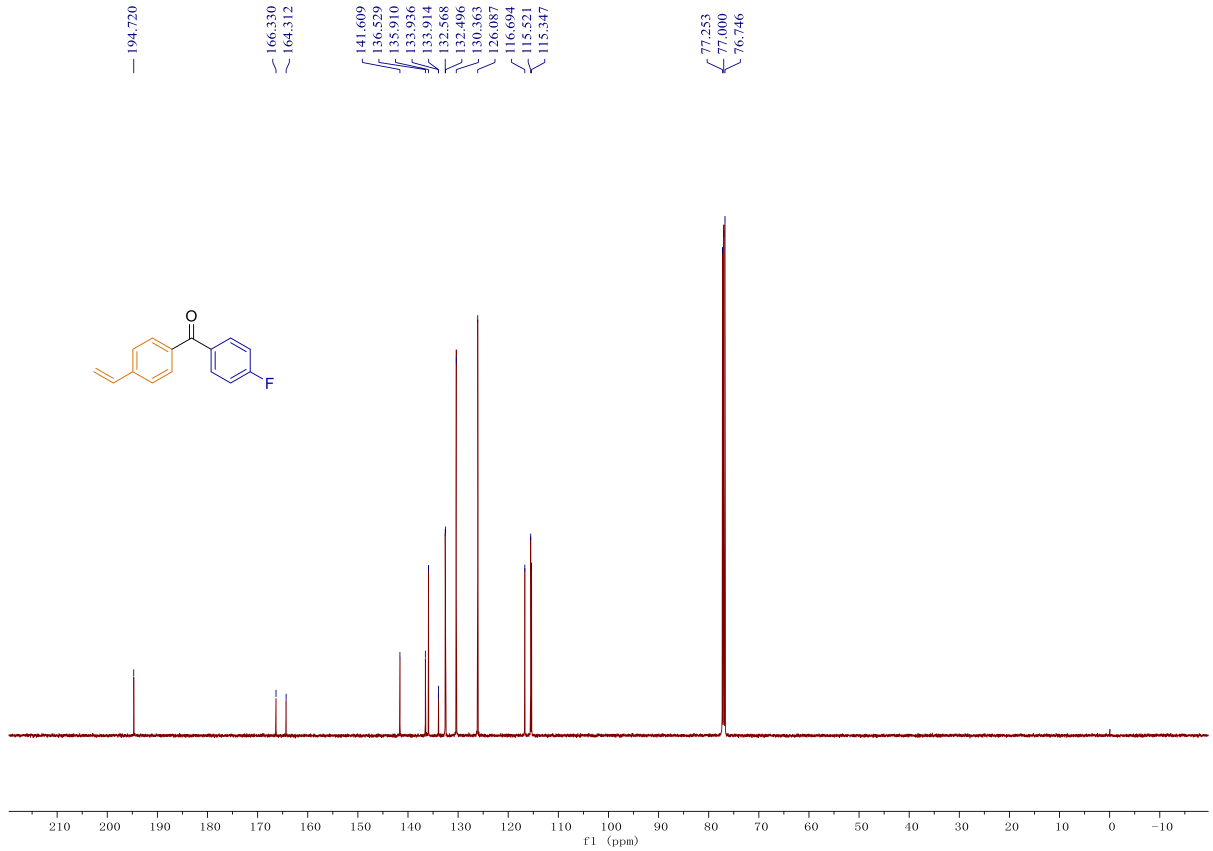

Supplementary Fig. 137 ^{13}C NMR (126 MHz, Chloroform-*d*) of (4-fluorophenyl)(4-vinylphenyl)methanone (1ac).

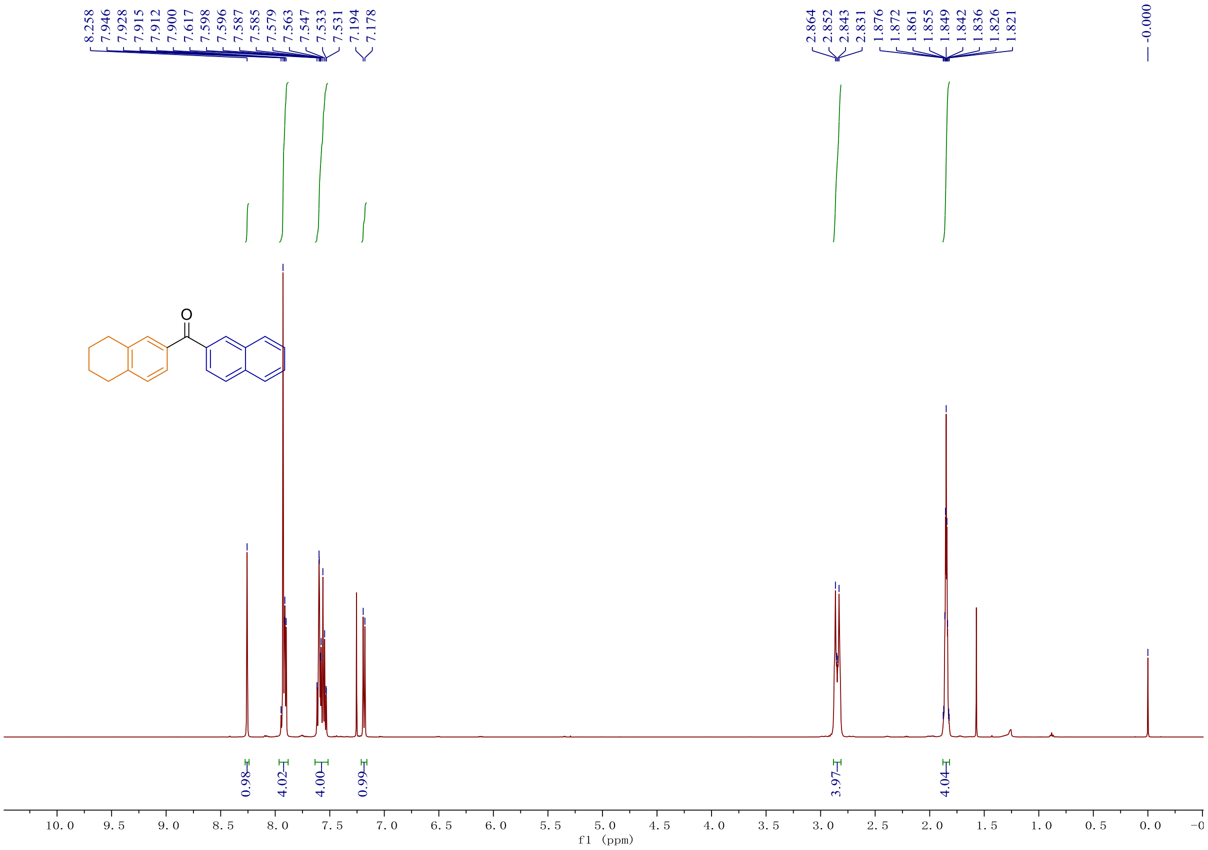

Supplementary Fig. 138 ^{1}H NMR (500 MHz, Chloroform-*d*) of naphthalen-2-yl(5,6,7,8-tetrahydronaphthalen-2-yl)methanone (1w).

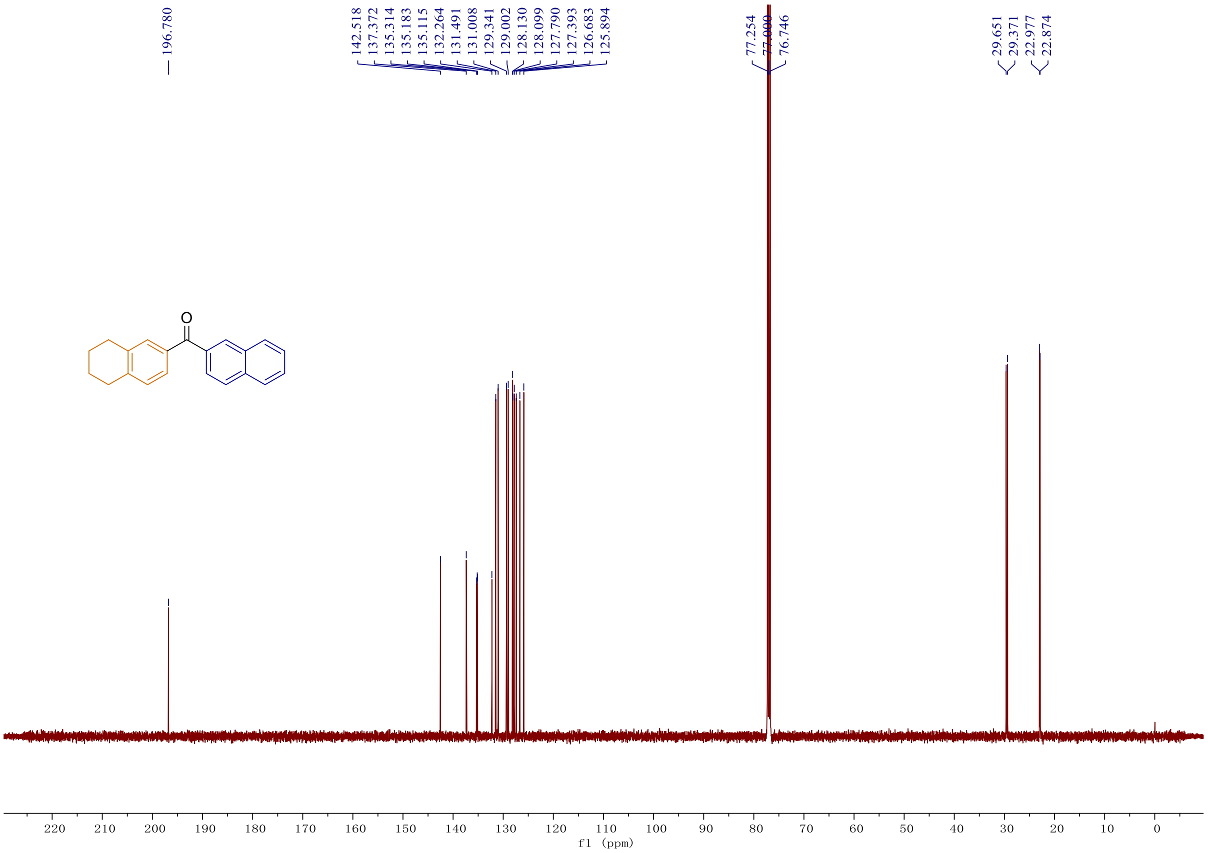

Supplementary Fig. 139 ^{13}C NMR (126 MHz, Chloroform-*d*) of naphthalen-2-yl(5,6,7,8-tetrahydronaphthalen-2-yl)methanone (1w).

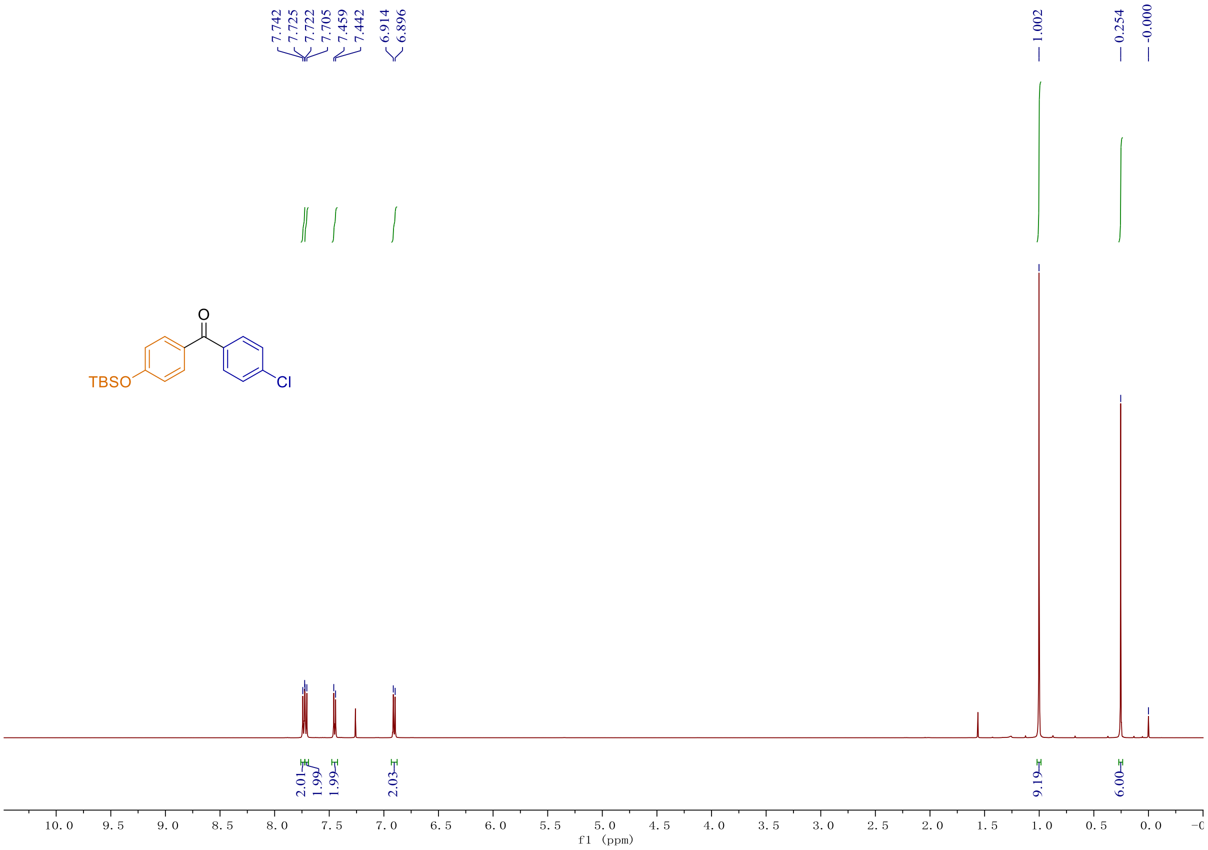

Supplementary Fig. 140 ^{1}H NMR (500 MHz, Chloroform-*d*) of (4-((tert-butyldimethylsilyl)oxy)phenyl)(4-chlorophenyl)methanone (1q).

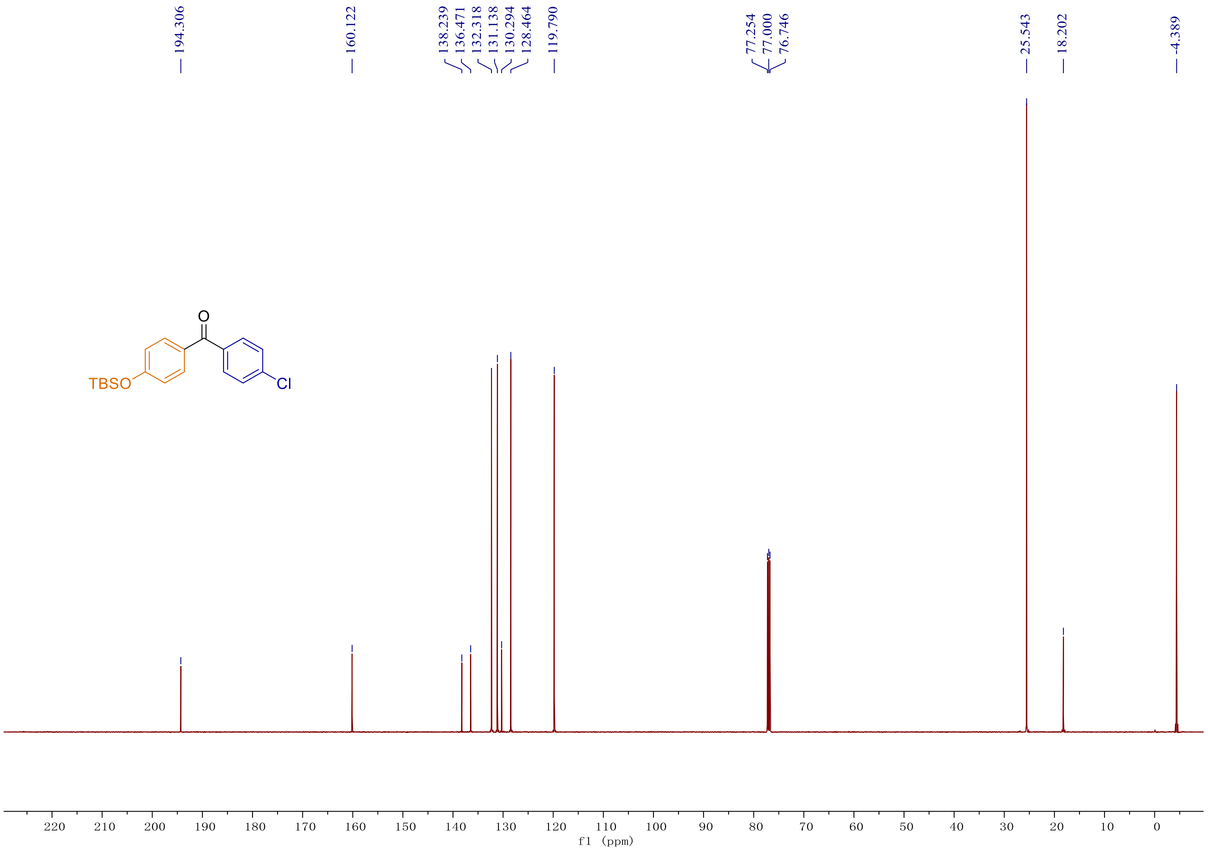

Supplementary Fig. 141 ^{13}C NMR (126 MHz, Chloroform-*d*) of (4-((tert-butyldimethylsilyl)oxy)phenyl)(4-chlorophenyl)methanone (1q).

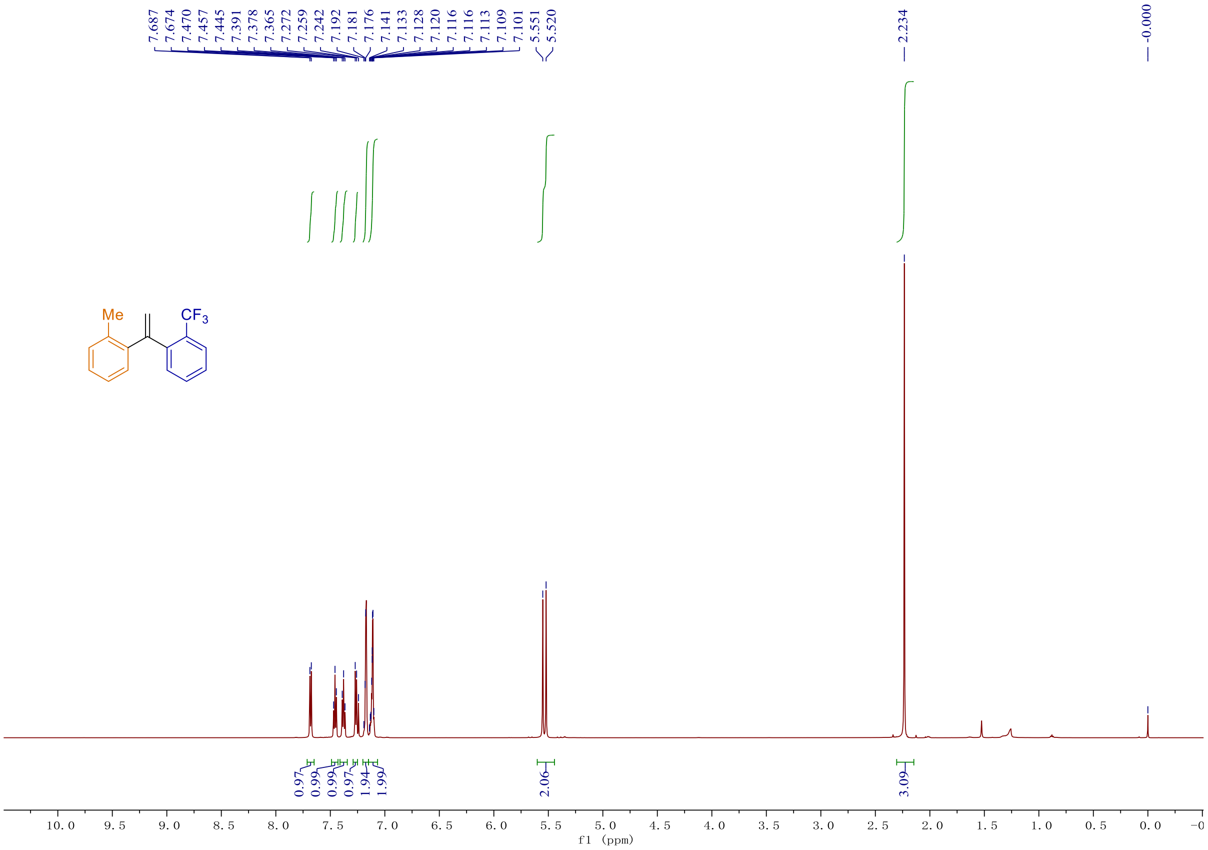

Supplementary Fig. 142 ^{1}H NMR (600 MHz, Chloroform-*d*) of 1-methyl-2-(1-(2-(trifluoromethyl)phenyl)vinyl)benzene (3d).

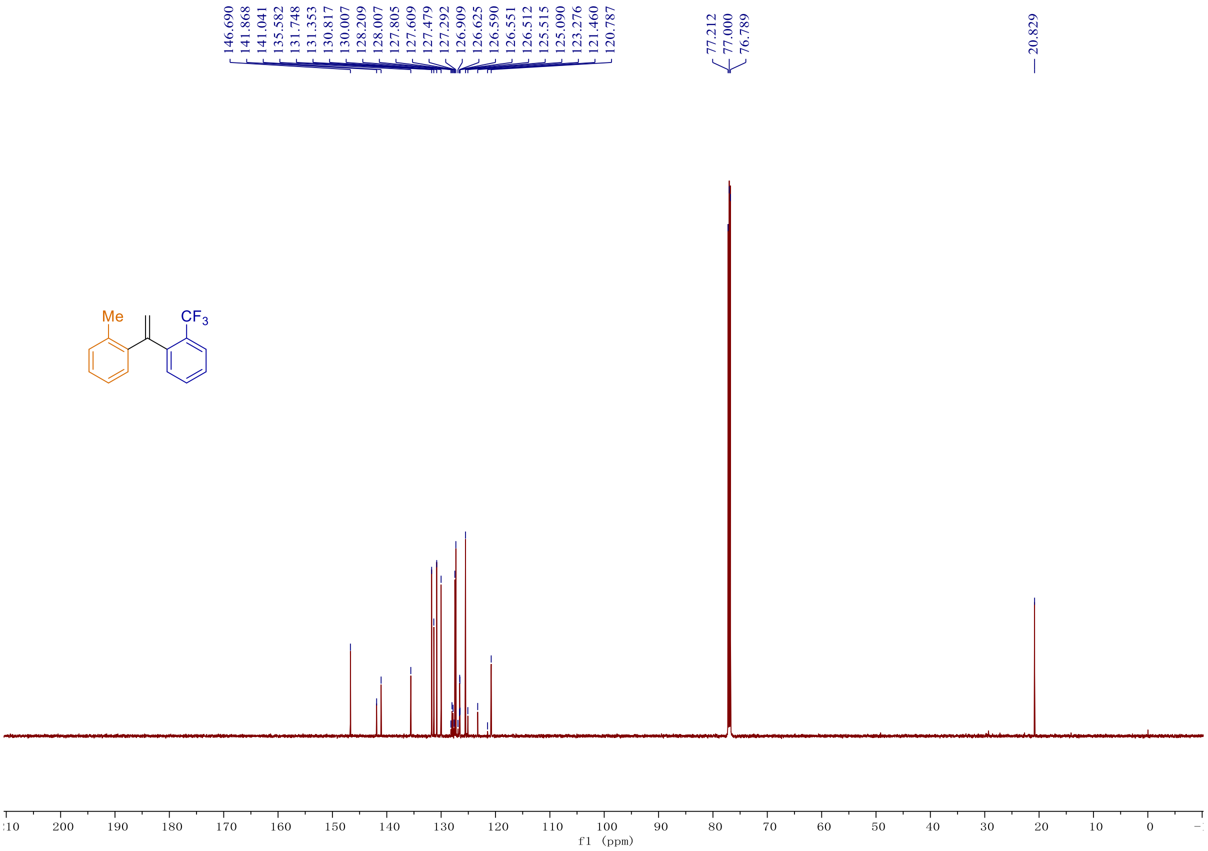

Supplementary Fig. 143 ^{13}C NMR (151 MHz, Chloroform-*d*) of 1-methyl-2-(1-(2-(trifluoromethyl)phenyl)vinyl)benzene (3d).

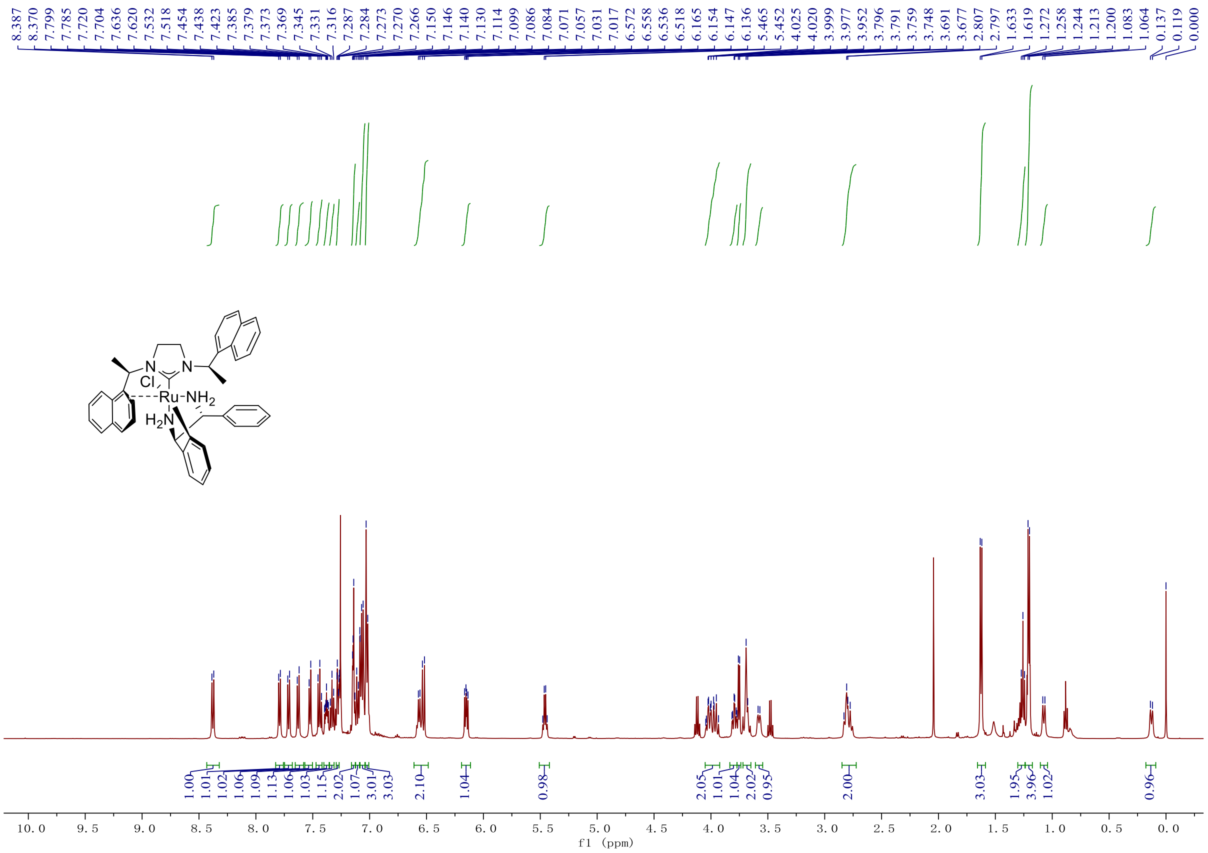

Supplementary Fig. 144 ^{1}H NMR (500 MHz, Chloroform-*d*) of Ru-1.

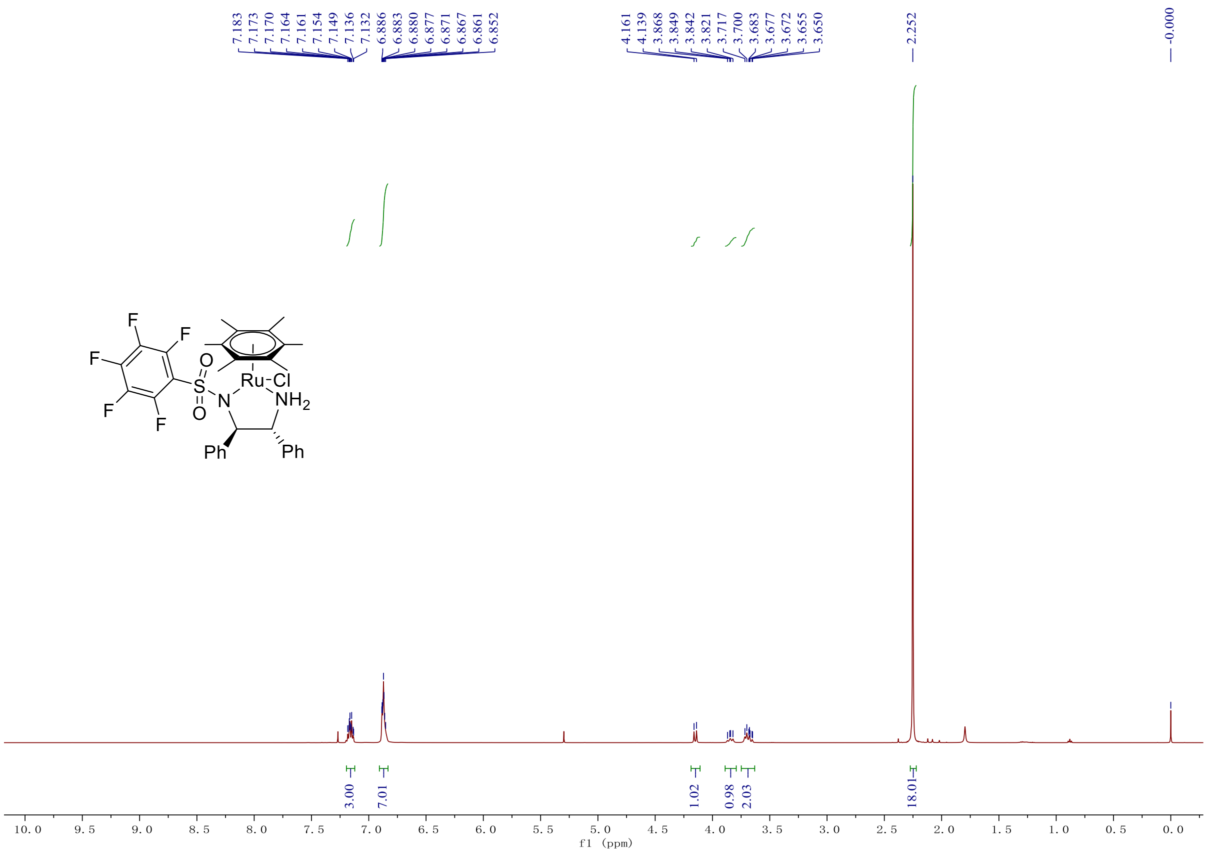

Supplementary Fig. 145 ^{1}H NMR (500 MHz, Chloroform-*d*) of Ru-2.

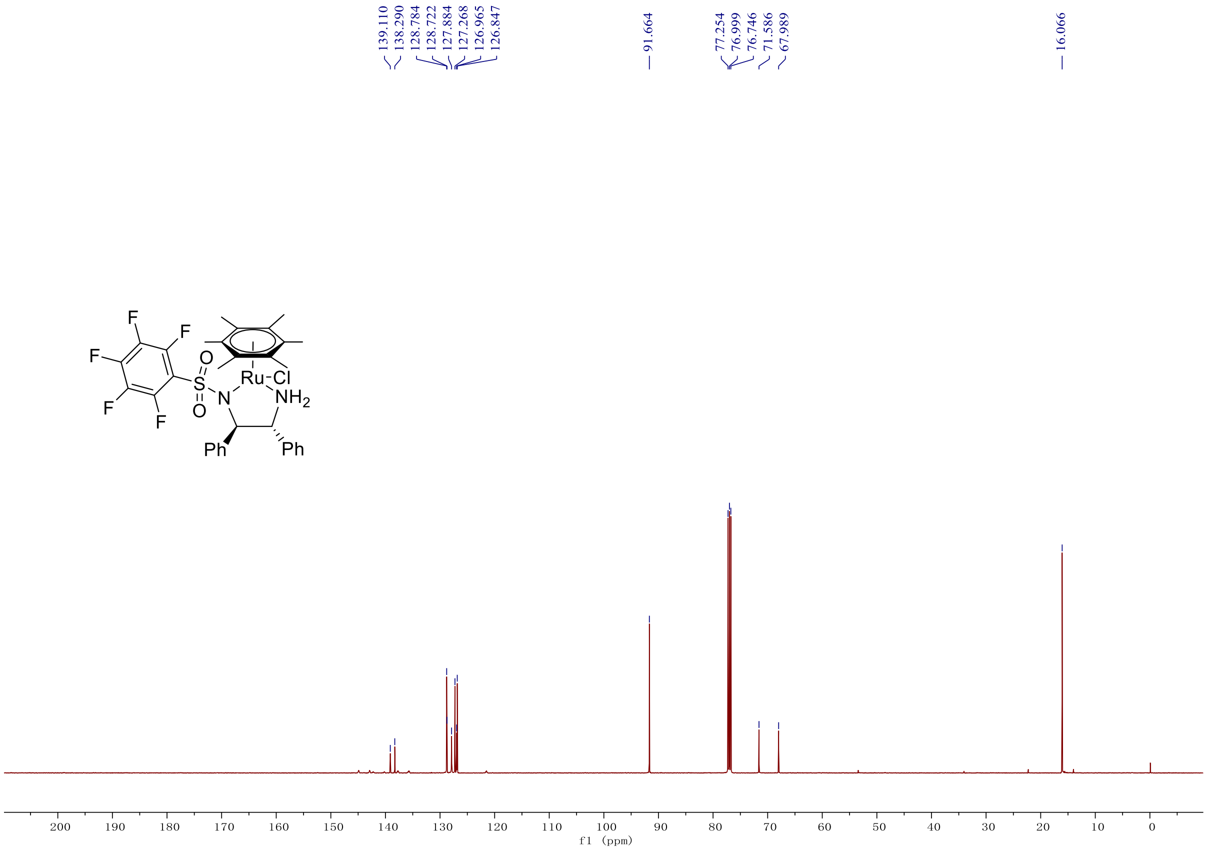

Supplementary Fig. 146 ^{13}C NMR (126 MHz, Chloroform-*d*) of Ru-2.

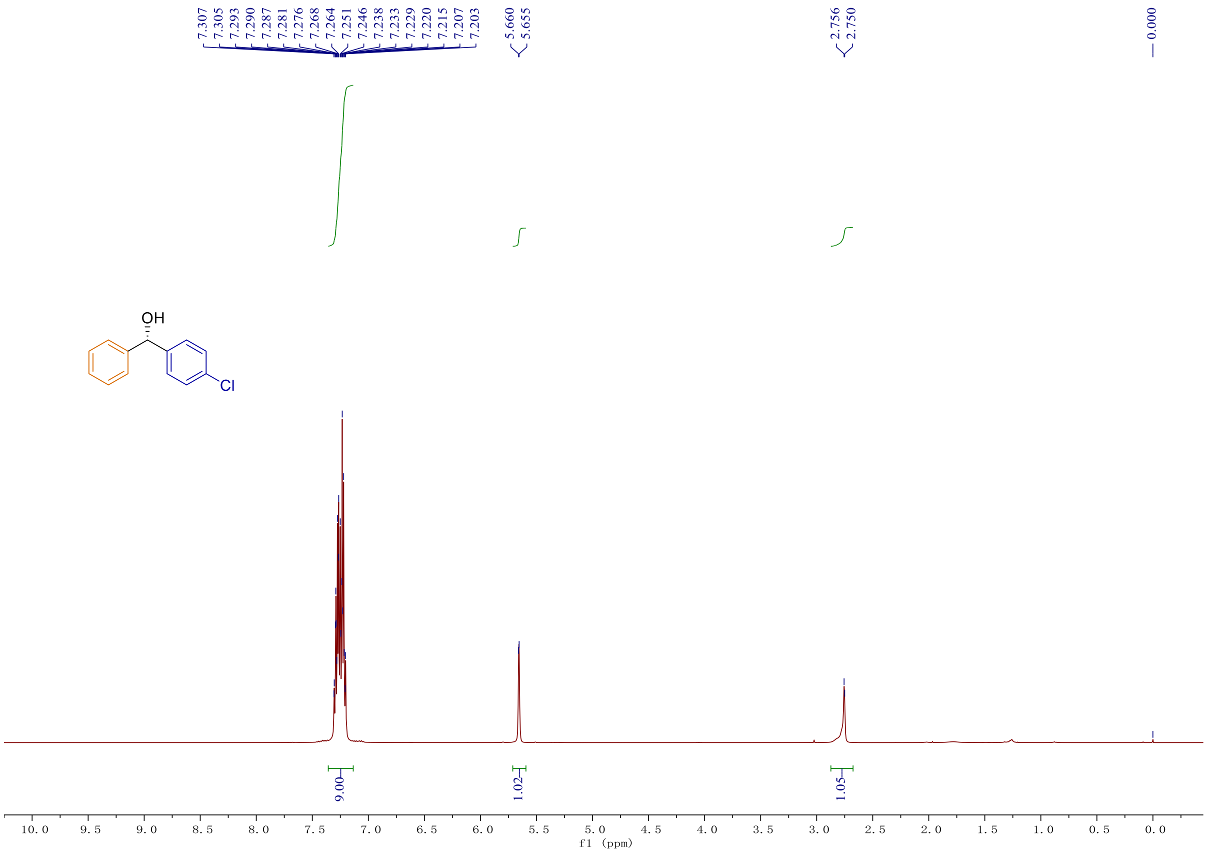

Supplementary Fig. 147 ^{1}H NMR (500 MHz, Chloroform-*d*) of (*S*)-(4-chlorophenyl)(phenyl)methanol (4a).

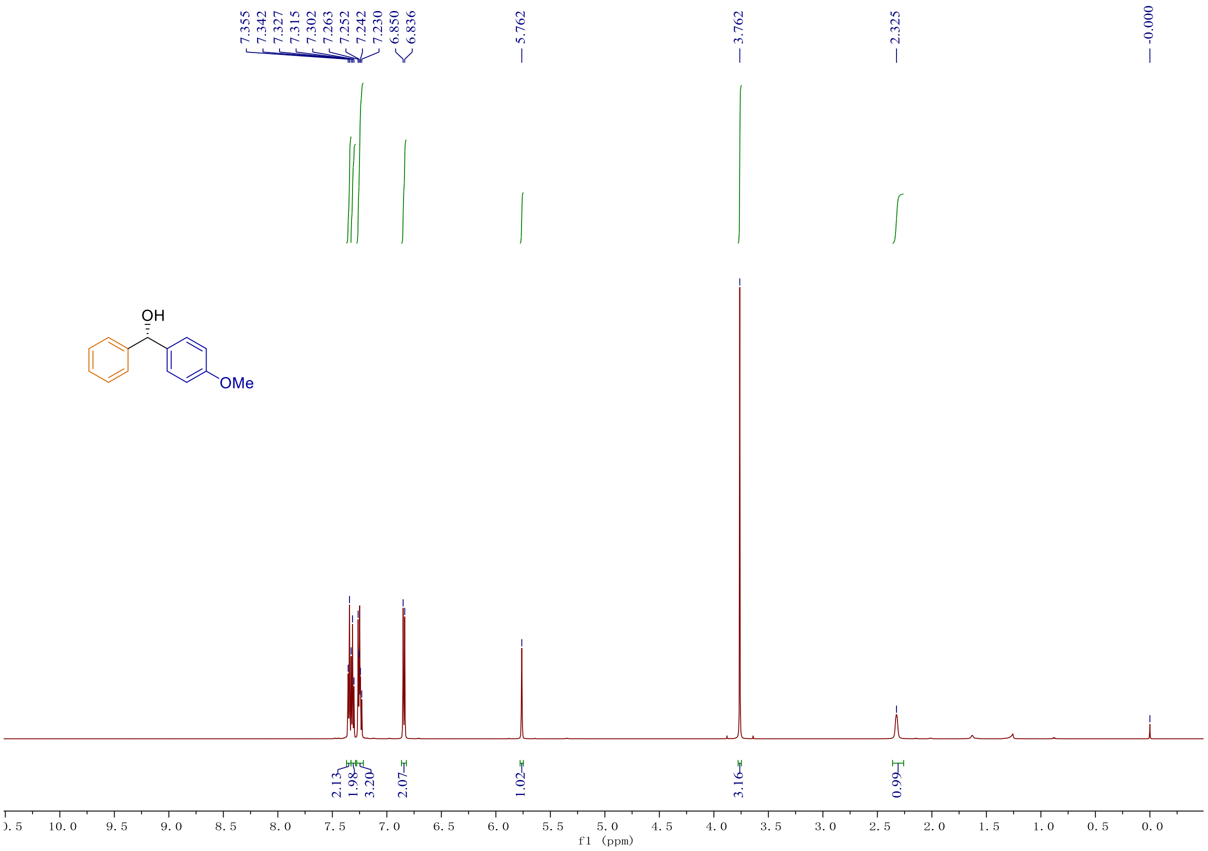

Supplementary Fig. 148 ^{1}H NMR (600 MHz, Chloroform-*d*) of (*R*)-(4-methoxyphenyl)(phenyl)methanol (4b).

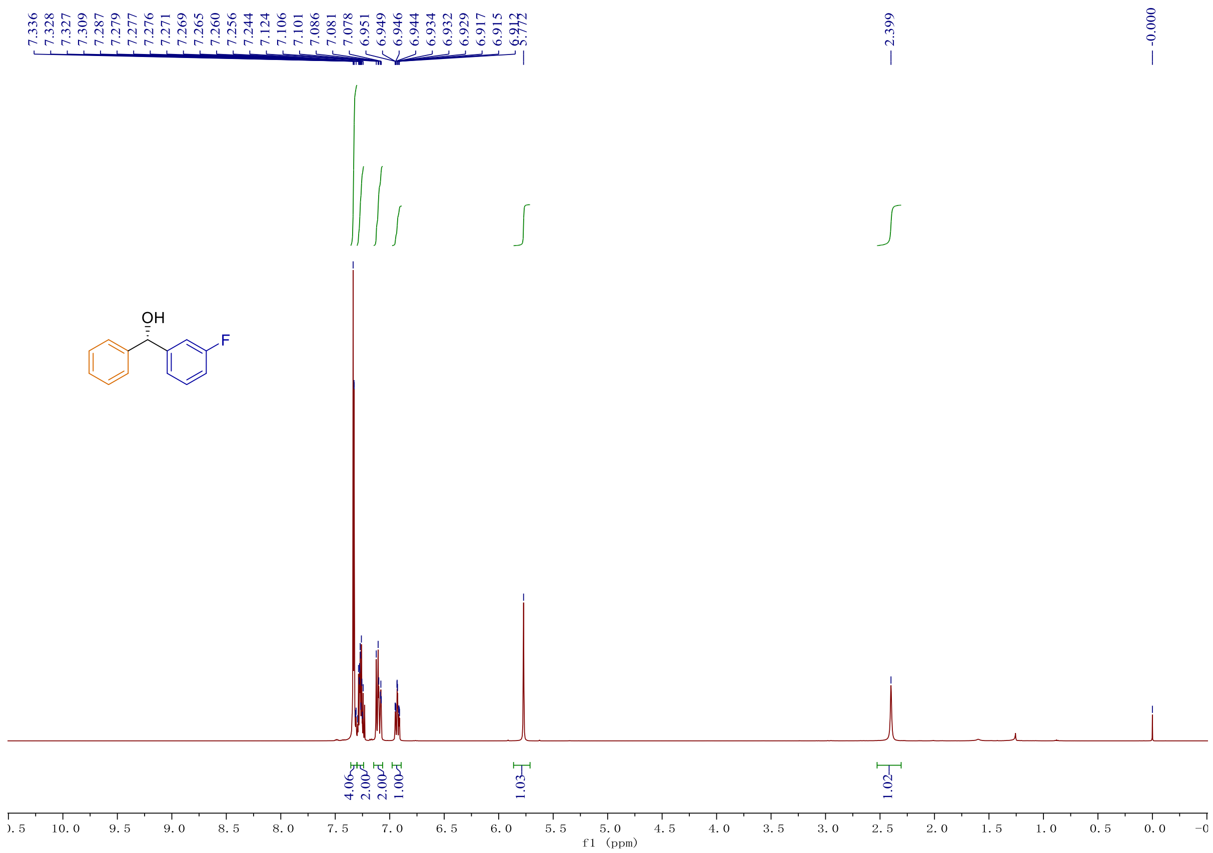

Supplementary Fig. 149 ^{1}H NMR (500 MHz, Chloroform-*d*) of (*S*)-(3-fluorophenyl)(phenyl)methanol (4c).

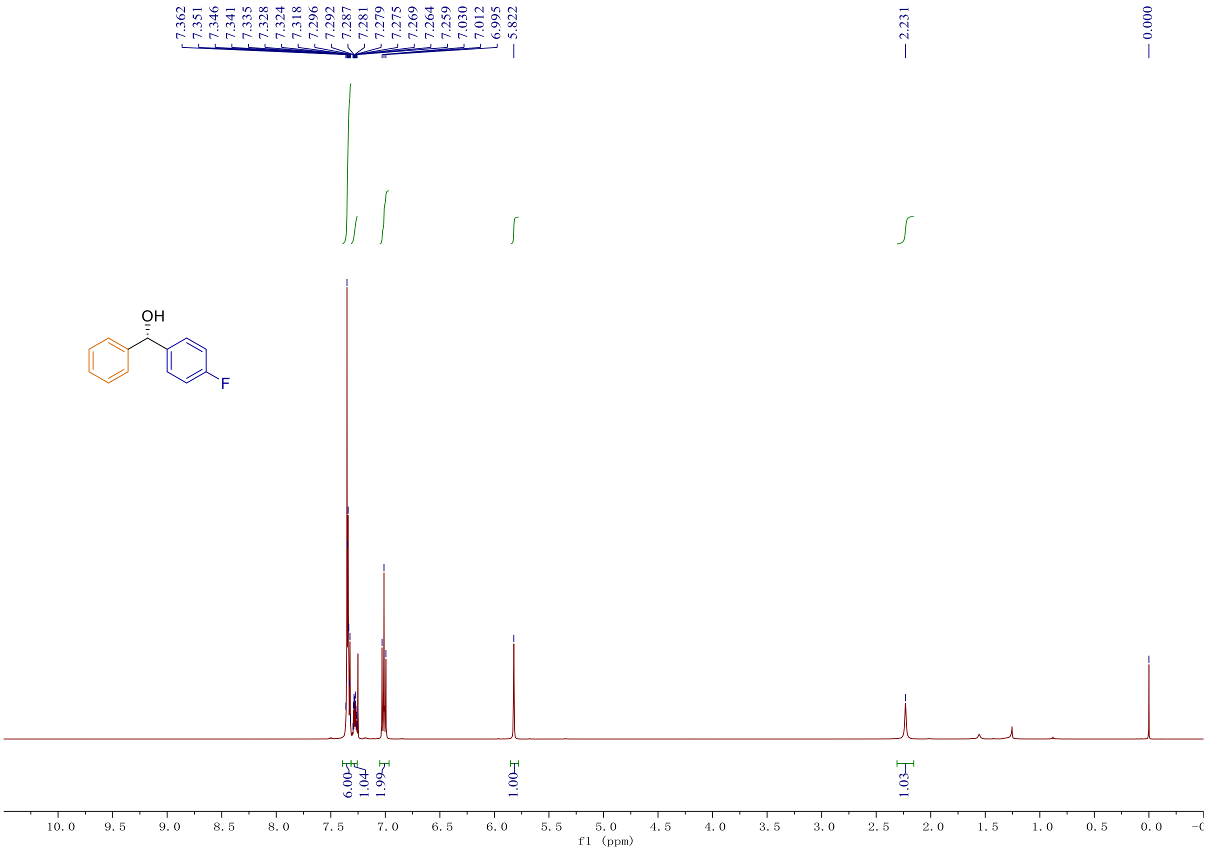

Supplementary Fig. 150 ^{1}H NMR (500 MHz, Chloroform-*d*) of (*S*)-(4-fluorophenyl)(phenyl)methanol (4d).

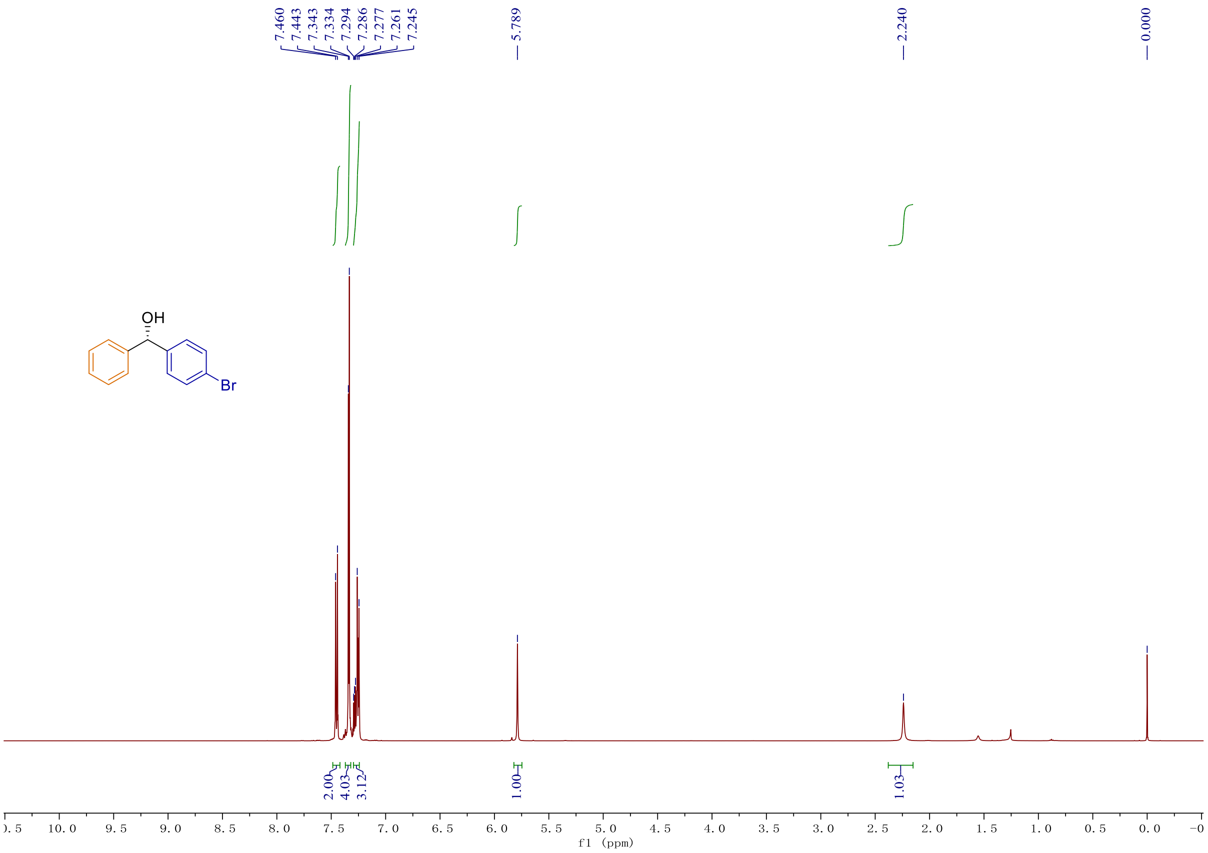

Supplementary Fig. 151 ^{1}H NMR (500 MHz, Chloroform-*d*) of (*S*)-(4-bromophenyl)(phenyl)methanol (4e).

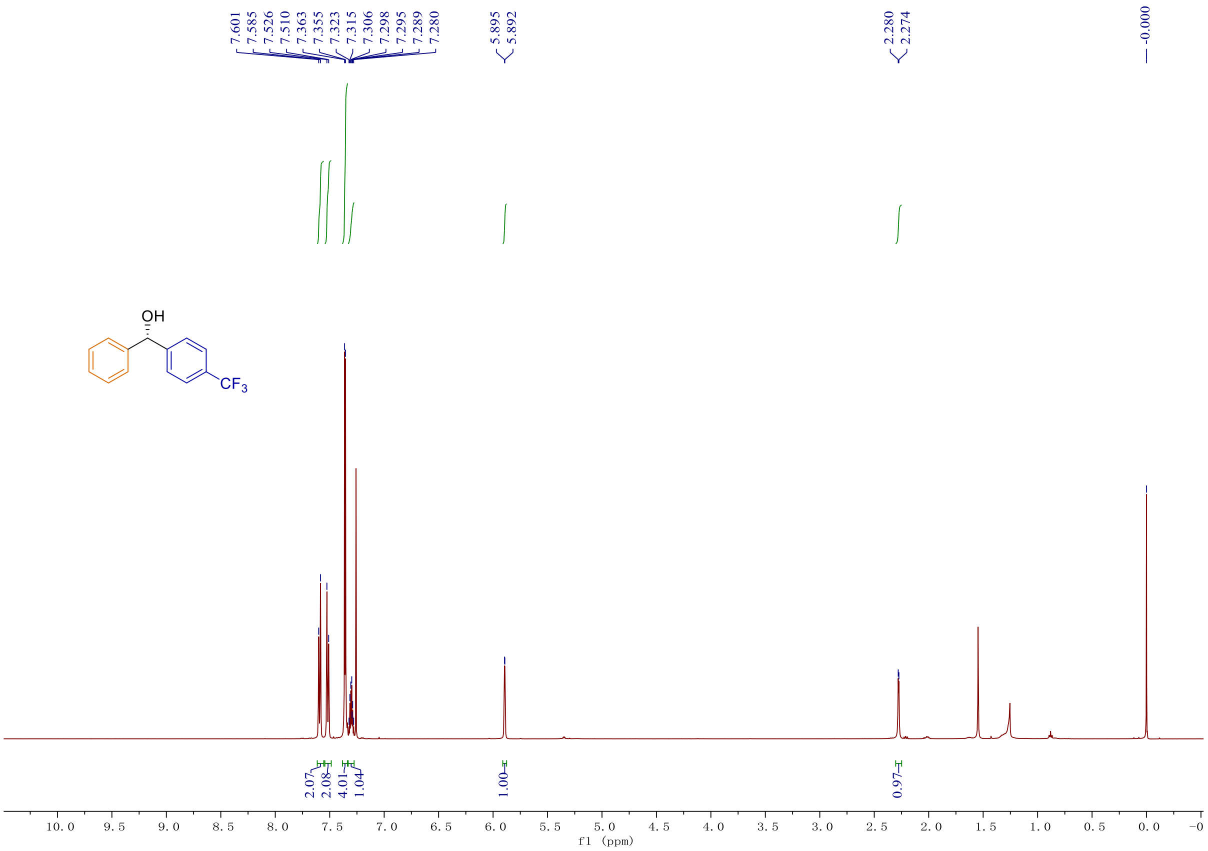

Supplementary Fig. 152 ^{1}H NMR (500 MHz, Chloroform-*d*) of (*S*)-phenyl(4-(trifluoromethyl)phenyl)methanol (4f).

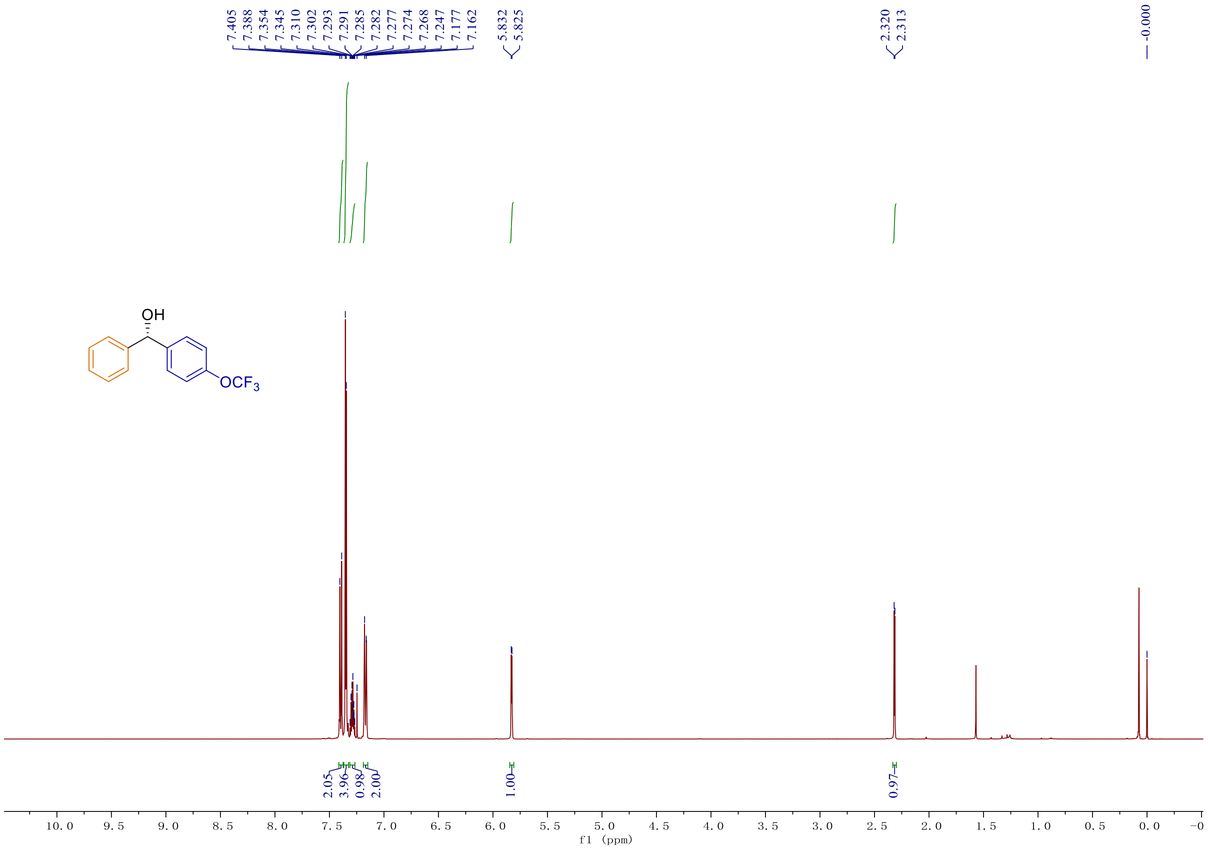

Supplementary Fig. 153 H NMR (500 MHz, Chloroform-*d*) of (*S*)-phenyl(4-(trifluoromethoxy)phenyl)methanol (4g).

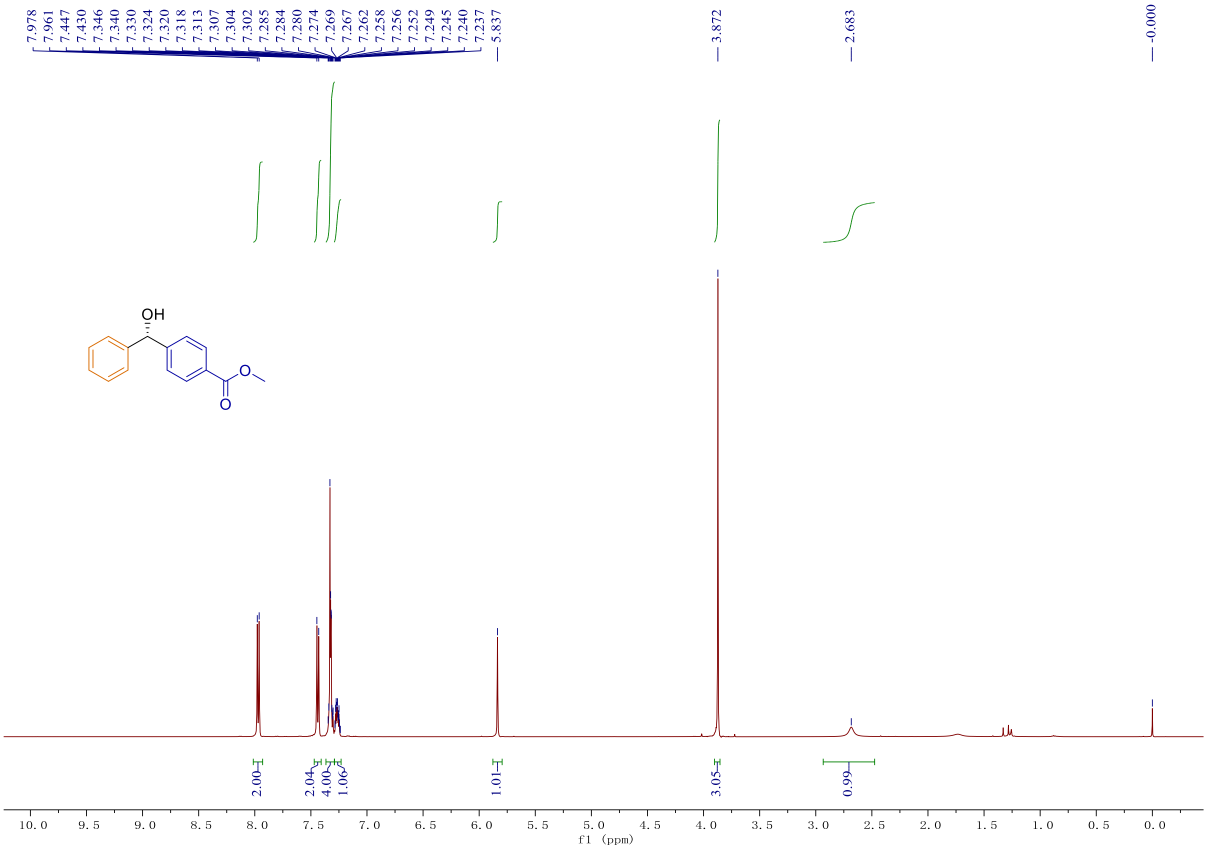

Supplementary Fig. 154 ^{1}H NMR (500 MHz, Chloroform-*d*) of (*S*)-4-(hydroxy(phenyl)methyl)benzoate (4h).

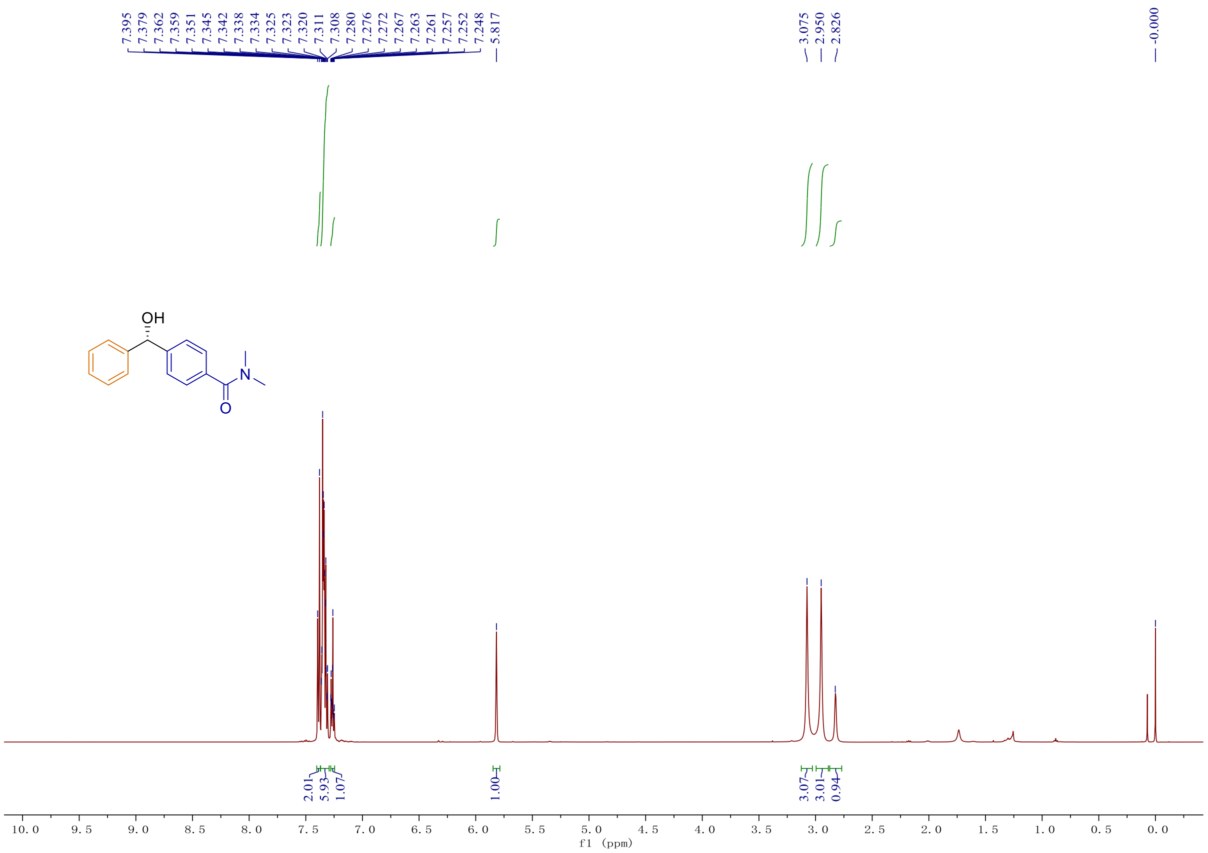

Supplementary Fig. 155 ^{1}H NMR (500 MHz, Chloroform-*d*) of (*S*)-4-(hydroxy(phenyl)methyl)-*N*,*N*-dimethylbenzamide (4i).

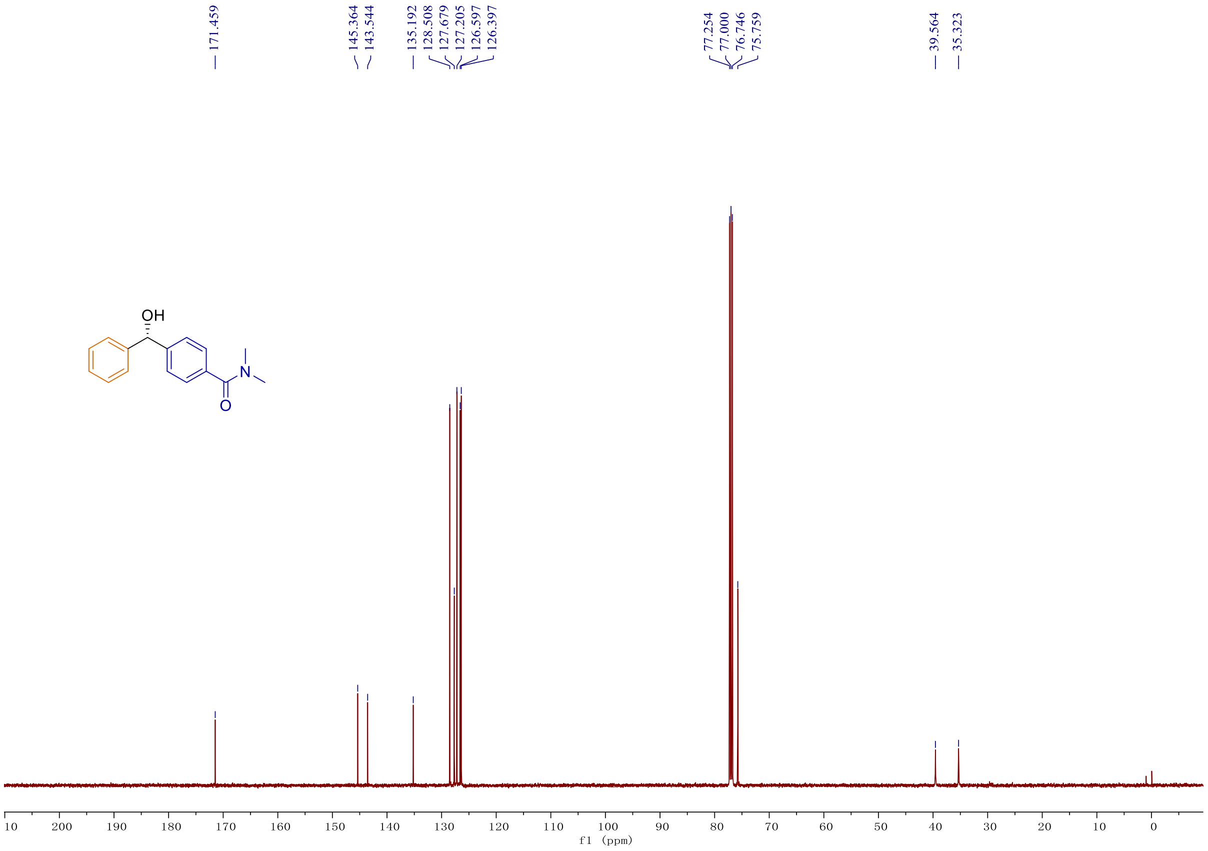

Supplementary Fig. 156 ^{13}C NMR (126 MHz, Chloroform-*d*) of (*S*)-4-(hydroxy(phenyl)methyl)-*N*,*N*-dimethylbenzamide (4i).

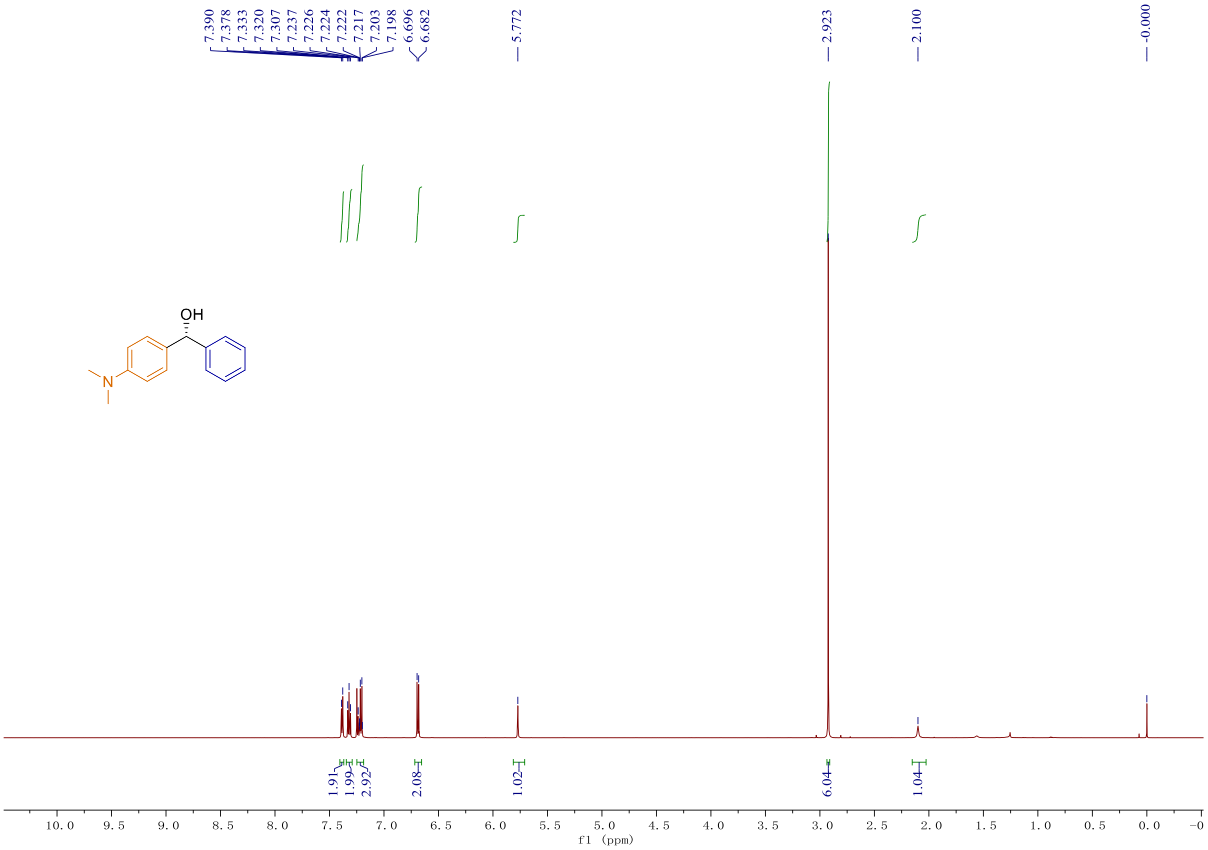

Supplementary Fig. 157 ¹H NMR (500 MHz, Chloroform-*d*) of (*R*)-(4-(dimethylamino)phenyl)(phenyl)methanol (4j).

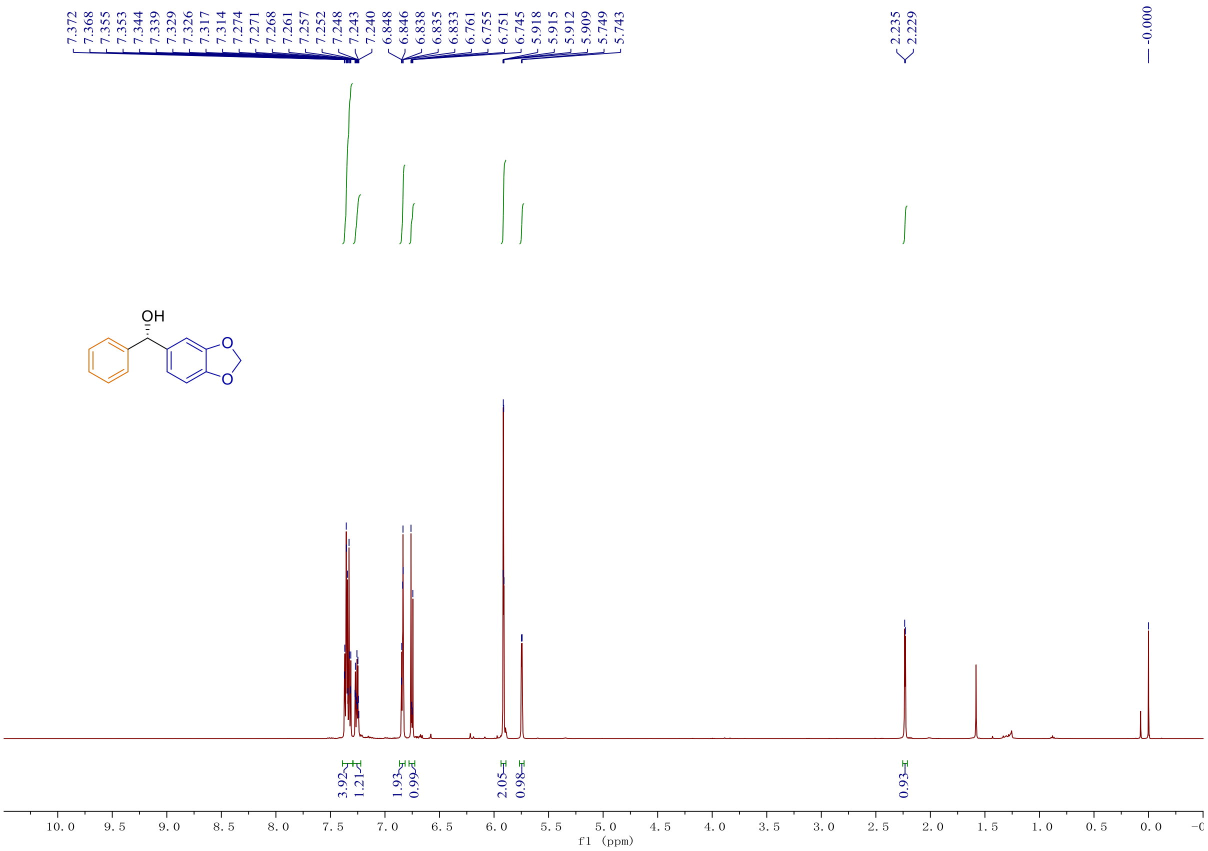

Supplementary Fig. 158 ¹H NMR (500 MHz, Chloroform-*d*) of (*S*)-benzo[d][1,3]dioxol-5-yl(phenyl)methanol (4k).

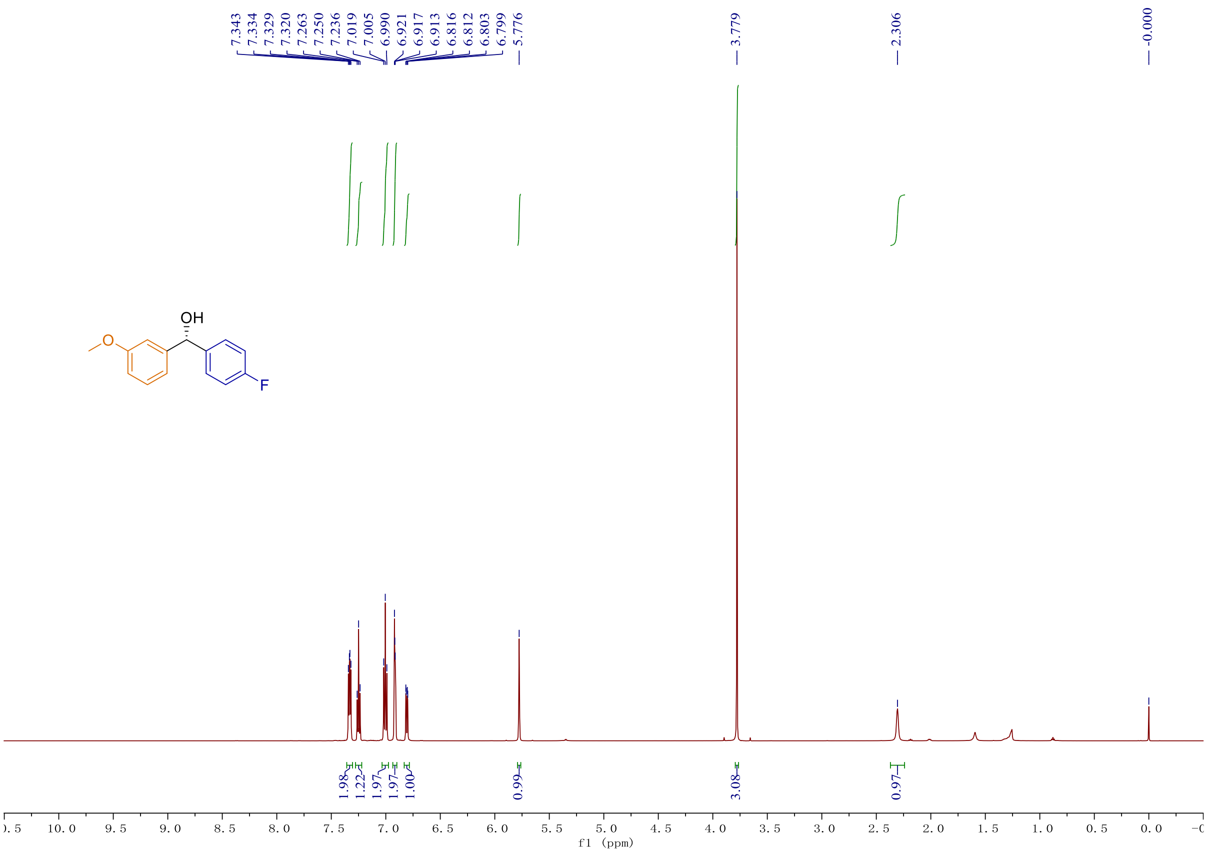

Supplementary Fig. 159 ^{1}H NMR (600 MHz, Chloroform-*d*) (*R*)-(4-fluorophenyl)(3-methoxyphenyl)methanol (4l).

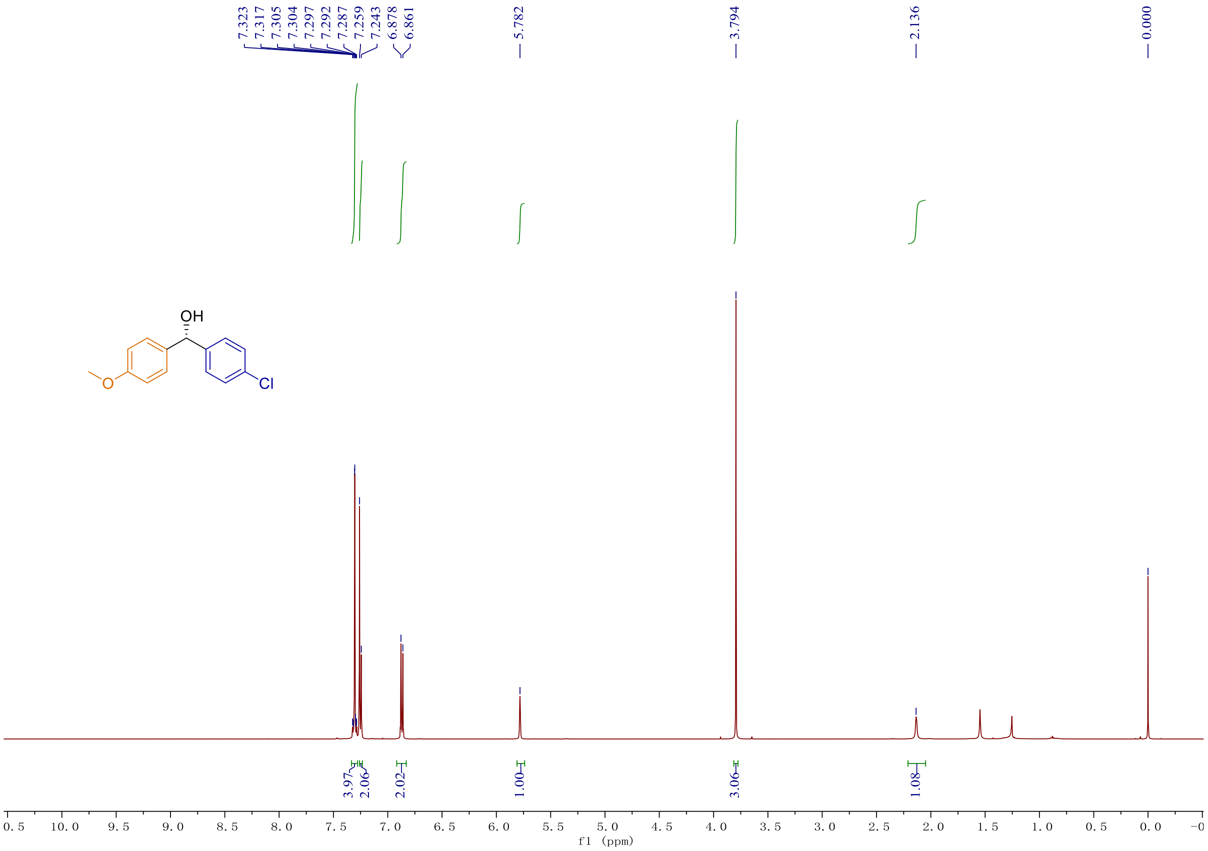

Supplementary Fig. 160 ^{1}H NMR (500 MHz, Chloroform-*d*) of (*S*)-(4-chlorophenyl)(4-methoxyphenyl)methanol (4m).

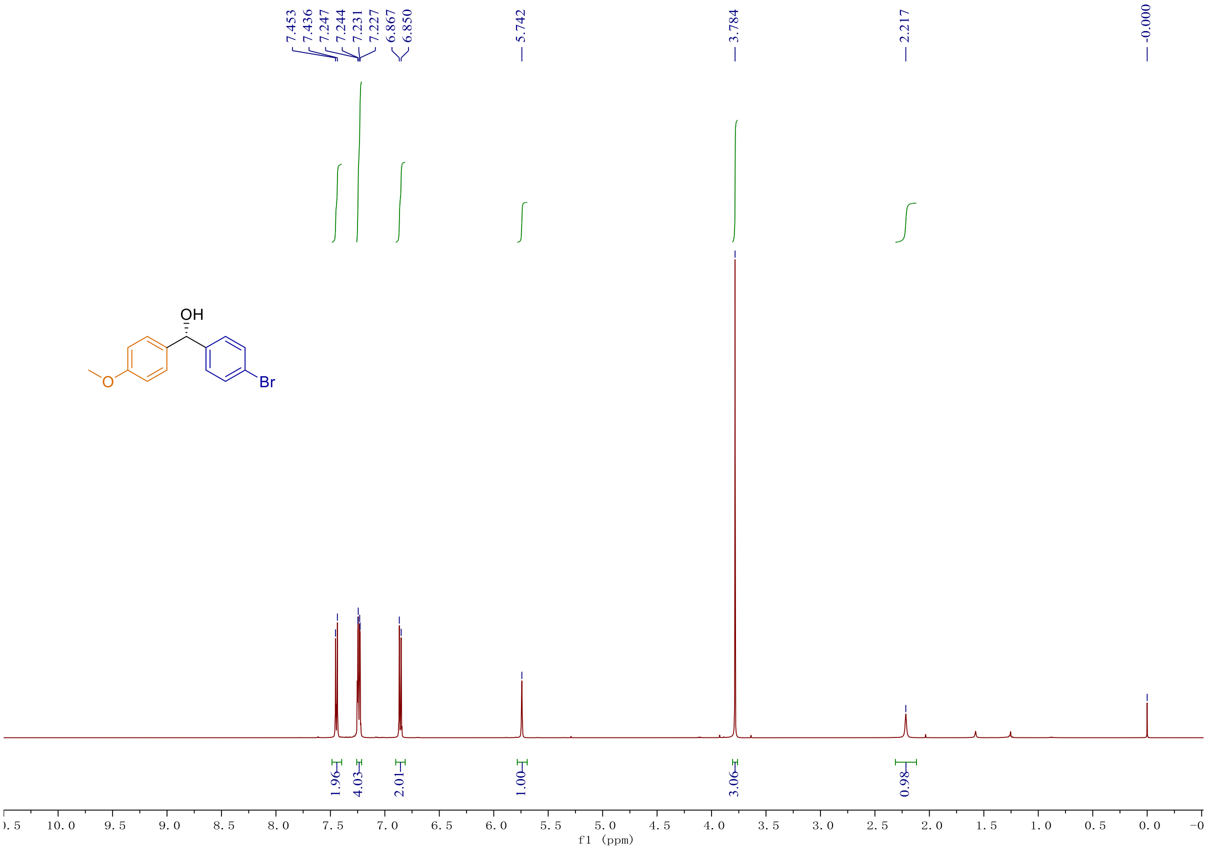

Supplementary Fig. 161 ^{1}H NMR (500 MHz, Chloroform-*d*) of (*S*)-(4-bromophenyl)(4-methoxyphenyl)methanol (4n).

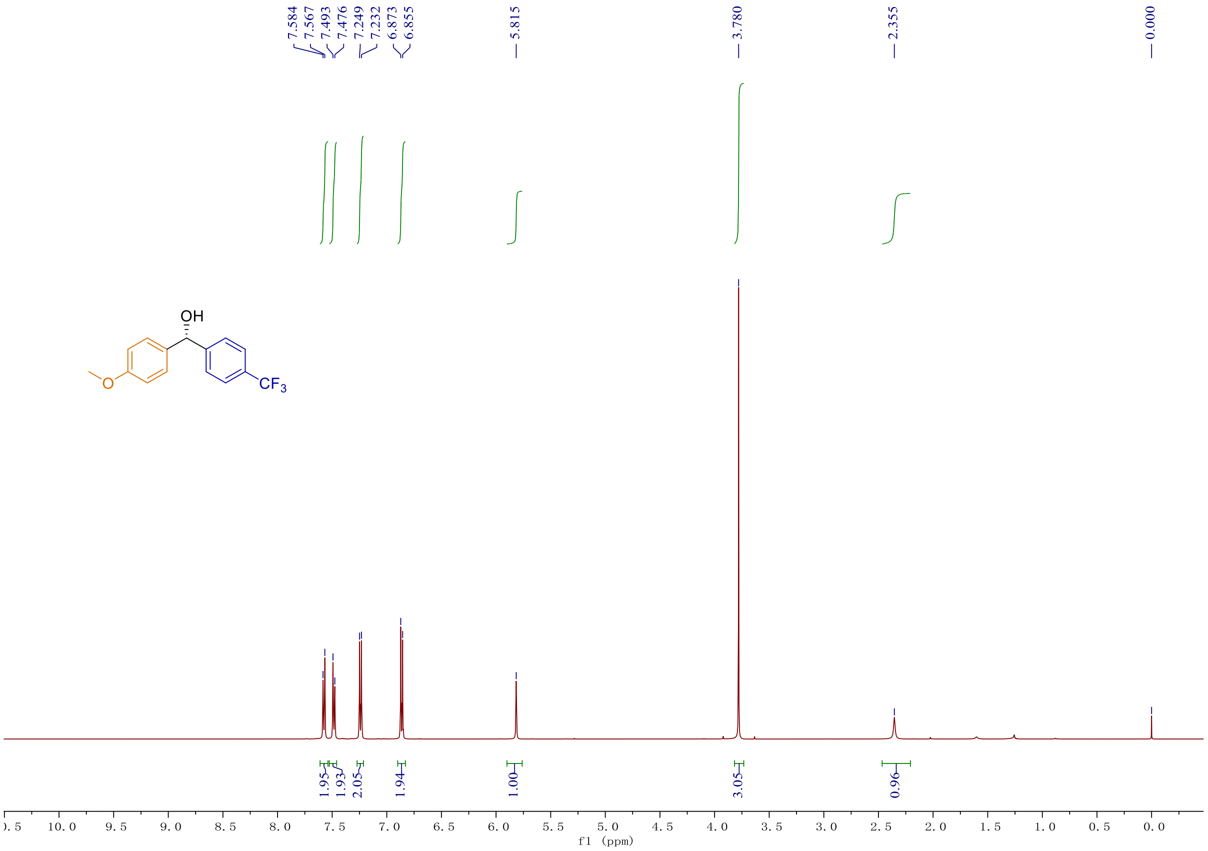

Supplementary Fig. 162 ^{1}H NMR (500 MHz, Chloroform-*d*) of (*R*)-(4-methoxyphenyl)(4-(trifluoromethyl)phenyl)methanol (4o).

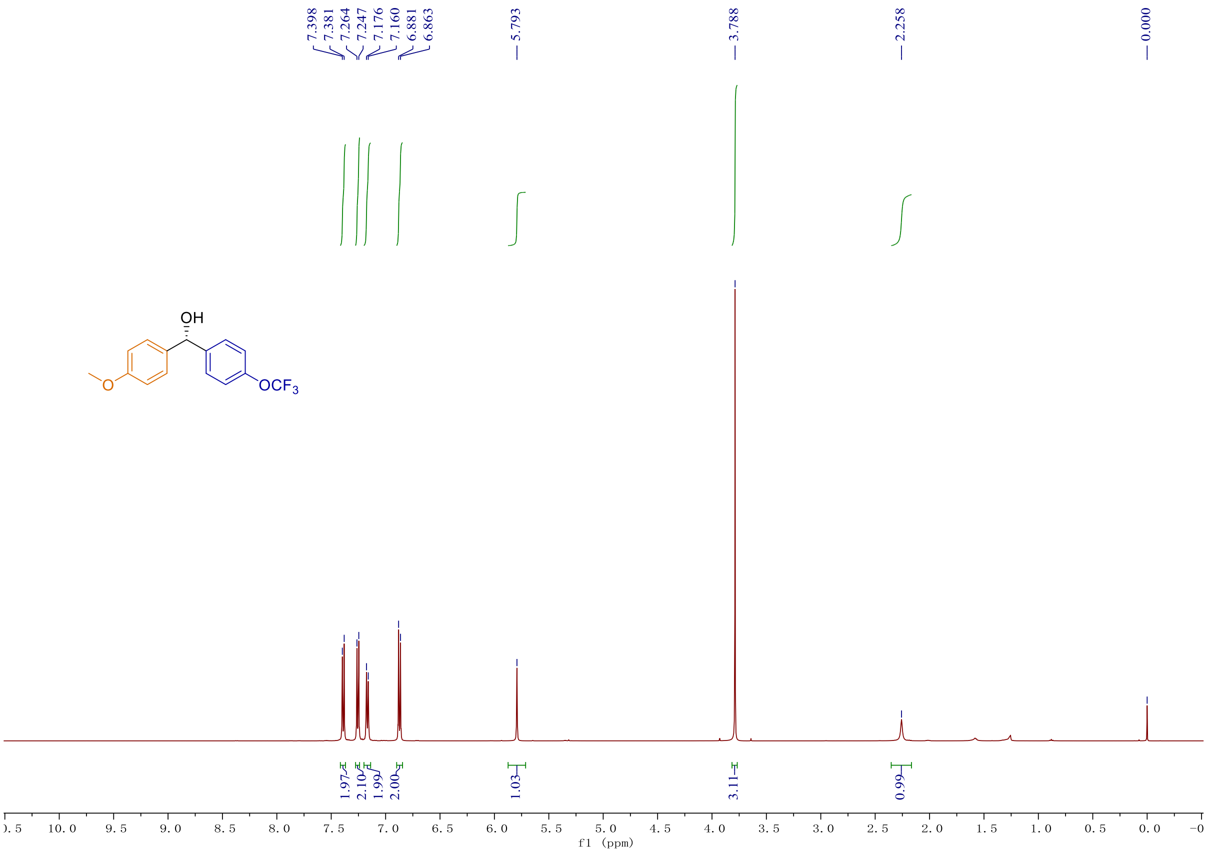

Supplementary Fig. 163 ¹H NMR (500 MHz, Chloroform-*d*) of (*S*)-(4-methoxyphenyl)(4-(trifluoromethoxy)phenyl)methanol (4p).

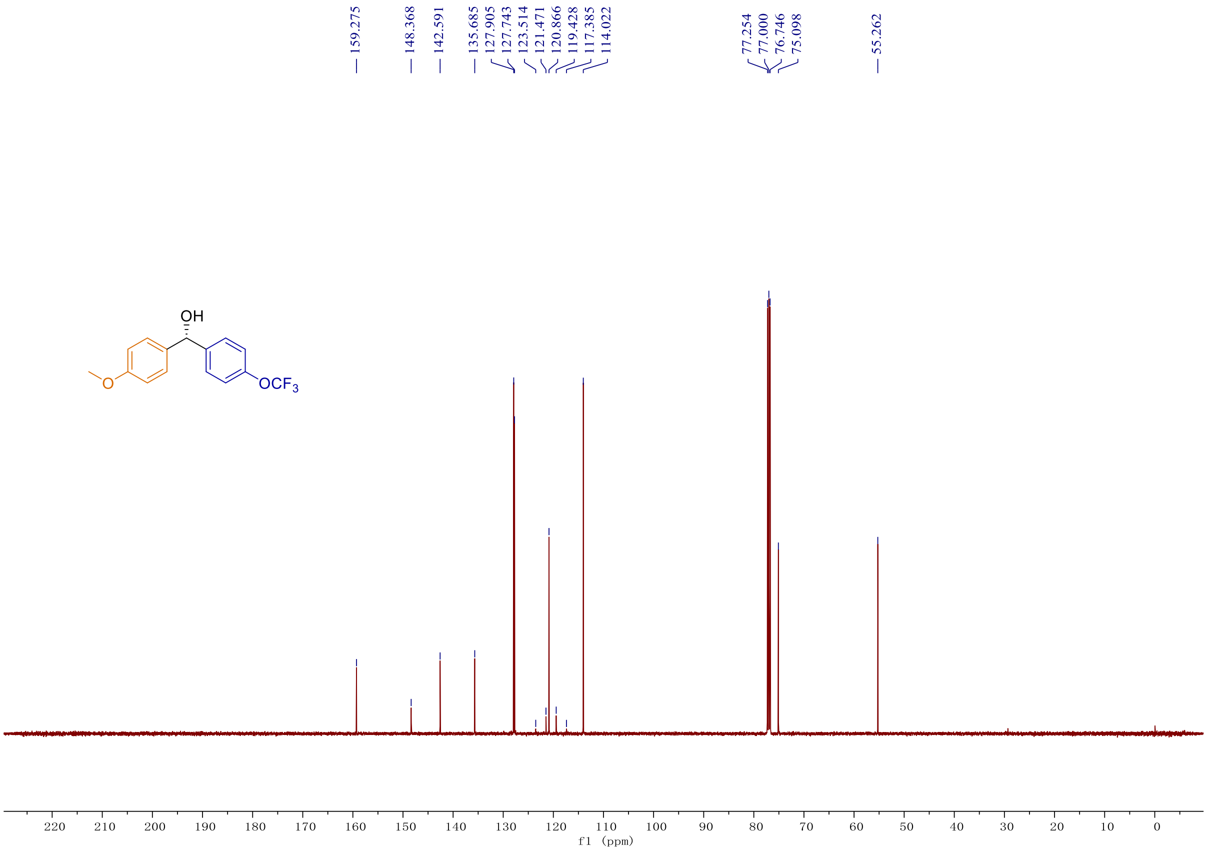

Supplementary Fig. 164 ¹³C NMR (126 MHz, Chloroform-*d*) of (*S*)-(4-methoxyphenyl)(4-(trifluoromethoxy)phenyl)methanol (4p).

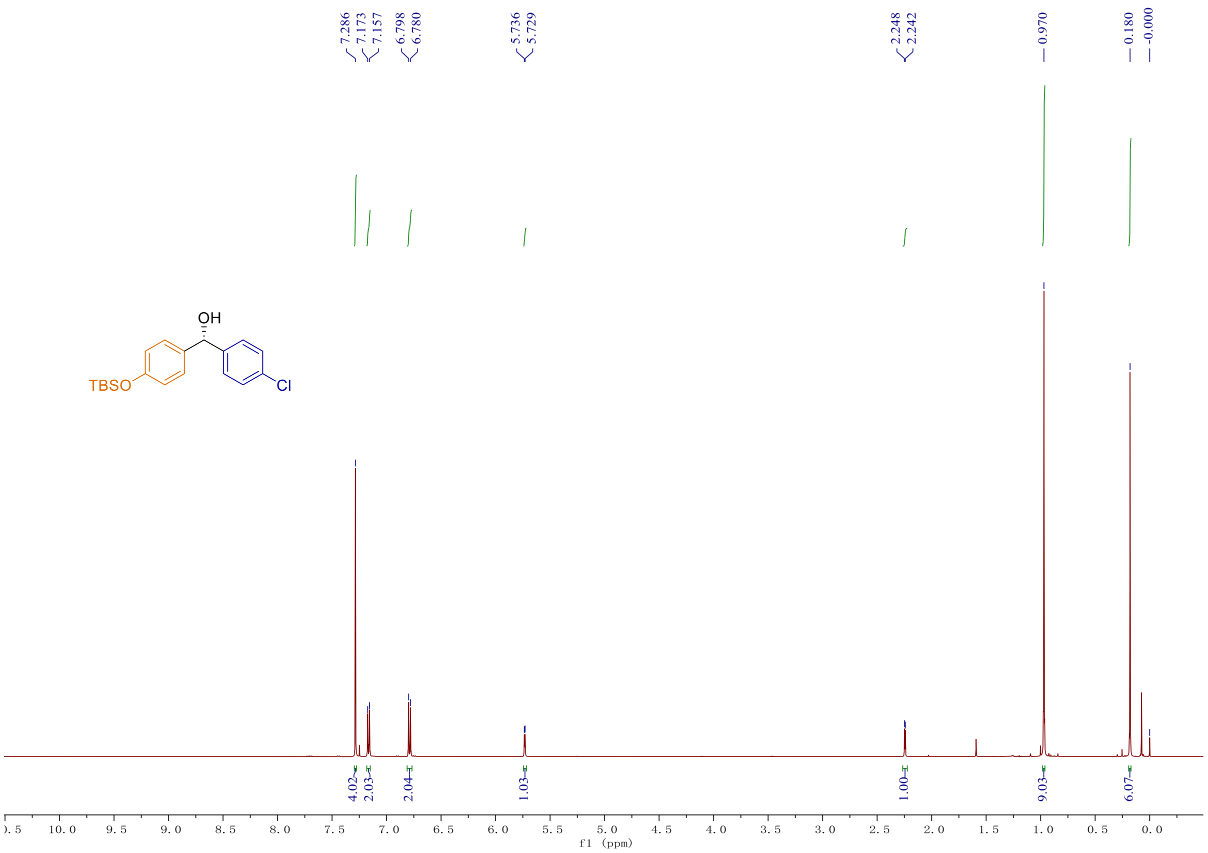

Supplementary Fig. 165 ^{1}H NMR (500 MHz, Chloroform-*d*) of (*S*)-(4-((tert-butyldimethylsilyl)oxy)phenyl)(4-chlorophenyl)methanol (4q).

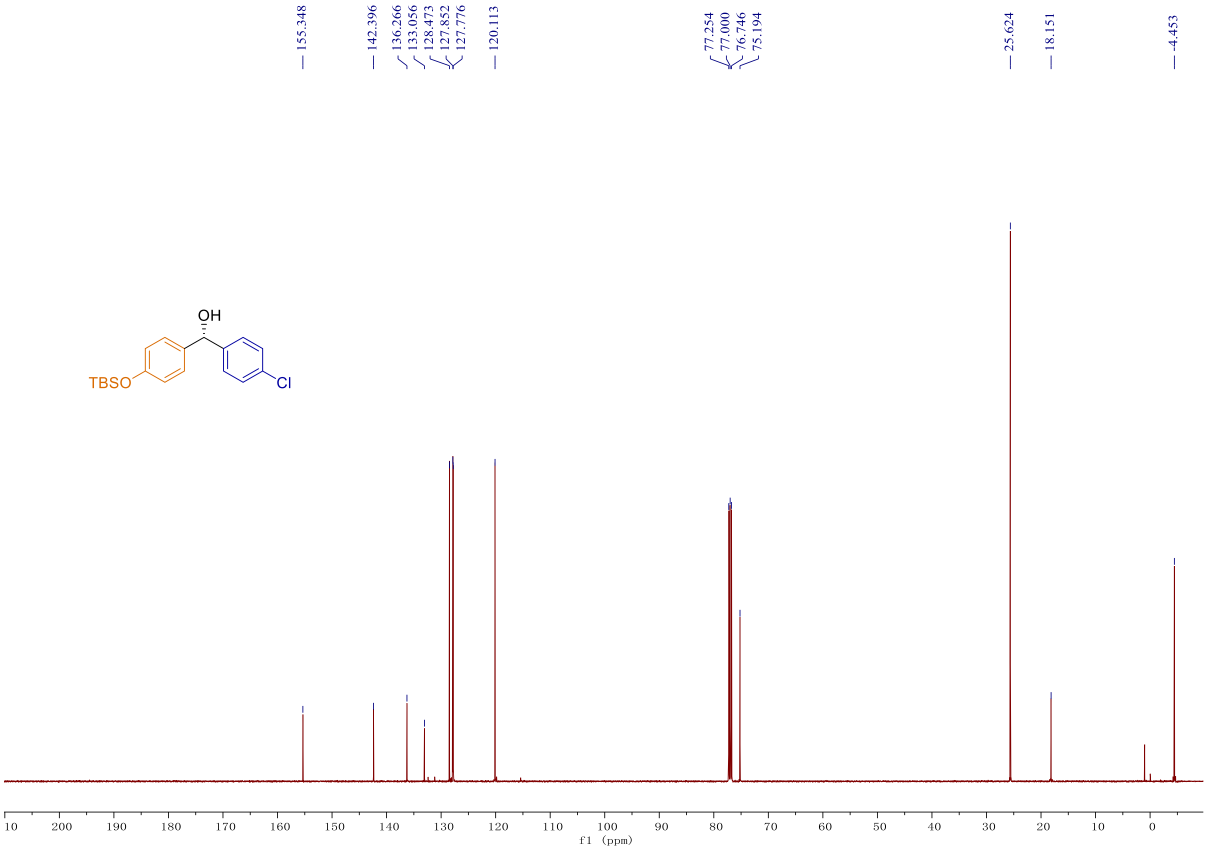

Supplementary Fig. 166 ^{13}C NMR (126 MHz, Chloroform-*d*) of (*S*)-(4-((tert-butyldimethylsilyl)oxy)phenyl)(4-chlorophenyl)methanol (4q).

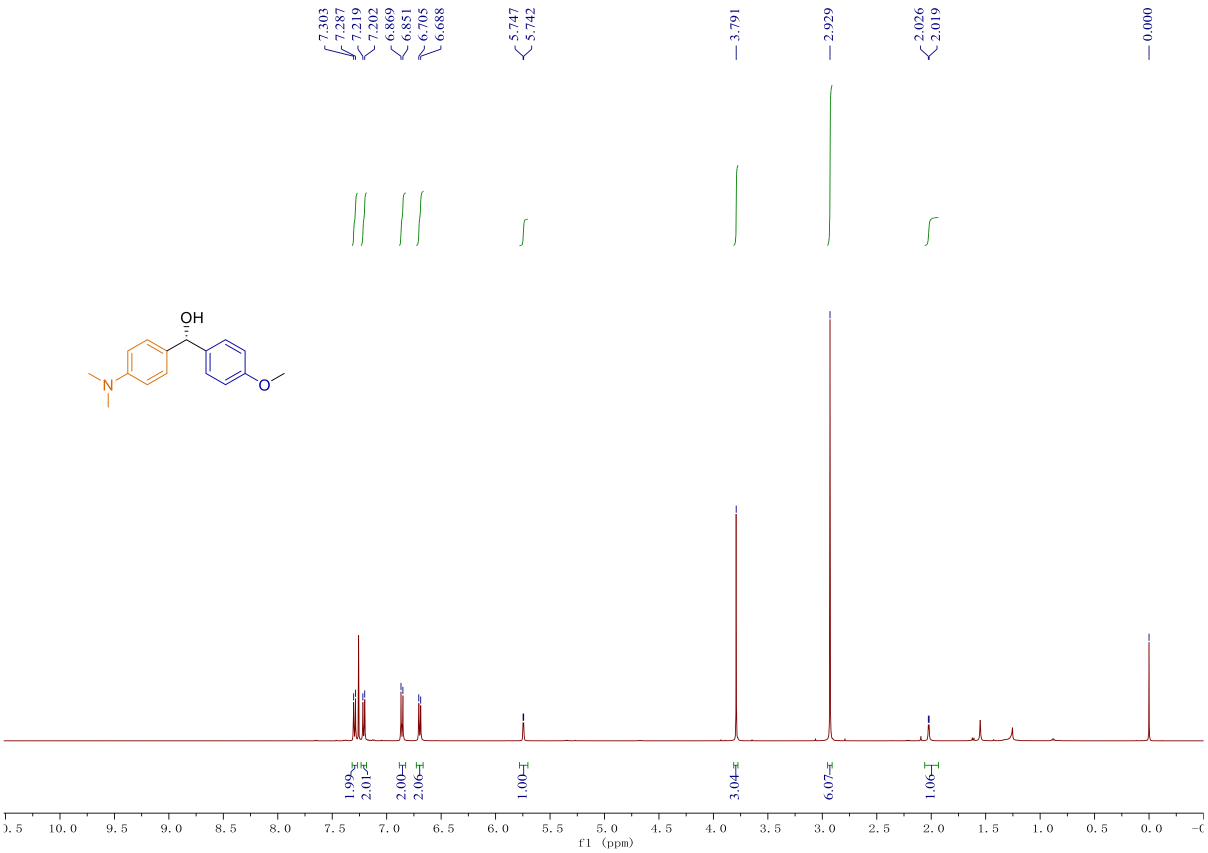

Supplementary Fig. 167 ^{1}H NMR (500 MHz, Chloroform-*d*) of (*S*)-(4-(dimethylamino)phenyl)(4-methoxyphenyl)methanol (4r).

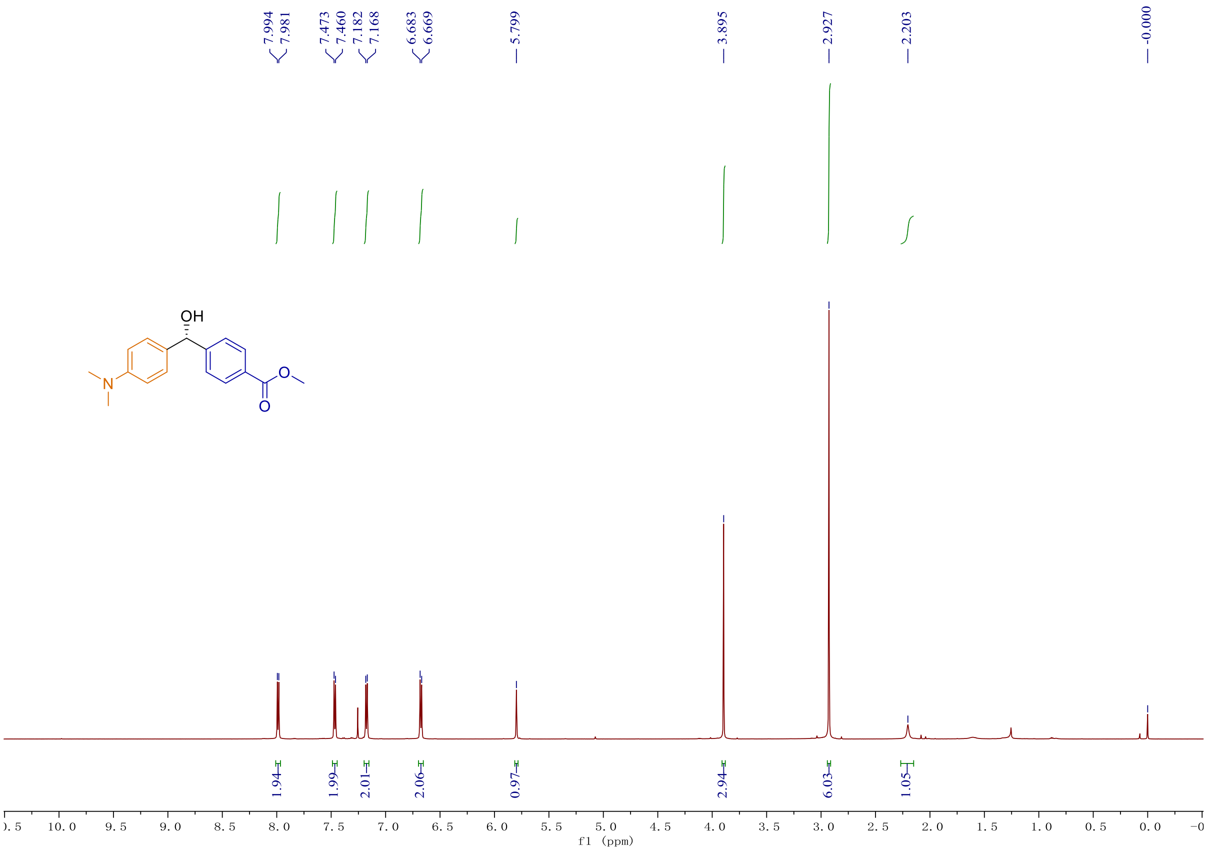

Supplementary Fig. 168 ^{1}H NMR (500 MHz, Chloroform-*d*) of methyl (*R*)-4-((4-(dimethylamino)phenyl)(hydroxy)methyl)benzoate (4s).

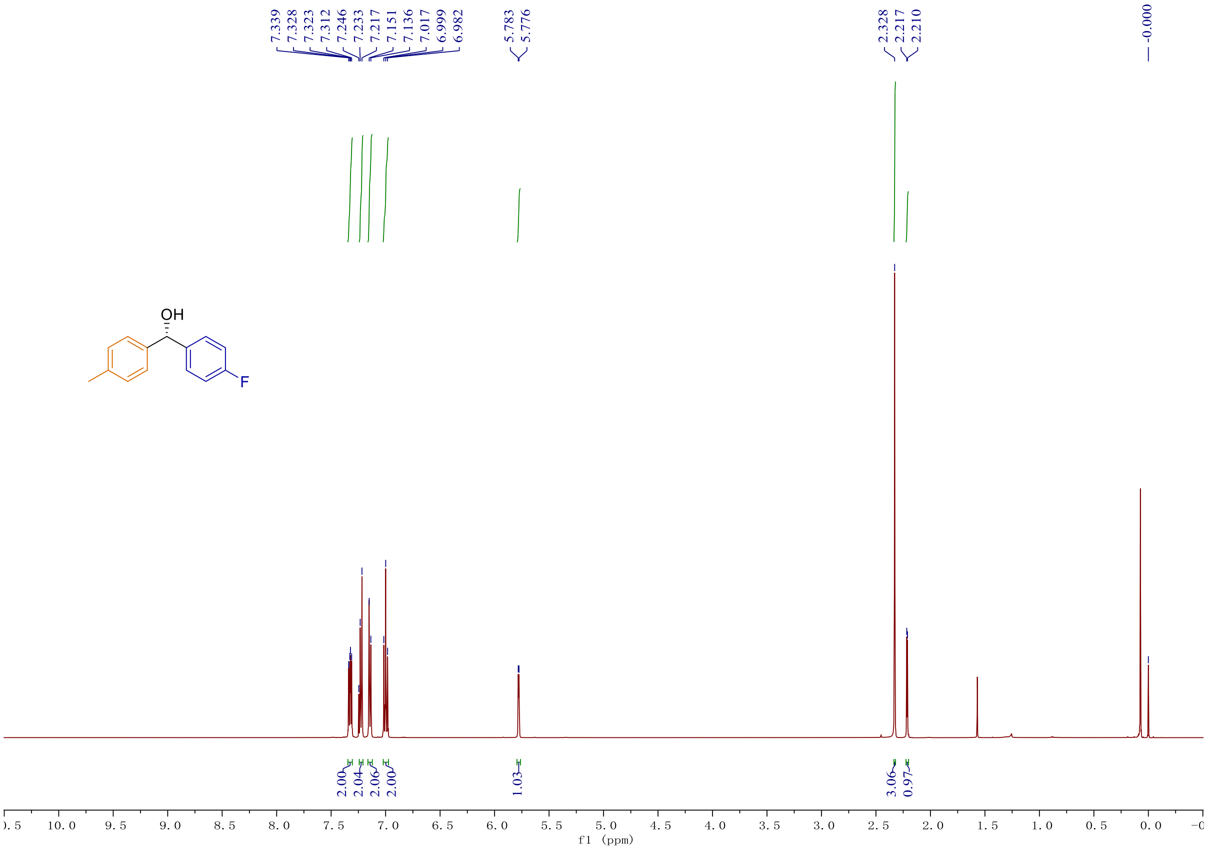

Supplementary Fig. 169 ^{1}H NMR (500 MHz, Chloroform-*d*) of (*S*)-(4-fluorophenyl)(p-tolyl)methanol (4t).

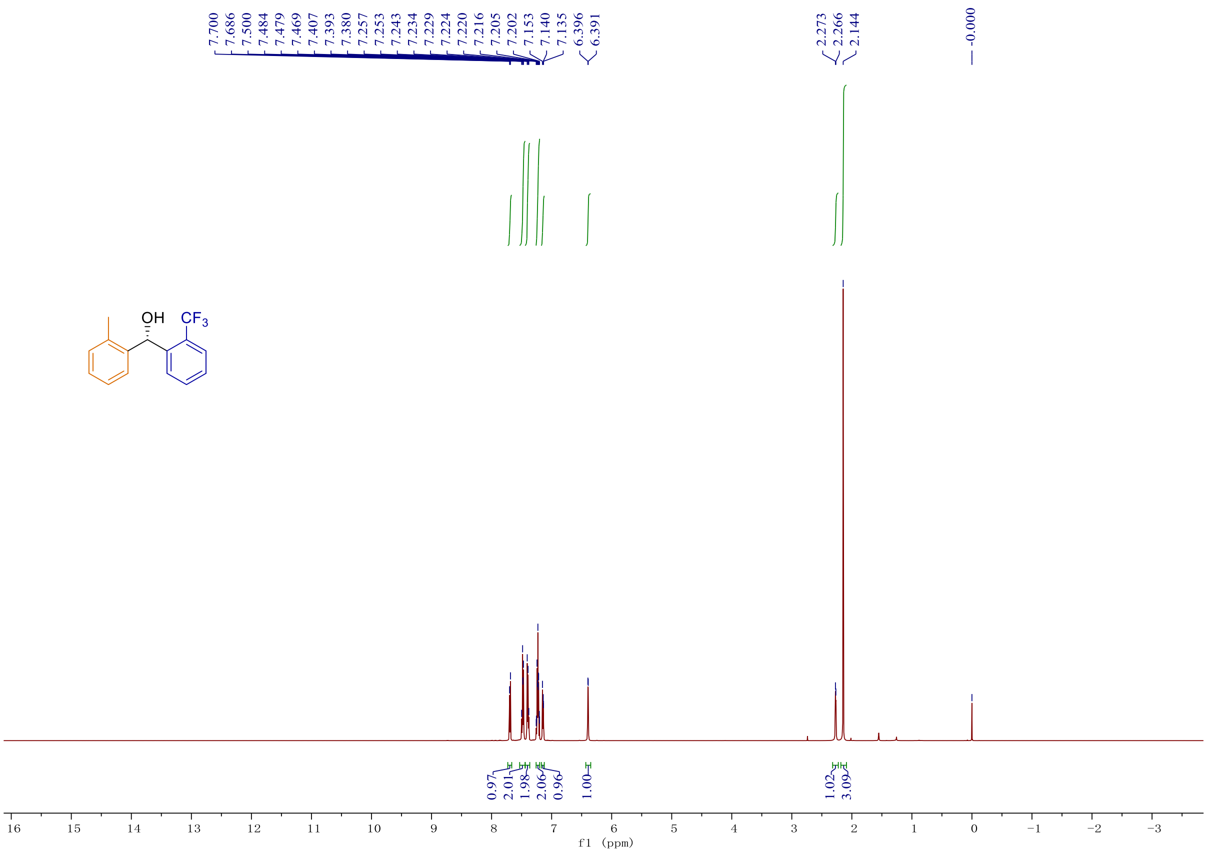

Supplementary Fig. 170 ^{1}H NMR (500 MHz, Chloroform-*d*) of (*S*)-o-tolyl(2-(trifluoromethyl)phenyl)methanol (4u).

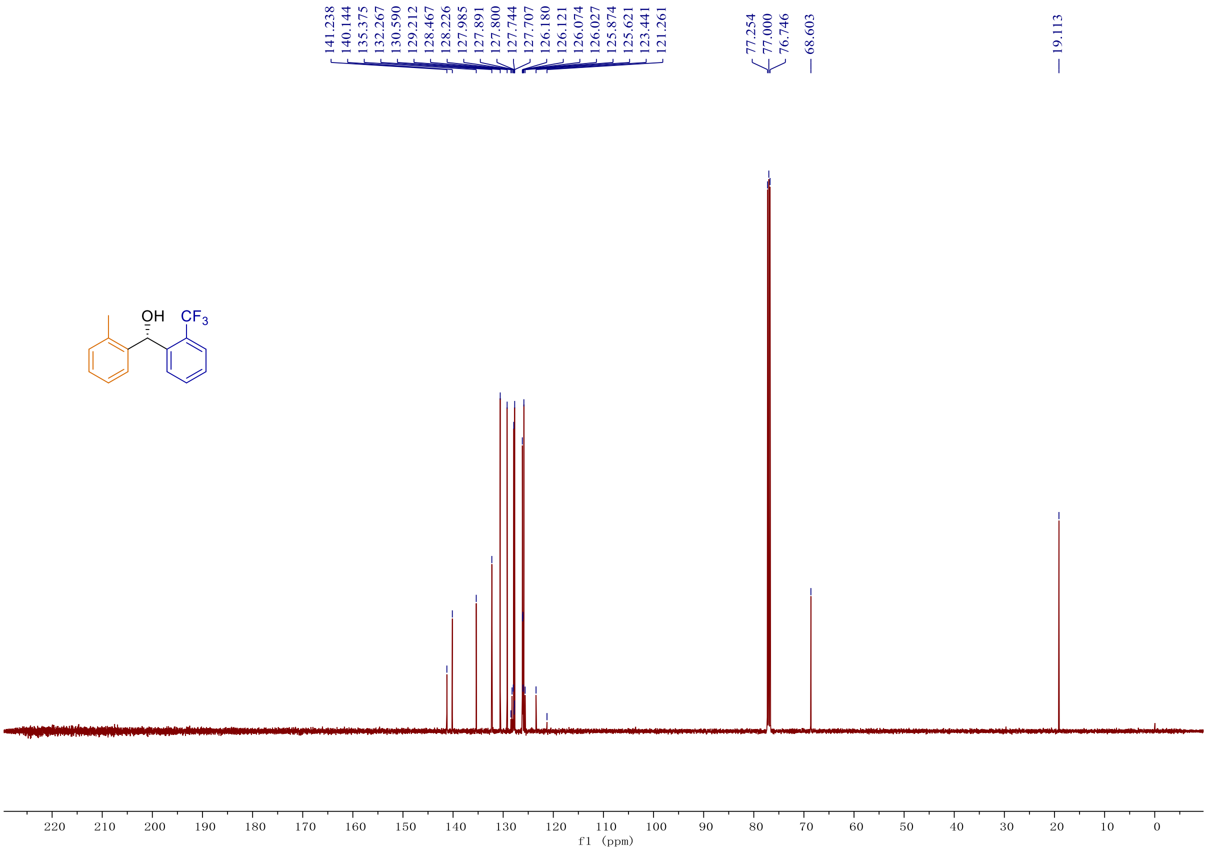

Supplementary Fig. 171 ^{13}C NMR (126 MHz, Chloroform-*d*) of (*S*)-o-tolyl(2-(trifluoromethyl)phenyl)methanol (4u).

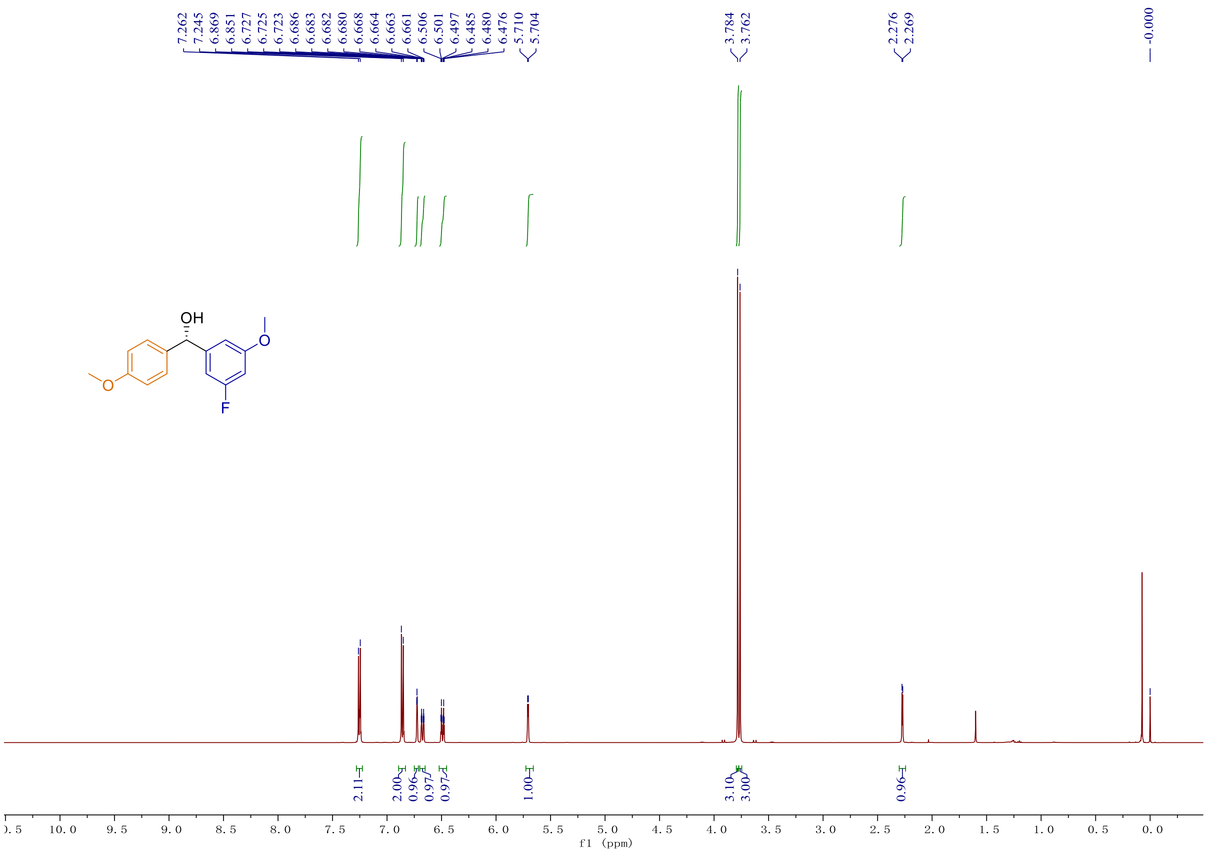

Supplementary Fig. 172 ^{1}H NMR (500 MHz, Chloroform-*d*) of (*S*)-(3-fluoro-5-methoxyphenyl)(4-methoxyphenyl)methanol (4v).

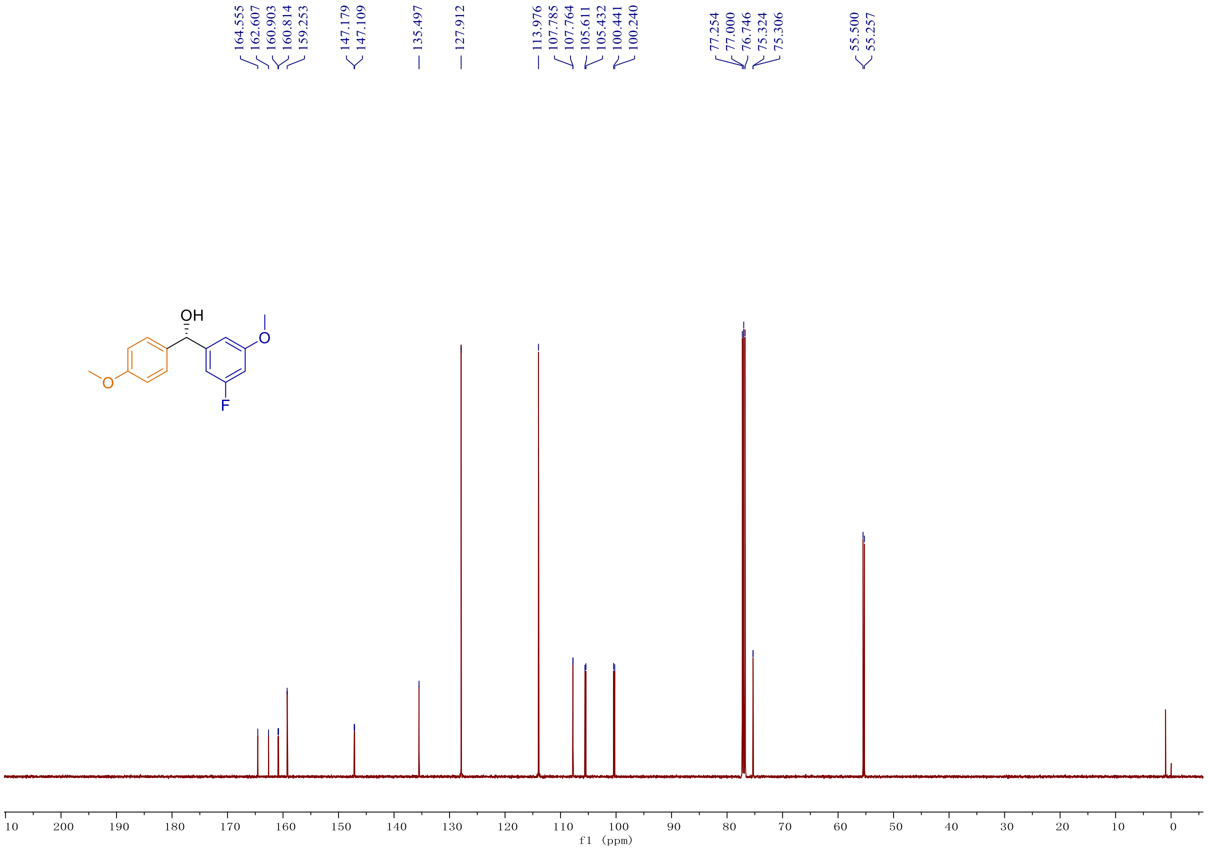

Supplementary Fig. 173 ^{13}C NMR (126 MHz, Chloroform-*d*) of (*S*)-(3-fluoro-5-methoxyphenyl)(4-methoxyphenyl)methanol (4v).

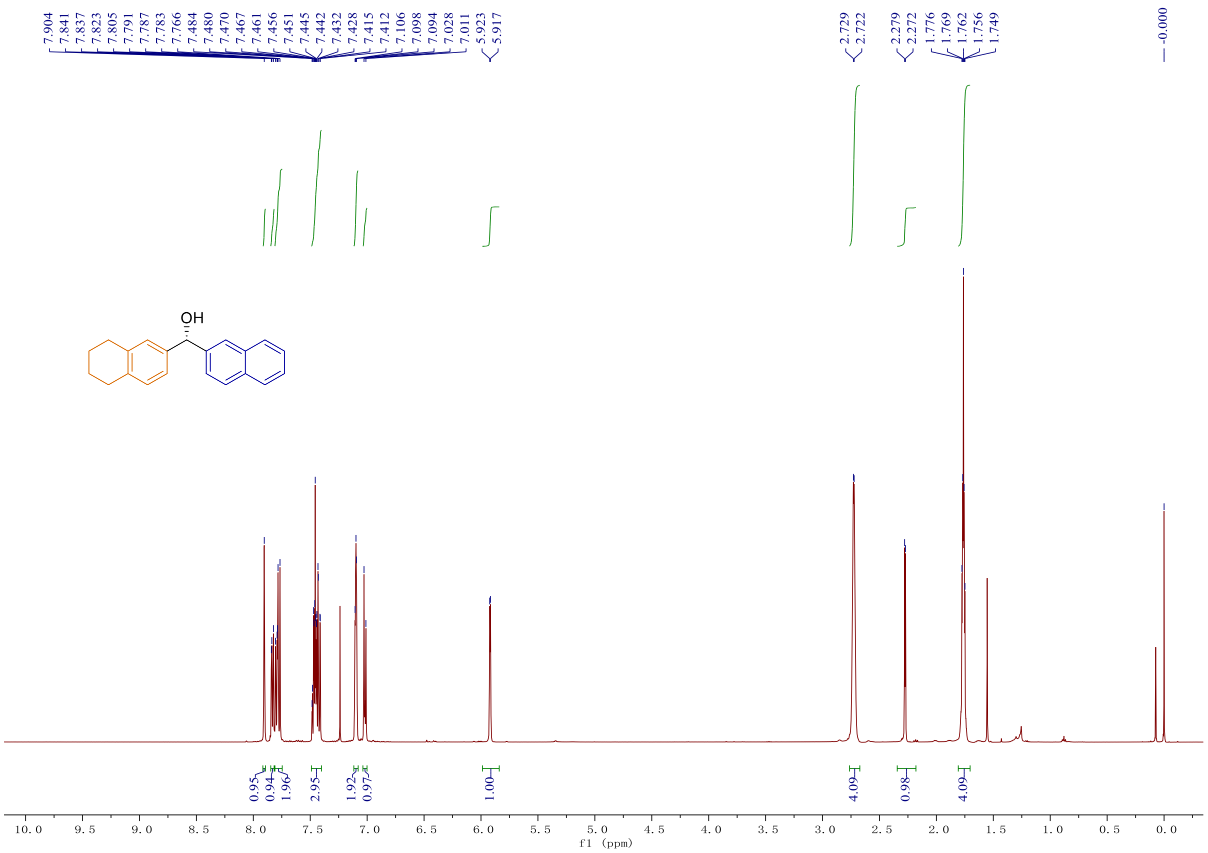

Supplementary Fig. 174 ^{1}H NMR (500 MHz, Chloroform-*d*) of (*S*)-naphthalen-2-yl(5,6,7,8-tetrahydronaphthalen-2-yl)methanol (4w).

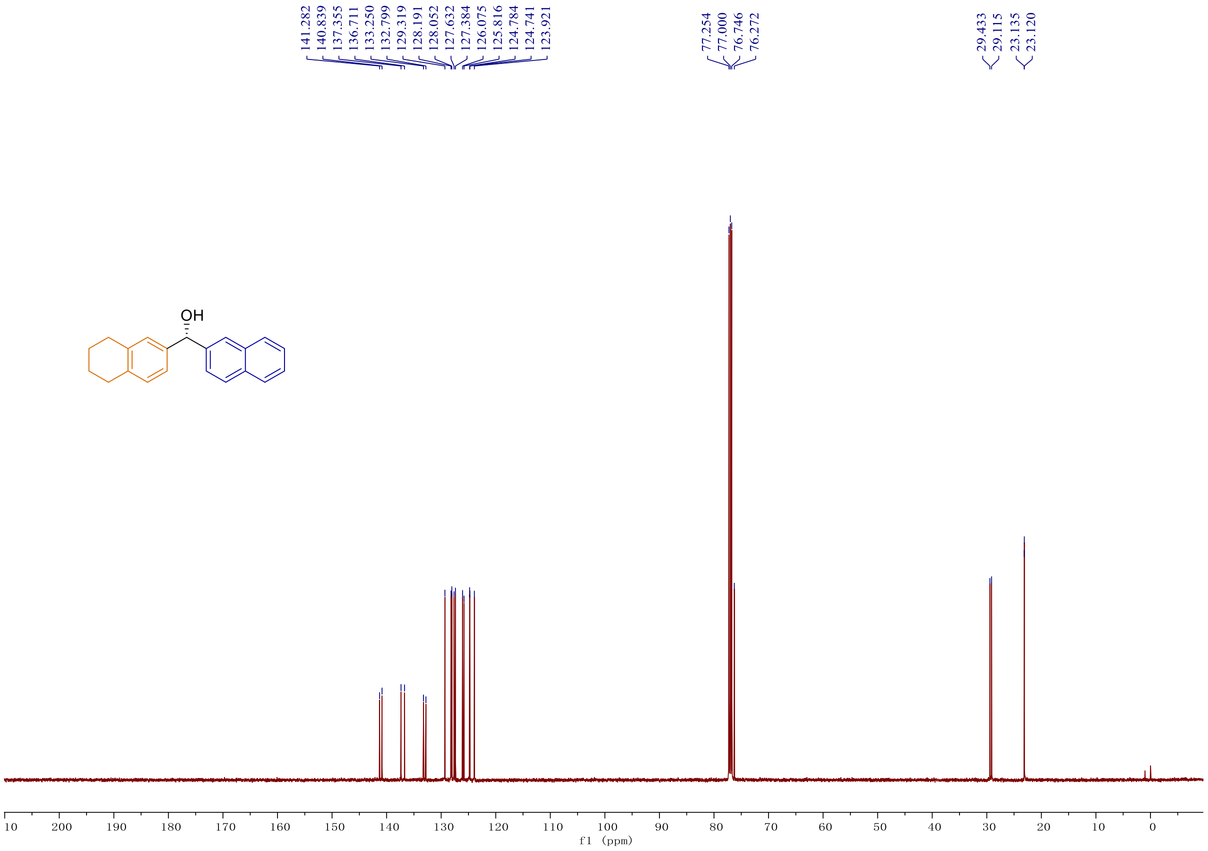

Supplementary Fig. 175 ^{13}C NMR (126 MHz, Chloroform-*d*) of (*S*)-naphthalen-2-yl(5,6,7,8-tetrahydronaphthalen-2-yl)methanol (4w).

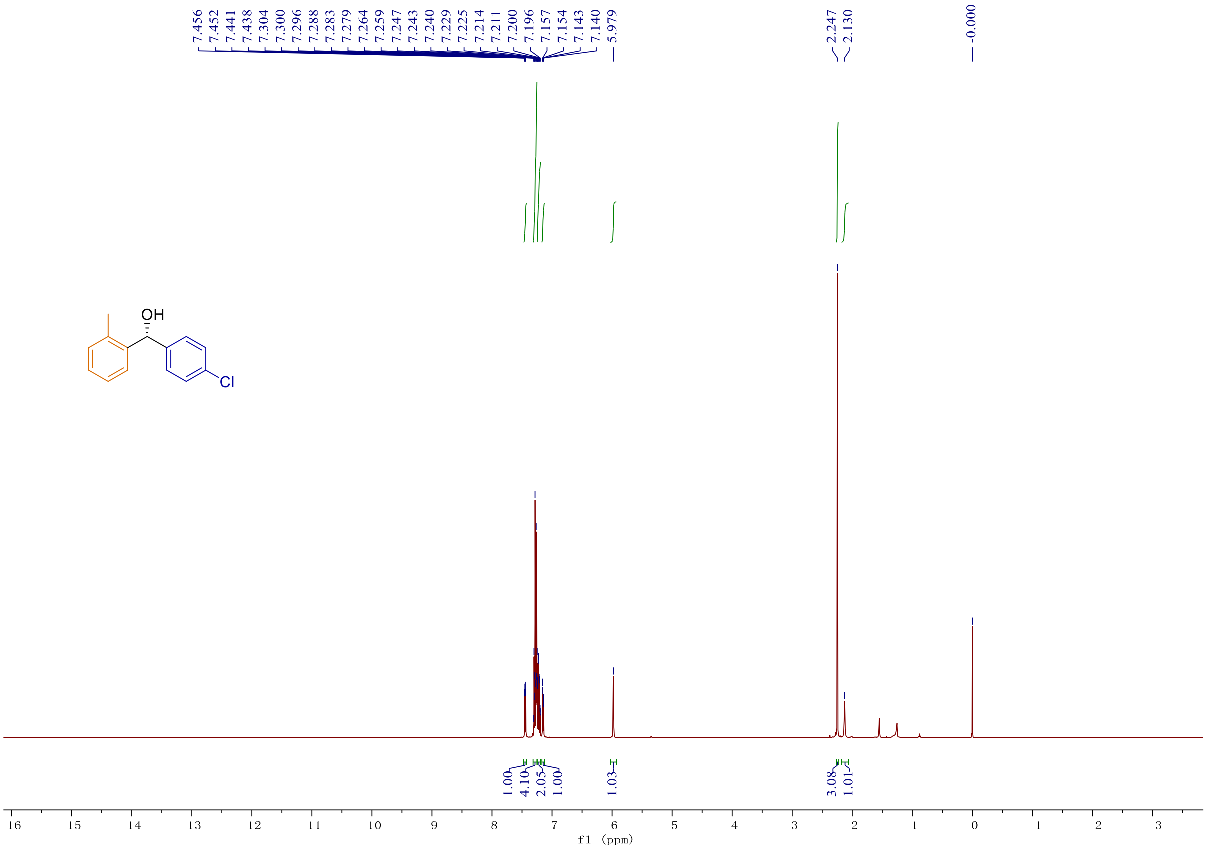

Supplementary Fig. 176 ^{1}H NMR (500 MHz, Chloroform-*d*) of (*R*)-(4-chlorophenyl)(o-tolyl)methanol (4x).

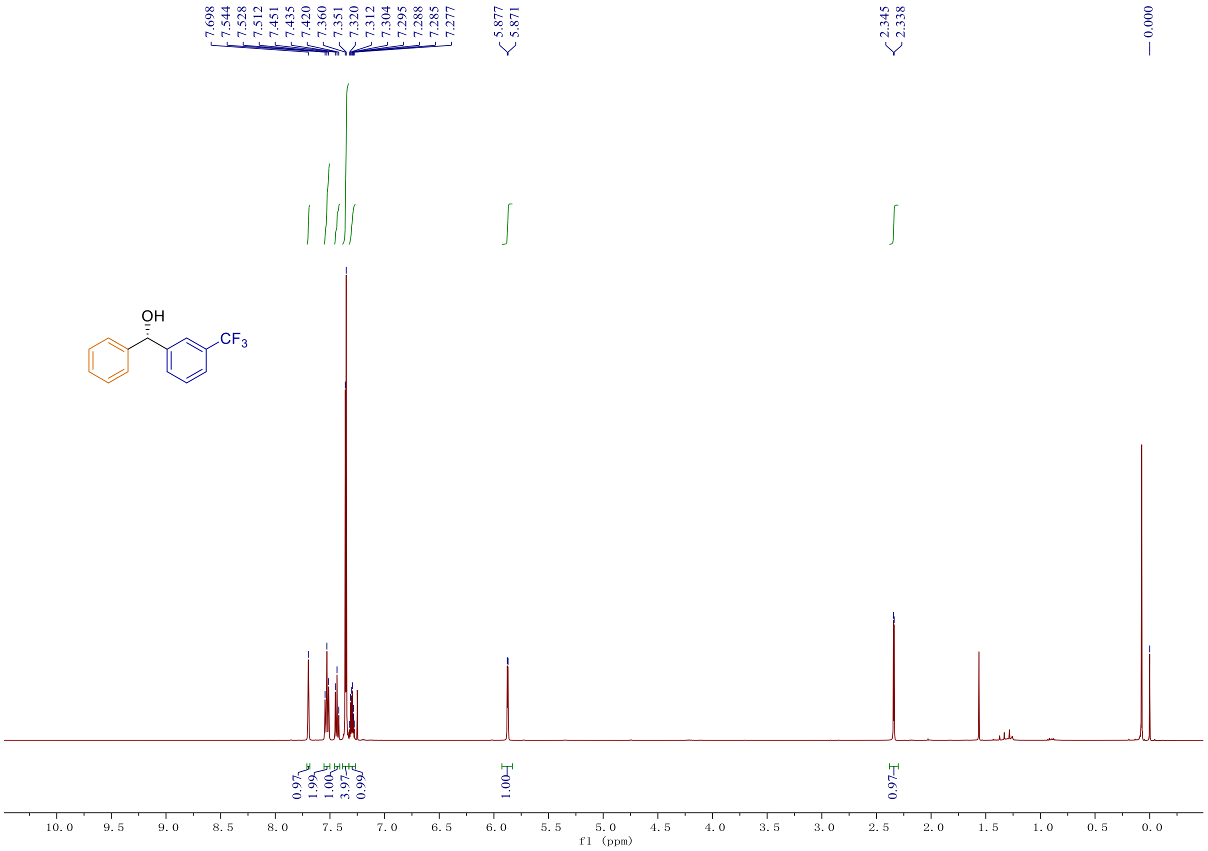

Supplementary Fig. 177 ^{1}H NMR (500 MHz, Chloroform-*d*) of (*S*)-phenyl(3-(trifluoromethyl)phenyl)methanol (4y).

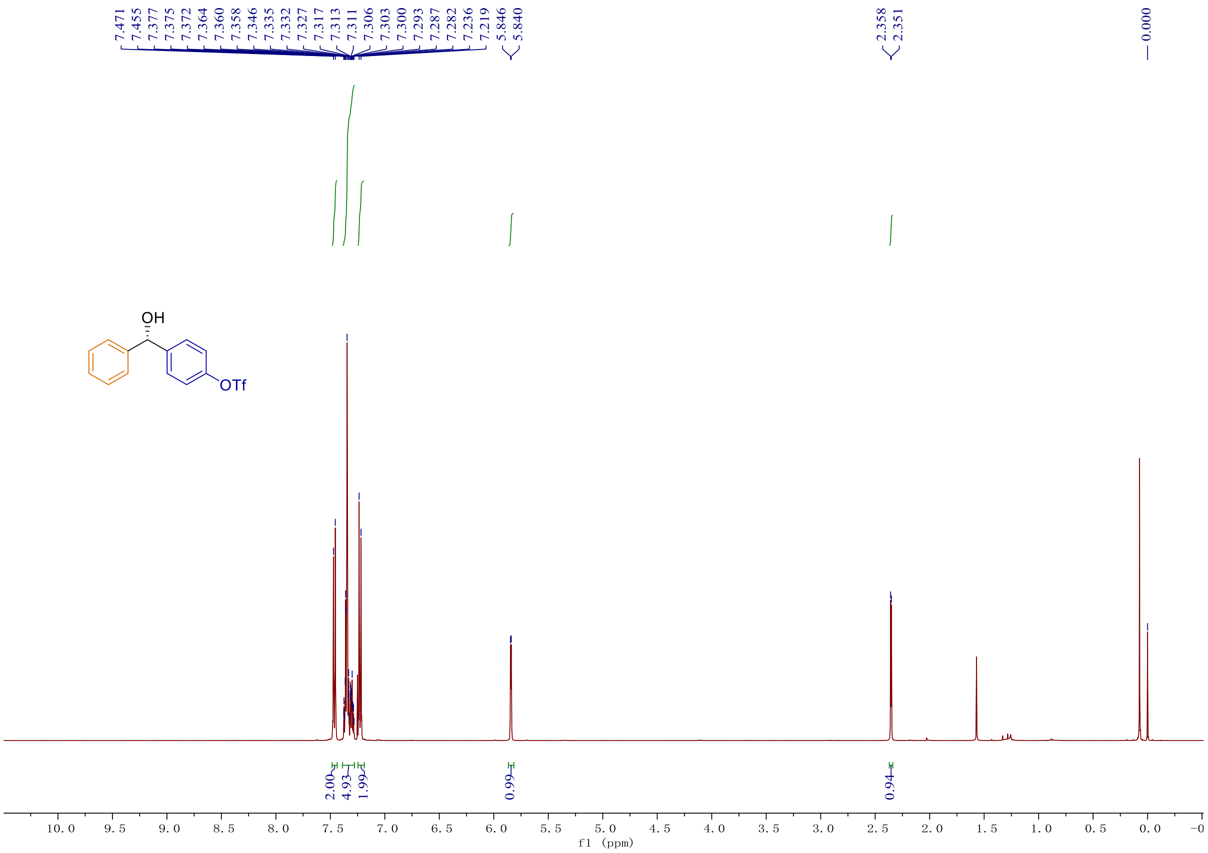

Supplementary Fig. 178 ^{1}H NMR (500 MHz, Chloroform-*d*) of (*S*)-4-(hydroxy(phenyl)methyl)phenyl trifluoromethanesulfonate (1z).

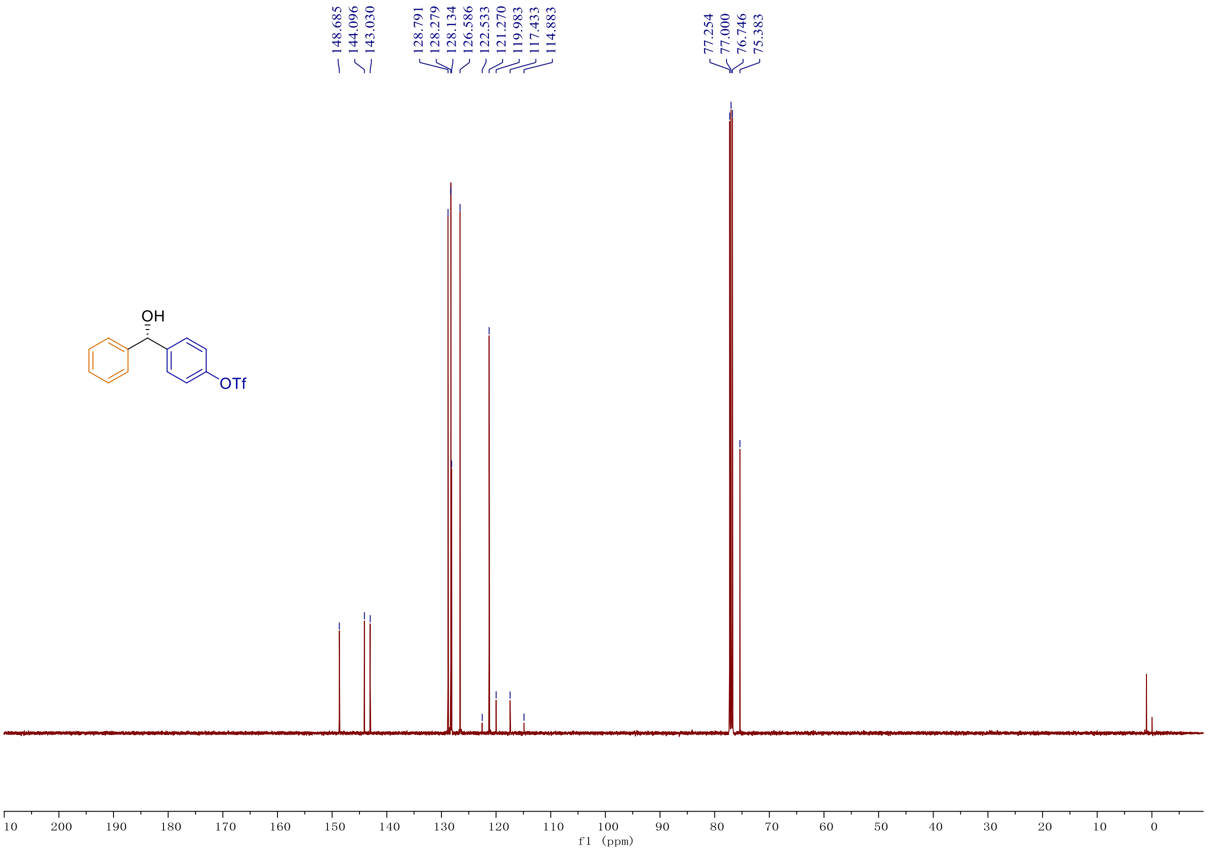

Supplementary Fig. 179 ^{13}C NMR (126 MHz, Chloroform-*d*) of (*S*)-4-(hydroxy(phenyl)methyl)phenyl trifluoromethanesulfonate (1z).

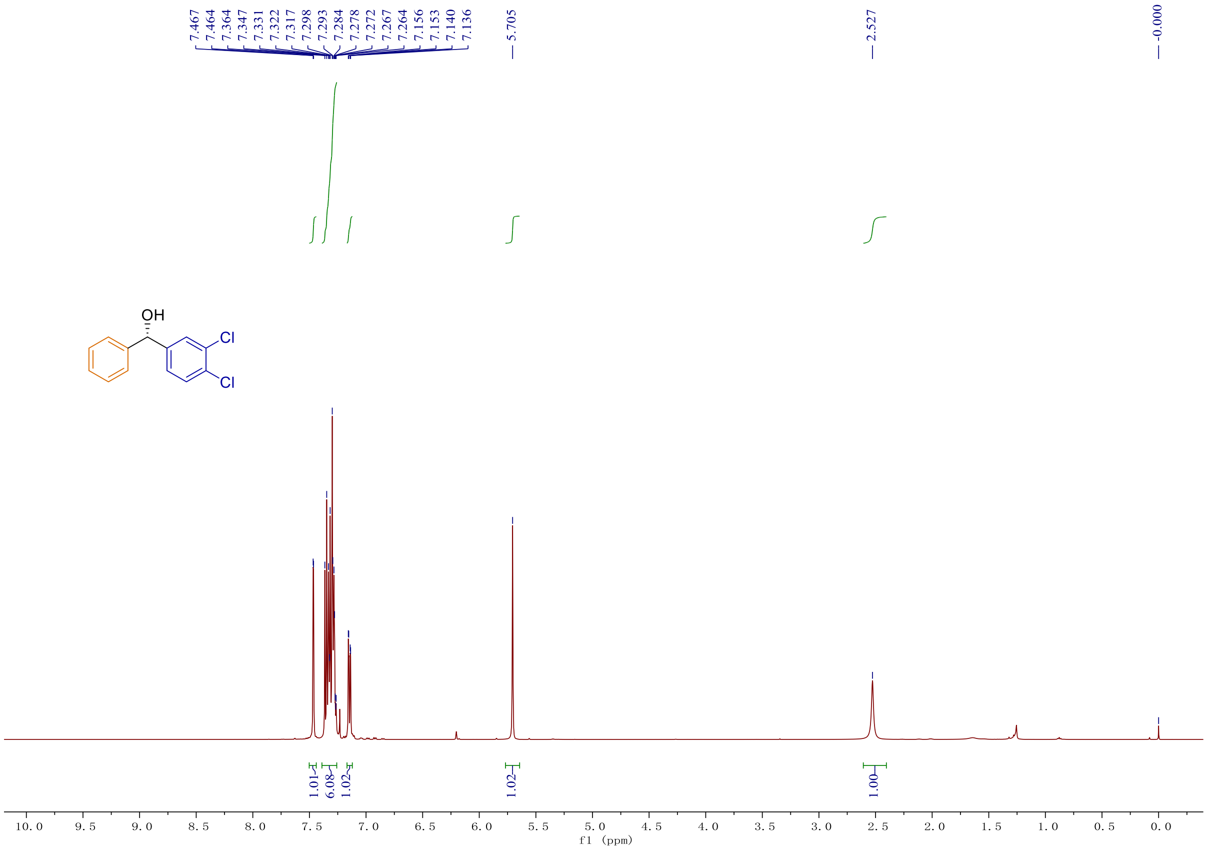

Supplementary Fig. 180 ^{1}H NMR (500 MHz, Chloroform-*d*) of (*S*)-(3,4-dichlorophenyl)(phenyl)methanol (4aa).

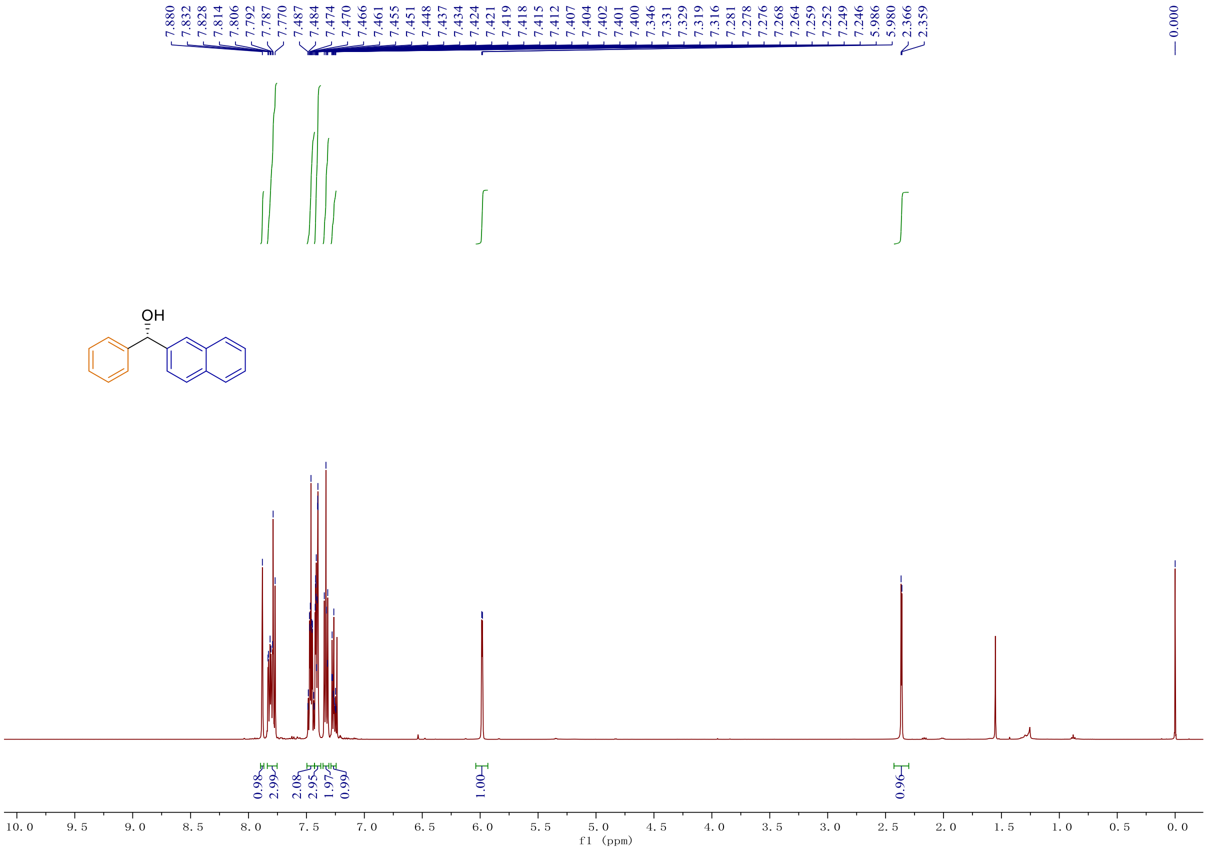

Supplementary Fig. 181 ^{1}H NMR (500 MHz, Chloroform-*d*) of (*S*)-naphthalen-2-yl(phenyl)methanol (4ab).

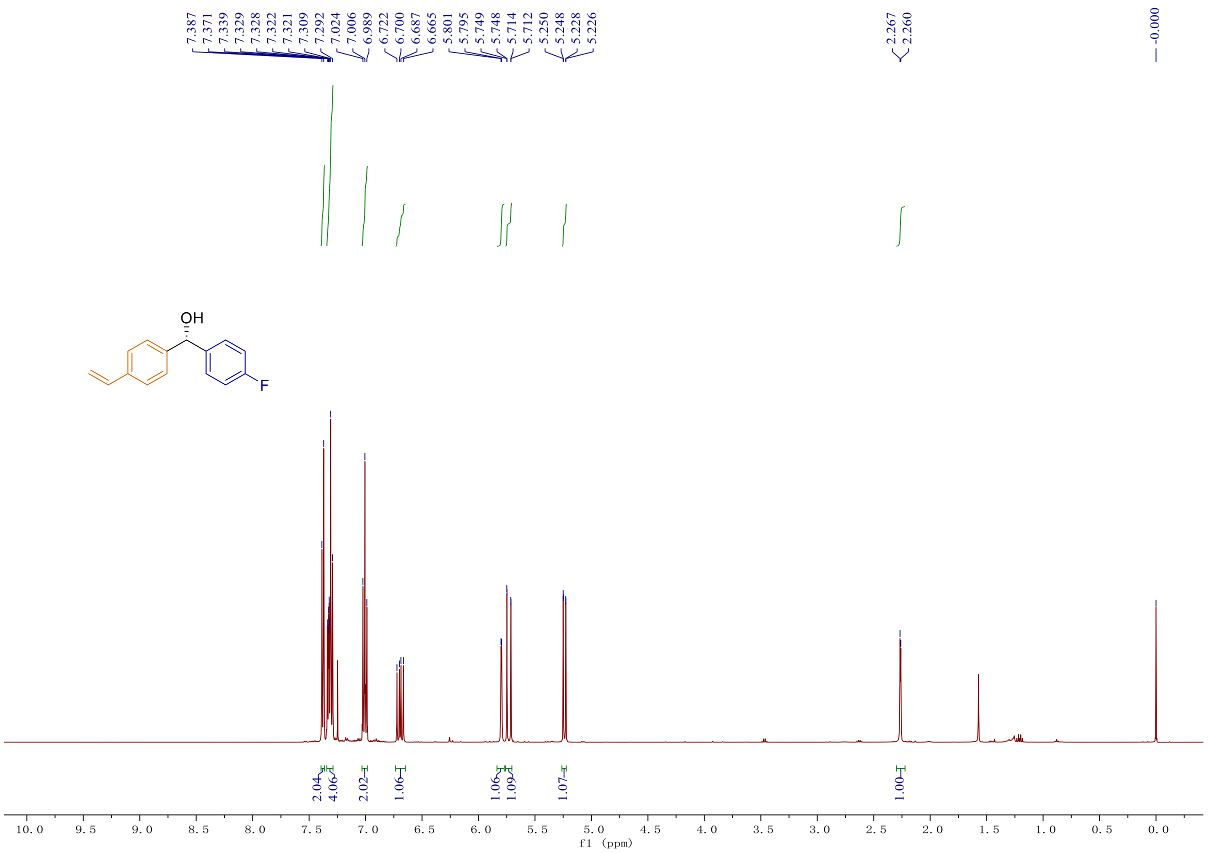

Supplementary Fig. 182 ^{1}H NMR (500 MHz, Chloroform-*d*) of (*S*)-(4-fluorophenyl)(4-vinylphenyl)methanol (4ac).

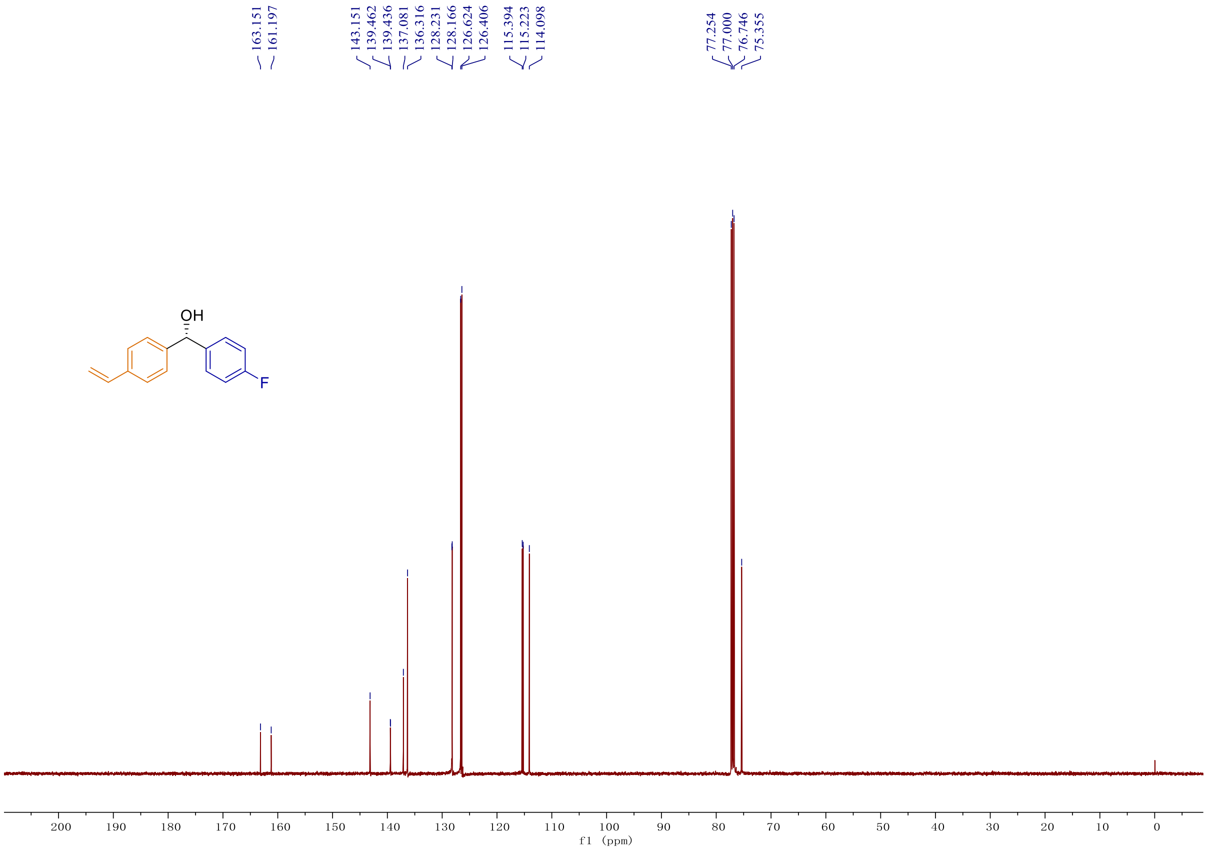

Supplementary Fig. 183 ^{13}C NMR (126 MHz, Chloroform-*d*) of (*S*)-(4-fluorophenyl)(4-vinylphenyl)methanol (4ac).

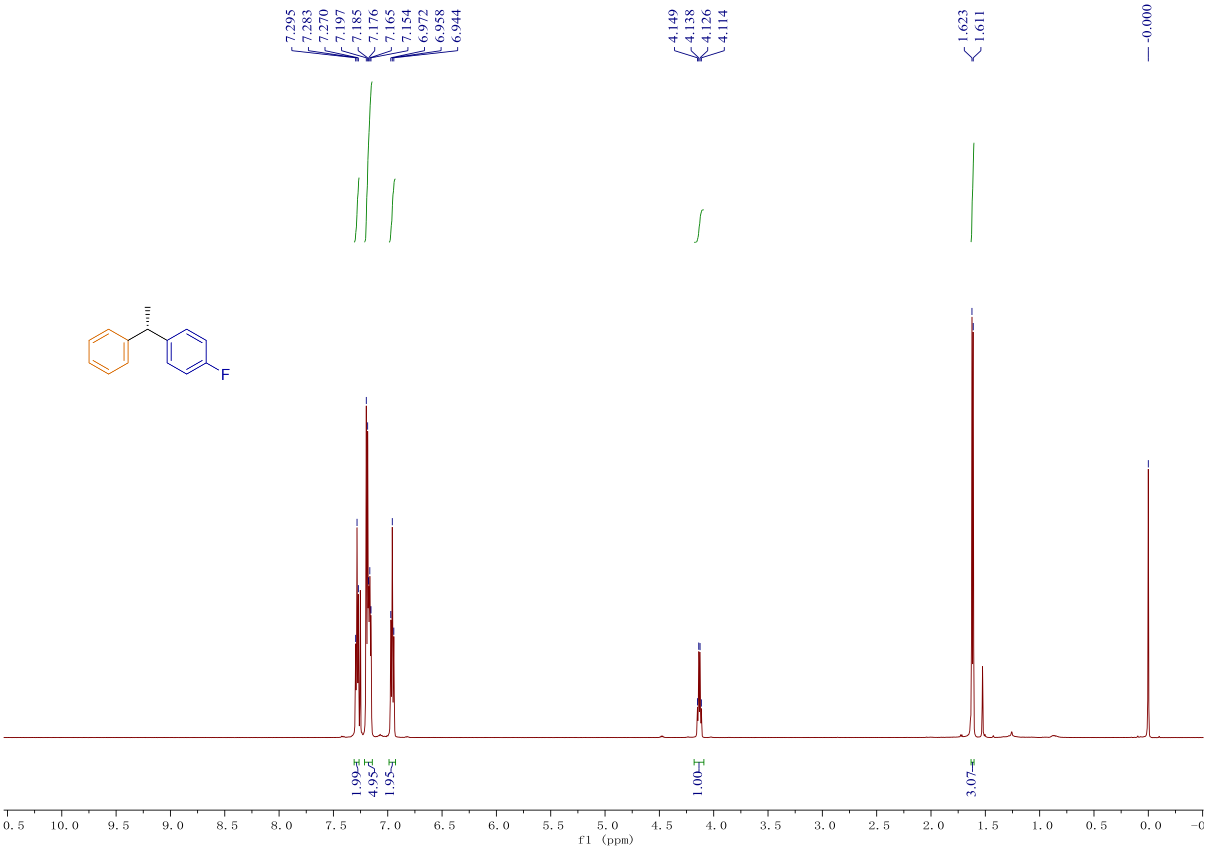

Supplementary Fig. 184 ^{1}H NMR (500 MHz, Chloroform-*d*) of (*S*)-1-fluoro-4-(1-phenylethyl)benzene (5a).

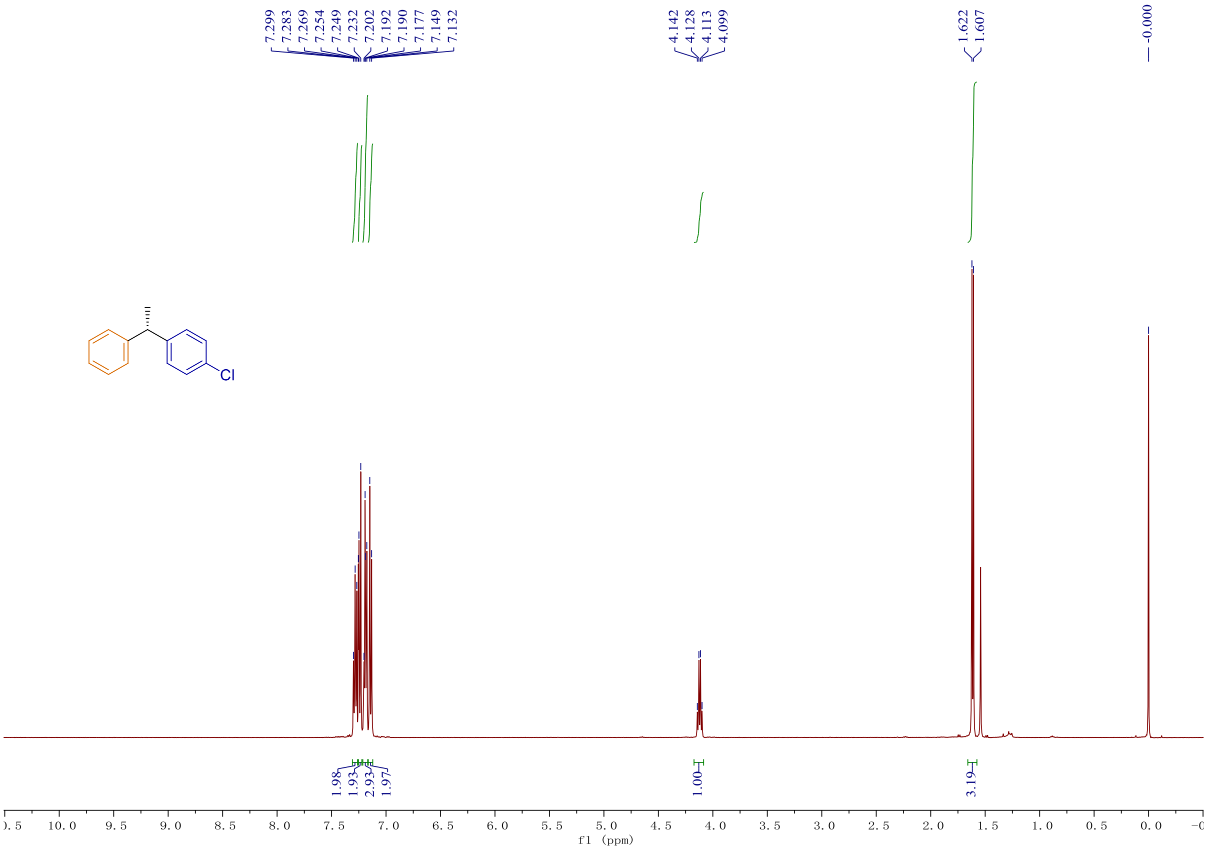

Supplementary Fig. 185 ^{1}H NMR (500 MHz, Chloroform-*d*) of (*S*)-1-chloro-4-(1-phenylethyl)benzene (5b).

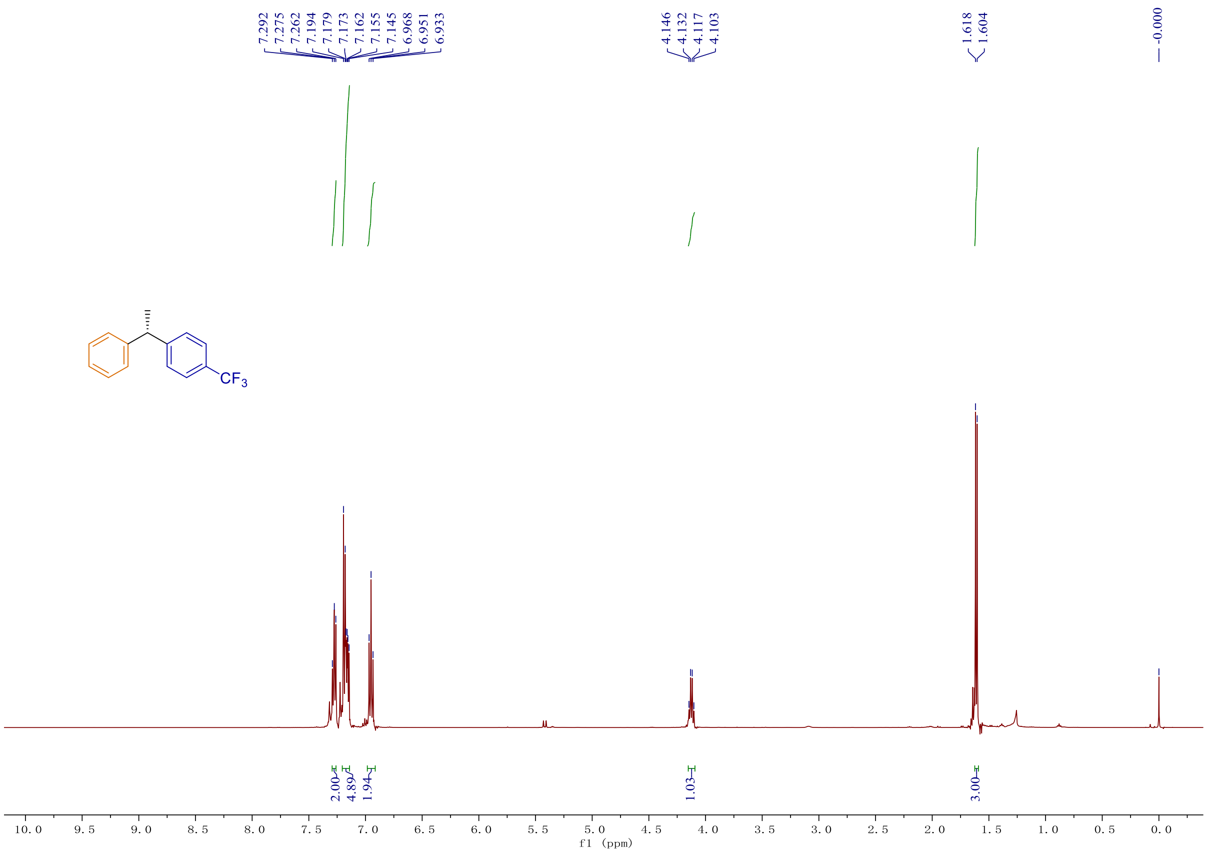

Supplementary Fig. 186 ^{1}H NMR (500 MHz, Chloroform-*d*) of (*S*)-1-(1-phenylethyl)-4-(trifluoromethyl)benzene (5c).

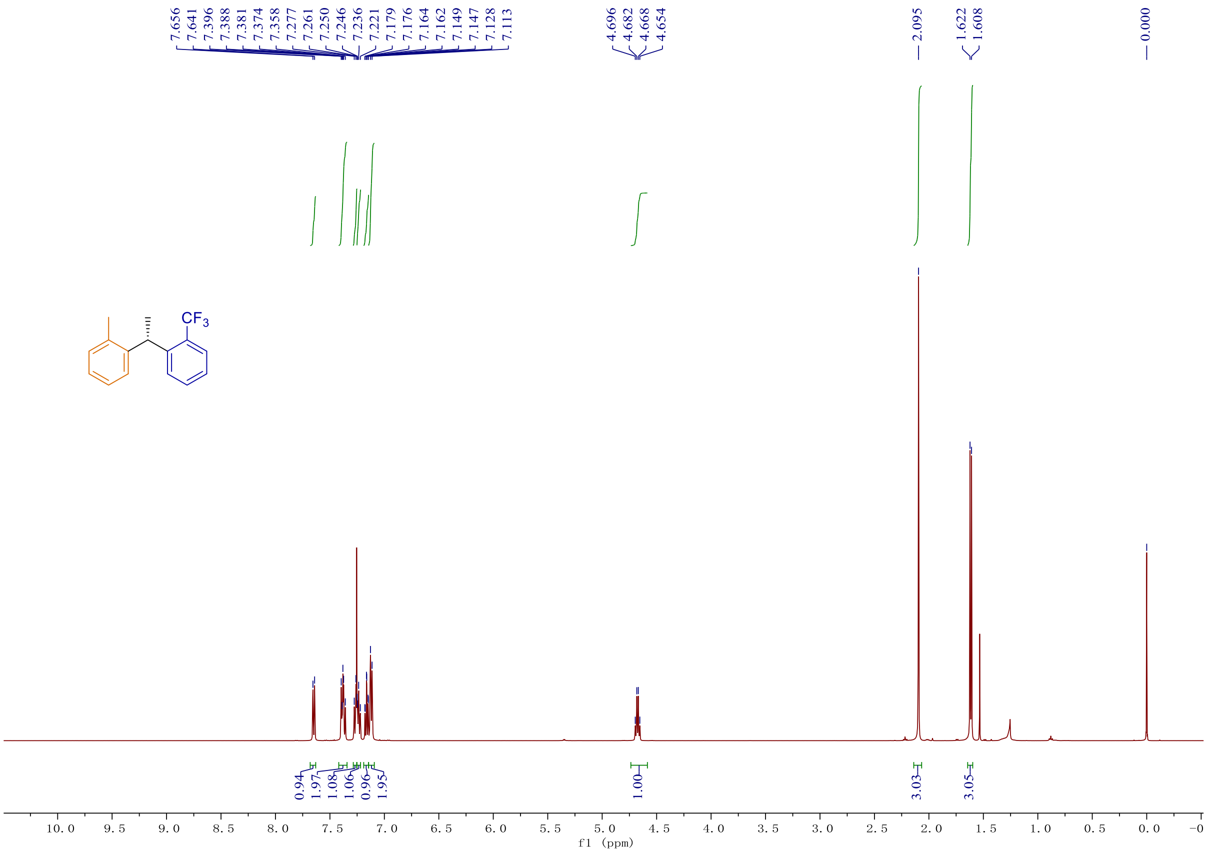

Supplementary Fig. 187 ^{1}H NMR (500 MHz, Chloroform-*d*) of (*S*)-1-methyl-2-(1-(2-(trifluoromethyl)phenyl)ethyl)benzene (5d).

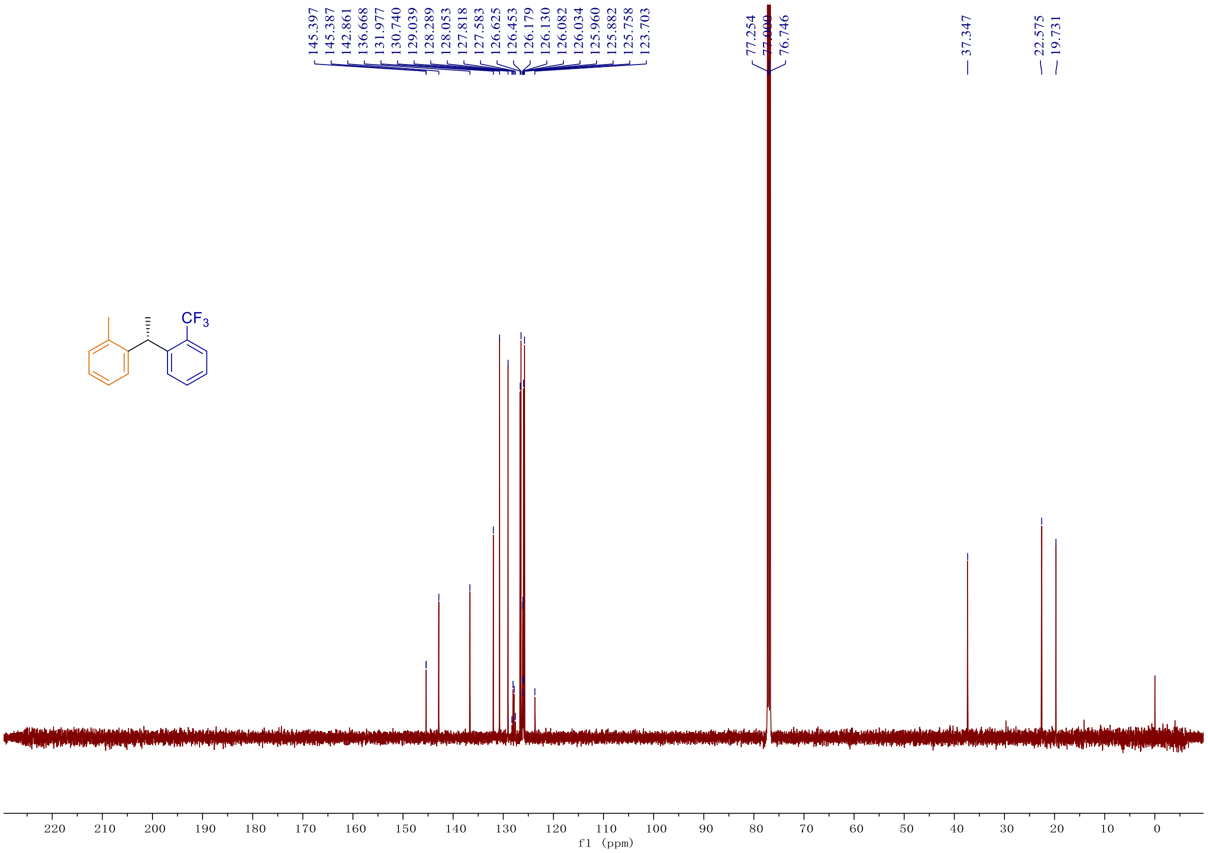

Supplementary Fig. 188 ^{13}C NMR (126 MHz, Chloroform-*d*) of (*S*)-1-methyl-2-(1-(2-(trifluoromethyl)phenyl)ethyl)benzene (5d).

Supplementary References

1. Sun, S. *et al.* (η^6-Polyarene)Mn(CO)$^{3+}$ Complexes as Manganese Tricarbonyl Transfer Reagents. A Convenient and General Synthetic Route to (arene)Mn(CO)$^{3+}$ Complexes. *Organometallics* **14**, 2613–2615 (1995).
2. Morley, J. A. & Woolsey, N. F. Metal arene complexes in organic synthesis. Hydroxylation, trimethylsilylation, and carbethoxylation of some polycyclic aromatic hydrocarbons utilizing η^6-arene−chromium tricarbonyl complexes. *J. Org. Chem.* **57**, 6487–6495 (1992).
3. Konovalov, A. I., Gorbacheva, E. O., Miloserdov, F. M. & Grushin, V. V. Ruthenium-catalyzed nucleophilic fluorination of halobenzenes. *Chem. Commun.* **51**, 13527–13530 (2015).
4. Kloetzing, R. J., Krasovskiy, A. & Knochel, P. The Mg-oppenauer oxidation as a mild method for the synthesis of aryl and metallocenyl ketones. *Chem. Eur. J.* **13**, 215–227 (2007).
5. Wakaki, T., Togo, T., Yoshidome, D., Kuninobu, Y. & Kanai, M. Palladium-Catalyzed Synthesis of Diaryl Ketones from Aldehydes and (Hetero)Aryl Halides via C–H Bond Activation. *ACS Catal.* **8**, 3123–3128 (2018).
6. Krach, P. E., Dewanji, A., Yuan, T. & Rueping, M. Synthesis of unsymmetrical ketones by applying visible-light benzophenone/nickel dual catalysis for direct benzylic acylation. *Chem. Commun.* **56**, 6082–6085 (2020).
7. Boehm, P., Roediger, S., Bismuto, A. & Morandi, B. Palladium-Catalyzed Chlorocarbonylation of Aryl (Pseudo)Halides Through In Situ Generation of Carbon Monoxide. *Angew. Chem. Int. Ed.* **59**, 17887–17896 (2020).
8. Chen, C., Liu, P., Luo, M. & Zeng, X. Kumada Arylation of Secondary Amides Enabled by Chromium Catalysis for Unsymmetric Ketone Synthesis under Mild Conditions. *ACS Catal.* **8**, 5864–5868 (2018).
9. Li, H., Xu, Y., Shi, E., Wei, W., Suo, X. & Wan, X. Synthesis of arylketones by ruthenium-catalyzed cross-coupling of aldehydes with arylboronic acids. *Chem. Commun.* **47**, 7880–7882 (2011).
10. Yang, Z., Zhu, X., Yang, S., Cheng, W., Zhang, X. & Yang, Z. Iridium-Catalysed Reductive Deoxygenation of Ketones with Formic Acid as Traceless Hydride Donor. *Adv. Synth. Catal.* **362**, 5496–5505 (2020).
11. Ushijima, S., Dohi, S., Moriyama, K. & Togo, H. Facile preparation of aromatic ketones from aromatic bromides and arenes with aldehydes. *Tetrahedron* **68**, 1436–1442 (2012).
12. Czyz, M. L., Taylor, M. S., Horngren, T. H. & Polyzos, A. Reductive Activation and Hydrofunctionalization of Olefins by Multiphoton Tandem Photoredox Catalysis. *ACS Catal.* **11**, 5472–5480 (2021).
13. Xie, J.-H. *et al.* Synthesis of spiro diphosphines and their application in asymmetric hydrogenation of ketones. *J. Am. Chem. Soc.* **125**, 4404–4405 (2003).
14. Li, W. *et al.* Design of Ru(II)-NHC-Diamine Precatalysts Directed by Ligand Cooperation: Applications and Mechanistic Investigations for Asymmetric Hydrogenation. *J. Am. Chem. Soc.* **142**, 7100–7107 (2020).
15. Gaykar, R. N., Bhunia, A. & Biju, A. T. Employing Arynes for the Generation of Aryl Anion Equivalents and Subsequent Reaction with Aldehydes. *J. Org. Chem.* **83**, 11333–11340 (2018).
16. Touge, T., Nara, H., Fujiwhara, M., Kayaki, Y. & Ikariya, T. Efficient Access to Chiral Benzhydrols via Asymmetric Transfer Hydrogenation of Unsymmetrical Benzophenones with Bifunctional Oxo-Tethered Ruthenium Catalysts. *J. Am. Chem. Soc.* **138**, 10084–10087 (2016).
17. Morikawa, S., Michigami, K. & Amii, H. Novel axially chiral phosphine ligand with a fluoro alcohol moiety for Rh-catalyzed asymmetric arylation of aromatic aldehydes. *Org. Lett.* **12**, 2520–2523 (2010).
18. Zheng, L.-S. *et al.* Synthesis of Ar-BINMOL Ligands by [1,2]-Wittig Rearrangement to Probe Their Catalytic Activity in 1,2-Addition Reactions of Aldehydes with Grignard Reagents. *Eur. J. Org. Chem.* **2013**, 748–755 (2013).
19. Yang, Y.-X. *et al.* Aryl bromides as inexpensive starting materials in the catalytic enantioselective arylation of aryl aldehydes: the additive TMEDA enhances the enantioselectivity. *J. Org. Chem.* **79**, 10696–10702 (2014).
20. Yang, L.-L. *et al.* Enantioselective Diarylcarbene Insertion into Si−H Bonds Induced by Electronic Properties of the Carbenes. *J. Am. Chem. Soc.* **142**, 12394–12399 (2020).
21. Greene, M. A., Yonova, I. M., Williams, F. J. & Jarvo, E. R. Traceless directing group for stereospecific nickel-catalyzed alkyl−alkyl cross-coupling reactions. *Org. Lett.* **14**, 4293–4296 (2012).
22. Yang, S., Tang, W., Yang, Z. & Xu, J. Iridium-Catalyzed Highly Efficient and Site-Selective Deoxygenation of Alcohols. *ACS Catal.* **8**, 9320–9326 (2018).
23. Matthew, S. C., Glasspoole, B. W., Eisenberger, P. & Crudden, C. M. Synthesis of enantiomerically enriched triarylmethanes by enantiospecific Suzuki−Miyaura cross-coupling reactions. *J. Am. Chem. Soc.* **136**, 5828–5831 (2014).
24. Harikrishnan, A., Sanjeevi, J. & Ramanathan, C. R. The cooperative effect of Lewis pairs in the Friedel−Crafts hydroxyalkylation reaction: a simple and effective route for the synthesis of (±)-carbinoxamine. *Org. Biomol. Chem.* **13**, 3633–3647 (2015).
25. Cao, D., Xia, S., Pan, P., Zeng, H., Li, C.-J. & Peng, Y. Light-driven MPV-type reduction of aryl ketones/aldehydes to alcohols with isopropanol under mild conditions. *Green Chem.* **23**, 7539–7543 (2021).
26. Ishida, S., Suzuki, H., Uchida, S., Yamaguchi, E. & Itoh, A. Nickel Catalyzed Intermolecular Carbonyl Addition of Aryl Halide. *Eur. J. Org. Chem.* **2019**, 7483–7487 (2019).
27. Kim, J. G. & Walsh, P. J. From aryl bromides to enantioenriched benzylic alcohols in a single flask: Catalytic asymmetric arylation of aldehydes. *Angew. Chem. Int. Ed.* **45**, 4175–4178 (2006).
28. Lee, C.-T. & Lipshutz, B. H. Nonracemic diarylmethanols from CuH-catalyzed hydrosilylation of diaryl ketones. *Org. Lett.* **10**, 4187–4190 (2008).
29. Frisch, M. J. *et al.* Gaussian 16 Rev. C03. (2016).

30. Neese, F. The ORCA program system. *WIREs Comput. Mol. Sci.* **2**, 73–78 (2012).
31. Neese, F., Wennmohs, F., Becker, U. & Riplinger, C. The ORCA quantum chemistry program package. *J. Chem. Phys.* **152**, 224108 (2020).
32. Weigend, F. & Ahlrichs, R. Balanced basis sets of split valence, triple zeta valence and quadruple zeta valence quality for H to Rn: Design and assessment of accuracy. *Phys. Chem. Chem. Phys.* **7**, 3297–3305 (2005).
33. Adamo, C. & Barone, V. Toward reliable density functional methods without adjustable parameters: The PBE0 model. *J. Chem. Phys.* **110**, 6158–6170 (1999).
34. Grimme, S., Ehrlich, S. & Goerigk, L. Effect of the damping function in dispersion corrected density functional theory. *J. Comput. Chem.* **32**, 1456–1465 (2011).
35. Grimme, S., Antony, J., Ehrlich, S. & Krieg, H. A consistent and accurate *ab initio* parametrization of density functional dispersion correction (DFT-D) for the 94 elements H-Pu. *J. Chem. Phys.* **132**, 154104 (2010).
36. Marenich, A. V., Cramer, C. J. & Truhlar, D. G. Universal solvation model based on solute electron density and on a continuum model of the solvent defined by the bulk dielectric constant and atomic surface tensions. *J. Phys. Chem. B* **113**, 6378–6396 (2009).
37. Chai, J. D. & Head-Gordon, M. Long-range corrected double-hybrid density functionals. *J. Chem. Phys.* **131**, 174105 (2009).
38. Lu, T. & Chen, Q. Independent gradient model based on Hirshfeld partition: A new method for visual study of interactions in chemical systems. *J. Comput. Chem.* **43**, 539–555 (2022).
39. Lu, T. & Chen, F. Multiwfn: a multifunctional wavefunction analyzer. *J. Comput. Chem.* **33**, 580–592 (2012).
40. Humphrey, W., Dalke, A. & Schulten, K. VMD: Visual molecular dynamics. *J. Molec. Graphics* **14**, 33–38 (1996).
41. Ess, D. H. & Houk, K. N. Distortion/interaction energy control of 1,3-dipolar cycloaddition reactivity. *J. Am. Chem. Soc.* **129**, 10646–10647 (2007).
42. Bickelhaupt, F. M. & Houk, K. N. Analyzing Reaction Rates with the Distortion/Interaction-Activation Strain Model. *Angew. Chem. Int. Ed.* **56**, 10070–10086 (2017).
43. Hirshfeld, F. L. Bonded-atom fragments for describing molecular charge densities. *Theoret. Claim. Acta (Berl.)* **44**, 129–138 (1977).
44. Lu, T. & Chen, F. Atomic Dipole Moment Corrected Hirshfeld Population Method. *J. Theor. Comput. Chem.* **11**, 163–183 (2012).
45. Marenich, A. V., Jerome, S. V., Cramer, C. J. & Truhlar, D. G. Charge Model 5: An Extension of Hirshfeld Population Analysis for the Accurate Description of Molecular Interactions in Gaseous and Condensed Phases. *J. Chem. Theory. Comput.* **8**, 527–541 (2012).
46. Abe, A. Conformational analysis and dipole moments of dialkyl esters of some simple dicarboxylic acids. *J. Am. Chem. Soc.* **106**, 14–19 (1984).
