# Supplementary material for: Asymmetric hydrogenation of 1,1-diarylethylenes and benzophenones through a relay strategy
Source: Nat Commun. 2023 Apr 15;14:2170. doi: 10.1038/s41467-023-37882-2 (PMC10105712; doi:10.1038/s41467-023-37882-2)
Supplement: Supplementary file 3 — Description of Additional Supplementary File [file 41467_2023_37882_MOESM3_ESM.docx]

File Name: Supplementary Data 1
Description: the cartesian coordinates of the optimized structures.

File Name: Supplementary Data 2
Description: the detailed data of electronic energy of relaxed scan of dihedral angles.
